# Russian young people’s subjective health evaluations, self-care practices, and therapeutic networks

**DOI:** 10.3389/fpsyg.2024.1247445

**Published:** 2024-02-23

**Authors:** Oxana Mikhaylova

**Affiliations:** ^1^Centre for Modern Childhood Research, HSE University, Moscow, Russia; ^2^Department for Social Institutions Analysis, HSE University, Moscow, Russia

**Keywords:** subjective health evaluations, self-care practices, therapeutic networks, young people, qualitative research

## Abstract

This study investigates the relationship between young people’s subjective health evaluations, self-care practices, and therapeutic networks using semi-structured interviews and the computerized qualitative thematic analysis capabilities of the MAXQDA software. In the summer of 2022, 41 Russian youths, ages 16 to 25, took part in this investigation. The major findings demonstrate that young people who had low health evaluations were more likely to conduct self-care with the intention of enhancing their health and to have mothers and other medical experts in their therapeutic networks. Furthermore, individuals who claimed that their health was inadequate engaged in more sports and took care of themselves even when they were ill. There was no association between the objectives of self-care practices, members of the therapeutic network, and self-care activities in terms of the type of health evaluation. Overall, it is possible to suggest that the practices and the structure of therapeutic networks are related to self-evaluated health, but quantitative study is needed to verify the causal relationship.

## 1 Introduction

Personal networks are related to individual self-care practices in young people (Hoffman et al., 2014; Littlecott et al., 2022). Self-care practices are the actions individuals use to maintain, monitor and manage personal health (Riegel et al., 2012). A person can impact others’ health behavior through 3 avenues: by (1) modeling health behavior and shaping the shared environment, (2) enacting behavior that promotes the relationship, and (3) employing strategies to intentionally influence the other person’s behavior (Huelsnitz et al., 2022). It is important to note that relationships between health behaviors and social networks are bidirectional; i.e., friend-seeking may be fostered by health behavior and friends could impact personal health behavior (Simone et al., 2018; Valente et al., 2023).

Social network analysis literature on health behaviors has largely focused on personal health behavior similarity with members of one’s social network and popularity analysis based on practiced health behaviors and has covered a limited selection of such behaviors, including smoking, drinking, and eating (Montgomery et al., 2020). From the aforementioned literature, it is known that young people in more established networks usually exhibit less unhealthy behaviors than those who have smaller networks (Montgomery et al., 2020; Palmer et al., 2022). Young people, including the vulnerable, report better health if they have more network resources, and health conditions may predict the number of social ties (Nevard et al., 2021; Hu and Cai, 2023). For example, the intensity of depressive symptoms is related to social network size (Negriff, 2019).

Furthermore, personal identity attributes have been shown to influence social network associations with self-care practices. For example, gender is an important mediator in physical activity, dietary practices, and drug-taking (Mario Arango-Paternina et al., 2022). Moreover, cultural identity (ethnic origin) is associated with practiced self-care (Craddock et al., 2016). Thus, parental influence on personal self-care practices could be mediated by race and ethnicity. Belonging to minorities (e.g., sexual) may mean healthier relationships with peers and does not predict involvement in unhealthy behaviors. This may be because friendships are more frequently reciprocated among members of a sexual minority, leading them to feel less stressed (Marshall et al., 2019). The effects of socioeconomic status are underreported for some health-related behaviors (Littlecott et al., 2022).

It is also important to understand who engages in self-care practices. For example, contraceptive self-care practices were observed more in those with parental emotional support, but peer emotional support did not significantly impact health self-care practices in young people (Mapanga and Andrews, 1995). However, the effects of health behaviors enacted by some could be mediated by others. For example, parental drinking may be less harmful to children if their peers are more welcoming and receptive (McCann et al., 2019).

Although self-care practices, therapeutic networks (the members of a personal network who are involved in health self-care activities; Smyth, 2005), and self-evaluated personal health (individual perception of their health condition; Dispenza et al., 2016) have been examined extensively separately and dyadically, the triad of these concepts has rarely been investigated (Winata and McLafferty, 2023). As such, there are currently a limited number of explanatory links to detail how self-care practices, therapeutic networks, and self-evaluated health are connected (Alsarrani et al., 2022). Furthermore, this topic is mostly researched in the elderly and populations with medically diagnosed conditions (Johansen et al., 2020), leaving young people without medically diagnosed conditions under-researched.

The current study aims to understand how health status subjective evaluations, self-care health practices, and therapeutic network composition are interrelated among young people (RQ). These concepts are explored in relation to gender and place of residence (which reflects socioeconomic status). Therefore, the research questions of this study are as follows:

How do young people evaluate their physical and mental health? (RQ1).

What healthcare practices do young people use? (RQ2).

Who constitutes their therapeutic networks? (RQ3).

How is health evaluation connected with personal self-care practices and therapeutic networks? (RQ4).

Understanding how therapeutic networks, self-evaluated health, and self-care practices are associated within young people can provide important insights for researchers interested in therapeutic networks and creating social network interventions to influence health-related behaviors, as well as guide future quantitative experimental research on this topic. This study is conducted in Russia, where the young people do not lead in self-care practices compared to other countries (Marconcin et al., 2021). Approximately half of Russians, according to poll data, characterize their health as good or very good (WCIOM, 2021). Self-care practices in this country are gender-specific; females tend to be more worried about their health and consult physicians more in comparison to males (Rostovskaya et al., 2021). In addition, access to healthcare differs across the country (Cook, 2015; Baidin et al., 2021), and people living in Russian regions usually have worse economic well-being than those living in Moscow (Voeykov and Anisimova, 2022). Therefore, it is reasonable to investigate health-related behaviors in Russia while accounting for gender and place of residence.

## 2 Methods

### 2.1 Participants

#### 2.1.1 Recruitment and sampling strategies

Study participants were recruited using quota sampling via three social network accounts belonging to researcher. I searched for young people aged 16–25, who were ready to discuss human autonomy and life choices. These interviews were part of project conducted at HSE University to study young people autonomy and volitional functioning in various life spheres. Age, gender, and place of residence diversity were monitored during sampling. The decision to stop sampling once 41 participants had been recruited was based on sample diversity and data quality.

### 2.2 Ethical considerations

All ethical standards set by the HSE University Ethics Committee are met by this study. Further information on the university’s policies can be found on this website: https://www.hse.ru/en/org/hse/irb/. Each participant gave written informed consent and emailed the researcher a photocopy of their completed consent form. During the consent process, young people were reminded that they could leave the interview at any time if they felt that it was necessary. We did not collect consent forms from parents since all participants were over the age of 14 and it is not mandatory in the Russian Federation to request for consent forms from parents if a person is over the age of 14.

### 2.3 Data collection

Forty-one young people participated in semi-structured interviews conducted online in Moscow, Russia, in the summer of 2022. Interviews were gathered by three female researchers with backgrounds in psychology and sociology. Guides for the interview included questions about individual autonomy and life choices and were not exclusively devoted to health, for instance: “Let me divide your life into spheres. Please tell us about each of these areas. What choices have you had to make in each of them over the past year?... and in the field of health?.” This likely makes the study more ecologically valid as the narratives on health that are analyzed were almost unsolicited. Health-related questions only regarded previous year choices made in the health sphere (participants needed to tell interviewers about them and it was necessary to talk about autonomy regarding self-care practices). The interviews have been conducted in Russian, the guide was written in Russian, and the data was analyzed in Russian. The proofreader reviewed this paper’s author’s translation of the study results into English.

### 2.4 Data analysis

#### 2.4.1 Thematic analysis

The data were analyzed in MAXQDA https://www.maxqda.com/ using guidelines for thematic analysis by one researcher (Braun and Clarke, 2006). This required researcher to read all transcripts multiple times (close reading) and find those via first and second-level coding (De Wet and Erasmus, 2005) that were related to (1) self-evaluated health (bad or good; mental health or physical health), (2) self-care practices (types of practices; goals), and (3) therapeutic networks (members; their functions). A total of 1823 s-level codes were produced. The codes with participant direct citations were considered first-level codes. Second-level codes used the researcher’s words to summarize the information provided by participants (citations – first-level codes). Text segments were categorized using multiple codes when they contained various thoughts and behaviors that the study participants reported. Each participant’s self-assessment determined the codes that were assigned. For example, if a person had mentioned that he felt awful, I would have coded this text segment as having “bad subjective health evaluation.” The Appendix contains the code system and links to the procedures of analysis carried out in this study and detailed in the program handbook used to complete the analytical steps.

#### 2.4.2 Epistemological stance

The coder held a constructivist stance, meaning that personal narratives were treated as social constructions around the topics of health self-evaluation, self-care practices, and therapeutic networks. During the analysis, I focused on linguistic constructions and regarded the discourses provided by participants as their visions of social reality (Willig, 2012). I acknowledge that my social position being White, age 26, having higher education, and living in the capital city may have influenced my interpretations. This means that, for instance, I could have demonstrated a deeper level of analysis in cases where I coded the fragments of participants with similar demographics and comparable personal health evaluations, practices, and therapeutic networks to myself. I repeatedly discussed the findings with my colleagues who work at the Center for Childhood Studies (HSE University) in order to address the subjectivity of this research.

## 3 Results

### 3.1 Demographic information

Of the 41 young people who participated in this study, their ages ranged from 16 to 25 years (*M* = 20.88, SD = 2.59), with 21 identified as female and 20 as male. Regarding place of birth, which was used as a proxy for socio-economic status, 17 were living in Moscow. The remaining 24 resided in 13 other cities.

### 3.2 Self-evaluated physical and mental health

Thirty-two informants evaluated their health as bad and 20 argued that it could be considered great (statistics on code frequencies by gender and place of residence are presented in the Appendix). There were no participants that argued that their health is neutral. These figures suggest that some young people concurrently believe their health to be bad and great. There was no difference in terms of the number of health types (physical or mental) mentioned in subjective health evaluations.

Among males, there was almost no difference in good or bad health evaluations. Thirteen argued that they consider it bad and 11 considered it great. The solid difference in health evaluations was among females, which reflects the general tendency in the sample: 19 female informants argued that their health is bad and only 9 considered it to be great. Only 1 female participant did not argue anything about her health.

### 3.3 Health care practices

The health care practices that informants participated in included sports, going to the doctor if needed, caring after self while being ill, eating well, drinking alcohol, working, being kind to self and others, volunteering, smoking, becoming health professionals to know how to care about self and others, self-talking, doing regular checkups, reading, talking with significant others, avoiding harmful recreational activities, art, hobbies, caring after pets, self-harm, playing computer games, esoteric behavior, taking drugs, self-monitoring for health problems and resting.

Gender differences used were noted in terms of the type of practice, e.g., becoming a health professional was more specific to females, however, drinking and smoking were more common for males (statistics on code frequencies by gender and place of residence are presented in the Appendix). As for regional differences, Muscovites more often used work to self-care, participated in sports, ate well, cared for themselves while ill, and smoked and drank alcohol. In comparison, regionals more often engaged in kindness toward themselves and others.

The goals of these activities included: help, maintenance, improvement, self-analysis, and decision-making. There were no gender-specific differences identified in practice goals. However, an association was found between goals of self-care and place of residence. For instance, regionals mentioned more often that the goal of their self-care was to achieve improvement.

### 3.4 Therapeutic networks members

Informants identified over 5 agents in their life that helped them with their health. Most frequently mentioned were health professionals (82). These included 46 physical health professionals and 48 mental health specialists. Regarding health professionals, the most frequent doctor discussed was a dentist. Informants told many stories about how they went to the dentist to improve their teeth health. Mother was the second most frequently mentioned (42), followed by father (23) friend (14), teacher (13), romantic partner (10), media (9), peer (8), work colleague (6), grandparents (5), stranger (3), and sibling (2). The reconstruction of the same narrative demonstrated that the mother, father, and health professional are the main triad of helpers interacting with each other in a young person’s therapeutic network (see Supplementary Figure S1 for the network of helpers visualization; Supplementary Figure S2, shown in the Appendix, describes their functions).

Females cited other people as self-care practice assistants almost twice as much as males (see the Appendix for exact figures). Furthermore, only females argued that their self-care practices involved siblings, strangers, and grandparents. Females also cited the media, health professionals, colleagues, and fathers more frequently than males. Gender differences were less pronounced or suggested in equal frequency for mothers, friends, peers, teachers, and romantic partners. Regarding residence, it was observed that regionals were more likely to have teachers and friends in their therapeutic networks. Other location differences were not found.

### 3.5 Subjective health evaluation, self-care practices, and therapeutic networks

#### 3.5.1 Subjective health evaluation and therapeutic network composition

In terms of health type (physical or mental), there was found to be no association with therapeutic network members. However, a connection was found between health evaluation (good or bad) and therapeutic network members. Informants who argued that their health is bad were more likely to have their mothers and health professionals in their therapeutic networks.

#### 3.5.2 Therapeutic network composition and self-care practices

Those who did sport commonly had health professionals in their self-care networks, consulted the doctor, took care of themselves when ill, and were more likely to become health professionals.

#### 3.5.3 Subjective health evaluation and self-care practices

Looking at intersections between type of health (mental or physical) and self-care practices goals suggests that there is no difference in goal practices by type of health. Nevertheless, there is an association between subjective health evaluation and practices. If health was evaluated as bad, informants were more likely to use help and maintenance-oriented practices. Relevant figures are presented in the Appendix.

## 4 Discussion

This paper investigated associations between health self-evaluations, self-care health practices used, and therapeutic network composition among Russian-speaking young people. These concepts were also explored in relation to gender and place of residence.

It was discovered that most young people evaluate their health as bad, but that sometimes their poor health evaluations coincided with highly positive ones. Females were more prone than males to characterize their health as bad, which corresponds with previous studies on young people (Joffer et al., 2019; Shi et al., 2022). Regional differences in health evaluations were revealed, with Muscovites perhaps being more negative about their health than regionals. However, the sample is too small to state this without any doubt. Young people spoke equally about mental and physical health, regardless of gender and place of residence. This may be interpreted as these components of health being equally important to them.

In addition, was discovered that the most popular practice of self-care is doing sport. This practice was especially common among males. Such results correspond with literature on self-care practices showing that exercising is one of the most popular self-care practices and that it is more common in males than females (Lukman et al., 2020; Osokpo and Riegel, 2021). Most informants stated that they use self-care practices for help and maintenance, and the goal of health improvement was less commonly identified. No marked gender differences in goals were noticed, however, there were small differences by place of residence, with regionals being more likely to use self-care for self-improvement. This may be related to differences in therapeutic cultures and values existing in various Russian regions.

Health professional was the most frequently mentioned self-care practices agent. This suggests that health system workers are a useful authority for young people (Riegel et al., 2021) and such findings correspond with literature highlighting that self-care activities must be planned to be regular and discussed with healthcare workers (Fiske et al., 2020; Świątoniowska-Lonc et al., 2020). Furthermore, it was revealed that health professional actions in therapeutic networks are related commonly to those of fathers and mothers. This indicates that there is a triad of self-care that is usually around young people that affects their well-being. Regarding gender, as in previous studies made in Russia and other countries, females had more established therapeutic networks than males (Rodríguez-Madrid et al., 2019). The network’s members also differed in terms of participant place of residence. Regionals were more prone to have teachers and friends in their networks. Such a finding could be due to multiple reasons and needs to be further investigated. On the one hand, it could be explained by the customs of social support that vary in Moscow and the regions. On the other hand, it could result from having had to move from a home city, which was characteristic of some young people who currently reside in Moscow. It is possible that they left their teachers and friends at home and did not form new connections with friends in the city, which may explain why they less frequently identified these people in their therapeutic networks.

Finally, it was discovered that informants who evaluated their health as worse were more prone to have mothers and health professionals in their therapeutic networks, and practice self-care with the goal of helping themselves. Also, those who argued that their health is bad practiced more sports, and took care of themselves while ill. In terms of type of health evaluation, no association was found with therapeutic network members, goals of self-care practices, and self-care activities. Overall, it could be argued that self-evaluated health is associated with therapeutic network composition and practices, but more research is needed to determine causality.

### 4.1 Strengths and limitations

The primary strength of the work lies in its use of the methodology included within the MAXQDA program for computerized qualitative data analysis. The application’s manual offers explicit instructions for reproducing the code intersection tables and code matrices utilized in this investigation. As a result, the process used to analyze the study’s data is transparent and reproducible on the other samples. Nonetheless, it is essential to take into account the several limitations of the present study as well. The findings cannot be generalized to all young people (worldwide) but may ring true for Russian young people since the sample included participants from different regions. In addition, it should be highlighted that this study investigated perceptions, meaning that evaluations of the analyzed constructs may differ if provided by other people from these participants’ social circles, or by other measurement techniques (not self-reported). Furthermore, evaluations may be temporarily unstable, i.e., being acute only at the time of the interviews, and the unreliability of memory whilst being interviewed means that participants’ responses might have represented only a snapshot of their health-related opinions, adjusted for the interview situation. Additionally, the interviews were coded by only one coder–me–despite discussions with colleagues that supported my interpretations. Finally, the sample was too small to fully elucidate gender and place of residence differences. For this reason, research on larger samples is needed.

### 4.2 Practice, policy, research implications

The findings of this article could be used by practitioners to develop interventions aimed at improving youth in Russia’s self-rated health, self-care behaviors, and therapeutic network components after COVID-19. In particular, practitioners should keep in mind that if they attempt to modify one of these social constructs, the other may also be affected. Additionally, without changing all of the constructs at once, one construct may only be marginally altered. Planning complex interventions that impact all three of the primary factors under investigation in this study at once is therefore necessary; concentrating on individual variables could result in insufficient and unsuccessful interventions. Thus, it is crucial that policy actors fund these kinds of intricate projects rather than cutting costs and concentrating solely on one or two factors. Researchers should focus heavily on developing a deeper grasp of the causal relationships between these ideas and conducting additional analysis in the framework of intersectional analysis in the future.

## 5 Conclusion

This is one of the few investigations that looked at young individuals without chronic medical diagnoses who concurrently assessed their own health, self-care routines, and therapeutic networks. The main findings show that young people with low health evaluations were more likely to have mothers and other medical professionals in their therapeutic networks and to practice self-care with the goal of improving their health. In addition, those who reported their health wasn’t good played more sports and took care of themselves even while they were ill. Regarding the kind of health evaluation, there was no relationship found between the goals of self-care practices, participants in the therapeutic network, and self-care activities. Overall, it is plausible to imply that therapeutic network practices and structure are connected to self-evaluated health, but quantitative research is required to confirm the causal relationship.

## Data availability statement

The raw data supporting the conclusions of this article will be made available by the authors, without undue reservation.

## Ethics statement

The studies involving humans were approved by HSE University Ethics Committee. The studies were conducted in accordance with the local legislation and institutional requirements. The ethics committee/institutional review board waived the requirement of written informed consent for participation from the participants or the participants’ legal guardians/next of kin because it is not requested in our country for children older 14.

## Author contributions

OM contributed to the whole study design and data analysis.
