# Supplementary material for: Russian young people’s subjective health evaluations, self-care practices, and therapeutic networks
Source: Front Psychol. 2024 Feb 23;15:1247445. doi: 10.3389/fpsyg.2024.1247445 (PMC10995920; doi:10.3389/fpsyg.2024.1247445)
Supplement: Supplementary file 1 [file Data_Sheet_1.PDF]

Supplementary Material

Russian young people`s subjective health evaluations, self-care practices, and therapeutic networks after COVID-19 pandemic

Oxana Mikhaylova[12]

[1]Center for Contemporary Childhood Research, HSE University, Myasnitskaya Str., 20, Moscow, 101000, Russian Federation

[2]Department for Social Institutions Analysis, HSE University, Myasnitskaya Str., 20, Moscow, 101000, Russian Federation

*** Correspondence:**

Oxana Mikhaylova

oxanamikhailova@gmail.com

1 Tables and figures

1.1 Table 1. Sample characteristics

Participant number	Age	Gender	Place of residence
1	19	Male	Moscow
2	19	Male	Rostov on Don
3	19	Male	Rostov on Don
4	25	Female	Tula
5	21	Female	Tula
6	19	Male	Moscow
7	18	Female	Izhevsk

Participant number	Age	Gender	Place of residence
8	24	Female	Sochi
9	23	Male	Moscow
10	23	Male	Moscow
11	19	Female	Kursk
12	24	Male	Moscow
13	24	Male	Moscow
14	18	Female	Rostov on Don
15	19	Female	Rostov on Don
16	19	Female	Rostov on Don
17	22	Female	Moscow
18	17	Female	Sarapul
19	20	Female	Moscow
20	22	Female	Moscow
21	24	Male	Moscow
22	22	Female	Rostov on Don
23	25	Female	Moscow
24	23	Female	Moscow
25	24	Female	Moscow
26	25	Male	Ufa
27	24	Male	Tula
28	16	Male	Istra
29	23	Male	Bali
30	18	Male	Novorossiysk
31	18	Female	Moscow
32	20	Male	Rostov on Don
33	21	Male	Rostov on Don

Participant number	Age	Gender	Place of residence
34	21	Male	Rostov on Don
35	19	Female	Moscow
36	21	Male	Rostov on Don
37	18	Male	Ekaterinburg
38	16	Female	Moscow
39	19	Male	Ryazan
40	23	Female	Moscow
41	22	Female	Omsk

Note: All of the tables that follow were created using MAXQDA. To perform similar calculations on code intersections, refer to the following information in the manual: https://www.maxqda.com/help-mx24/visual-tools/code-matrix-browser-visualizing-codes-per-document

In essence, this app collects data on code frequencies and their intersections with one another among various interviewees. Features of the interviewees, like gender or residence location, can be utilized to compare code frequency distributions amongst different interviewee groups.

I created Word documents containing each interview and transcribed the interviews in order to build the tables and image-maps prior to utilizing the software. 41 files in total. These files were then imported into MAXQDA. I added the variables for age, gender, and place of residence—which are listed in table 1 of this appendix—to each MAQDA file. In the end, I looked through every transcript in the program and gave the relevant parts of each of the three categories of codes: 1) self-evaluated health (bad or good; mental health or physical health), 2) self-care practices (types of practices; goals), and 3) therapeutic networks (members; their functions). I also provided subcode details for them. Table 2 presents the coding findings. The intersections of codes with research variables (gender and location of residence) are the main focus of Tables 3–13.

1.2 Table 2. Code system and frequencies

Codes	Frequency
Total	1823
(1) Subjective health evaluation	293
1.1. Type	

Codes	Frequency
Mental health	82
Physical health	72
1.1. Valence	
Bad	81
Great	51
(2) Self-care practices	306
2.1. Goals	156
help	103
maintenance	50
improvement	38
self-analysis	18
decision making	7
2.2. Types	150
doing sport	49
going to the doctor	29
becoming a health professional	16
eating well	14
making tea and other care activities, when ill	14
being kind to self and others and volunteering	13
smoking	12
drinking	11
working too much	10
self-talking	8
doing regular checkups	4
reading	4

Codes	Frequency
talking with significant others	4
recreational activities avoidance that are harmful for health	4
making art	2
playing computer games	2
self-harming	2
doing esoterics	3
taking drugs	1
self-analysis marking symptoms of illnesses	1
resting	1
doing hobbies	1
caring for pets	1
(3) Therapheutic networks	130
3.1. Health professionals	83
cures	83
3.2. Colleagues	5
invigorate to self-care	2
deteriorate mental health	2
neglect	1
make the company in self-care practices	1
3.3. Mothers	42
give advice	15
parental self-care health practices influence child health	12
reacts to self-care practices	7
control	5

Codes	Frequency
neglect	4
facilitate	3
monitors, reads the child feelings	2
teaches how to take care	2
makes instead of child	2
conflict	2
separation	1
3.4. Fathers	23
neglect	6
facilitate	5
parental self-care health practices influence child health	4
separation	3
control	3
give advice	3
conflict	2
makes instead of child	2
monitors, reads the child feelings	1
3.5. Friends	14
make the company in self-care practice	3
discussion partner	3
the source of doubt, stress	2
helps these friends with mental health problems	2
neglect	1
death	1

Codes	Frequency
give advice	1
compares with friends	1
3.6. Teachers	13
facilitate	7
give advice	2
make the company in self-care practice	2
neglect	1
criticize	1
discussion partner	1
control	1
3.7. Romantic partners	10
invigorate to self-care	5
deteriorate mental health	4
give advice	1
control	1
discussion partner	1
3.8. Media	9
give advice	6
invigorate to self-care	3
influence close people make them change their behavior	2
3.9. Peers	8
make the company in self-care practice	5
invigorate to self-care	4
compares with peers	2
reacts to self-care practices	2

Codes	Frequency
criticize	1
3.10. Grandparents	5
neglect	1
make the company in self-care practice	1
reacts to self-care practices	1
give advice	1
facilitate	1
3.11. Strangers	3
helped	1
helped the stranger	1
neglect	1
3.12. Siblings	2
influenced parental attitude to the person	2

1.3 Table. 3. Health type in context of participant gender and place of residence (count per document)

	Gender		Place of residence		Total
Health type	Males	Females	Region	Moscow	
Mental health	12	18	18	12	30
Physical health	14	16	19	11	30
SUM	28	34	37	36	96
N = Documents/Speakers	20	21	24	17	41

1.4 Table. 4. Health valence in context of participant gender and place of residence (count per document)

	Gender		Place of residence		Total
Valence	Males	Females	Region	Moscow	
Bad	13	19	18	14	32
Great	11	9	13	7	20
SUM	24	28	31	21	52
N = Documents/Speakers	20	21	24	17	41

1.5 Table. 5. Self-care practices types in context of participant gender and place of residence (count per document)

	Gender		Place of residence		Total
Self-care practices types	Males	Females	Region	Moscow	
doing sport	15	13	11	17	28
doctor choice	8	10	9	9	18
becoming a help professional	1	7	4	4	8
eating well	5	4	3	6	9
making tea and other care activities, when ill	5	5	3	7	10
being kind to self and others and volunteering	4	5	7	2	9
smoking	6	3	2	7	9
drinking	7	2	3	6	9
working too much	2	7	1	8	9
self-talk	4	2	2	4	6
doing regular checkups	2	2	1	3	4
reading	3	1	2	2	4
talking with significant others	1	3	2	2	4
recreational activities	2	2	3	1	4

	Gender		Place of residence		Total
Self-care practices types	Males	Females	Region	Moscow	
avoidance that are harmful for health					
making art	1	1	1	1	2
computer games	1	0	1	0	1
self-harm	0	1	0	1	1
esoterics	0	1	0	1	1
taking drugs	1	0	0	1	1
self-analysis marking symptoms	1	0	0	1	1
resting	0	1	0	1	1
doing hobbies	1	0	0	1	1
pets caring	0	1	1	0	1
SUM	70	71	56	85	141
N = Documents/Speakers	20	21	17	24	41

1.6 Table. 6. Self-care practices goals in context of participant gender and place of residence (count per document)

	Gender		Place of residence		Total
Self-care practice goals	Males	Females	Region	Moscow	
help	14	18	18	15	32
maintenance	14	12	16	10	26
improvement and disciplinary	9	10	15	6	19
self-analysis	8	7	10	5	15
decision making	2	3	4	1	5

	Gender		Place of residence		Total
Self-care practice goals	Males	Females	Region	Moscow	
SUM	47	50	63	37	133
N = Documents/Speakers	20	21	24	17	41

1.7 Table. 7. Therapeutic network members in context of participant gender and place of residence (count per document)

	Gender		Place of residence		Total
Therapeutic network member	Males	Females	Region	Moscow	
health professionals	11	17	16	12	28
colleagues	1	4	3	2	5
mothers	10	15	14	11	25
fathers	5	10	8	7	15
friends	6	5	8	3	11
teachers	4	4	7	1	8
romantic partners	2	3	3	2	5
media	2	6	5	3	8
peers	4	2	3	3	6
grandparents	0	4	2	2	4
strangers	0	3	1	2	3
siblings	0	1	0	1	1
SUM	45	74	70	49	154
N = Documents/Spea	20	21	24	17	41

	Gender		Place of residence		Total
Therapeutic network member	Males	Females	Region	Moscow	
kers					

1.8 Table. 8. Intersections between codes on therapeutic networks and health type (count per document)

Therapeutic network member	Mental health	Physical health
health professionals	24	22
colleagues	4	5
mothers	23	20
fathers	14	11
friends	9	8
teachers	7	7
romantic partners	5	3
media	8	6
peers	6	6
grandparents	4	4
strangers	2	3
siblings	1	1
SUM	327	303

1.9 Table. 9. Intersections between codes on therapeutic networks and health valence (count per document)

Therapeutic network member	Bad	Great
health professionals	26	15
colleagues	4	4

Therapeutic network member	Bad	Great
mothers	21	14
fathers	14	10
friends	8	8
teachers	6	7
romantic partners	5	3
media	6	4
peers	5	5
grandparents	4	1
strangers	3	0
siblings	1	0
SUM	307	214

1.10 Table.10. Intersections between codes on self-care practices goals and health type (count per document)

Self-care practice goals	Mental health	Physical health
help	26	27
maintenance	23	23
improvement and disciplinary	17	16
self-analysis	14	11
decision making	5	4
SUM	169	165

1.11 Table.11. Intersections between codes on self-care practices goals and health valence (count per document)

Self-care practice goals	Bad	Great
help	31	18
maintenance	23	17

Self-care practice goals	Bad	Great
improvement and disciplinary	16	15
self-analysis	14	11
decision making	5	4
SUM	170	122

1.12 Table.12. Intersections between codes on self-care practices types and health valence (count per document)

Self-care practices types	Bad	Great
doing sport	24	16
going to the doctor	18	12
becoming a health professional	8	5
eating well	7	7
making tea and other care activities, when ill	10	5
being kind to self and others and volunteering	9	6
smoking	7	5
drinking	7	6
working too much	7	6
self-talking	6	5
doing regular checkups	3	4
reading	3	3
talking with significant others	3	3
recreational activities avoidance that are harmful for health	3	2
making art	2	1
playing computer games	1	1
self-harming	1	0
doing esoterics	1	0

Self-care practices types	Bad	Great
taking drugs	1	1
self-analysis marking symptoms of illnesses	1	1
resting	1	1
doing hobbies	0	1
caring for pets	1	0

1.13 Table. 13. Intersections between codes on self-care practices types and health type (count per document)

Self-care practices types	Mental health	Physical health
doing sport	21	26
going to the doctor	16	15
becoming a health professional	8	6
eating well	7	9
making tea and other care activities, when ill	8	9
being kind to self and others and volunteering	8	7
smoking	7	9
drinking	7	9
working too much	8	8
self-talking	6	5
doing regular checkups	4	3
reading	3	4
talking with significant others	4	3
recreational activities avoidance that are harmful for health	2	4
making art	2	1
playing computer games	1	1
self-harming	1	1

Self-care practices types	Mental health	Physical health
doing esoterics	1	1
taking drugs	1	1
self-analysis marking symptoms of illnesses	1	1
resting	1	1
doing hobbies	1	1
caring for pets	1	1

1.14 Fig. 1. Therapeutic networks.

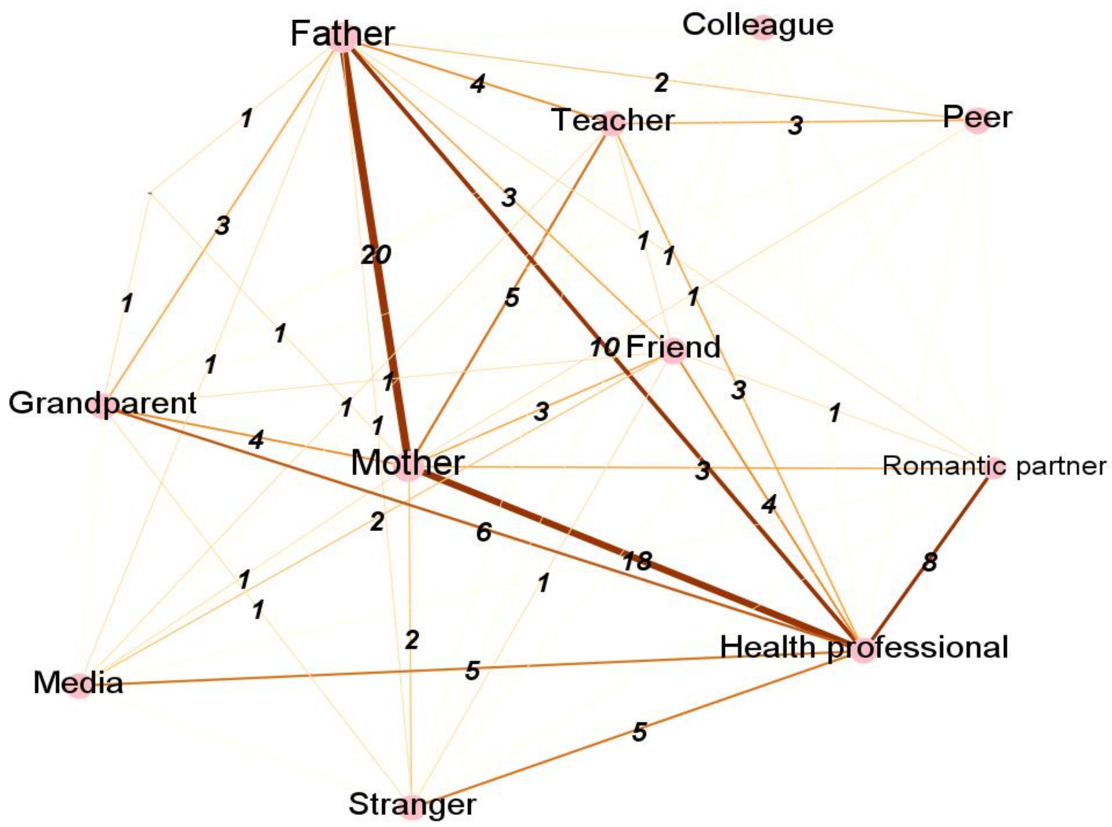

Note: Nodes are individuals who engage in young person self-care activities, while ties are references to self-care behaviors made within the same story. The MAXQDA software was used to create the figure. Please refer to this free program manual, https://www.maxqda.com/help-mx24/visual-tools/code-relations-browser-visualizing-overlapping-codes , for further details on creating networks out of code interrelations.

1.15 Fig 2. Therapeutic network members and their role in self-care practices

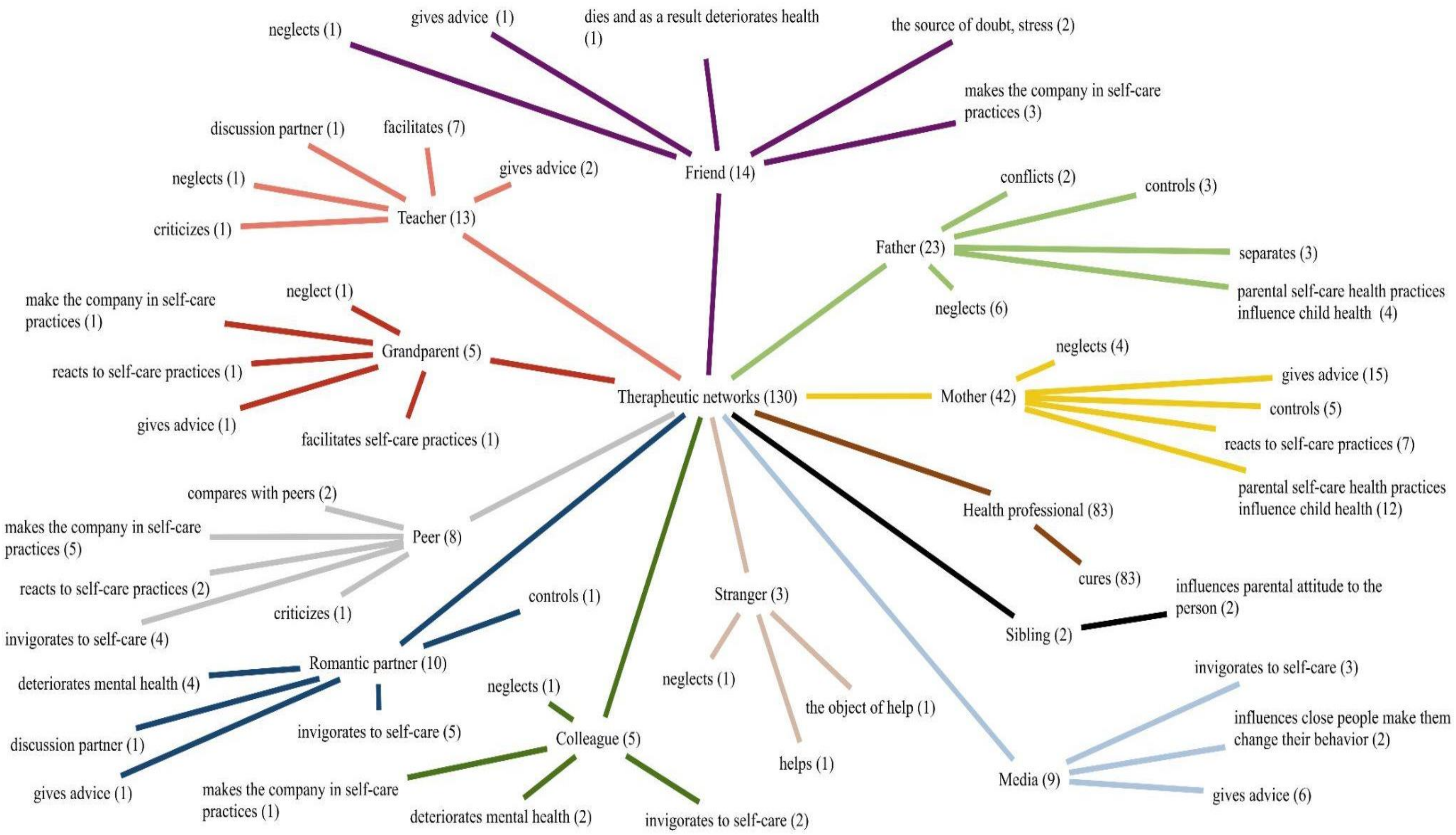

Note: This network of general codes and subcodes illustrates precisely what participants in therapeutic networks do to support an individual's self-care. In total, specific individuals and their roles were referenced 130 times across all interviews. People are included in the general codes. Their roles are subcodes. The overall code and any associated subcodes are colored the same to aid with clarity. The frequency with which each code and subcode appears in the narratives is displayed next to it. Please refer to the free program manual at https://www.maxqda.com/help-mx24/visual-tools/code-relations-browser-visualizing-overlapping-codes for more information on how this map was created using MAXQDA.

2 Interview Guide (translated from Russian by the researcher)

Hello! Thank you for agreeing to take part in the study. Today we will talk to you about autonomy and life choices. Our conversation will be recorded on a voice recorder, but we will maintain the confidentiality of the data. The interviews will be analyzed in aggregate form. You can refuse to continue our conversation at any time. Please answer the questions in detail. If you agree, then let's start?
To begin with, please tell me how best to contact you, how old are you, what city do you live in now, where do you study? Do you have any hobbies/hobbies? Whether you work?

2.1 Part 1. Biographical history

Now please tell me about your life from the moment of birth to the current one. Try to dwell in detail on all the ages at which you remember yourself, events and life choices that you consider important. The story should be long, about 10–15 minutes, then I will ask several clarifying questions.

2.2 Part 2. Life choices

Thank you for your detailed story, probably more than once in your life you have had and have to make some kind of choice, and now we will talk in more detail about such situations.

2.2.1 Spheres of choices

Let me divide your life into spheres.

Please tell us about each of these areas, what choices have you had to make in each of them over the past year?

- Training, professional development

- Health
- Job
- Relationships with parents
- Friendly relations
- Location
- Lifestyle/leisure
- Romantic relationship
- Finance

Please rate the areas of elections in terms of the difficulty of making a choice for you now, where 1 is “very easy to make a choice”, 10 – “very difficult to make a choice” and explain why.

2.2.2 Unsuccessful choices

Are there any choices in your life that you consider unsuccessful? Why do you consider them such?

Tell me about any experience in your life that you view as a BAD choice that has had a significant impact on your life now.

When narrating, focus on the following points:

-what happened (the essence of the situation);

-when did it happen (time);

-who were among the participants in this situation (participants);

- where it happened (place);

-what feelings did this situation cause then (feelings then);

- how does this situation make you feel now (feelings now);

- what thoughts does this situation cause in you now (thoughts then);

-what do you think about the situation now (thoughts now).

2.2.3 Successful choices

Which choices do you consider successful? Why do you consider them such?

Please think about any incident in your life that you consider as a GOOD choice that significantly influenced your life now.

When telling your story, focus on the following points:

-what happened (the essence of the situation);

-when did it happen (time);

-who were among the participants in this situation (participants);

- where it happened (place);

-what feelings did this situation cause then (feelings then);

- how does this situation make you feel now (feelings now);

- what thoughts does this situation cause in you now (thoughts then);

-what do you think about the situation now (thoughts now).

2.2.4 Future choices

Probably you will have to make a number of choices in the future. What kind of choices do you think you will face with in the next 10 years?

3 Code queries with citations from the interviews

All citations are translated from Russian language automatically using DeepL https://www.deepl.com/ru/translator. Each row presents citations from the interviews.

3.1 Table 1. Subjective health evaluations (bad)

Citations
Yes, it turns out that after that I trained for three more years, I also went to different competitions, in different cities, I already understood, well, since I refused that chance, that chance, a second one most likely won't come to me, but I've already trained, let's say So, semi-professionally, I played for our academy, CSKA, but I (.) didn't dream of going there to some Wimbledon and so on. And one day, when I went to a competition in Taganrog, I was fighting for first place, the last one turned out, the last game was for first place, and I fell on my back, so. I injured my back, then I couldn't walk at all for a week, I was thinking that it was a fracture, or that it was a bruise. They took a picture and said that it was just a spinal bruise, well, it would go away soon, but after that I already quit tennis, because even after rehabilitation there, about six months, when I returned to tennis, the indicators were completely different and, accordingly, , guys who were plus or minus my age, they continued to increase the pace while I was rehabilitating there and so on. Well, basically, I was, roughly speaking, no longer needed, even in this, at this level of tennis, and I decided to leave.
Yeah, health. Until I was 17 years old, well, I was an athlete, and so on, and so for a very long time, well, until I was 17 years old, I always told myself that there is no smoking, no drinking, I will never be there, it's not for me, especially for me Dad smokes there, I think no. But then, at the age of 17, after (.) the death of my friend, my second, I started smoking. I still smoke to this day, I reacted at first... I still have thoughts, why did I do it? Maybe it's worth quitting? But then I think that, well, for now this, for example, brings me pleasure and some kind of outlet, in its own way, so I think for now I'll make a choice in this direction, and while it develops in this way, well, I'll drink there, I don't know, there's a glass of wine on holidays, roughly speaking. That is, not a fan. The choice to start smoking was conscious; I purposefully wanted to try smoking and start smoking. That is, I don't know how to describe it, one day I think - I want and will smoke, and that's it. And I kind of tried it, and that's what I wanted. M. It worked. M. Yes. (laughter) That's how it all happened. Therefore, the choice was also probably easy, I probably don't really regret this choice, well, although sometimes, perhaps some thoughts slip through there again, but in terms of complexity this choice was, I also think there were about three or four, probably maybe, something like that.

M. Yes, I think I'll quit, to be honest, now I'm already trying cigarettes, I don't smoke, I've switched to alternative, let's say, methods of obtaining nicotine, like IQOS, well, for example, something like that, it's still unknown of course, which is less harmful, but at least warms the soul, which they say may not be so harmful. And I think yes, and I'm gradually starting to come to the point of quitting smoking, completely, because I'm starting to realize that very often I smoke there, when, for example, I'm either bored or need to occupy myself with something there, any stressful situations, and so on. And when this is not such a strong need, then it is quite possible that this can be easily abandoned. And therefore, I think soon (.) I will quit. Here. Anything else to say on this topic? Or (.) can we move on?

As a result, I smoked from the second to the fourth grade, that is, I had been smoking for almost 2 years, being generally a schoolboy who had his head on the table (npt) himself because he was running. But it turned out that I had to quit for two reasons. First, and most importantly, health problems began. I'm sitting there, this is all for me, I've played enough, I won't.

And in the end, the actions reached the point where the teachers all scolded me for a very long time, and were about to label me as, well, like, not a street child, but whatever it is, well, in short, register as a child. Here. But there it turned out to be somehow simple, I was simply put on the internal school register. And they said that if something happens again, then we will have the same horror. A little time passed, a week later the fight started again and then they started beating me, probably about seven people beat me, on the street, it was in winter. But at some point, I kind of just lay there angry, I don't know, something confused me, I abruptly shoved everyone who was standing, fought off with steps, quickly jumped up, kicked someone else there and caught up with one, and just I took the snow that was lying, and then there was some kind of ice and I decided, I thought that I would just wash his face with snow, it so happened that I took a piece of ice and it accidentally hit him in the temple. Nothing terrible happened there, the ice just cut the skin a little, that is, not even a cut, it was so strong. But in the end, I was still scared too, that's it, I won't go to school anymore. Let's go to some doctor and say that blah blah blah, let's check the child, I'm there, I don't remember exactly what happened. Well, in short, the doctors there determined that there was some kind of prolonged depression, they prescribed the child, well, fifth grade, antidepressants, and they forced him to sit on antidepressants. I spent a long time on them, on the course, I don't really know, me, well, not systematically, but at times, my mother seemed to say to them - let's continue to drink this. And these were, I really don't remember, either some very mild tranquilizers, or antidepressants, I remember... They were probably called something, I've already forgotten. But the point was that my studies failed because of this, I just stood at the blackboard like a vegetable. I didn't have a single thought in my head and the teachers often looked sideways at me, and to themselves, that is, like this drug addict, to the narcologist... What's going on there. Carefully. I'm sorry, the girl dropped something. Here. We went to a narcologist, where he told me that, buddy, everything is fine, they showed him a prescription for these antidepressants, and for a long time they refused. But somewhere around the eighth grade, I realized that there was a huge difference in interaction between me and my classmates, that is, more and more of the time that I sat at my desk, I simply watched how my classmates' adolescence was passing. And at some point, like in some "Rocky," I stood up in my room and said - I won't drink, that's it. I refused. And somewhere from the eighth to the ninth and eleventh grades, I began a time of real rehabilitation, when I, well, there my first interactions began, just like those who were more mature. Here. There's a story with friends, one there, others, the fourth, it still didn't work out with the girls, that's it. But somewhere around the tenth or eleventh grade, I was already completely rehabilitated, both as if just on my own, and in the eyes of my peers, who saw me both in the sixth and seventh grade. And we kind of fell into the same sort of path, that is, the same groups, and on our own we seemed to communicate normally. And this, this dynasty that I carried with me from the bad events that were

covered by my pills, somehow left. Then it so happened that even by my first year it all continued; I began to experience a simply stormy, simply huge, stormy life as a freshman. And I slept, it was so that I slept at night every other night, one night. That is, I worked in the morning, afternoon and evening on projects at the university, at night we went to parties, the next day I still worked in the morning, afternoon and evening, and at night I slept. There were nights when I didn't sleep, not because there were parties, but because I didn't have time to complete projects by the deadline, and I also had to work at night, because, of course, there were some situations, they just calculated it wrong , one of the teammates did not fulfill his duties there, and so on. Here. And I, as the leader of the project, just needed it to be completed. No matter what a person does, I will try for him now. Here. And my classmates, whom I later met, joked that I was speedrunning my life. Speedrun, this is true, just in case, for those who will decipher this later, this means accelerated, accelerated passage. That is, among the players there are those who compete to see who can complete the game faster. This is called speedrun. I was told that I was in the speedrun of my life. That is, in terms of (..) everything that they felt there during the entire time when they studied there from the fifth to the eleventh grade, I felt literally in 3 years, and even further, what they had not yet had time to experience, I already felt it. That is, I was carried away somewhere there.
M. Ilya, very detailed, thank you. In general, I'll start with your first phrase, when you started speaking, you said that reflection is always welcome. And as if from your story, it also came up a couple of times that you have, as it were, experience communicating with different psychologists. But tell me, tell me, are you, in general, inclined to reflect on your own, or how did this experience influence you, why are you talking about yourself in such detail, analytically? I. It's like two in one. Like these non-cafés. But it turned out like, the story itself from the 8th grade there, when I came off these antidepressants, well, it turned out that in order to understand this, it was necessary, of course, to reflect there. I have minimal communication with a psychologist, well, except for this psychologist I just talked about, 27 years old. Because every time I went to a psychologist, it ended, well, there were one, two, three or four trips. And they told me what I already know. And most of the psychologists that I just went to said, "I don't need to go to a psychologist, I calmly delve into my own head." That is, I will scatter if I can. The only times when I actually went to a psychologist with benefit was when I went for IQ tests. This was when I went, I sat there for an hour with him, he solved the test, then yes, this was my only useful trip. And the rest, if they didn't just interfere, somehow changed life for the worse. Although it is also unclear which one, good or bad. Probably a good one after all. Then the rest were useless. Well, it probably turned out that I myself am a reflective person, I even think at the Skolkovo university the moderators told me that at times I was even hyper-reflective, let me look for meaning where there is simply none. (..) But it so happened that the element in which we learn, it implies reflection after the end of each activity, project. Necessarily. That is, we have to sort everything out, what, why, but they didn't teach it in a very good way. But when I practiced with the Skolkovo team in game technology, a prerequisite there was, as it were, reflection itself, game technology, and in detail, that is, I had to write a plan, with the tools that I used, that is, I said what I want to try on the guys there such and such, such and such a method, there is interaction between them, maybe there (npt) according to Weldon a couple of people there can move from an idea generator to an executor, and the like. And then come back and write a reflection. It didn't work out because like this, like this, like this. You need to try this, maybe something like this. So much so that I needed to read the reflections of those guys whom I sort of moderate myself. Therefore, it's like I'm reflective myself, and it so happened that I also needed to practice this intensively.
M. But I'll immediately clarify this episode, when you already felt that you, well, smoking began to affect your health, and now your parents burned you, this moment, in general, you wanted your

parents to burn you in these activities, or did you not care at all? I. On the contrary, I wanted them not to burn me, (laughter) to receive this strongly, and then for them to know, in principle. Yes, it's just that my father, in my earlier time, probably around the sixth grade, had big problems with alcohol, very big ones. (..) And there it got to the point where I was just sitting, he told me - look, cigarettes, smoke the whole pack there, so that you can get more, so that you feel that this is not the same, and don't smoke there anymore. Well, the like. Well, basically, since I was in school, my father probably doesn't drink anymore. That is, I don't know how many years have passed there, maybe even 7 years for sure. (..) Yes, in general, it's been more than that, probably almost 10 years or something, since my father doesn't even drink anything strong. Well, similar. He didn't even seem to imply that smoking a pack of cigarettes would just be like, well, a pleasure. Here. But no, I didn't want my parents to burn me at all, it happened by accident, a classmate burned me, that is, she complained. Oh, I forgot to specify, so that my parents wouldn't burn me, I sprayed cologne into my mouth. It was an old children's cologne, I don't remember, it was unlikely that it was triple aged, because it was a regular children's cologne. But it was definitely alcohol-based, and I even remember it was "Pirates of the Caribbean", then these colognes were also coming out. And I just poured cologne into my mouth. Here. It was terrible, but what can you do, you had to make some sacrifices.
About health, with health... (..) The story is that, let's say, as soon as I received normal money, I finally went to have my teeth treated. I had a big problem with my teeth and I passed, then I took a genetic test to check for some congenital diseases and discovered several congenital diseases. Here I would not say that I was unlucky, on the contrary, it was good, I saw there and saw some predispositions in terms of hormonal ones too. I went to the endocrinologist and there they also developed, drank some zinc and so on. Here. Well, plus I had a dental operation that didn't go very well, they pulled out a tooth, it turned out to be the sixth, chewable one, and he had a cyst on the root, and the cyst ate a layer into the sinus. And now between, well, where the tooth was, I have a hole in my sinus. That is, such a good message, air can go there, back and forth from the sinus to the mouth, water can go into it, so. And this, in principle, can only be healed with an implant. This, well, with health, it means that it was necessary, well, it was necessary, well, most activities were prohibited. But now, for example, it's literally been a week here, and after work I'm going to go, well, recover in the gym. Because now I couldn't go to the gym for a while.
M. And now there are those moments on which you consult with your parents? I. (sigh) (h) (...) Well, probably for health reasons, I always consult with my mother, because she is a nurse. Here. That is, if I suddenly start to get sick there somehow, in a way that I have never treated myself before, that is, if I start some kind of ARVI or something there, I can basically cope with it myself. Here. And when it comes to some symptoms that I haven't had before, I call my mother and talk, if they don't help, then I go straight to the doctor. Here. (..)
M. Well, great. That is, in addition to your profession, you also have a hobby that gives you pleasure. And you seem to have mentioned something else in your hobbies, music, right? M. Yes. Yes. Here. Well, it's more... There is some kind of story in me that I want to learn how to play well, and so on, but I'm probably too lazy for this, and even more so, it seems to me that I don't have good potential for this . That is, I can play some banal things there, play something on chords, sort of a little strumming, that is, I can play, and... Well, it's not like I studied there, that is, it's more by instinct it sort of went, I liked it there, how I typed some kind of melody, I started to continue there. Here I am... M. How old were you when you started playing music? M. (sigh) (..) Oh, probably 18-19 somewhere. In this regard. M. And it was also your choice, right? Or... M. Yes, yes, it was my choice, completely. It's just that at one point I realized that... Well, again, I'm probably a depressive psychotype, I don't remember, if that's what it's called, so. And I bought myself a musical instrument simply because sometimes I'm in such a state that I just want to sit in

the dark and strum something sad to myself. This somehow makes it easier. M. But it's cool that you have such a resource that you know what to do in such conditions. M. Well, before, I used to, as they say, beat myself up with sad music, but still doing this, as they say, this is probably my favorite self-flagellation and so on. Well, it's probably not self-flagellation, it's probably just some kind of self-harming. Here. First I bought a ukulele, realized that it didn't sound sad enough, and bought a guitar. (laughter) Here. There's more bass, more sadness can be created, let's say. Here. Well, sort of in this format. M. Yes, it's interesting, that is, you can take this sadness out from the outside and create it yourself. That's great too. M. Yes. (laughter) M. No, it's really great that sadness comes out of you and is expressed in creativity. This is great. M. Well, including... Oh, yes, by the way, I remembered another hobby, you said, expressing yourself in creativity, also not so long ago, a year ago, I think, I started writing a poem, here. Also, as they say, sadness had a very strong influence on this, along with depression, so. Because there is not a single funny poem that (laughter) I wrote. Because, it seems to me that the strongest emotion is the worst emotion. And based on this, here comes, well, let's say, what I do.
Health. Health is the area that suffers greatly in my life, I'm sick right now, right now. And I recently took a Covid test there, yesterday, and I'm waiting to see whether it will happen or not, but it seems that it won't, I already feel fine. But, damn it, I haven't been doing much about my health lately, but I just support it with sports and that's it. But I would like to undergo some kind of examination, the fact is that the right side of my head has been hurting all my life, and it's such a noise, it's just as if, I can describe this pain as a noise. I've already gotten used to it, because I know that damn it, this never happens, once in the tenth grade I did an examination that did not give any intelligible results, they told me that I was healthy, I'm into it I don't really believe it. (laughter) Here. Still, something is wrong. Otherwise, health...
A. Oh, you know, this is very interesting, I had no thoughts. Well, that is, if we are talking about some kind of rational reflection, and I mean perspective reflection, that is, when we assess risks, when we, we don't know, analyze a situation that could happen, it in general (xxxx) (laughter) there was none. Well, that is, it's purely based on some kind of emotion, some kind of euphoria, it's probably possible, I didn't use psychoactive substances very much, I smoked marijuana twice, but it seems to me that this is something similar to what people experience under psychoactive substances, such an absolute frenzy and absolute lack of self-control. Here.
Next, health. (..) Yeah, health, health. Here, you know, there is a rather difficult aspect, because for probably several years my health has fluctuated, (laughter) let's say, like the exchange rate of the ruble on the stock exchange. Probably until 9-10 grade, I was quite (.) a chubby boy. And I probably made the choice in favor of a healthy lifestyle on the basis of some, let's say, personal oppression and personal motivations. But for the most part this was due to teenage conflicts. And, accordingly... (..) Any problems. M. Did someone offend you? I didn't quite understand about oppression. A. Let's just say, (..) at some point it began to seem to me that at school, on the part of friends, on the part of teachers, at some sharp moment the attitude towards me changed. That is, they stopped noticing me and (.) paying any attention to me. Although I didn't seem to do anything wrong. Nevertheless, I didn't really understand what happened, but at that moment it was probably quite difficult for me to communicate with people, because I didn't understand what this attitude was connected with. That is, if I had a teacher with whom I was in close contact, and (.) with whom, in principle, I was conducting some kind of project activity, then at that moment it happened, I don't know how some day came and as if at the snap of a finger What happened was that this person's entire attention switched to another audience. And essentially, you know, it's like a child who has been sharply deprived of attention. You want it, but they don't give it to you. And I probably began at that moment to look for disadvantages in myself and in my appearance, in my

health. I began to think that this was probably connected with some characteristics of my body or characteristics of my thoughts. And then I probably started soul-searching. (.) Therefore, the choice in favor of health, it was probably given to me, let’s say, through a battle, due to some experiences, through an attempt to prove to myself and many others that everything could be different, that I can do something... something else. Therefore, in terms of severity, probably (..) well, let it be a ten. At work... M. So you connected this deterioration in your relationship with the fact that you somehow gained weight, or somehow didn’t look the way it seemed right, right? Because you were talking about the love for sweets, and about being chubby, you said? Or did I misunderstand you? A. Well, for the most part, I’ve been pretty chubby since childhood, and I probably stayed that way until the 9th grade. Therefore, purely physically, I didn’t change much, due to weight gain or loss. There was simply, let’s say, one consistency of the body, which was maintained as it grew. Therefore, I don’t know, I probably connected this not with an increase or change in weight, but with the fact that at some point some skills and shortcomings that people did not want to take into account simply became more noticeable. M. Did you say something to you, did you somehow become yourself, did you say about choosing a healthy lifestyle? Something like this. A. Yes, I decided that... The Unified State Exam had just ended... the Unified State Exam, in the 9th grade, I thought that since such a situation was happening, I needed to rethink myself, I needed to somehow start taking care of myself, to show myself, well, to change something in myself, not only mentally, but also physically, so I probably made the choice in favor of a healthy lifestyle, in terms of athletics. That is, I got up at about 5 in the morning, slammed the doors, everyone was scared of what was happening so early in the morning, and I just left the house and started running at the stadium, ran to some park and spent my time there.
M. I see. How do you help your friends? Well, in fact, were there any similar interesting situations? A. I don’t remember them so often, but most often these are situations related to a psychological and moral point of view, because they fall into some kind of depression, something bad happened to them, I try to support them, I remember sitting at night , talked to them, tried to support them, although he himself took the test in the morning. It was so. Here.
M. And if we talk about health, then when you make decisions in this area, what helps you do this, for example, a consultation with a doctor or how do you even choose a dentist? T. Well, it turns out that in this regard I’m probably suspicious about my health, that is, if some kind of sore pops up, I start there, well, not exactly panicking, but it’s faster, faster to take some action to remove this pain. Just with teeth, and everyone knows that this is a rather expensive procedure, namely dental treatment. And therefore, finances did not quite allow, or rather allowed, but there were other, well, one might say, demanding, that is, aspects that required an urgent investment of money. And that’s why I put off just about the teeth. Here. And therefore, in principle, since I have already said that I am suspicious, I quickly, quickly tried to fix all this as quickly as possible, so as not to start all this. Then don’t pour in even more finances, already eliminating the consequences of all this. M. But I’m even asking more about how you understand who is a good dentist there and who is bad, about this. So, where do you get this? T. Well, here again, already through acquaintances, that is, I found out from people who treated there, and about prices, and so on. There was no such thing here that, yeah, I typed dentists there into Google, and went to the first link that came up, and immediately went there. That is, naturally, at first there was some (..) research, so to speak, on this matter, that is, I found out from friends there who was doing good work and, accordingly, I went there.
I. So health... What elections, elections, elections, elections, elections, well, it seems to me that with my health pah-pah-pah, everything is fine, I try not to get sick, (laughter) here. It seems to me, well, if we take health in general, well, my mother just had a stroke there. I had to make certain choices there, I don’t know, about apartments, about inheritance, and so on and so forth. This all also falls on me, in any case, so for me, well, it was hard for me, it’s still hard for me to resolve all

these issues, because in many areas, with many of these things, I'm meeting for the first time, there the insurance company, the hospital, I don't know, they have to process certain documents, it's incredibly difficult for me. As a child, my health was bad. As a child, after childhood bronchitis, I had very severe bronchial asthma, I was sick for a very long time. I rarely, I didn't go to kindergarten at all, and I went to school, but I was sick very often, of course. (..) It was very difficult for me as a child, so it seems to me that I would rate my health as exactly this, I would rate it as an eight
when everything seems to be bad, but I always keep the thought that life, this is also such a clichéd concept, that life is like a zebra, that is, there is a black stripe, there is a white stripe. And I began to notice it too, it seems to me that maybe this is self-hypnosis, maybe, I don't know, it's more like you, as a psychologist, will say. (laughter) I also began to notice that yes, there are some lows in life, but there are always ups and downs in life. And... (..) And the same goes for good and bad things, for me too, that is, I have neutrality here, but in life, yes, there are some good and bad moments, just even bad ones moments, I never call them bad, I just think that this means now is the time, some kind of quiet, calm life, or doing nothing, or I don't know, it means that life is telling you - rest there, devote time , I don't know, some family issues, don't engage in public affairs. There and then next week you will have five new acquaintances there and ten new projects will arrive. And this is how it usually happens, I don't know, I was there (..) about two weeks ago, I think somehow, and when autumn began, probably at the beginning of September, I was just moping, moping, I think - nothing It turns out that it still doesn't seem to work and it seems like my colleagues and friends also have problems, somehow they need to be helped, no one helps them, and I can't help them either. And now, October has arrived and there are just so many different projects and you're thinking, how can I get rid of them as quickly as possible, from all these projects, because somehow they all just landed on your head, and you don't understand what to do with them? . (laughter)
M. But in general, this is the period before yours, before your state now, right? M. Yes, there was also the military registration and enlistment office story. M. Tell me more about this. M. Well, I was in such a disgusting state of mind that the doctors and the military commissar said, no dear, you are somehow really bad, let us observe you in the hospital and sort of decide whether you need to go there or not. In general, I spent 7 unforgettable days in the Gannushkin Design Bureau, well, I lay there, I just lived there, like, I don't know, in a hotel. Here. And... Well, I was declared unfit, given category "B", I never left to serve anywhere. M. How would you characterize this period of your life? M. Well, these 7 days, it would seem, should have been quite difficult, but the only difficult thing was the fact of being there. The stay itself, in fact, gave me some kind of respite or something, because it was even harder at home then. Here. M. So it was some kind of escape? From what was going on at home? M. Escape, escape. M. What happened, how did you react later when you were declared unfit? M. Well, it's a relief, because if I left, I would feel even worse.
M. Yes, then you can move on to the next point. M. Choice of lifestyle and leisure. Well, this is where it gets more interesting, by the way, because I decided to change my lifestyle. He was like this, he is like this now, more sedentary, not very active, I found myself in such a rather interesting situation that after my previous job and previous company, all my interests simply died, because all my time was taken up by this sweatshop work and gatherings there with drinking there and so on. The rest just somehow fell away. And now I think what is interesting to me, what is not interesting to me, I restored my physical activity to a minimum there, in the future I will... In the future I will return to sports. I've even already decided what exactly it will be, what it will be.
M. Completely, I don't remember the last time I drank. In fact, with addictions everything is very interesting, I can, as if I don't want to and I don't. I also tried smoking then, but I didn't understand

this joke at all, to be honest, I just started and ended. Moreover, alcohol also makes me feel bad. Because alcohol is such a depressant or something. That I won't go back there at all. But I didn't have time to develop alcoholism, I... I don't have that.
V. Well, yes, with health, well, I'm generally not the healthiest right now, I have allergies, and a bunch of other diseases, but our medical system, it's usually in no hurry to help, and usually, until you get completely ill , somehow they're just trying to tell you, like, go drink oak bark there and everything will be fine with you. In general, my health taught me that no one except me will worry about my health. Therefore, you need to show maximum will to get a referral, to get the tests that are needed to get a referral to a research institute, and so on. And there are elections, that is, here, too, it's probably difficult to implement, that is, to withstand the doctor's pressure, because the doctor has authority, that is, like, are you going to teach me, puppy? You (npt) like me, did not undergo residency training. But, you need to show pressure, I then wrote the first complaint in my life to healthcare, to the Ministry of Health, here. This is just such a space of struggle for me, health, so I can say that this is... M
A. This means that what happened, well, I had certain health problems, (..) they are related to reproductive function. She was questionable to me. Accordingly, I needed to get a referral for surgery, this is why the health sector is such an important thing. So, I needed to get a referral for surgery and it's very difficult to do, it was, well, this whole story began in 2019, that is, the diagnosis came to light. So, I tried for 3 years to get a referral, and at one time I received it, but then I got sick, it expired, and they didn't want to give me a new one, so. Who were the participants in this situation? Well, the main participant in this situation was me, naturally, and doctors were involved in this situation, but they were more like passive observers than participants. I would say my mother participated more, because she asked some doctors she knew there and so on. My girl too. Where did this happen? So, okay, let me tell you a little further, in general, I tried to get a referral, I didn't succeed, then, in one private clinic... Well, in general it turns out that this operation is very rarely done without any additional shady payments, that's it. And I, I have a principled position that I don't give bribes for anything, well, in general, I don't want to violate this life principle. Here. Well, in the end I decided to do this operation for a fee and it was unsuccessful. And if... Which I perceive, in fact, as a wrong choice, that I probably should have taken a more careful approach to choosing a doctor.
Q. A year ago. A year ago I had this operation, here. Then I felt pretty bad for six months, well, it was very bad for probably three months, so then I made a decision for myself that, well, sort of... Well, first of all, I don't blame myself for this, for the fact that I there may not be enough... First of all, there is no guarantee that if I tried another doctor it would be better. M. Well, yes, there's no way to check it. V. Yes, there is no way to check. Here. And I was guided by the recommendation that was given to me, that is, in principle, I'm not just like - oops, I'll go to any doctor. I've really come a long way, but the fact that it ended in failure does not mean that the path was wrong. M. Well, yes, it's not your responsibility. You did, you approached this situation responsibly, as far as I hear. That is, they did everything. V. Well, yes. So, in this regard, I calmed down. At first I kind of thought, damn, maybe it could have been better, then I thought - no, better, well, much better, as if I did everything I could, for my part. This is the first moment. And then, then I decided that I needed to get a good examination now, that I could... And then I kind of asked myself - what can I do now, that's what I need to get examined again now, I started knocking out all sorts of directions, there same level of hormones, what can be done about it? Here. And I began to move in this direction, received a referral to doctors, so. And then I started dating the girl I'm dating now. When I was just going through the referrals, and before I started dating her, that is, well, it was as if we had already started to have some kind of romantic relationship, so. And I tell her that well, right now I'm not at all sure about any sexual things that I can give you, so I say that let me first go to

the doctors, they will prescribe me some kind of adequate treatment, and.. It's just that if I'm in a relationship, then I will feel uncomfortable, that maybe I'm not giving something that I should give in terms of my ideas about relationships. Here. And in general, somehow in the end I... By the way, this motivated me to speed up in that direction, to move, specifically to go to doctors faster, and to get directions with greater intensity, that's it. Well, somehow we continued to communicate with her, with this girl, and in the end all my functions returned to normal on their own. Here. Yes, but this happens, there are paired organs, the functions of one are taken over by the other. Here.
M. Tell me, do you remember the transition from your state of complete hopelessness to the fact that I can do something and in general, well, I can be responsible for my life and can take some action? So what was this connected with? Any external circumstances or more of your internal transformation? V. Tax. (...) Now, I'm trying to remember. So so so. (...) Well, at first, yes, I would probably describe it this way: at first I was just in shock, I didn't understand what was happening at all. Here. Then I began to feel the consequences, that I began to gain weight, and then I began to worry. Well, that everything is not going according to the best scenario that could have been hoped for. Here. Then, it's not like there was some kind of sudden event, it's just me, this question bothered me, it's like, I like to talk to myself, so. According to the method, like these two chairs, like what are you experiencing. How would I do something like this? So, this guy talked, so, okay, what do we want? We want to worry, or we want something, not to worry. Here. Let's think about what we can do. So, well, we, I mean, I just address myself like that, (laughter) here. M. Well, maybe some parts of you. V. Yes, yes, we are... M. They decided to act together. V. Yes, yes, yes. Well, yes, we are me, torn apart by the contradictions that exist within me. Here. As a result, I talked to myself, came to the conclusion that I... Yes, another thing... Yes, I remembered, I was very worried, really very much, and it always scared me... And I was even worried not so much because of what happened, but because I didn't understand what to do next. Here.
M. So the uncertainty was scary? V. Yes, I didn't understand, that is, which doctors to contact, where to go, that's it. Both then and there there was still such a problem that everything is very slow in our healthcare system, that is, there, for example, they give a referral, but the appointment is only a month and a half later. I'm like damn, what am I supposed to do for a month and a half? I, I'm very worried about this, but I can't do anything other than this recording. M. Yes, it's terrible. V. Well, that's when I started writing complaints, this greatly speeds up our healthcare system, by the way (laughter). Here. Moreover, even those to whom I did not write, you just tell them that you have already written one complaint, immediately become more and more talkative. Here.
M. Do you have any hobbies or interests? K. Yes, there is, this is football. (?) I used to play and watch, but now I just watch. There for health reasons. Problems have arisen, which is why there are various injuries that are now preventing me from playing football. Here. From sports I would probably say table tennis, in addition to this, reading, various things, including scientific literature, geography, waste sorting and the environmental agenda. Then I'll probably say, (..) well, let's put it this way, yes, the fight against domestic violence.
Well, next is probably health. A year ago, well, a little over a year ago, (..) I had an operation there on my leg, to straighten my toes, that too, it was a necessary operation, and after which there was six months of recovery. Here. And... M. Necessary, that is, it really interfered with your life? Has this problem been around for a long time, or has something happened? K. Well, about two years ago, even 3 years ago already, it appeared, at first I didn't do anything, it went away after some time, there after ointment or some other such exercises, but after that the condition began to worsen, and It got to the point where it was painful to walk. Here. And so a decision was made there, here. And this summer I also thought about having the same operation on the other leg, but now all this is being postponed for me. Perhaps next year, perhaps even the next year. Because

there are (..) various issues, including financial, time, because you definitely need to spend a week there under observation in the hospital, and after that you need to wear special shoes, and now it will soon be quite cold. It will be uncomfortable to walk in such shoes, so I am putting off solving this problem.
Here. Finance. Well, here the choice is probably only within the framework of finances, it's about health and about resolving some issues in this regard. M. Is this about leg surgery? K. Yes, including, plus other various problems, there in the summer I decided to do dental treatment, so. Also when it started to become quite expensive, and then other ailments appeared that needed to be urgently addressed medically, so. Therefore, dental treatment there, too, has been postponed and is being postponed there indefinitely.
M. Yes, Dima, thank you for the detailed story. I have a few clarifying questions. Firstly, I notice, well, naturally, here there is a moment of professional sociological education, albeit lasting one year, that you use a lot of terms, and with them you seem to indicate some kind of processes that arise in your life, but according to Basically, I have the following question, sometimes you say, for example, that there's this, this stupid procrastination, or... That is, you give some kind of assessments, and in connection with this it's interesting, but you're with someone... Are you discussing it now, or have you discussed those difficulties, including psychological ones, that you have encountered, and sometimes, well, naturally, they arise in life? D. I discuss them, but I discuss them mainly with friends. With my family, (.) I don't have the habit of sharing anything in particular and asking for advice, for some reason I have a big prejudice in this regard. It just seems to me that older people, my mother is a provincial teacher there, she is unlikely to understand (.) the things that I can tell her about. And you don't discuss it with friends, but only after the fact. Now here, with newfound acquaintances, I can discuss what I went through, but every time I go directly through a crisis, I usually don't share it with anyone, well, also probably because I don't think that anyone something can help me with this. Therefore, this is only after the fact.
y, the choice of lifestyle and leisure, well, perhaps nothing has changed here, except that I began to drink less, because the first six months of my studies, when you constantly sit in the dormitory... Here, in fact, besides (..) drunken pastime, You don't have any special choices anymore.
K. (.) Yes, these are some restrictions on the part of the parents, because of vision, for example, you couldn't play on the computer for more than one hour a day, there are some restrictions on the time spent on the street, that is, when to take a walk there , restrictions...
M. I understand. And here's another thing about your vision, has it changed somehow now, or what's wrong with it at the lyceum? K. Well, in high school it got worse, but there it's also hereditary, and because I read a lot (.) in the wrong position, it got worse, and it's still bad now. (.) For now.
M. Did you have any prohibitions in childhood that you definitely shouldn't, both in childhood and in adolescence? S. Only if you consider it an allergy, that's all. M. That means taking care of your health, right? S. Yes. M. Was this somehow instilled in you by your parents and did you want to break it, or how did it happen? S. No, it's just sometimes... Well, it's like I'm allergic to nuts, but I really like nuts. But I understand that... What will be the consequences. And, well, like, as a child, I somehow didn't attach much importance to this, but I go to camps, let's say some sanatoriums, my mother always tells me - do you remember that you can't have nuts? Do you remember what will happen? And then I, here. And in terms of alcohol and smoking, I was somehow not prohibited at all, but I also had no desire. Here. And my parents forbade many of my friends and now they are just having a nightmare there. M. That is, on the contrary, the ban gave rise to... S. Yes. Because with this ban, they seemed to have more and more desire to try. They didn't forbid me, my mother always told me that you can try, but it's not a fact that you will like it. Here. I tried alcohol for the first time at the age of 18, at graduation. M. That is, quite recently, right? S. Yes. And somehow, I

don’t know, now I drink a maximum of champagne, wine, and on holidays. All. Smoking, I have it in general, I don’t even have the desire, I’ve been offered to try it so many times, I say, I don’t know, I don’t want to. There is no desire at all.
decided to carry it myself. (..) Well, I went up to the guys, near the tents, to the boys, I said - boys, can you please help me carry the suitcase, I can’t do it myself. They told me - no, we can't do it now. I say, okay, okay. I'll carry it myself. Here. And somewhere in the middle of the journey, I already realized that I simply couldn’t cope, that this rib was starting to ache a lot, there was pain. And I wrote to the chat with the guys, I said - guys, the boys really need you now, please help. Here. And no one reacted at all. Here. And I just walked, roared, carried this suitcase, so I carried it. But most of all, I don’t know, what made me laugh was that I was walking, and so I met many young people, they were just walking like - damn, she’s so cool, really, strong, independent, carrying a suitcase herself, in general. I walk and think... M. And it was very painful for you. S. And I walk, roar and think - you didn’t think of offering help, somehow, I don’t know. I even, I don’t know, I always somehow offer help... (..) I, I don’t know, I’m used to doing this, so. Then I just went to the doctor, they told me a lot of things about how I shouldn’t carry heavy things, but I went and did all this, that’s it. So. Do you often think about it, about this situation? (.) Well, recently, often, because it happened recently. (laughter) Here. I told my parents, my mother said that everything can be expected from me, and what emotions arise? The emotions are so mixed. M. Can you explain? S. Well, in the sense that yes, I myself was able to convey all this, but it was to my own detriment, but also that, as it were, I don’t know, we were always told from childhood that boys should help girls and all that. And then they just go like this...
Well, perhaps, when my neighbor became ill, she began to choke, and... That is, a person is suffocating, he doesn’t know what to do, this is his first time, and it’s like... That is, I called an ambulance, there I woke up everyone , she said that yes, we started googling something together, what to do and so on, that is, as if she had something like this, nervous, panicky, that’s it. But then she went to the doctor, well, it was like, you know, an independent decision that at the moment I should help the person. Because she can hardly help herself. M. This was probably a very stressful event and somehow you reflected on the moment when - now I’m doing it, or did you just start doing it yourself because you realized that the responsibility was only on you at this moment? M1. I guess I just started doing it on my own. M. Do you think this situation played any role for your independence, or did you simply show what you already had? M1. I think I just manifested what I already had, because my sister is often sick, (laughter) more precisely constantly, so I kind of got used to packing things in bags when I have to go to the hospital, that is, for me it’s a familiar situation, so it was sudden in terms of its appearance, but in terms of (..) how should I say it, in terms of factors, it was similar to many situations in life.
M1. Now I'll think about it. (...) So, well, one successful one. (..) I was in the eleventh, no, tenth grade, I wanted a dog, and at that moment I already understood that I definitely (xx) would not stay in my hometown, in Rostov, that I would definitely move. It doesn’t matter which college I go to, but I’ll definitely move. And... But I really wanted a dog. And as if we were looking for a dog, we found it, we had already paid a deposit, well, like, in a few days I have to go pick it up, and I understand that how can this be, then I will leave the dog with my parents, because I will live in a hostel , well, for sure, I had a choice either not to get a dog, and let’s say, not to fulfill my dream, because I never had my own dog, we have a lot of dogs in our family, but we didn’t have our own. And no one knows when I would be able to get myself a dog. Or cancel everything, let’s put it this way, and simply not burden the parents. And that was the choice. Either you blindly follow your dream, then you burden other people because they need to take care of your animal, or you care about other people more than your desires. This happened in the tenth grade, I was probably 17

years old at the time, and me, my mom and my dad were involved in this situation. Because my sister doesn't live with us, therefore she won't take care of her. Where this happened, this happened in my hometown, this situation made me feel like I needed to decide something, that I needed to do something and that I was afraid of making a mistake. I'm afraid of ruining the dog's life there, I don't know, causing some kind of trauma to myself, or burdening my parents with this too. That is, the situation was not pleasant. The choice was not a pleasant one. But now, (..) I treat her calmly, I can't say that she directly somehow excites something in me, probably, I just remember with some pleasantness all this then... All These are my decisions that led to the fact that I now have a dog. (..) It evoked different thoughts. (...) Don't even know. I think, probably, I was just very afraid to hang some kind of responsibility on another person, I was very afraid that the animal would forget its owner... And it's not very pleasant for me either. (NPT) kind of like for myself, but like for others, too, so. Now this gives me (..) also mixed feelings. Well, that is, I don't know why I did this then and decided to get a dog after all, and I don't know what I would do with this situation now, well, that is, it's like you just decide in the moment, yes or no, and probably Then I just let go of all the consequences and lived in this moment, that yes, now I want a dog, now let's live in this gorgeous moment, and then I will accept the consequences and decide what to do with them. Here. But I think that it turned out well, because I love her very much, I have a gorgeous friend who will always hug you, lick something, and so on, make you laugh, play with you, that is, how These would be very pleasant moments, memories, so I think that I made a good choice.
probably my sister also has health problems, so she, a little like this, somehow maximally, (sigh) I don't know, reaches maximum heights during education, and therefore, probably, this is also a little bit of my responsibility, (laughter) also everyone, and they also pay a lot of attention to her education, even more than mine, because it was more difficult for her to study, but certain hopes were probably pinned on me in this regard, but I'm not exactly (npt) on this topic of approval, that you should do something there, always do everything well, I don't know. (laughter) Well, yes, in general, this was also important to my grandparents, but what my grandparents on my father's side, basically, my grandmother was more likely to just say, go ahead, well, it was important to her that I do in basically anything, and I moved somewhere forward, and managed to do everything. But it didn't matter to her in what area I was doing it. Here. Probably so. M. But it turns out that both pairs of grandparents have higher education? I. Yes, everyone has a higher education, yes. M. I'll carefully ask about my sister: what health problems did she have, were and still have, what is the reason for this? I. She has autism spectrum disorder. But she is quite highly functional, that is, there is no such thing that she, I don't know, it's probably not very noticeable, in terms of... (..) For people who are unfamiliar with this, they probably won't immediately understand that something is wrong specifically. That is, they may think that she communicates in some strange way, or something, I don't know. Well, in general, not so much that she couldn't study, for example, but in life it interferes with her. M. But you said that due to the fact that your sister has such difficulties, sometimes they even paid less attention to you, and so you, well, how much did this bother you? That is, did it evoke any emotions? I. In general, no, probably (laughter) only because they always paid a lot of attention to me, and sometimes, I would say that it was even some kind of overprotection, so for me, I don't know, it's just very difficult to assess the effect and the cause , that, for example, they looked after me a lot, because my sister has difficulties, for example, for a very long time I was not allowed to go anywhere on my own, (..) well, on the subway, or on some public transport, and they were always driven, and it annoyed me because I wanted to travel on my own. And I don't know what this is connected with, maybe because my sister couldn't drive for a long time, and I started driving earlier, my parents were afraid to let me go. It seems to me that some of my parents' fears, as it were, related to my sister's health, are transferred to me. Well, at

least it was like that before. Probably not anymore. But maybe, maybe I'm just thinking, in general, I've never had the feeling that they pay more attention to my sister, because I also received a lot of attention. And vice versa, probably at some moments it was good that this attention did not go to me and I, I had more freedom.
M. Did you feel older precisely because she had just such health difficulties? I. Yes, because for quite a long time, but even now, she still doesn't have it so strongly, for a very long time she behaved as if she were much younger. Therefore, at some point, well, I remember, right when I realized that she had some peculiarities, I asked my mother, by the way, I even remember this moment, I was driving, we were going either to a dance or with dances, and my mother began to tell me, I was about eight or nine, and she gave me a book to read, well, it's such a cool book, "Autism through the eyes of a sister," it's like from the point of view of a girl whose brother has autism and she tells . I think yes, it's quite similar, because some patterns of behavior are described there very, very similarly. Here. And when I realized this, I somehow began to relate to her easier, because when you know why she behaves so strangely and it annoys you, and it's not clear what to do about it, when you know it, it's easier. But still, I noticed that my sister acted as if she were younger, as if she were younger than me. Therefore, I didn't have the feeling that she was older, and that somehow... In general, I was probably ahead in terms of development after all.
Regarding health, there are probably no solutions. At least nothing comes to mind. There, every year you just go to the doctor, and he tells you what to do and that's it. (laughter) M. So you are generally calm about your health and therefore somehow don't even remember? I. Well, I probably haven't had anything like serious health problems lately, if something happens to me, I go to the doctor, but I don't know there, I recently had poisoning, I go to the doctor, he me right away (npt) (laughter) but there were no such, well, particularly important ones, but probably if I had had any health problems lately, I would have remembered it better, but so, (laughter) nothing there was no specific one.
M. But it seems as if you have done a lot of work compared to the way you talk about your adolescence and the way you behave now. You are very open, you are very emotional, and talking about such experiences is a great courage. In fact. N. (laughter) I have chosen a psychotherapist several times and now I finally go to a normal one, and everything, plus or minus, yes, is being worked out, so it's just... Well, that's exactly what I'm talking about, about the fact that from the age of eighteen, I can finally make choices myself, and I can, as it were, in an emotional way, that is, choose what I feel, what I don't feel, and what, I can behave this way and be like this, that is, in some moral things, and in some actions. That is, I decide where I will do the actions one day, and I chose this, and I will, well, kind of lead you like this. And be like that. This is also like a choice, in my head, it's considered, because, well, just then, all these things, they were very strong, I cried there for months, and in general, I felt even more downtrodden, but fortunately, some things started there. then a fairly adequate relationship that lasted quite a long time, and this somehow made me feel that I was not at all... There is someone who loves me, someone who appreciates me, the same one like this... Replacing unconditional love with parental love, that's it. And probably, in parallel with all this, probably in parallel with this, there were also elections in the 11th grade... M. Can I have a second? Tell me, do you attribute your transformation to a greater extent to psychotherapy, or perhaps there was also some event that made you look at yourself differently and begin to behave differently. N. More likely, to a lesser extent with psychotherapy, but that is, I started adequate psychotherapy literally four months ago, because before that I just went to psychotherapists, and somehow, well, I couldn't find someone with whom I would be comfortable, Here. So I associate it rather with the fact that the most important thing is that now I can, since I was eighteen, I do not depend on my parents, that is, if before that my parents could forbid me

something and somehow control me, now, well, this is absolutely not the case, and we have some kind of, well, adequate distance, Well, you know, there is an expression, healthy indifference, something like this, probably with us. It's clear that I love them and this is unconditional and absolute, but how could I...
M. And after 18 years, what important elections did you have? Besides work. Maybe in some other areas? Independent travel, I heard this spring, maybe something else? Choosing a psychotherapist? N. Yes, choosing a psychotherapist. That's for sure. (laughter) An important choice, I'm very glad that I found my current therapist, she's very strong there. Yes, independent travel, because at first I started traveling around Russia, it was also funny, of course, because I turned 18 and Covid began. Like hello, thank you. Nastya, who had never been abroad before, or even in any other cities, here. Yes. So I myself began to travel slowly around Russia, sometimes in a company, sometimes on my own, and then I decided - damn it, I've never been abroad. Here. And yes, in general, I'm very glad that I went to Istanbul, so. Because I went there myself too, that's it. That is, completely alone, not knowing much (laughter) English, that's it. But it was great and I'm very, very glad that I went after all, because well, that's exactly my trip, I bought tickets there a few weeks before February 24, and the tickets that I repurchased were canceled 10 thousand times , and I was already thinking, damn, how am I going to go in such an atmosphere, but I went anyway. And I'm very glad, because... Well, at least this ticked some box, because you still felt as if you were in some kind of geographical cage and (npt) get me out. And this summer I also went to Dagestan, on my own. And this was also wonderful, it seemed to slightly support your own feeling that you are doing something with your life, and you are sort of solving not only some work or romantic processes, so. But you also allow yourself to receive some kind of pleasure. Here. Yes, what other elections, well, probably a partner, we still have to talk about it, because this is my first breakup with that young man with whom we dated for two and a half years, this is probably an important decision that I accepted. And after that we dated for a year, because I, in fact, I left him for another partner, with whom we were in a relationship for a year, it was such a very cinematic relationship, with... I think you know the expression Dead Inside, here, (laughter) this is it. (laughter) So, in the end, we parted ways, well, that is, I can generally say that he abandoned me there... Because of his own pens, because he was somehow against me projected, as they say, and this was also a rather difficult period, just around March - April, all this was also layered on February 24, and (..) there I did not make any choices, (laughter) this is exactly there was a moment when I directly felt that I had lost some control, and because of this, well, it was really very difficult. Well, it's also related to this, that is, I found a psychotherapist right on the same day. Here. And thanks to the fact that I decided to find her at all, and because I found her, I somehow, well, generally survived all these moments, because of course the first separation was very difficult, well, actually without your will, which was done. Here. Well, then there, after some time, it seems that I met a young man with whom we now have, well, some kind of relationship begins, this can also be considered some kind of, well, choice in the romantic part is important, because Well, there is 50 percent of work that calms you down, and there is also, well, it's clear that it's not 50, there are other breakdowns, probably 30 percent of work, 30 percent of relationships, 30 percent of friends, and then there's 10 percent, this is some kind of totality , oh well, the family still needs to give some part of the interest, so. Well, that is, I just remember that just when I was in Dagestan, I felt very happy, because finally everything in my life, plus or minus, seemed to work out, and this, well, if you look at all these segments, Well, they're pretty good. Here. That's why...
N. Well, then, I think that I can go back to the choice that I made when I was 16, when I was in the summer, when I decided to go to the young man with whom, who hinted to me for sex, for some kind of romantic relationship, that's it. And to his friend's house. Well, accordingly, I was 16, that is, what year was it, 2018, yes, it seems. Yes, summer 2018. Here. (..) Accordingly, I was there,

this young man was there, and his friend was there. And so I ran away from home to spend time with them, because it seemed to me that it would be cool and that I would probably get closer to him, and indeed, he would kind of like me as a person, and that's how he would like me probably appreciate it more. Well, in principle, I liked the feeling that he was paying some attention to me, that a person seemed to appear who appreciated me, at least for something. Here. Maybe sexual desire, and it seemed to me that, well, in general, I could probably somehow, well, show interest, and he would appreciate me even more, but for something else. Here. And all this took place in the Moscow region, at his dacha, in his house. And... Well, it happened, (laughter) how to describe it, I just don't want to go into any super details, but (..) in the end, after we first talked with his friend, who told me what - there with these vodkas, so, and then this friend himself came, oh, not a friend, but the young man himself, with whom we had some kind of fuss, so, and he took me upstairs, and then I continued I remember it very well, (..) just in very small snatches, here. But it was all connected with some kind of (sigh) (..) well, sexual actions. In the end, I don't know how he would have entered me or not, but he tried, let's say so. Here. And what kind of feelings this situation caused then, obviously - not the most pleasant ones, well, that is, I was probably in some kind of teenage age, so that everyone, I was just, I was just worried about everything. Well, it seems to me that everything was just finishing me off gradually, that is, it wasn't so much the situation itself that was finishing me off, that is, the fact that... Although, well, in general, it's difficult for me to separate this for me, the fact that we then stopped communicating with him , that is, he seemed to write to me at the beginning, well, that is, maybe he wrote there twice, so. And that's all for me... (..) Well, sort of, the fact that he's no longer interested in me at all, and the fact that he absolutely doesn't need me, and the fact that no one loves me, doesn't appreciate me, I I felt absolute loneliness. I remember that I imagined that I, firstly, well, that is, I just had pictures in my head, like I was alone in the middle of a white sheet of paper, and in general there was no one close to me, as if my parents don't give a fuck, my friends and I are so close We don't communicate, that's it. And, well, that is, it just felt like such total loneliness, rather, this whole situation led to these feelings, so. And what feelings now, (sigh) does this situation evoke, now, for a second. (...) (sigh) Well, probably (..) I just thought it through, thought it over 10 million, billion times, turned it over in my head, and somehow the psychotherapist and I seemed to have worked through it, give or take. .. And now I just feel some kind of, I don't know, sadness, sympathy, probably for myself, but it's global, because, well, obviously none of this would have happened if I felt somehow there much better, that is, as confident and calm as she is now.
M. Yes, of course. If you think that's it, move on. A. Relationships with parents. This is a very difficult question, because... Well, I have always had a very (..) (h) great attachment with my parents, because they literally did not let me go anywhere, and it was this year that I decided that I would completely separate from them, I worked with a psychologist about this. And... (..) And I tried to distance myself from them as much as possible, because I understood that I was already independent, I was already old enough to make decisions on my own. And, (..) well, not even listen to any advice, realizing that my experience, conditionally, of living in Moscow, studying and working is completely different, like theirs, in another city, and since we are completely different generations , because, well, my parents are adults. Here. And in this regard, it turns out that relations with parents may have cooled somewhat, and they are not as active as they were before. (...) Are there any additional questions on this point?
And it was six months of hell, and some kind of moral torment, when they just constantly call you, I don't know, a person who doesn't know how to do anything, can't do anything, and that everyone has already written a diploma, but you haven't, and it was just what Here's a psychological panic for six months, that's it.

M. Yes, there is such a thing. Health? L. Health. Yours? (laughter) M. Yes. What were you even thinking about? L. Well, it often happens to me that I force my loved ones to take care of their health. At the same time, I don't pay that much attention to it. Because for example, there, I don't know, I think when I lived with my parents, someone's blood pressure would rise, and I almost called an ambulance, but they didn't do it. And in the end I understand that I did everything right. But as for mine, I'm like, damn, again I feel sorry for the money for the dentist. Well, let's probably say here then... (..) Probably a five, that is, somewhere in the middle, because, well, as far as it concerns words, I understand everything perfectly well. But when it turns into business, it is much more difficult. For example, I couldn't get to one doctor for 3 years. But when I got down to it, I immediately realized that thank God. M. Okay. Job?
Well, not a profession, but probably just a hobby, I just understand that in fact all my energy is there, and when I step over myself, well, I forget to do this creativity at all because I have no time, but I begin to suffer from it. Sometimes I have some kind of blues there, I can even get sick, psychosomatics starts to turn on. Well, in general, these are the moments. M. Katya, tell me, have you ever had a consultation with a psychotherapist? K. Yes, by the way, it took place at the Higher School of Economics.
M. And in what terms is health? Can you cure yourself of an illness? Or make an appointment with a specific doctor? M1. No. (laughter) No, just decide that I need to understand on my own that I don't feel well and go to the doctor and understand which doctor.
M. I see. Great. Do you have any hobbies or hobbies now, do you work in the summer or in general? N. Well, I worked, but I worked in Yandex, in Yandex.Textbook, in an educational project, then I left there because it was... I worked in support, and in computer science in Yandex.Textbook, Well, in computer science, I left there because I had family circumstances there, and I had to write a diploma and everything else fell in, well, just because of stress and plus the manager changed there, so it didn't suit me and I left. So, I was in Moscow as a psychological internship in my fourth year, I did it for a charity foundation in "Shalash", so, I don't know whether you know or not? M.
M. Well, about this and that. N. Well, in general, I'm interested in the sphere of parent-child relationships and the sphere... Well, in short, working with children, with teenagers, as if I, which in my experience was not very clear, seems to understand what to do as a teenager, so I kind of do everything... Well, I just like this age, that the worldview is formed there, and so on, and so I kind of want... I would like, for example, to have such a person, to To whom I would come and somehow tell everything without judgment, for example, he or she would simply listen to me. Well, in general, I would like, for example, to have such a person, if anything, like a school psychologist, so. At my school everything was like that, well, very like that, not humane, that's why... Well, in particular, that's why I went to the psychology department. And in general, I want to be more careful with children and teenagers, so that they can be treated, and, well, treat them in general. That's why it's like this.
And there's this very thing, I don't know when there will be grandchildren, when I'll get married, that is, there's this very insistence on a certain way of life, there are some expectations from the parents, from society, and I feel them, but at the same time I kind of went through psychology and psychotherapy, that is, it all seems to help me, somehow my beliefs, to live according to my own beliefs, and not social ones. But I still feel like it's frowned upon. And for example, when you don't listen to someone, it's condemned. There, when you are, as it were, in the minority, this is also condemned, that is, it seems even to me now, this situation, which is the war with Ukraine, that, as it were (.) some people who are vehemently opposed, or something like- then, in general, some people stand out from the masses, they are always, well, they are always perceived negatively, it seems to me. Here. And when, for example, you are different, it's just somehow

scary as if people can be different, and this seems to me to be such a problem that it happens because of this condemnation that you are somehow different. Here.
And then, and also the fact that I was probably not afraid to take specialized mathematics, because to become a psychologist you need specialized mathematics, and it was like, it was very difficult for me, and every time it ended... Andrey, my husband, he seemed very He's good at math, but he tried to explain it to me, I didn't understand, it ended in tears every time and all these tutors and all that, I didn't like it at all and I literally forced myself to learn it. But I'm like, I want to take psychology, I'll pass and I'll never need it again. Well, how important it was for me to pass it well and then I passed it, and I, I had such, well, it seems to me that such a low score for admission, but then I was very... At first I was upset, then I was very I was happy because it was an entrance exam for psychology and that I was going to get into it after all. And I was very happy about this, and my decision, and the fact that I passed these points, and my decision to go to the open day and to enter the Higher School of Economics, although I did not see other universities, that is, I did not go there and didn't scout anything. I sort of decided that I needed to go there.
M. What about making everyday and important decisions? Here, in fact, we are, of course, slightly duplicating what we have already discussed. N. Yes, yes, I understood. (..) Well, I think, it seems to me, let it also be seven, because I, well, in general, it seems to me that this is a process that there are no correct ones... In general, I'm kind of really worried about there right, wrong there, or how it will affect others, or something like that... Well, in short, I worry about this in every possible way, and it seems that the more I work there with a psychologist, well, the more I see, that no matter how people react normally to my decisions, the more calm I am about this, the calmer I begin to feel about it, so how would I consider that it's seven, there I'll just be there later, well, what's my attitude specifically towards making a decision , it's very similar, it's quite saturated with anxiety, so I wouldn't say that it comes too easily to me, but I kind of understand that I always make adequate decisions, plus or minus, I just have problems making decisions, that's it. And the choice of lifestyle, leisure, well, right now I would probably say that (..) six, because I think that (..) well, I just feel that I'm a little burnt out and tired after studying there, after school, when things weren't very good there either, at the university, when there were some difficult moments there, that's it. And it seems to me that I just need to recover now, just somehow settle down, that sometimes I get shaky, like how I behave there, or how I need to do or something else, well, like a lifestyle, and for me, I'm just now focusing on the fact that I need to rest, and otherwise be more careful with myself. Here. And about leisure, I don't really like spending time in company, I rather like one-on-one communication, and well, I kind of, (.) well, I don't do any supernatural leisure, like, for example, I knit there, or maybe I have some kind of hobby that is very accessible, well, drums are not very accessible. That is, I disturb everyone, and it happens that they irritate everyone, but as if I could occupy myself with something, I don't feel like I'm very bored, that is, in general, in every possible way... I sometimes scold myself myself for rest, that is, it's as if I can't rest, because I'm not tired and all that. But this is again a balance, there is a work life balance and therefore... (...) Well, about leisure, I meet with friends, I read books there, watch films, some educational things there, well, educational videos on various there platforms. Well, in general, I do, it seems to me, what I want, what interests me, and it seems to me that this is enough. Here. Romantic relationship. (laughter)
M. What do you mean, what didn't coincide? I didn't quite understand? They said that it was possible, but... N. No, rather that they... Well, for example, dad said that you should, for example, not lie, that it was better to be honest, and then it turns out that he has a second family there, that he him there... Well, in short, (laughter) some secrets from us, roughly speaking, that is, that he says one thing and does another. And that is, as it were... It doesn't work out, then there is no clear

picture, you still understand that there is this duality, and not... (..) Well, what is it... And you begin to think like about some of your actions, that's it. That's what I'm talking about, that there weren't any where, for example, they told me - go and do it, for example, my mother said - go there, do something, take an interest there, something else, if only I had such opportunities as you have, then I would have done this, that, that, but she, despite the fact that she seemed to have opportunities, she practically sat at home with us all the time, that is, she developed, some She worked through her own problems there, with the help of psychology she studied there, but she didn't seem to really realize herself and it was also like this was a call, like go do something there, realize yourself, but she herself didn't do that. And it was as if I was expecting that if my parents said something, then they would do it, but that didn't happen, and so I had a question, like, how can this be, like, people say one thing, but do another, And I had such a conflict. Here.
Well, that is, yes, you will go to medical school, Yes, doctors, doctors, this is always needed, well, they are like that, one of the pillars of society that will always come in handy, you will always need to treat people. Here. But it is very difficult to find, unlearn, firstly, spend a lot. And then, I couldn't answer why I needed this, so somehow, not finding answers, I slowly walked away from it. Well, it's a plus due to the fact that then my mother, grandmother is a pharmacist, my mother is there in the field of obstetrics, and somehow, knowing this whole kitchen, I didn't go there, so this is also an important, it seems to me, such a stage, when I reoriented myself, but it was as if it was unclear where. Further, my interests developed - social studies, history, well, closer and closer and closer I seemed to come to education and the humanities, to the humanitarian sphere.
A. So, training and professional development, I already said that. Health choices. If we talk about the second point, then for the last year, but probably I have had more changes in six months, because there was a change of place of residence and somehow I concentrate more on it. Because the choices that were made in the previous six months were not in favor of my health, but there the only important option, or rather there was a choice, was to go to a psychologist. To deal with this destructive story with the leader, with (sigh) (..) change of position. Here. And if you look more towards the physical rather than the psychological, then this is probably a choice in favor of taking care (.) of some kind about yourself, to searching for, well, understanding in general your health there through the body, changing a little nutrition, attitude towards sports, and so on Further. Well, something like that, I returned there again, to proactive history. Job. (..) One important thing, well, it seemed to happen to itself, it so happened that for a new position, again over the last six months, first there was the building of horizontal communication, despite the fact that there is a manager and subordinates. Yes, I am the director there, but these are still subordinates, a turquoise organization was built. There was this, well, how it was clearly articulated, said internally in the collective, in the team.
M. Basically from the elections. A. Well, after all, what is still very much unclear and difficult for me is the case of accepting responsibility for the whole, the position of a manager. M. Yes, I remember you talked about this in great detail. Yeah, that's what happened. A. This is really very complicated... Well, it seems to have been partially worked out, this situation, in psychotherapy, but not yet fully resolved on some emotional level, it also seems to me that this has created some kind of trauma, (laughter) here. In terms of what was wrong, why was it wrong, right? M. Yes, why the wrong choice, do you think? A. Because, again, it was probably from the position of - well, who else? Well, very often I have such a choice when there is no other outcome, well, that is, some kind of critical point. And only then do I, as it were, completely regroup, into some kind of monster, make this decision, well, that is, I say this - yes, I can, we'll do it this way, I'll pull it off, but somehow, as if at a critical peak, I take upon myself the whole responsibility that is possible. Well, as if this were directly noticed, and then it usually works out. (xxx) But in this situation, which was and where my choice was, as it were, wrong, I underestimated it. Well, that is, the gap

in this critical situation, the gap between what I can do and what definitely won't work out, it, well, happened more. Revaluation. (..) Here. M. Yes, but how do you feel about this situation now? What thoughts or feelings might arise about her? A. Well, it's cool that it was the first thing. Second, an important decision was made that in the next couple of years, I don't know, I won't take a management position at gunpoint. Not yet, well, no, I won't go there again for now. Just now I started, well, I kind of started to sniff out, if possible, from this state when you, well, got a very strong electric shock, well, you just intuitively won't get into it anymore. Here.

M. You can go straight through the list, you don't have to arrange them. K. Training and professional development is... Probably there will be seven, because (..) you always have to learn everything, and sometimes it's very difficult to allocate your resources and brains, (laughter) let's put it this way. And do one thing. Health is five, (laughter) it's either there or it's not, work is also six or seven, because... M. Wait, let's go back to health, making specific decisions. In terms of health. K. Regarding my health, everything seems to be fine, I feel good, but sometimes there are times when I get sick, (laughter) Covid and so on. And I try to recover as much as possible. But if I get sick, then I don't do anything, it's immediately minus work, (laughter) minus friendships, only the place of residence will be there then. (laughter) At maximum. M. What about choices, such as choosing which doctor you need to go to, or even understanding whether you need to seek help or not? K. Choosing a doctor, I don't know. I somehow... M. How difficult or easy is it for you? K. No, it's easy for me to choose a doctor. I just see, either from reviews, or using word of mouth, that this doctor, we went to see him, he's good. I'm so good, I'll go to him too and everything will be fine. In principle, this is how it turns out, so I don't have such a difficult choice here to choose a specific doctor. Well, usually, I just probably didn't get sick with anything serious, no matter where it depended on me... The doctor's choice is between life and death. Therefore... M. In general, you said that you rate your health as a five, then what is the difficulty in making decisions regarding health? K. Health, difficulty making decisions, (laughter) giving injections. This is where it's difficult. (laughter) Or force yourself to go... It's just that when you're sick, you don't really want to go out. M. So it's difficult for you to understand whether you need help or not? K. Yes, I can't, it's sometimes difficult to assess the extent of my illness, it just seems to me that even with a temperature of 38 you can still do something there and at work, everything is a doctor (NPT) in principle (NPT) and you can live.

M. And now I will ask you to tell me about one story of a successful choice, and also, please, in accordance with this layout from the chat. K. Successful... (..) Now, we need to think. (...) Something... (...) I thought, I think it's good that I changed one job for another. Before that, I worked in another place, not even at a university, but in a clinic, well, in a hospital. And this is very, well, at first it seemed to me that this is very cool and great, because the equipment is new, but then I realized that this does not give me any career growth and if I stay in this position, I'll be there (npt). Then, but this also happened at school, then they gave me... Work, a feeling of extreme fatigue, every day. And it's like (npt) Groundhog Day. And I realized that this had to stop somehow, otherwise I would simply burn out at this job. M. So you also seemed to be guided by your emotions? K. (..) Yes. M. It seems to me that you have someone else there? K. Yes, now there are students again, we should have opened another classroom for them. All. (..) Well, it seems like I told you everything. M. No, more about feelings and thoughts? K. Oh, (sigh) feelings, then this situation, (laughter) it seems to me that this whole situation sucked out any emotions and feelings from me at that moment, because working in a hospital is very energy-intensive. And now I'm looking at it too, the same thing, the only thing I've gained is experience, put it in my work book and just to fill out my resume. M. Tell me, Karina, how do you determine which choice is successful for you and which is unsuccessful? How do you separate them? K. (..) Successful, if I

feel some kind of return, I feel needed. And unsuccessfully, if I feel some kind of return, I feel needed. And it's unsuccessful if I feel like I'm wasting time and wasting it.
D. It turns out that at the age of 11 I was told that I had the fourth degree of scoliosis, idiopathic, and that this could no longer be treated, perhaps I should have started going to some massages there a couple of years ago, doing something, but I had already encountered with a severe form of this scoliosis, and they told me that... Well, I was again given a choice, I remember how my mother seriously decided to talk to me, at the age of 11, that Dasha, you have such a situation, you can (..) live with this scoliosis, that is, it's like, well, I would have developed a hump, very strongly, it had already begun to appear, or I would have to undergo surgery, and they would put me in a metal structure for life. Into the spine. And when you're 11, you don't really understand that they're going to do something to you now, and you'll have to live with it, well, for the rest of your life, and I made up my mind, and basically, my mother told me that I walked this path very steadfastly , and lay in the ward after the operation. The girls who were 10 years older than me were there, they cried, it was hard for them, and my mother said that I went through it all with courage (laughter). Here. This is probably also an important path. M. Yes, I really sympathize, well, it's really a choice, and it's such a significant one, and I think that you are probably faced with some of its consequences. Does this have anything to do with tennis or not? It seems like... D. My mother thinks yes, that it's because of tennis, but I think that... (..) Perhaps, but there is a reason, there is a reason. Perhaps this somehow prompted, well, aggravated the situation, but obviously it was not the reason. Most likely it's something genetic, perhaps I had crooked feet from birth, and they tried to correct them for me somehow, here. Did not work out. And you understand, yes, that when the weight is unevenly distributed, then all sorts of scoliosis results. That's probably because of this.
When I went to the hospital, I didn't immediately tell my parents, only when I felt good there
want to be the kind of person who (..) takes responsibility for all the negative events that happen to him, and for example (..) does not ask, well, that is, it's normal to ask for help, but when you really need it, that's it. I (npt) did this, and I started to practice it now, and if I feel bad, I don't call right away, but before I immediately picked up the phone, immediately dialed and cried. (laughter) And now, first I'll cry to myself, listen to music, maybe talk with friends about some abstract topics, and then, when I've calmed down, I'll share. Like this.
Z. Yes, yes, yes. Here. Choice of lifestyle and leisure. I really want (laughter) for this area of activity to change for me, because over the past year, all I did was study. True, I don't have time for anything else, I didn't have time. I'm trying to change, I'm trying to change this moment, I really want to do... Well, in general, I would like to do yoga, go to group classes, I realized that I really like it when (..) probably a new team appears, and there are new people, meeting new people, it's very interesting, it's something new and unusual. I would really like this area of mine to improve, because again, all I'm doing right now is learning. And this drains you and you need to recharge somewhere. Where can I get it? Well, I realized for myself that I would like to go to a slightly different space, to other people, and replenish my resources with the help of sports, yoga, etc.
M. Okay, let's now take a closer look at your elections, and now I want to ask about the elections that you may consider unsuccessful in your life, were there any? And why do you consider them unsuccessful? Z. (...) Yes, it was a bad choice in terms of health, so I already told you that I turned to a surgeon, and... Well, I regretted it. I had an operation that was not required at all, that is, in vain, they completely removed my nail plate just like that. Why this was done is unclear. When I came to a highly qualified specialist, a podiatrist, they told me that this was a complete disgrace, it was impossible to do this, and now we are correcting this whole problem. Here. That's why for me this is such an important choice, a health experience. And now I think this is very important. When

they wish you health, now I don’t just, well, health, happiness. Happiness, health - yes, this is what I need. (laughter)
M. Great, great. Yes, it seems we have now discussed some better choice. Maybe you can remember, for example, how a bad choice was made? For example, with an ingrown toenail, maybe you can remember your thoughts and feelings while you were going for surgery? Z. Yes, yes, of course. At that time, I was at home, not in Rostov, and we went to the hospital. In general, it turned out that I was immediately sent to a surgeon to solve this problem. I come to the surgeon and he looks at me slightly and immediately says - that’s it, operate. I'm like - what? And I had never had any health problems before. Everything was fine, no surgeries at all. Well, it was a shock for me, I was admitted to the hospital right away, that’s it, they left me, my parents brought my things, and the next day I was waiting for the operation, so. So it turns out (..) day X comes, the time approaches, they take me to the operating room, perform the operation, and the worst thing was that after the operation on the nail, well, it turns out that the nail plate was completely removed, and a bandage was wrapped around this, on this skin. It’s very tender and soft there, and it’s still a little unhealed, because an inflammatory process began from this nail, it was painful. Here. And they wrap this bandage around me and say, well, that’s it, now lie down and rest. Everything is fine. The next day it was necessary to do (..) soak the nail in a special solution so that it, the nail... The finger in the solution so that it heals. Here. And for this it was necessary to remove this bandage. I start to unwind it, but it won’t come off. He was completely stuck to this super soft, unhealed spot, it was just terrible. The time was probably just right... (..) Well, there was a day, I was sitting, I tried to soak this bandage in different solutions, a couple of hours passed, it didn’t help, we just started trying, well, tearing it off a little, that didn’t work either helped. It was terrible. We probably sat like that until very late at night, with the nurses, they tried to take it off for me, nothing worked, it hurts, it’s a super soft, tender place... In the end they told me, let’s do this to you. In general, well, it’s like a bandage, it’s even, applied to this finger, here. They cut off the entire part of the bandage and left only the piece that was glued to my finger. And in general, they tried to remove it with tweezers, pulling out each one of these... (laughter) A piece of lint from the bandage. It was terrible. It's just a nightmare. This also didn’t help, and I’m already in tears, of course, all upset, nothing is working out... I’m also a very suspicious person, (laughter) I’ve already thought of a bunch of things, that’s it, now he’s dried up for life, I’m like with him I’ll live, (laughter) upset, that’s it. Well, they told me to wait for the doctor in the morning. But that’s all that was left to do, and I ended up sleeping through the night with this bandage, and the next morning the surgeon came and in the operating room he made me some kind of, I don’t know, glued some kind of plate on me, and just tore it off with a very sharp movement. Yes, from this soft place, they simply tore off the completely dried bandage. It was very painful, oh well, and it was all bearable, it all went away, that’s it. I thought that was it, my problem would end there, thank God, everything was cured, everything is fine. So six months pass, the nail grows back, it grows very ugly, ugly, really, ugly, yellow, clumsy, all like this... I turn to the surgeon again, already here in Rostov, he says that Well, we need to do an operation and rip it out again. I think - what kind of person are you, really? Here. I’m thinking, well, are there any more humane methods of treatment, maybe it’s possible to even it out somehow? They tell me that such treatment is done only in private clinics, this is a paid treatment, but they said - try it, maybe it will suit you. And I thought that I didn’t want to suffer anymore, I wanted to know something specific from a specialist, some exact answer as to why this was happening, not just to rip it off every time, but to find out the reason. And what to do with it. And I turned to a podiatrist, in a private clinic. And now this doctor and I are still aligning my nail plate, everything is fine. Here. Of course, I was upset that I had experienced all this, but probably without this I would not have understood that it

is very important to go to, well, really good doctors and it is better to pay a little for your treatment, and be sure that they will really help you and correct you your problem, than every time, as they suggested to me, tearing off a nail. Here. M. What did you think, what did you learn... What do you think, what did this situation possibly teach you? Z. (..) Taught? Well, probably after all (..) until this moment I didn't really think about the qualifications of doctors, that is, you come to the hospital, doctors, they're all doctors, they probably know everything, smart people. And now, you look at it from a slightly different angle, well, at least I do. I pay attention to certificates, I pay attention to where I studied, what merits I have. Well, in general, for qualifications and I think this is still very important. And also thank you that we live in the modern world, we have the Internet, we have the opportunity to look at people's reviews, at a certain doctor, for example, or a clinic. And now, depending on the reviews, you can make a decision whether or not to go to this specialist. Here. Probably, after all, yes. The situation taught me to take health more seriously, this is not a joke, and to pay attention to who will treat you. This is also very important.
J. Because it's such a difficult topic, because I still... (..) Well, that is, I still live, for example, with my mom and dad, I communicate every day, I'm still building some kind of relationship with them. I myself go to a psychologist, somehow I'm already trying, from the side of some more... Well, not a teenager anymore, but some more adult person, I'm trying to look at any situations from both sides. Both from my position and from the position of my parents. Before that, at school, I always had some kind of very blaming position. That is, I thought that the way I think is exactly like that, and I never really wanted to consider any other points of view on this situation. Not how I see it from my side, but how other people could see it. Well, my parents. And that's why it's always been very difficult for me to understand them. And I was like that, a rebellious child inside, that is, on the outside I was quite calm, but I always disagreed with them, with something, I always somehow kept my opinion about you. And now I'm just trying to take it easier, it's easier to accept that someone may have a different opinion and that's normal. Other views on some situations. I don't know, I don't understand at all what choice means in terms of relationships with parents, but (..) in any case, it was always somehow very difficult for me, I... We always had good and bad, then good again. It was such a thorny road.
M. I think that this decision is also very difficult, and somehow very worthy. But you speak warmly about your father, and about your similarity with him, in principle, in part. J. Well, yes. Well, I went, as soon as I had the opportunity, I immediately went to a psychologist. And I still go to him. We have a free psychologist at our faculty. They did it for us. This turned out to be a very popular service. A lot of guys go. And this is really very cool. Yes, I'm working on my relationship with both my dad and my mom, how it all affected me, and where in this whole situation I am, and where are my parents' opinions about me, (npt).
Well, firstly, I didn't want to lie, I never had a need to lie or hide something, and (.) secondly, it's always obvious from me if I'm lying. Since I apparently didn't learn this, my mother always knew that something was wrong, even if I kept silent about something. She says - so, do you want to tell me something? She always (xxxx) saw and read me. Right always. Well, at the age of 12 I had a rebellion, at 13, maybe it lasted until I was 15, and then it started that I don't want to tell my mother this, she will scold me there or she forbade me to do this How can I tell her about this? After this, this situation, which lasted maybe a year or a year and a half, with that boy, no more situations like this were repeated, that is, this was enough for me, I completely lost all her trust, it didn't suit me, I stopped feeling this connection , so that I needed it, that is, she stopped believing me, stopped supporting me, it became hard for me. And this was not a reason for me to stop communicating with him, I just matured a little and realized that he really was something strange. What did I even find in him? (laughter) Here. And then I realized that I still regretted what I did. And from that moment on, I no longer deceive in any way. (.) And that is, my mother and I

discussed this, I say, like it happens, my girlfriends drink, and she says - okay, like if they drink, then wait a little, from 16 I will officially allow you, come on, (. .) whatever... (..) Only you will warn me, write and, as it were, you must come home, you don't have to go anywhere, and so on. Well, okay, that is, there were no secrets from my mother at all.
but initiative can be punished, because (.) I studied excellently, I had to, well, I wanted to participate in various competitions, Olympics and others, but I simply didn't have enough time in the day, I didn't have time to be good everywhere. And sometimes I felt bad because of this, I had periods, I don't know, now you can call it burnout, but I just felt bad, I was starting to get sick, that is, apparently this is how my body reacts, I don't pay attention to tired, but I pay attention when I start to get sick. And I just went into some kind of illness like the flu or something else, I might get dizzy, I would faint and bleed. These are the stories I have, but I just don't notice it. That is, this can affect your health, this initiative, because you are ready to be everywhere, but there is not enough time and energy.
M. But if we talk about, for example, a dance studio, a school and then a university, there, inside these institutions, they somehow tried to cultivate your independence? A. (.) At the dances... (.) We developed... We had such a strict teacher, I studied mostly classical dances, and training was constant, and there the teacher developed for us... (.) Probably character. That is... But I can say that it's probably... (.) Well, yes, on the one hand, I'm very disciplined, that is, in this regard, dancing gave me discipline just 10 out of 10, that's because there was such a teacher. And there was a certain atmosphere there, but (xx) it destroyed other traits of my character in me, that is, I could not develop as a person, because there, for example, there was criticism all the time, it was squeezing, that is, constant pressure. (.) So we also had a group, there were only girls, it's very difficult, especially when you're 14-15 years old, and everyone somehow had slightly different interests, well, in general... And I didn't feel like there in some cool company in which I feel good, so on the one hand I really loved it, but now I already understand, looking at this, that yes, the discipline turned out great for me, of course, but there are certain mental , I still have psychological traumas.
M. Yes, in general, undoubtedly. Are your relationships with your parents too distant, distant? Or somehow you quarrel, or... D. Sometimes they are very close, and most often these are just situations when we become close, which is extremely rare now, they make me feel guilty that I'm so bad and I offend them. But in general, well, I don't know, they certainly didn't do much harm to me, but purely morally, they did. That is, there is some kind of financial support at all. (..) But psychologically, of course, it's very (.) difficult. (..) Here. Varya, I'm a little busy. They are shorter in the kitchen. OK. In short... (..) So what should I tell you? M. I asked about relationships, about support or about conflicts, well, a little, in general terms, in order to somehow understand. D. My father drinks often, and as if before it didn't directly affect him very much, it was limited to the fact that we were walking along the streets, he was drunk, and just did all sorts of bullshit, I was ashamed of him. And then, in the ninth grade, when I began preparing for the Olympics, I studied them seriously for the first time, I almost didn't leave the room, because I wanted to seriously prepare. And my parents said that you sit so much and seem to be moving away from us, well, they fought because of this. And my dad also came home drunk, and my mother asked me to sit with him in the kitchen and talk every evening, he drove me to hysterics, and in ninth grade I can't even imagine how I pulled it out, but it was... Emotionally it was difficult . Here. And with my mother, well, I don't know, in general I've always been my father's daughter, well, I thought so, but for some reason I don't have a particularly good relationship with my dad now either. Mom sometimes, sometimes she somehow tries, like, when she sees that I'm really really bad, and that's not always the case. Here. But she doesn't take my problems seriously. Well, yeah, she doesn't take

my problems seriously. Yes, they don't take my problems seriously. That is, if I say - I'm tired, I worked, they're like - why are you tired, what problems can you have at 18 years old? Yes, it's true that there are none. Here. Well, in short, not being perceived as an adult also depresses me. Here. (...) So, it's very difficult now all at once, in fact, the relationship with parents is quite complicated, that's it. And now I've simply protected myself from them. When they are at home, I practically don't leave my room. Now my mother is offended by me, and I gave up, I don't care what she was offended by, I don't even want to find out, let her really be offended, if she doesn't want to enter into dialogue, it's her problem. Somewhere in the middle of the tenth grade, I tried with her, no, or at the beginning of the tenth grade, I tried to talk and say that I need support, at least just come and say that you're great, you can handle it, you'll succeed. And that's it, I don't need anything else. And this dialogue boiled down to the fact that I'm ungrateful, they spend a lot of money on me, they spend almost their last money on me, and I'm ungrateful. Then I got sick of them saying that they were spending a lot of money on me, so in April I went to work. That is, school and work were certainly difficult, but I managed. And since April, I haven't asked them for any money, I've bought clothes and something to eat here, well, I've gone to the neighboring town, all with my own money. And then they say something like this to me - why don't you go to the village with us, like you're at work again, you're cutting yourself off from us. Are we not giving you enough money? Something like that. Well, well, yes, I don't have enough money. So I went to work. Here. (..) In general, they are (..) children. M. Children? D. I can honestly say, they are not trying to change themselves, they are not trying to hear (..) me, that's it. It's quite difficult. M. Yes, I'm very sorry, what you're saying is very sad to listen to. And it's even so paradoxical, that is, well, it's clear that you're trying very hard and learning, and it's true, a lot of things are working out, and so, when they tell you that you're studying too much, and maybe you need to stop and go do something else, yes, I think it's very disappointing to hear. D. Yes, and when I wrote the Olympiad poorly and walked around upset, they told me that I just didn't study enough. Here. Something like this... M. Well, yes, in such conditions, of course, it is difficult to get support. Apparently we really need to look for it somewhere else. Please tell me, do you like being so independent, do you want to be more independent, or do you want to share this responsibility with someone? D. I began to notice that... Apparently I don't know at what level this works, but here... (..) somewhere inside I am still a child and in most situations, sometimes stressful, I begin to behave like a child. And then I, well, kind of think about it and... Well, I analyze it, and I understand that (.) did not behave like an adult in this situation. But it was necessary. Here. Well, in short, this independence from childhood, it actually affects me. Because even more so sports, they also tried like professional sports, all the work, and there, well (..) no study, no personal life, only sports. In short, this is it. You also need to be responsible. And now sometimes it takes its toll. Here.
M. That's why you chose a different trajectory now, right? D. Well, not because of this, in general I just have a lot of problems with my physical health, I realized that I definitely wouldn't go into professional sports, well, science was interesting to me, that's why too.
And on July 2, they left me alone. And in short, I went for a walk with my friend from the entrance. I really loved swinging on the horizontal bar, letting go and grabbing. In general, I once thought - like, how long, how long can I stay in the air without getting caught? Well, I fell on my face. And in general, it was a very cool day, so eventful, I rode in an ambulance, and then I probably walked around for a very long time with a blue face. Well, that's how my independent life began. (laughter) M. Yeah, what do you think this story says about you? How does this story characterize you? How do you think? How do you think about her and about you now? D. I don't know, it's just funny to me. M. But then it probably hurt a lot? D. No, by the way, I don't remember being in pain at all, I just remember that I fell and lay there for a while, then I thought that I needed to get up, and I felt discomfort in the area of my face, I wiped it and saw blood on my

hands. I think I'll go home and wash myself. And in our case, in short, then they were repairing the roof, and there were guys standing at the entrance, they saw me and they lifted me up to the fourth floor, called an ambulance, and called my mother. Now, if they weren't there, what would have happened? Or if these men turned out to be inadequate, what would have happened? Interest Ask. But everything turned out well actually, no concussions, nothing, I didn't break my nose. M. Yes, but what did the parents and grandmother say when they came home and found out all this? D. Well, grandma was at home, mom came running, scared of course. Some guys called her and said that your daughter is covered in blood, that's it. M. Nightmare. And on July 3rd they left you alone again? D. I don't remember anymore. Maybe. Mom works, dad works, and of course. Well, sometimes my grandmother went to the garden, probably... I don't remember. But I'm still living, so it's okay. (laughter)
And, by the way, I started going to doctors alone very early. Here. At first it was very scary, very unpleasant, we have a very inadequate woman at the site, a local pediatrician, and before she could bring me straight to tears, so. Now I just come with a straight face, immediately dissatisfied and angry, so that she feels my bad aura (laughter) and does not start putting pressure on me. (laughter) In short, I begin to put pressure on her first. Here. And everything seems to be fine. Of course, it's still sometimes scary to go to doctors, I don't really like hospitals, that's why. Well, yes. As I already said, sometimes I endure until the last minute and don't go when I need to, on time, that's it. This is of course a minus.
M. Did you end up in the hospital because of this? D. (..) No. But... Well, yes, I was at Sirius this summer, and in short I was slightly ill, I had a sore throat and a runny nose. Well, I didn't go right away, but I went to the first-aid post, they prescribed antibiotics, I took it for 4 days, that's all, I didn't go to the first-aid post and then say that everything was fine with me. I just didn't want to go there anymore, that's it. And in the end, after 2 days I felt very bad, I could not tilt my head, my sinuses hurt, everything was wrong, I was afraid that I had sinusitis. I had sinusitis, that's it. And I decided to go. They scolded me very much there, they said that this was impossible, they took me to the hospital, in the end they wanted to admit me, but everything worked out, and in the end I was on antibiotics for another two weeks. That is, half of the entire shift that I was there, I was on antibiotics. Now I have stomach problems because of this. And all because I didn't go and tell him again that everything didn't work out for me. Here.
M. This is the responsibility for the elections, right? D. Yes, yes. I am not responsible for my health at all. I don't know, I somehow feel very bad about my health. (npt)
M. Is someone else watching him, do they remind you? Or, well, just... D. Well, they remind me, but they don't make sure that I'm taken right by the hand to the hospital, because after all, I'm already 17 years old, I'm not a child. Here. And even more so, there was such a situation that since childhood I've been in hospitals, I don't know, I've had a lot of different things, I also have tuberculosis, some kind of problem, that is, that I have this mantu and Diaskintest are always big, something was stupidly infected there or something like that. She was registered. Then a cardiac surgeon, oh, a surgeon, (laughter) we also went to a cardiologist, I had problems when I started playing sports normally, in the fourth grade. Then, when I started playing sports even better, in the seventh grade, I walked like a disabled person because of my knees, and we also went to another city. Well, in short, a lot of things. And this is not a complete list. That's why I'm very tired of this, I don't really like going to the doctors and wouldn't like to at all, but somehow I wasn't particularly lucky with my health, to be honest.
M. I see. Are there some situations that you want to hide, on purpose and in general so that they don't find out and maybe no one finds out at all? D. (.) Probably self-harm. M. Do you do self-harm? D. (.) Not now, but through sports I can. Come to the gym and just... (..) To the point where

I'm just falling. Here. M. And does this help you in any way? D. Well, (.) not to think about something, I don't know. Just to finish myself off, probably. I don't know how to come home, go to bed and that's it. M. It's a pity, this is of course an inappropriate way, but as you say, it seems to me that you seem to have more different available tools in order to somehow deal with your condition. She showed me the book, told me about the exercises, and told me about her friends. D. Well, I'm trying, I'm trying to somehow get myself out of all this, in fact, I'm trying.
M. Can you think of, remember some example, from there, I don't know, from a movie, from a TV series, for example, I don't know, from another work, from a book, of a person who is independent and not independent? Maybe some celebrities, someone famous, or some bloggers, someone in plain sight? L. So, (...) I don't know directly whether this can be such an example, that is, interpret... (..) And in the work, well, Bukowski, there is Henry Chanaski, as his alter ego, the work " Women" is called, "Post Office" too, so, I remember the main character, in my opinion he is very infantile, he also lives the life that he wanted there, he is rather an example of this, he works there at the post office, with a huge number of sexual relations, beer, and it's as if he has nothing else in life. He didn't escape anywhere, and he didn't have to do anything. And if he's just so independent... (...) What's his name, I watched some interview today, I don't know, I remember, Mark Guarden, I think his name is. Something like that. March... Some psychologist, I listened to his podcast today. Some kind of person, I don't know... (...) I can give examples, so I look, I don't know, the rector at my university is an independent person, but I don't know (npt) work or not.
And after sixteen, yes, the first independent decision I made, I remember, it was on nerves, consciously, I don't know why, why, it didn't happen, I lit a cigarette. I came straight away, and I didn't have a situation where I was wandering around somewhere, in garages, I came openly and said, like this. Here. Naturally, my mother didn't like this, of course, but due to the traumatic experience there, she somehow turned a blind eye to it then. There she said - I'm sure it will fall off you, but now, okay. Well, we'll sort of assume that these aren't some kind of steam locomotives, it's not necessary. She is the only, it seems to me, the wisest thing that she told me, she says - please, you can, just don't buy some crap for yourself there, it's better to let them lie there at home, if you want, take it, like. Well, this is something normal. Because again, she says - what's the point, well, I would take it away from you, shout at you, so that you would stop smoking? Nothing would change. I would (npt) where is it, well, I would be hiding somewhere. No, and it fell off. I don't remember, I went there for two years, I worked through the traumatic experience with a psychologist, and one day I just said, no, I don't need this anymore. I wasn't so drawn to it when it was like this - well, you want it, right? Please. How (npt) is it normal, let's not just somewhere, over there at home on the balcony, you want, well, that's it, so that I can be calm.
M. That is, after you, I don't even know how to describe it, when you broke off relations with this man who showed domestic violence, with your stepfather, when he disappeared from the family, as I understand it, or he was imprisoned, I don't know. You had some kind of surge in all sorts of independent decisions and some kind of activity, right? L. Yes, well, yes, but rather it was probably, I don't even know how to classify it. Well, because yes, then it turns out that for 6 years you seem to live in a social cage anyway, there you don't think about how you can choose or think about something, but I don't know how it is conventionally, what jacket to wear today, so that there are no visible bruises. Anyway, this stupid childish trait - I won't show it, it lived, here. And this was the only thing in which you made (laughter) any independent decisions. Previously, you already go into some kind of, it seems to me, even aggression, in relation to those decisions in which you were limited, and you are already trying to grab everything that you couldn't take there before. Well, here I need to give credit, again, probably to my mother, but she somehow directed it all correctly, what are these, well, that is, my kind of eccentric, some kind of emotional outbursts, I don't know, or what - radical decisions, like, I don't know, smoking the same thing, were not met

with aggression on her part, which would make me even more willing to do something against. She manipulated it all so much that you were like, so what. And that's all there is to it, and that's okay. Yes, well, thank you, okay. Fine. Fine. Well, then, but I remember, yes, she gave... Well, the start date, oh, now, as if I didn't tear off my charger. I'll put it in. But before the start of, yes, my first year, it turns out that the first year and everything negative fell away from me.
M. Are there some things that you prefer to hide from your parents, maybe you don't tell them from everyone? You mentioned tattoos, but that was quite a long time ago. L. Yes, well, what can I say, now I wouldn't hide it, now I would come proudly (npt), to my mother and say - here it is, new. I just don't want to. I guess sometimes I can hide some of my emotional anxieties from my mother, because in general I'm such an emotional person, I can be sad, or just emotional... The only thing is, I try really hard, but I never succeed straight hide it from my mother, when I catch a cold there, or something like that, in short, it happens, I know that she will be nervous, and I don't like it so much, and I'm always trying to do something, somehow - yes, everything is fine , Fine. (laughter) These are things, yes, I try not to tell my parents, because they will worry, well, that is, I don't know, ordinary ARVI, but mom already - oh God, you live there alone, who will give you tea? do? Now, well, that is, these are not some kind of terrible things, I just (npt) don't want (npt) only dad. No one will lose anything from this, I just don't know, they will save 1000 nerve cells by doing this (npt). Without thinking about the tea I made for myself or not.

3.2 Table 2. Subjective health evaluations (good)

Citations
M. How do you like it, how, I don't know... M. Well, I decided for health reasons, I think it's necessary in principle, all my life I was an athlete, a tennis player, I tried a lot of sports after tennis, then I tried again, football, volleyball. .. M. As far as I remember, swimming, you said? M. Basketball, swimming, water polo, billiards, if you can call it a sport, I tried a lot of things, and when the time came for college, there was much less time for such activities, but I decided that at least just go to the gym there for I need some kind of general health, some kind of physical development, so I went to the gym, probably already about... (..) Well, I've been going for about a year now, (..) every week three times. I also made this decision quite easily, since I, in principle, connected my whole life with sports, some kind of unnecessary activity in my life, on the contrary, it was only a plus for me, my desire for this was there, therefore, too, the choice to go there was rude speaking, go to the gym, don't go, one, probably in terms of difficulty. Or something like that. Well, I probably can't say more about my health (..), well, I think there's nothing to highlight.
M. I hear some kind of conflict between what you think and how you act. And how is it generally given to you, is it easy to make a choice in such a situation or not? That is, why do the scales still start to tip in one direction every time? M. (sigh) It's hard to say. Probably because... (..) How to say... Well, again, probably there is a point in psychology that people often treat others the way they want to be treated. And among other things, I give people, other people, a chance because I would like to be given a chance one day if I stumbled. This is exactly why I give people there some kind of warmth and so on, that is, everything, all these moments, because I myself lack it. That is, what happens is, let's say, the opposite situation. That is, I give what I lack. Here. M. But this is generally interesting. How did you come to such an understanding, to such an experience? And to your own behavior? M. I unconsciously stumbled upon all sorts of psychological things a lot. (NPT) I unknowingly came across all sorts of psychological things, I don't know, because you scroll through your VK feed and there, for example, there are some posts about psychology,

something else, in the same TikTok you come across a lot of all sorts of psychological analyzes, oh behavioral types of a person and so on. So it all stuck to me like that and, in principle, some kind of awareness came. And that includes still coming. Here. And so... M. So, that is, you are not a naive respondent, right? And pumped up.
M. As if your experience of depression was for you also such a marker of where my friends are and what choices can I make in relation to them? And as if you, despite the fact that you are used to giving people a second chance, decided that you will not give them this chance? M. Well, I just, again, it seems to me that I have some kind of innate probably psychological things, well, that is, I can read people to some extent, I understand how they behave, how they will behave throughout to lead for a certain time, that is, it seems to me that I have some kind of psychological habits in terms of reading a person and so on. And all this. Understanding other people, including yourself. And based on this, I probably, well, probably don't give these people a chance. That is, I understand that they will not change.
M. Well, yes, it seems that you hear yourself very well and understand what you need and what your needs are. Tell me, with your activities, what you called a hobby, did you also listen to yourself or perhaps it was imposed by your parents? M. Regarding sports, I probably always liked to do something like that, to feel some kind of power over my body, that is, when you grow, including over yourself. Including remembering, as they say, (laughter) the classics of fight club, development through self-destruction, that's also a wonderful thing. This is also wrong, but I probably came to this too.
M. (laughter) Yes. Here. Therefore, I probably don't particularly value my health, and making decisions here is quite simple for me, I still try to control my health and so on, in the sense that, well, I try to see something in myself if something hurts me and so on, but otherwise, it's as if I've never had such a thing that I have to go straight to the doctor, that is, it seems to me that I'm more than healthy. I recently took tests, and they told me that they could at least launch me into space.
A. Yes, I work in an IT company, well, it's an ordinary development studio, I'm a backender. M. Tell me, do you have any hobbies, interests, besides study and work? A. Yes, my scientific interests are research in the field of education, I am mainly engaged in the history of the philosophy of university education. Psychology a little, I also do origami, sports are athletics, that's it. Just like that. How long have you been doing sports? Two years. Well, intermittently, I did a lot of sports as a child, well, I consciously came to this (npt).
Health - ten, it's very difficult to make a choice, I have some kind of masculine fear, I don't know, or in general it's a universal fear of going somewhere, or arranging an appointment with a doctor, going there, I don't know, to the dentist once every six months. Well, in general, it's just some ten, and I don't know what's stopping me.
Next, health. (..) Yeah, health, health. Here, you know, there is a rather difficult aspect, because for probably several years my health has fluctuated, (laughter) let's say, like the exchange rate of the ruble on the stock exchange. Probably until 9-10 grade, I was quite (.) a chubby boy. And I probably made the choice in favor of a healthy lifestyle on the basis of some, let's say, personal oppression and personal motivations. But for the most part this was due to teenage conflicts. And, accordingly... (..) Any problems. M. Did someone offend you? I didn't quite understand about oppression. A. Let's just say, (..) at some point it began to seem to me that at school, on the part of friends, on the part of teachers, at some sharp moment the attitude towards me changed. That is, they stopped noticing me and (.) paying any attention to me. Although I didn't seem to do anything wrong. Nevertheless, I didn't really understand what happened, but at that moment it was probably quite difficult for me to communicate with people, because I didn't understand what this attitude was connected with. That is, if I had a teacher with whom I was in close contact, and (.) with whom, in principle, I was conducting some kind of project activity, then at that moment it

happened, I don't know how some day came and as if at the snap of a finger What happened was that this person's entire attention switched to another audience. And essentially, you know, it's like a child who has been sharply deprived of attention. You want it, but they don't give it to you. And I probably began at that moment to look for disadvantages in myself and in my appearance, in my health. I began to think that this was probably connected with some characteristics of my body or characteristics of my thoughts. And then I probably started soul-searching. (.) Therefore, the choice in favor of health, it was probably given to me, let's say, through a battle, due to some experiences, through an attempt to prove to myself and many others that everything could be different, that I can do something... something else. Therefore, in terms of severity, probably (..) well, let it be a ten. At work... M. So you connected this deterioration in your relationship with the fact that you somehow gained weight, or somehow didn't look the way it seemed right, right? Because you were talking about the love for sweets, and about being chubby, you said? Or did I misunderstand you? A. Well, for the most part, I've been pretty chubby since childhood, and I probably stayed that way until the 9th grade. Therefore, purely physically, I didn't change much, due to weight gain or loss. There was simply, let's say, one consistency of the body, which was maintained as it grew. Therefore, I don't know, I probably connected this not with an increase or change in weight, but with the fact that at some point some skills and shortcomings that people did not want to take into account simply became more noticeable. M. Did you say something to you, did you somehow become yourself, did you say about choosing a healthy lifestyle? Something like this. A. Yes, I decided that... The Unified State Exam had just ended... the Unified State Exam, in the 9th grade, I thought that since such a situation was happening, I needed to rethink myself, I needed to somehow start taking care of myself, to show myself, well, to change something in myself, not only mentally, but also physically, so I probably made the choice in favor of a healthy lifestyle, in terms of athletics. That is, I got up at about 5 in the morning, slammed the doors, everyone was scared of what was happening so early in the morning, and I just left the house and started running at the stadium, ran to some park and spent my time there.
I really don't like this, so I'm trying to gradually return to my usual way of life. And somehow connect sports to your activities. But for the most part, probably due to my (..) fear of people (xxx), some kind of tightness, I don't always succeed. That is, I cannot, relatively speaking, sit in a hostel, do something, get up and start doing push-ups there. Or, I don't know, stand in the plank. Because, (.) you know, this is an internal experience when you look stupid in someone's eyes...
M. Well, yes, I understand. A. Yes, for the most part that's why I got up early in the morning and ran away earlier, to get away from prying eyes. M. Well, yes, very clear. A. Now, well, basically, what you can do is go out late in the evening, we have a small park, a student park, and do a few laps of running there if you have free time. If not, then I try to diversify my diet and make it, let's say, less high in calories.
T. And I remember myself probably from the age of one... Well, probably from the age of six, from five, somewhere like that, directly consciously, so to speak. Well, as a child, I initially wanted to become... (..) To become a policeman, that is, at that time, a policeman, and, in principle, my parents did the same, if we talk about independence, that is, they always signed me up somewhere, to some clubs, some sections, that is, since childhood I was kind of fond of sports, that is, I was such an activist, that is, both at school and in some extracurricular activities, social events, that is, always for the most part, they attracted me, too, and (..) it seems so, and some leadership qualities were probably emerging, so this is where the roots probably go, maybe some of my independence in making some decisions. That is, as I have already said, the sphere, so to speak, of my activity is quite wide, that is, starting from the public... That is, this has been going on since childhood, that is, now I, let's say at work, am the chairman of the council of young scientists specialists, these

leadership qualities that have been going on since childhood also manifested themselves. And I finished school, that is, until the eleventh grade, passed the exam, and entered the university. At the university, of course, it's also a little less, since after school, for some reason, I began to devote more... More time to studying, not in the periphery, but specifically to studying, and therefore, a little less, I had classes that were, so to speak, excellent from studies. But still, no matter how I gave it up, I also took part in all sporting events and social events. So I studied and studied, and naturally, after that, after that, I went to work. And at work, from the first, as they say, days, that is, he showed himself as a fairly active person, went out with his colleagues somewhere, that is, he was always, so to speak, easy-going. And, that is, after that the management noticed and, as they say, I am still, so to speak, in plain sight. That is, just like the leadership, as it was before, like at the university, like at school, and now, let's say, the strategy can be action, that is, just independence, leadership qualities, and so on, then are present at the moment. Here. In principle, I think, more or less answered. (laughter)
T. Well, I have this character trait: sometimes I follow the majority. That is, in order, as they say, to go against the flow, namely, my closest circle. That is, my environment was quite like this, just sports, that is, we were engaged in dancing, and naturally this was vocals. I was, I looked at other people, I looked at how they were, whether they were achieving success in this regard, and then I tried it on myself, whether I was even interested in the same activities that were popular at that time, and I already made a decision. And if, so to speak, in percentage terms, they probably have their own opinion and people's opinion, well, in general, the opinion of the current, the opinion of society, so to speak, is probably about 50/50. Well, maybe 60/40, in my favor. In this plan.
M. In general, well, now it turns out that you are discussing with someone some things that may be difficult for you, well, be it some psychologist, I don't know, a close person? T. Well, yes, there were moments, that is, when I visited a psychologist, that is, in principle, there were moments when I was just put in a leadership position, and I, having worked for only 2 years, yes, about two years , one and a half, in general at work, that is, I was put in a leadership position, naturally, there was stress. In general, there was a lot of stress and I needed some kind of psychological help, (..) it turns out to be qualified. Yes, I also consulted a psychologist. And in principle, this gave its micro-fruits, that is, well, perhaps, including the fact that I have already gotten used to it, that is, this so-called adaptation period has passed, and now I am more or less in a normal, so to speak, psychological rut and everything is fine.
T. Well, in principle, all my successful decisions, which were made at the junctions of the most important stages in my life, were at school, that is, the choice, I don't know, not just there, I don't know, walking around the entrances, smoking and so on, have a drink there, namely, go in for sports, that is, some kind of social, some kind of personal development. At the university, that is, to enter exactly the university that you are now, well, in which you naturally studied, (..) it is precisely the choice of the field that is most promising in our country. And at work. That is, I work in a fairly large company and this, in principle, makes me happy. I don't think that somewhere in these probably most important stages for myself, in my life, I made some wrong decisions.
T. Well, yes, that is, before, I'll interrupt, I'm sorry, I just had a problem before in that sense, that I, well, when you see a cat trying to attack a mouse, that is, it first prepares itself there, sits for a long time, waits, and at some point pounces. I've always had this in anticipation and preparation, just before communicating with a psychologist and so on, that is, I've always had this for a very long time, that is, I'm trying to calculate every little thing that is impossible to calculate, and sometimes just you miss your goals. This is exactly what is needed, it seems to me, we simply mean to wait, but not to wait out in general, roughly speaking. That is, to evaluate yes, more or less seriously, (npt) you act as you see fit. Here. Something like this.

I. So health... What elections, elections, elections, elections, elections, well, it seems to me that with my health pah-pah-pah, everything is fine, I try not to get sick, (laughter) here. It seems to me, well, if we take health in general, well, my mother just had a stroke there. I had to make certain choices there, I don't know, about apartments, about inheritance, and so on and so forth. This all also falls on me, in any case, so for me, well, it was hard for me, it's still hard for me to resolve all these issues, because in many areas, with many of these things, I'm meeting for the first time, there the insurance company, the hospital, I don't know, they have to process certain documents, it's incredibly difficult for me. As a child, my health was bad. As a child, after childhood bronchitis, I had very severe bronchial asthma, I was sick for a very long time. I rarely, I didn't go to kindergarten at all, and I went to school, but I was sick very often, of course. (..) It was very difficult for me as a child, so it seems to me that I would rate my health as exactly this, I would rate it as an eight
Well, here's an example. That is, well, really, communicate with people. Well, I'm proud that I was able to defend this Olympics, the championship, I'm proud that I have some skills, I'm proud that I found an internship, I found it myself, yes, I'm proud of that. I'm proud that I was able to achieve certain things in sports, just when I wanted to do karate myself. I'm proud of this too. That is, here.
Well, this is what happens in life, in general, it always happens, for example, if there was a need to get answers to some questions and close your own questions, whether for business or personal reasons, I found a good psychologist, went to a psychologist, solved the questions, closed questions. Somehow like this. Should I answer in this format? Is that normal? Is it clear now?
A. For health reasons. Well, in fact, in terms of health, what can you say, what can you say in terms of your health... Well, I've had problems with health for quite a long time, it was decided that health needs to be dealt with systematically, and the decision was precisely on the advice of on the advice of parents. I adhere to the following approach - once a year you need to do a complete Check-Up of your health, and regularly take tests and monitor them. And this decision was made very simply, because there was advice from my parents, accordingly, after the advice was received, I understood why this was needed, why you need to regularly track the indicators of your body, I realized how important the energy that you have is, to exist well, and there would be enough of it for what you do, what you do. Well, that's why I regularly take care of my health, track and live happily and energetically.
M. I think that you not only check, but you said about surfing and probably, well, you look very athletic from here, at least it seems, I think that in addition to checking, you also do things, well, you said, go to the gym, right? To the hall. A. Well, yes, because I realized quite early that sport is energy, well, more precisely, at least for me, that's it. And in general, I just can't live without it, because... Well, no matter what your condition is, if you go to a training session, or go surfing, or go jogging, then you get a huge boost of energy, and in general I feed on this. Well, that is, sport is one of the key ways to get energy, to recharge in order to do those energy-consuming things that we do either in business or in other aspects of life. Here.
M. What brings such warmth to memories of school? In primary school? M. There was somehow more friendly communication there, with the team, but the only thing was that I was very far behind in sports. When they played football, volleyball and so on, I usually sat on the bench. M. Did you want to continue, did you want to play together? Or were there some reasons why you didn't play? M. To be honest, it's difficult to answer, because on the one hand, it seems like you want it, but you don't seem to want it, this. M. Was it your choice to sit and not play? M. (..) No, I think my lack of skills played (..) in this.
M. And what will it be? M. I want to go to boxing. It's very necessary, in fact. M. Why boxing? M. Well, because it is he who has a great psychological influence on people. Well, according to my

observations. This is both confidence and the ability to stand up for yourself, which is very important to me.
And I started, that's how I played computer games for 16 hours, in the same way, for 16 hours, I read literature and classics of literature. And of course I tried to write poetry. Here. And then, in all the blogs that I kept on games, I began to write about the fact that games are, of course, cool, (npt) but it is necessary, well, the trick is that they greatly, they very much limit the space in which you're cool. And I really love being cool, still do. Here. And how could I, of course, be the top one in the game, but if I go for a walk around Moscow, it will give me little. Here. I wanted to develop something with more generally accepted values, in some more generally accepted values. Well, that is, for example, knowledge of foreign languages, erudition, career prospects, and so on. So, at the age of 16, at 16, yes, at 16 and a half, in short, somewhere there, at the end of 11th grade, I realized this and began to work in this direction.
V. Yes. Yes, I wouldn't even call it stress. I kind of liked this state of competition again, another one, here. Well, so did the entrance exams, and I was like, oh, I entered Moscow State University without a basic philosophical education, that's fine, now I'm also a translator. In general, this energizes me. So, in short, I always loved this state of competition, and as a child, when you are not competing in the professional market, this is probably one of the only niches where you can compete, these are games, well, such games, board, computer, or sports games. Here, by the way, I would actually dwell here in a little more detail, because I love, since I love this whole business, I did both, and the third, that is, I always played board games with pleasure, a la cards and sports games. I say, I played chess as a child and table tennis. Here. But... But just at the age of about 12 years old, that is, at 11, I started playing computer games, and at the age of 12, I left sports and stayed only in computer games. Here. I wonder why this happened, well, I think I don't know. (laughter)
In general, in principle, I don't like, I don't think that anyone knows better than me, especially in pedagogy, in matters of education, because I have a psychologist's education, pedagogical, I know perfectly well that no one knows my educational does not diagnose needs. Moreover, no one has as much knowledge as I do about which method of presenting material is suitable for me, so this education system does not give me a choice, well, let's say this happens often, the curriculum says that there are many disciplines by choice, but in fact there is nothing there anywhere, plus the budget needs to be distributed correctly, in the end there is one, well, at most two, you have a choice. In general, I don't like this all the time, I like it when I build this educational route for myself. M. Well, that is, studying at the psychology department helped you understand yourself? Q. I don't think it was specifically my training at the psychology department, although... I think that it structured my thinking, of course, certainly my thinking and worldview. In principle, psychology, pedagogy, and philosophy, which I studied, help me understand myself better.
V. Yes, well, accordingly, I created my own group, and spent the whole last year working with this group. And now I want to try again, now I really don't know, but I still hope to somehow go to study in France, so here I have the same problem, that I'm not a psychologist, that I'm a psychologist-educator, and France is more strict system in terms of changing qualifications, so I need a year at a Russian university. Here. Therefore, I want to study at a Russian university for a year at the Russian State University for the Humanities, and then try to transfer to... Well, there is the city of Caen and there is training for translators in three languages - Russian, English, French, and so on.
Well, a good choice, a good choice, I would probably attribute to my first university, that is, psychological and pedagogical education. And again, this is one of those choices that I made, guided by one criteria, and with the hope of one result, but it turned out to be good, that is, the results turned out to be different, but better. Than I could have imagined. That is, I chose a

psychological-pedagogical course, because I knew biology, chemistry, mathematics, Russian, with these subjects I could enroll either in some kind of biological-chemical thing, like there you could go to Timiryazevka to study genetics, or to honey didn't pass. Well, in short, there was an option, either to go for something like that, biological, I was basically interested in genetics, at school I loved problems on genetics, but on the other hand, the option was to go to a psychologist, a psychologist-teacher, that’s it. And, in fact, I thought, damn, now I’ll go see this geneticist. Although I like solving these problems, is it that I will spend my whole life developing new plant varieties, animal breeds and stamps, strains of microorganisms? This is like the definition of genetics, actually. (npt) I think, God forbid, this is boring. I want to communicate with people. And I went to become a psychologist-educator. And why is this still a good choice, because I really liked my university, the Moscow State Pedagogical University, it’s not very overloading, I can, as a person who has already studied at a bunch of universities, and who has many friends from different universities, I know that it is so, moderately stressful. And there was a little more tension in the first year, and then just not at all, not very much. And I really liked it, because I think that if I had studied at another university, I would not have been able to simultaneously learn French, for example, to a high level, because I would have to complete some endless tasks there. And I would not have been able to gain work experience either at Artek or at Detsky Mir, or as a chess teacher, I was still working. In general, I would not have gained much work experience. I would describe the choice of university as successful, but now let’s talk about your criteria. So, when this happened - in 2015, who was among the participants in this situation? Well, I can say that naturally I was the main actor, but here my parents were naturally involved, like the parents of many children after the 11th grade, so. They gave me some advice there. I probably made this choice at the dacha. I was just wondering where, well, I was looking. I sat with the lists and in the end decided that, well, I went to different ones, and decided that psychological and pedagogical education, it still sounds universal, like a psychologist and a teacher. Of course, this is actually a trap, if anything, it’s a trap. In fact, you won’t be able to work as both a teacher and a psychologist (laughter) because you don’t have a subject to teach. (laughter) And the psychologist is also only a school psychologist. Here. (laughter) But I didn’t know that then.
About training, professional development - I decided to engage in self-development in the field of programming, I selected courses for myself that, in an amicable way, I would need to take. Because, in fact, I personally plan to work in the field of (NPT). Well, that's probably all. Health, I’m in... Six months ago, when my dark streak of procrastination ended, I started playing sports again, going to the gym. And during that period, I tried to arrange healthy meals for myself in the hostel. I stopped subsisting on some fast food and other semi-finished products and started cooking for myself. choose a diet.
I try to be kinder to myself and to others. Excessive self-flagellation and excess severity towards your friends, it rarely bears fruit, I try to refuse it. (...)
Okay, I'll try now. Training, professional development, probably six, making a choice may not be that hard, easy to say (npt) here to understand. But it is difficult to implement this choice. Health, probably three, but in fact, the gym and healthy eating take away almost everything from me...
N. (sob) Thank you, it’s very nice to hear all this. It’s just that now it’s actually much easier for me to tell all this, because it’s all already agreed upon with my loved ones, and well, my friends, my former partner, or my current partner. And to the psychotherapist, here. That is, like another year or two after what happened, of course it would have been more difficult for me to tell all this, but now it’s like... Well, you just understand that... Well, somehow in general, it’s all rational, meaningful and... But thank you very much for the words of support, it’s very (..) nice every time, me too...

M. I really like the way you praise yourself, it’s really great that you acknowledge your victories, understand and build it into your story, you understand that yes, I really did it right, and now I get what I wanted, and even unsuccessful elections in the past become not so unsuccessful; on the contrary, I am very glad that I made a different decision then. N. Well, I think that this is all kind of connected, plus or minus, with the fact that I turned eighteen, I began, plus or minus, to somehow adequately perceive myself and the world, and then again, now it’s like I’ve added to all this psychotherapy, and but I just, as if my emotional intelligence is growing a little, you can, well, somehow listen to what you feel and already determine what you feel, and not just feel and be like - oh, I feel bad, (laughter) here.
As for health, well, as such, well, firstly, I chose to do a health check-up in order to identify some... Some areas in which there are some problems, this was already a big step, because I couldn’t get to a regular doctor. And so I chose not to put off some moments. Of course, now, with a new workload, it’s been postponed for some time, but I think I’ll get back to it soon. (..) And I will start again the procedure that I was doing. M. And what about regular health checks? How regularly will this happen? A. Regularly - this is six months. (..) And about work, work... M. Sorry, please, can I ask you more, about your decision about regular health checks? A. Yes, of course. M. How did you decide to do this? How did you make this decision? A. (..) Well, since we now had Covid, I... And I never had confirmed Covid, I decided to go and find out if everything was fine with my lungs and everything else, and since now there are pavilions in Moscow, health pavilions, and there is one of them literally next to my house, I thought that it would take an hour, an hour at most, and I have an hour of free time, I can go there and do it. And also, well, when... (..) When you underwent medical examination at work, mandatory, it’s also like a choice that we are not given, (..) what you are obliged to do, and also this, well, some your own feelings that something is wrong and you should go to the doctor, and he will refer you where you need to, according to your needs.
M. Please tell us in more detail how it happened that you wanted to start volunteering? A. I accidentally saw an announcement about holding a marathon, in general, in my city, I had to run it, but there was some kind of large fee, because, well, I used to do athletics, and I thought that I could run, but there there was some kind of large contribution, and I thought that well, I don’t really want to ask my parents for money, and I saw that volunteers were needed there, and I simply wrote to the post office. And after 2 months they answered me, here, come. And then again, again, again, again, and so on, now several projects a month. (laughter) Or maybe even a week, and in general, now no longer as a volunteer, and not only there on all-Russian, but also on international projects. Even now, no longer as a volunteer, but as a manager of volunteer projects. M. Great. Is this related to sports, are these different projects, or is it one direction? A. We did this with sports, but now sports are a big, well, sports projects, they are a big part of my life. And the rest of the projects are of different directions, just the very fact of volunteering, the very fact of helping, it remains unchanged. M. Can you tell me what feelings the situation caused you to decide to volunteer? A. Then it was very interesting to me, because this is new communication, this is a new community, these are new opportunities, because I really missed communication exactly in adolescence, then it caused a feeling of such, perhaps instant admiration, that is happiness is in the moment, now this also happens from time to time, precisely at some event, but you just realize that you have something to do, and you can go to any event and go help someone. M. How do you feel about this now? A. Now I’m completely delighted, because you live this, you love doing this, when you’re at some event that you really like, you just almost cry with happiness that you’re there.
And probably one of the things that helps me very, very much is that I have been involved in sports since childhood, at first I went to a circus studio, probably from the age of 4 to 9, I really liked it. And I liked the fact that there is freedom of creativity, and there seems to be some kind of framework, in which you have a coach, you have your leader, and you, for example, put on a

number with him, that’s it. We also traveled a lot and participated in some international events, and I probably even received my first salary, well, as I believe, I received it abroad. And it was interesting, because these (.) were moments that relieved the fears of the unknown, because for example, we performed in front of a huge audience, there on the streets and did some kind of performances, despite the fact that we were all small, we in fact, they were very shy, that’s it. We somehow overcame ourselves, and it was somehow unobtrusive or something, because we perceived it all as a game. And now I understand that I can also use this approach in some life situations.

And I kept asking, asking my parents to bring me to the gymnastics group, and then I still don’t know, maybe my persuasion somehow influenced my family, maybe something else, that’s it, but I still They brought me in and I started playing sports. And I understand that it’s probably sport, it has played a lot in my life and in my character, too, a lot from sport. I have there, regarding the achievement of any goals that I set for myself, that I can gather there, even if I understand that it’s hard for me, bad, or something else, I understand what I have after all, there’s this kind of core that probably doesn’t allow you to give up when you just need to pull yourself together and do something, come up with something, that’s it. Or somehow cope with the situation that exists. Here. And probably the fact that in my family, in general, (..) there is such a moment of complete freedom. That is, I chose, after school I entered a technical school, because I did not enter the university, and my parents said that you choose a profession and direction yourself, because we want you to be a good person. And of course, perhaps, I think that this was bad advice, because when you are 16 years old, and you want to get specific, well, some steps, how to choose, what to do, maybe how to look at the test results, according to career guidance, which shows you 16 areas of activity, and you don’t understand what to choose. Here. But it seems to me that this is my path in education, it seemed to be a little in different directions, but it seemed like it was almost cultural, almost literary, probably. Therefore, somehow everything comes together, as if like a puzzle, here you are, seemingly from different places, but you find puzzles that your picture is missing. It's probably something like this.

P. Health, oh, I had to choose several clinics, here. And I also read there, looked at it, asked some people who live there for some advice, what’s what, what’s best to choose, that’s it. And that’s probably all there was in terms of health. M. But where do you usually get advice regarding clinics there, and the health sector in general? P. Here, as advisers, I probably have experienced guys from the dormitory where I lived, because (..) they, well, I asked the guys from the second year, and I asked, because we had several clinics around the dormitory, which is better, is the university clinic better, and so on. Here. Plus, I looked at the reviews, and looked at more reviews on the main website of the clinics. Here. Well, I chose and I'm happy with the choice. Because everything somehow turned out very cool there. Well, just one piece of advice that I was hooked on, I read it, and I realized that this is a great clinic, and now I’m really happy

stopped playing sports at the age of 16, and probably now, after a certain number of years, I would tell myself that I still need to continue to exercise, even a little, even a little bit at a time, maybe a couple of times a week , but still do it. Because at that moment I had completely finished studying, that is, I had some activities, not much at all, but still, here you go. But I wouldn’t want to quit. This is probably the choice I regret. M. But if you go deeper, when exactly, how old were you when this happened? P. I was 16 years old, it turns out that I graduated from school, began studying at a technical school, and I simply did not study in the area in which I live, and not in the area, and not even close to the area in which I played sports. And plus it so happened that at that time the group in which I was studying was disbanded, and then I thought that, well, the coach and I also discussed that it would probably be better for me to look at some university or technical

school somewhere. activity, because I trained with girls who are younger than me and from a sports point of view, this is kind of a step back. And significant, because if you train with those who are on the same level as you, or a level or two higher, that’s good. And when you train with those who are kind of far behind you, it’s not good. Because you, too, will roll back to approximately their level. Here. And then, yes, at that moment I thought that yes, it would probably be difficult to travel, because school would end anyway, it would be very late and there was still a road to class, and classes too, and it would be difficult. Here. Well, then I bought a membership, a membership to the fitness room, that’s it. But it was also difficult with him, because he was also not so close, but still. Here. And I went and worked out, but I understood that this was not enough for me, and I just wanted gymnastic activity at that moment. M. But you said that at that moment, well, as an adviser, well, the coach was present in this matter, and who else? Who else was involved in this situation? P. No, just me, me and the coach. So, it turns out, I told her (..) my situation, and so, well, she suggested this option. Of course, she wouldn’t have refused me if I had said that I would like to continue, so. But she outlined the situation to me, as she sees it, as a professional, as a coach. M. But if, in contrast to unsuccessful ones, we talk about successful elections, what elections do you think were successful in your life? P. I would probably highlight the choice of sports, gymnastics, as especially successful. I understand that it's mine, and I understand that it was very important and great that I liked it. And I’m probably lucky in that this sport is interesting not only to me, but also to my family, and my family really supported me in this, then the choice... (..)
M. Tell me, how did the desire to go to study journalism come about? Why did you decide? L. Well, in general, until the seventh grade I wanted to be a psychologist, so. But... M. How interesting. L. Yes, my dad is just a psychologist, that’s it. But in short, there was such a story that I began to find out how to enter the psychology department, and found out that I had to take biology there. M. Biology, yes. L. And biology was generally my favorite subject at school, and I say - no, I’ll look for biology (npt) for something else. And so I started buying all sorts of magazines there, these ones, for girls, (npt) then later, I became interested in all kinds of fashion journalism, and then it means, I don’t know, there in the eighth grade, in the ninth, I became more interested in politics, I wanted to be a journalist who travels to all sorts of hot spots, that is, something like that, little by little it became more and more boring, then I just wanted to be some kind of observer, that’s it. Well, that is, it was just some kind of interest of mine, based on some of my predispositions. That is, I was always good at writing texts, in principle, my language was so good, well, I understood that this is what I am passionate about. Here. M. That is, but it was as if it was a choice from the opposite, that if not a psychologist, then I would be a journalist. L. Well, I wouldn’t say that this is probably a choice from the contrary, that is, well, my interest in psychology never went away there, my dad always gave me some books to read, he conducted all sorts of tests with me, that’s it. It’s just, well, maybe I’m also partly burned out, because, well, I’m basically the kind of person who, if I really want something, I’ll hurt myself, but I’ll achieve it. Well, that means it wasn’t such a really great goal, because if this goal had been great, well, I would have learned biology. Well, what can you do? (laughter) M. Why did you want to become a psychologist? Because dad was a psychologist or is it somehow different? L. No, I actually found out that my dad is a psychologist after I decided to become one. But this, you know, is such a story that you never know what your parents do, and then you find out. Here. Well, in my opinion, it all started because of the series “Lie to Me”, I was very interested in this topic there, like body language here (npt) that’s all, it was so cool, then I read that There are sports psychologists, I thought that was also interesting. I wanted to be a sports psychologist. And then I began to get interested in serial killers, just reading their biographies, and I wanted to be a psychologist who works with all sorts of criminals, that is, he understands why they commit such terrible things.

Well, maybe I also watched enough of some TV series there, like “The Crypt”, where I remember going to the dacha, watching with my grandmothers in the evenings, where a criminal is also sitting there, and a psychologist is working with him. Here. Well, somehow it attracted me because it was such a very socially significant profession, which, in general, was aimed at a good cause. M. At what age did you decide to become a psychologist? L. Oh, well, it seems to me when I was maybe 10-11 years old, that is, before that I wanted to be an actress, well, let’s say, many girls in elementary school want to, and then I think - no, it’s too fickle, a psychologist - that’s the most That.
M. It turns out that the Department of Psychology helped me to be more independent, if I heard correctly? N. Yes, it seems to me that yes, and it seems to me that it’s just plus the people there, my friends and my husband, it’s like, that is, we somehow switched to a more healthy (npt), healthier kind of then the atmosphere, somewhere there it is normal to experience some emotions, well, emotions such as anger or hatred, or... Well, in short, that is, which previously seemed to me that they should not be experienced, or that it is bad to experience them, or something else. Well, in short, there is no plus about family (npt) there (npt), that these are all sorts of non-adaptive patterns and so on and so forth, but how very upset I would have been at first when I took this subject, that this is how bad everything is and all that, but then I thought that, in general, this happens not only in my family, it happens even much worse than in my family, for example, and that parents, they just seem to try as hard as they can, that is, they seem to they also draw conclusions for themselves, based on those who raised them, based on life experience, and what they learned is what they passed on to us, so, and we can then also take it out and pass it on, and so on it will always be like this, and therefore it seems to me that this is, in general, a positive dynamic, that it is, in principle, enough, and not what you say there - this is bad, this is not the same, this is not adaptive, this is something else... That.
N. I would rate it a 10, to be honest, because my parents, my dad, were very much against me going to the psychology department, he wanted me to go to law school, I have a mother, her first education was in medicine, then she studied in psychology. went, but she was like, well, it’s like I want to be like her, she thought, here. But I didn’t go there because of her, I was just interested, as if about the people, there, like with the people, what’s in their heads, how to understand it all, that is, it was somehow for me it was interesting about behavior and emotions, so I kind of... And so my mother says something to me: go to these courses, go to these courses, there’s something else, and I say no, I won’t go, I’ll decide for myself what courses I should take, or my dad says, I need to go to a master’s program somewhere to become a lawyer, or something like that, and I say like, no, I don’t want to, or whatever Here I am this year, well, in the next academic year, for example, I won’t study, because, well, like my parents, they would rather have me go to a master’s program, but I just understand that I’m tired of studying (laughter) that I can’t, (laughter) I need rest. (laughter) And that I just don’t understand where, what kind of master’s program I would like to go to, I didn’t understand, and therefore I didn’t go anywhere, and how would I make this decision for myself, how would I want to work and understand maybe there's a better place for me to go. About the job, I would probably rate it a seven, because my first job was in my dad’s sales office, that is, my dad, he owns a construction company, and I worked in his sales office. But I wanted to work myself, as if I wanted it myself. And I kind of came there every day, stayed there for some time, they already told me like - go home, but I was like - no, I still need to do this, and I tried to come up with something there, whatever didn't come up with it. Here.
M. Do you have any hobbies? Besides work. A. Well, probably yes, part of it is the field of education, psychology, everything connected with tutoring, with... (...) Drawing, exhibitions, studying, well, I don’t know, the work of artists, this is also connected with literature, Let’s say

now, the main focus of interests is the book (npt), Annie Lee Lang, which describes art, artists, and, in principle, the concept of loneliness. And how artists reflected it, how it is depicted in culture, in what images. Here. Something like that. M. Great.

Another important choice, which in general, if in the future 10 years, then (..) it's probably closer to the family. Well, that is, as it were, a choice in favor of (..) building long-term relationships, and another related choice - health. Well, that is, as if I don't want to waste what I already have, because our body is only depleted, but I want to somehow fill it up, maintain balance, different areas of life that affect health, ultimately well-being, and the feeling of happiness when you feel good. This is a choice in favor of, well, taking care of yourself in different areas. Well, that is, starting there, I don't know, with some kind of tests, check-ups, regular support for physical fitness, psychological health, such a minimum of some kind of psychological hygiene, then yes, this is directly connected for me with family relationships, because in Ultimately, if you make a choice, then for now I have the feeling that I am not childfree, but I still want some kind of (.) continuation of the family and continuation of the clan, if you can call it that. Again, I want healthy children, a healthy child, and this means that I must be healthy. And the other person must be healthy. Therefore, this is a long-term construction, with certain resulting requirements for a person. (..) And probably not by requirements at all, but by similarity of beliefs. When they look in one direction. Well, probably for 10 years, well, another important one, this is first, in the short term, somewhere up to 5 years, a period of time to take, then this is a set of expertise and experience, packaging it and going out not to work for someone, but for myself.

M. You can go straight through the list, you don't have to arrange them. K. Training and professional development is... Probably there will be seven, because (..) you always have to learn everything, and sometimes it's very difficult to allocate your resources and brains, (laughter) let's put it this way. And do one thing. Health is five, (laughter) it's either there or it's not, work is also six or seven, because... M. Wait, let's go back to health, making specific decisions. In terms of health. K. Regarding my health, everything seems to be fine, I feel good, but sometimes there are times when I get sick, (laughter) Covid and so on. And I try to recover as much as possible. But if I get sick, then I don't do anything, it's immediately minus work, (laughter) minus friendships, only the place of residence will be there then. (laughter) At maximum. M. What about choices, such as choosing which doctor you need to go to, or even understanding whether you need to seek help or not? K. Choosing a doctor, I don't know. I somehow... M. How difficult or easy is it for you? K. No, it's easy for me to choose a doctor. I just see, either from reviews, or using word of mouth, that this doctor, we went to see him, he's good. I'm so good, I'll go to him too and everything will be fine. In principle, this is how it turns out, so I don't have such a difficult choice here to choose a specific doctor. Well, usually, I just probably didn't get sick with anything serious, no matter where it depended on me... The doctor's choice is between life and death. Therefore... M. In general, you said that you rate your health as a five, then what is the difficulty in making decisions regarding health? K. Health, difficulty making decisions, (laughter) giving injections. This is where it's difficult. (laughter) Or force yourself to go... It's just that when you're sick, you don't really want to go out. M. So it's difficult for you to understand whether you need help or not? K. Yes, I can't, it's sometimes difficult to assess the extent of my illness, it just seems to me that even with a temperature of 38 you can still do something there and at work, everything is a doctor (NPT) in principle (NPT) and you can live.

How could it be, what else? (laughter) (..) I think, I think. (laughter) I still have something from childhood, like, let's say, principle, choice, I never drank alcohol, well, cigarettes, that's understandable. And even when I... Well, that's how it is in Russia, they are always trying to offer something to you, and as if in theory, the majority drinks and considers it normal. (laughter) Yes, and when you don't drink, in general, they are all so surprised, well, come on, well, try it,

(laughter) but I always had a clear motive, this has been since childhood, that I am not going to drink and I will never. Well, as an example. Well, even when, relatively speaking, I was moving into a hostel, the neighbors somehow, well, there are three of us, and they suggested how to celebrate a housewarming, they asked me - will you? I'm like, no, I won't. (laughter) And they were like - well, okay. But I always hear this phrase - you will start someday anyway. That is, I don't drink at all. Not for the holidays, well, it doesn't matter. Just never and not at all. Here. This, so to speak, (sigh) is my principle and choice. (laughter) (...) So, I don't know how many minutes have already passed there. (laughter) It just happens, you don't remember everything right away. (..) Damn, I guess I, I can't think of anything yet, it's hard to come up with, that is, come up with.
M. But you also said that mom changed a little, softened her views. What does this have to do with? L. Oh, (laughter) with her life experience, probably. Well, oh, in general, yes... (..) Well, she looks, reads these psychological books, and something else. In general, it develops itself, so we plant it like that. (...) Because she doesn't like her life, she wants to change something in it. M. Lisa, was it really noticeable to you that suddenly she changed there and became softer? Or how? L. Yes, yes, she just talks a lot, and since she has no friends, in our city, well, they were, they all went to Tyumen. (laughter) And a new friend appeared, and she also left for Tyumen, (npt) And so it always turned out that she was talking to me and all these topics, well, I don't know, she talks a lot in general, as if about herself, then about how she lived there (sigh) then she speaks about herself, about her changes. Or it says that you can reprimand me there if you see that I'm going too far there. Well, in general it was not difficult for her. (laughter)
..) Rather, this is how it is, somehow significant, this is health. (...) So, health. (laughter) (...) I would probably classify it more for me as some kind of sport, some kind of sport, (..) they are connected. And, (npt) I also said that alcohol, (laughter) (npt) let's say, refuse (npt). That is, I don't want this one (npt) yet, but even before that, this is the last one. And as for sports, I, I have a new kind of sport, that is, always, (NPT) development, I liked it, there was a subscription for eight classes. This is something new for me. But as they say, well, jumping on a trampoline, doing (npt) or something like that, in my opinion. (npt) Then work. Work, well, work, here I chose, "Yandex.Food". I was choosing between Yandex.Food and Delivery Club, well, it seemed that in Yandex.Food, well, how to work there, on the contrary (npt), with large orders, but since I could, I had initial information... M. So, now everything is turned on, now, I hope, everything will be heard well and there will be no interruptions. L. Well, yes, I said that I was choosing between Delivery Club and Yandex.Food, and there was more difference, that is, I went to Yandex.Food. Oh, and then I also worked at Pyaterochka, but I initially went to the order area, since it turned out to be there from morning to evening, and I needed (..) evening part-time work, or for a number of hours. But, I said, they offered me a cashier job, and I, I had a choice... M. Yes, Lisa, something went wrong. Yes, about the order picker, and you were offered cashiers. Here in these words. L. Yes, I had a choice whether to agree or refuse. And I thought, okay, I'll agree, I'll try, why not. Although it was a little scary and exciting, in the end everything, so to speak, worked out, I was trained and I worked calmly, so to speak. Here. Later, but then I had to leave, so I quit. But I warned that I wouldn't be around for long and, in principle, (laughter) since they didn't quite suit me, (laughter) and it would have been a choice in general, (laughter) probably to quit. And, so, (npt) in my opinion, (..) I would roughly give an example, like choosing a vacation. Since it's difficult for me to get from Rostov to Nizhnevartovsk, so to speak, at the moment, since the airport is closed, and you can get there by roundabout routes, but it's expensive. Because from Moscow (laughter) to us one way it's 10 thousand, but I just had a direct flight, I could fly (npt) when I was lucky in principle to get it and when you already flew for 3, and for 5, and for 7, then you don't want this, well, yes, you don't want to go one way for 10. But in principle, it happens, it's a lot, so to speak,

well, I would come to my city, but there’s not much there, there is nothing to do, and in general, my mother and I agreed that we would meet in the summer, but not in the city, but somewhere, so to speak, for us on neutral territory. Well, we went to Sochi, that is, she (npt) flew, and I had to get there by train, and... (...) Well, we generally chose tickets, well, where is the best place to go, we chose these ones, where Sochi is. But since I was already there, a year ago, for a few days, it was somehow very, there is such an example, but... (sigh) So, (..) friendly relations. (...) Well, choice. (..) Well, here, probably, who would you like to be a friend, (laughter) and who would not. Don't know. Or, let’s say, where there is a person like this, I would like someone... (sigh) (...) So... (laughter) In general, they offered me to take a walk there, and I, (..) let’s say, I didn't want it in general. I don’t like this person, so I don’t want him to be (laughter) my friend. And you probably... Well, I kind of refuse. Don't want. Don't know. (laughter) What’s a better example to give, honestly. Well, I don’t know how I could have one friend here, (..) I don’t even know. I'm moving on for now. Place of residence, regarding the place of residence, by the way, I live in a dormitory, and initially I didn’t want to live in a dormitory, because, well, it seems to me that here, well, all sorts of events are held, as if there was a place to sit, let’s say I had to film apartment or something like that. And plus I have a hostel, it’s very comfortable, well, I just won a competition, (laughter) and the hostel in which I live, it’s like, well, it’s an apartment type, and that’s why (laughter) it’s here for me in general, not only that , that I’m like this in a hostel, where I have connections, but it’s also almost home. (laughter) But, of course, I didn’t know which one I would end up in, but initially I wanted to go to the hostel, so that there would be all sorts of these events in which I could participate. That is, renting in one there, in an apartment, is not interesting, it is boring. Here. (...) Everyday and important decisions. (sigh) (..) Well, about important decisions, everyday ones, probably just like where, where to go, if everyday, where to go, to have fun, to see. Well, also, for example, I took part in the race there in May, for me it was such an important decision. In such a major event, because it is not held here, and I have never participated. The Russian Federation race, which took place in May, I... Well, it costs money, and you kind of have to decide on it. (laughter) I took the plunge, bought it and didn’t regret it. And also (..) I liked it, I finished the race (npt) I also decided, I realized that I liked it, that (laughter) I need to train for it, and that, well, more.
M. Yes, but do you have any decisions in life that you consider unsuccessful? L. Solutions... Health related? M. In general, not only related to health, but are there any choices that you consider to be completely unsuccessful? L. Yes. The elections were unsuccessful. Well, (..) probably yes, yes. (laughter) But for me everything is connected with sports. (laughter) In the eighth grade, no, not even in the eighth, but earlier, in the sixth grade, they offered me to go to... (npt) To the All-Russian Olympiad for schoolchildren in physical education, but I refused then, and in the seventh they offered me, but I She also refused, or rather, at first she agreed, then for some reason she refused. In the eighth, (sigh) I also refused, and in the ninth, (laughter) and only in the tenth I came, and even then I agreed. And... I regret that I refused before, because I really wanted to go to the final stage, that is, to Russia, which took place then in the eleventh grade, in Kursk, that is, you go through the regional, well, first the school, then municipal, regional, and final. And (laughter) in general, two years of preparation is not enough, and if in the tenth grade I failed there, then in the eleventh, in the region, although I was a prize-winner, I took first place, but I was 17 hundredths short of Russia, and I was very I wanted to. And in general, I regretted then that I had not agreed to another school earlier. And in general, yes, and the fact that in addition to physical education there, let’s say I would also take part in mathematics, I somehow even thought about it... Well, I didn’t think about what the Olympiads are like, and what they give, and what in general is that? In general, then it was a bad choice that I refused. (sigh) In this regard. M. So you consider those elections unsuccessful, where you missed some opportunity? L. Well, probably yes. M. But out of

these several situations with physical education Olympiads, which of these, well, which of these situations is the most unfortunate, in your opinion? L. (...) Well, what's the worst? (laughter) The situation, well, (laughter) I didn't have enough points, (laughter) for Russia, these hundredths... Hundredths, well, it's just literally the whole point, or (npt) in gymnastics, or a second of running. (laughter) M. And you feel like you're blaming yourself a little for not pushing through? L. Yes. I just wanted, and even more than to pass the Unified State Exam well, that is, (laughter) well, damn it, I still can't come to terms with it. It's a shame. (laughter)
So I realized that as a result, you have not yet enrolled in any specific business specialty in order to do quests. But as for sports, why wouldn't it become some kind of professional field, well, in which you would become, well, a professional athlete, why not this? L. Yes, it's interesting, I mean, in general it's, well, firstly, we don't have that many sports in our city, and if it's all over there (npt), then basically, in principle, then in our regions... Well, even if not only all kinds of sports, Moscow has everything, I recently made such conclusions, but in the regions, in one there is no such thing, in another there is no such thing, in a third this is not the case, and since we have a lot of sports, I started somewhere with basketball, then I liked it, before I was banned anymore (npt), then I went to sports tourism for 2 years, it's not like It's just mountaineering, but that's the point, there's a program there, on the ropes like that. In principle, I liked it, but for some reason I got tired of it. I don't know. (laughter) Honestly, I got tired of it and just quit. (laughter) The same about athletics. And athletics, it's even more boring. Well, again, it's a cyclical sport, where you only do the same thing, and that is, we only have running and nothing else. There is no throwing or jumping, just running. And I'm not interested in running. (laughter) It's the same thing, in general, I'm truly not an athlete. I understand this because, well, I don't like it. Well, I don't know, and somehow other sports, I didn't try much, then I just somehow in the ninth, or even the eighth grade, (laughter) forgot that I was an athlete, and to some extent a sport was absent from my life, and then, in the tenth grade, I somehow remembered this, and here, when we were just preparing for the Olympiad in physical education, we have, in addition to, well, theory, gymnastics and running. And since I liked gymnastics, it's clear that it's not like real, professional, sports gymnasts, there's a lot of other things, and I (laughter) by the way, I regretted that damn, it's a pity I don't do this, but they only take from childhood. And my mother told me that she somehow brought me when I was 5 years old, but they refused me. But I don't remember this, they kind of told me that I was too big. (laughter) But I kind of liked it, and because of this I kept looking in Rostov for some kind of acrobatics for adults, gymnastics for adults, that's it. And trampoline jumping, but it is combined with gymnastics for adults. But the problem is that damn, it takes a day. (laughter) Well, yes, sort of. And so, well, I didn't manage to go to any professional sport, because I couldn't find one for myself. M. That is, if, perhaps, when you were younger, you would have been sent, well, sent somewhere... L. Yes, perhaps.
Then I went to school, and in the first grade I did synchronized swimming. All first grade. And then, the workload became so heavy that my mother suggested that I either change schools, or that I study in the second shift and be able to devote all the remaining time to training. Or stay in school and quit swimming. And she didn't decide this for me, she said that it was entirely my choice. And then, during that period, I decided that I would quit swimming. And I'll stay in school. Well, it seems to me that then I chose friends and some kind of social life, it seems to me, at the age I was, as if friends were more important, and sports had never been some kind of meaning of life for me. I went, I basically liked it, but he was never a priority for me. But now I think that... Now I don't think at all, but I used to think that if I had stayed, I would now be some kind of master of sports or take part in competitions, be an athlete. And life would have turned out completely differently. But nevertheless, I never regret the decisions I made, in any way. I studied the same way, in the same

school from first to eleventh grade. (..) I was like that, not very much, but that is, not a completely withdrawn child, but also not one who directly craves attention, that is, I was afraid of public speaking, but at the same time I communicated well with everyone, well, I was the kind of person who He seems to communicate with everyone, but only a few people are good there. But everyone treated me well. Then, the elections, where... In principle, even when I was in school, my parents always put the whole choice on me, they didn't decide anything for me. Which sections should I go to? I also tried a lot of different sports, only because I wanted it myself, and I left the same way because I wanted it myself. And it seems to me that this... Well, to some extent it's probably correct, perhaps those who... Those parents who want their child to actively engage in some kind of sport, who have their own ambitions, they somehow insist, but I, on the other hand, have tried a lot of different sports throughout my life, and I realized what I like to do. I still swim, for example, like it was with synchronized swimming, I realized that I like swimming, I still go to the pool. And when I started doing athletics, I realized that I like to run, but kind of (..) not... But I only like to run, for example. I don't like everything else we did. And I just left, just doing it for myself. Well, that is, it seems to me that we need to somehow give the child a choice, although they already asked me then... (...) What... (...)
Now, I have some kind of frantic energy, I want to meet someone all the time, learn something new, go somewhere new, to theaters, museums, concerts, walks, etc. whatever. I feel like I'm being filled up by this, I don't know. So I went, when I went to a psychologist, somehow after that I became more open, and then people themselves began to reach out, after that. (..) Oh, romantic relationships, that's also a ten. I have a very hard time with this, because it's also about opening up, you have to open up to people, and at some point I start to quickly, quickly close myself off, as if when I understand that this is some kind of... Well, it's not like that anymore, (..) when I realize that I am becoming a little vulnerable, I, (..) my brain seems to automatically begin to push away, that is, it is so much so that I even (..) at first may not notice it, and only when I analyze it later, that is, everything is so quirky that it seems to me at the moment when this happens that the reason that I chose is really important, but in fact then I understand that I just had some kind of fear and (..) I just, well, sort of decided to run away. Now I'm actively working with this, but this one is hard. For ten.

3.3 Table 3. Subjective health evaluations (mental health)

Citations
And in the end, the actions reached the point where the teachers all scolded me for a very long time, and were about to label me as, well, like, not a street child, but whatever it is, well, in short, register as a child. Here. But there it turned out to be somehow simple, I was simply put on the internal school register. And they said that if something happens again, then we will have the same horror. A little time passed, a week later the fight started again and then they started beating me, probably about seven people beat me, on the street, it was in winter. But at some point, I kind of just lay there angry, I don't know, something confused me, I abruptly shoved everyone who was standing, fought off with steps, quickly jumped up, kicked someone else there and caught up with one, and just I took the snow that was lying, and then there was some kind of ice and I decided, I thought that I would just wash his face with snow, it so happened that I took a piece of ice and it accidentally hit him in the temple. Nothing terrible happened there, the ice just cut the skin a little, that is, not even a cut, it was so strong. But in the end, I was still scared too, that's it, I won't go to school anymore. Let's go to some doctor and say that blah blah blah, let's check the child, I'm there, I don't remember exactly what happened. Well, in short, the doctors there determined that there

was some kind of prolonged depression, they prescribed the child, well, fifth grade, antidepressants, and they forced him to sit on antidepressants. I spent a long time on them, on the course, I don't really know, me, well, not systematically, but at times, my mother seemed to say to them - let's continue to drink this. And these were, I really don't remember, either some very mild tranquilizers, or antidepressants, I remember... They were probably called something, I've already forgotten. But the point was that my studies failed because of this, I just stood at the blackboard like a vegetable. I didn't have a single thought in my head and the teachers often looked sideways at me, and to themselves, that is, like this drug addict, to the narcologist... What's going on there. Carefully. I'm sorry, the girl dropped something. Here. We went to a narcologist, where he told me that, buddy, everything is fine, they showed him a prescription for these antidepressants, and for a long time they refused. But somewhere around the eighth grade, I realized that there was a huge difference in interaction between me and my classmates, that is, more and more of the time that I sat at my desk, I simply watched how my classmates' adolescence was passing. And at some point, like in some "Rocky," I stood up in my room and said - I won't drink, that's it. I refused. And somewhere from the eighth to the ninth and eleventh grades, I began a time of real rehabilitation, when I, well, there my first interactions began, just like those who were more mature. Here. There's a story with friends, one there, others, the fourth, it still didn't work out with the girls, that's it. But somewhere around the tenth or eleventh grade, I was already completely rehabilitated, both as if just on my own, and in the eyes of my peers, who saw me both in the sixth and seventh grade. And we kind of fell into the same sort of path, that is, the same groups, and on our own we seemed to communicate normally. And this, this dynasty that I carried with me from the bad events that were covered by my pills, somehow left. Then it so happened that even by my first year it all continued; I began to experience a simply stormy, simply huge, stormy life as a freshman. And I slept, it was so that I slept at night every other night, one night. That is, I worked in the morning, afternoon and evening on projects at the university, at night we went to parties, the next day I still worked in the morning, afternoon and evening, and at night I slept. There were nights when I didn't sleep, not because there were parties, but because I didn't have time to complete projects by the deadline, and I also had to work at night, because, of course, there were some situations, they just calculated it wrong , one of the teammates did not fulfill his duties there, and so on. Here. And I, as the leader of the project, just needed it to be completed. No matter what a person does, I will try for him now. Here. And my classmates, whom I later met, joked that I was speedrunning my life. Speedrun, this is true, just in case, for those who will decipher this later, this means accelerated, accelerated passage. That is, among the players there are those who compete to see who can complete the game faster. This is called speedrun. I was told that I was in the speedrun of my life. That is, in terms of (..) everything that they felt there during the entire time when they studied there from the fifth to the eleventh grade, I felt literally in 3 years, and even further, what they had not yet had time to experience, I already felt it. That is, I was carried away somewhere there.

M. Ilya, very detailed, thank you. In general, I'll start with your first phrase, when you started speaking, you said that reflection is always welcome. And as if from your story, it also came up a couple of times that you have, as it were, experience communicating with different psychologists. But tell me, tell me, are you, in general, inclined to reflect on your own, or how did this experience influence you, why are you talking about yourself in such detail, analytically? I. It's like two in one. Like these non-cafés. But it turned out like, the story itself from the 8th grade there, when I came off these antidepressants, well, it turned out that in order to understand this, it was necessary, of course, to reflect there. I have minimal communication with a psychologist, well, except for this psychologist I just talked about, 27 years old. Because every time I went to a psychologist, it ended, well, there were one, two, three or four trips. And they told me what I already know. And

most of the psychologists that I just went to said, “I don’t need to go to a psychologist, I calmly delve into my own head.” That is, I will scatter if I can. The only times when I actually went to a psychologist with benefit was when I went for IQ tests. This was when I went, I sat there for an hour with him, he solved the test, then yes, this was my only useful trip. And the rest, if they didn’t just interfere, somehow changed life for the worse. Although it is also unclear which one, good or bad. Probably a good one after all. Then the rest were useless. Well, it probably turned out that I myself am a reflective person, I even think at the Skolkovo university the moderators told me that at times I was even hyper-reflective, let me look for meaning where there is simply none. (..) But it so happened that the element in which we learn, it implies reflection after the end of each activity, project. Necessarily. That is, we have to sort everything out, what, why, but they didn’t teach it in a very good way. But when I practiced with the Skolkovo team in game technology, a prerequisite there was, as it were, reflection itself, game technology, and in detail, that is, I had to write a plan, with the tools that I used, that is, I said what I want to try on the guys there such and such, such and such a method, there is interaction between them, maybe there (npt) according to Weldon a couple of people there can move from an idea generator to an executor, and the like. And then come back and write a reflection. It didn't work out because like this, like this, like this. You need to try this, maybe something like this. So much so that I needed to read the reflections of those guys whom I sort of moderate myself. Therefore, it’s like I’m reflective myself, and it so happened that I also needed to practice this intensively.

M. I hear some kind of conflict between what you think and how you act. And how is it generally given to you, is it easy to make a choice in such a situation or not? That is, why do the scales still start to tip in one direction every time? M. (sigh) It's hard to say. Probably because... (..) How to say... Well, again, probably there is a point in psychology that people often treat others the way they want to be treated. And among other things, I give people, other people, a chance because I would like to be given a chance one day if I stumbled. This is exactly why I give people there some kind of warmth and so on, that is, everything, all these moments, because I myself lack it. That is, what happens is, let’s say, the opposite situation. That is, I give what I lack. Here. M. But this is generally interesting. How did you come to such an understanding, to such an experience? And to your own behavior? M. I unconsciously stumbled upon all sorts of psychological things a lot. (NPT) I unknowingly came across all sorts of psychological things, I don’t know, because you scroll through your VK feed and there, for example, there are some posts about psychology, something else, in the same TikTok you come across a lot of all sorts of psychological analyzes, oh behavioral types of a person and so on. So it all stuck to me like that and, in principle, some kind of awareness came. And that includes still coming. Here. And so... M. So, that is, you are not a naive respondent, right? And pumped up.

M. As if your experience of depression was for you also such a marker of where my friends are and what choices can I make in relation to them? And as if you, despite the fact that you are used to giving people a second chance, decided that you will not give them this chance? M. Well, I just, again, it seems to me that I have some kind of innate probably psychological things, well, that is, I can read people to some extent, I understand how they behave, how they will behave throughout to lead for a certain time, that is, it seems to me that I have some kind of psychological habits in terms of reading a person and so on. And all this. Understanding other people, including yourself. And based on this, I probably, well, probably don’t give these people a chance. That is, I understand that they will not change.

M. Well, yes, it seems that you hear yourself very well and understand what you need and what your needs are. Tell me, with your activities, what you called a hobby, did you also listen to yourself or perhaps it was imposed by your parents? M. Regarding sports, I probably always liked to do something like that, to feel some kind of power over my body, that is, when you grow,

including over yourself. Including remembering, as they say, (laughter) the classics of fight club, development through self-destruction, that’s also a wonderful thing. This is also wrong, but I probably came to this too.
M. Well, great. That is, in addition to your profession, you also have a hobby that gives you pleasure. And you seem to have mentioned something else in your hobbies, music, right? M. Yes. Yes. Here. Well, it’s more... There is some kind of story in me that I want to learn how to play well, and so on, but I’m probably too lazy for this, and even more so, it seems to me that I don’t have good potential for this . That is, I can play some banal things there, play something on chords, sort of a little strumming, that is, I can play, and... Well, it’s not like I studied there, that is, it’s more by instinct it sort of went, I liked it there, how I typed some kind of melody, I started to continue there. Here I am... M. How old were you when you started playing music? M. (sigh) (..) Oh, probably 18-19 somewhere. In this regard. M. And it was also your choice, right? Or... M. Yes, yes, it was my choice, completely. It’s just that at one point I realized that... Well, again, I’m probably a depressive psychotype, I don’t remember, if that’s what it’s called, so. And I bought myself a musical instrument simply because sometimes I’m in such a state that I just want to sit in the dark and strum something sad to myself. This somehow makes it easier. M. But it’s cool that you have such a resource that you know what to do in such conditions. M. Well, before, I used to, as they say, beat myself up with sad music, but still doing this, as they say, this is probably my favorite self-flagellation and so on. Well, it’s probably not self-flagellation, it’s probably just some kind of self-harming. Here. First I bought a ukulele, realized that it didn’t sound sad enough, and bought a guitar. (laughter) Here. There's more bass, more sadness can be created, let's say. Here. Well, sort of in this format. M. Yes, it’s interesting, that is, you can take this sadness out from the outside and create it yourself. That's great too. M. Yes. (laughter) M. No, it’s really great that sadness comes out of you and is expressed in creativity. This is great. M. Well, including... Oh, yes, by the way, I remembered another hobby, you said, expressing yourself in creativity, also not so long ago, a year ago, I think, I started writing a poem, here. Also, as they say, sadness had a very strong influence on this, along with depression, so. Because there is not a single funny poem that (laughter) I wrote. Because, it seems to me that the strongest emotion is the worst emotion. And based on this, here comes, well, let’s say, what I do.
A. Yes, I work in an IT company, well, it’s an ordinary development studio, I’m a backender. M. Tell me, do you have any hobbies, interests, besides study and work? A. Yes, my scientific interests are research in the field of education, I am mainly engaged in the history of the philosophy of university education. Psychology a little, I also do origami, sports are athletics, that’s it. Just like that. How long have you been doing sports? Two years. Well, intermittently, I did a lot of sports as a child, well, I consciously came to this (npt).
Health. Health is the area that suffers greatly in my life, I’m sick right now, right now. And I recently took a Covid test there, yesterday, and I’m waiting to see whether it will happen or not, but it seems that it won’t, I already feel fine. But, damn it, I haven’t been doing much about my health lately, but I just support it with sports and that’s it. But I would like to undergo some kind of examination, the fact is that the right side of my head has been hurting all my life, and it’s such a noise, it’s just as if, I can describe this pain as a noise. I've already gotten used to it, because I know that damn it, this never happens, once in the tenth grade I did an examination that did not give any intelligible results, they told me that I was healthy, I'm into it I don't really believe it. (laughter) Here. Still, something is wrong. Otherwise, health...
A. Oh, you know, this is very interesting, I had no thoughts. Well, that is, if we are talking about some kind of rational reflection, and I mean perspective reflection, that is, when we assess risks, when we, we don’t know, analyze a situation that could happen, it in general (xxxx) (laughter)

there was none. Well, that is, it's purely based on some kind of emotion, some kind of euphoria, it's probably possible, I didn't use psychoactive substances very much, I smoked marijuana twice, but it seems to me that this is something similar to what people experience under psychoactive substances, such an absolute frenzy and absolute lack of self-control. Here.
M. I see. How do you help your friends? Well, in fact, were there any similar interesting situations? A. I don't remember them so often, but most often these are situations related to a psychological and moral point of view, because they fall into some kind of depression, something bad happened to them, I try to support them, I remember sitting at night , talked to them, tried to support them, although he himself took the test in the morning. It was so. Here.
M. In general, well, now it turns out that you are discussing with someone some things that may be difficult for you, well, be it some psychologist, I don't know, a close person? T. Well, yes, there were moments, that is, when I visited a psychologist, that is, in principle, there were moments when I was just put in a leadership position, and I, having worked for only 2 years, yes, about two years , one and a half, in general at work, that is, I was put in a leadership position, naturally, there was stress. In general, there was a lot of stress and I needed some kind of psychological help, (..) it turns out to be qualified. Yes, I also consulted a psychologist. And in principle, this gave its micro-fruits, that is, well, perhaps, including the fact that I have already gotten used to it, that is, this so-called adaptation period has passed, and now I am more or less in a normal, so to speak, psychological rut and everything is fine.
T. Well, yes, that is, before, I'll interrupt, I'm sorry, I just had a problem before in that sense, that I, well, when you see a cat trying to attack a mouse, that is, it first prepares itself there, sits for a long time, waits, and at some point pounces. I've always had this in anticipation and preparation, just before communicating with a psychologist and so on, that is, I've always had this for a very long time, that is, I'm trying to calculate every little thing that is impossible to calculate, and sometimes just you miss your goals. This is exactly what is needed, it seems to me, we simply mean to wait, but not to wait out in general, roughly speaking. That is, to evaluate yes, more or less seriously, (npt) you act as you see fit. Here. Something like this.
when everything seems to be bad, but I always keep the thought that life, this is also such a clichéd concept, that life is like a zebra, that is, there is a black stripe, there is a white stripe. And I began to notice it too, it seems to me that maybe this is self-hypnosis, maybe, I don't know, it's more like you, as a psychologist, will say. (laughter) I also began to notice that yes, there are some lows in life, but there are always ups and downs in life. And... (..) And the same goes for good and bad things, for me too, that is, I have neutrality here, but in life, yes, there are some good and bad moments, just even bad ones moments, I never call them bad, I just think that this means now is the time, some kind of quiet, calm life, or doing nothing, or I don't know, it means that life is telling you - rest there, devote time , I don't know, some family issues, don't engage in public affairs. There and then next week you will have five new acquaintances there and ten new projects will arrive. And this is how it usually happens, I don't know, I was there (..) about two weeks ago, I think somehow, and when autumn began, probably at the beginning of September, I was just moping, moping, I think - nothing It turns out that it still doesn't seem to work and it seems like my colleagues and friends also have problems, somehow they need to be helped, no one helps them, and I can't help them either. And now, October has arrived and there are just so many different projects and you're thinking, how can I get rid of them as quickly as possible, from all these projects, because somehow they all just landed on your head, and you don't understand what to do with them? . (laughter)
M. But about the karate trainer, tell us how he helped you develop independence, since you speak about him so warmly and in detail. M1. Yes, I speak very warmly about him, about the coach, because, well, this overlaps with the fact that I spent a lot of time in training, a lot of time, from the

tenth grade I did karate, it got so bad that I had, let's say Monday, Wednesday, Friday, two workouts a day, 2 hours each, and the remaining time, let's say one. Each training session lasts somewhere for 2-3 hours and I was with him all the time, like listening to how much he loved to share some stories, how he not only enlightened us, but simply talked about all sorts of life topics, I heard him talk about it, I drew some conclusions for myself, what I can do, how I can do, similar things. Here. In general, just in my opinion, even in terms of education with a child, the main thing is to talk to him stupidly. Just talk. Tell some moments, life situations, how he got out of these situations, so that the child has some kind of template example that he could use in the event of some new situation. And then impose your own, having some kind of behavior model. That is, it seems to me that it is from conversations that a certain model of behavior is formed so that the child can use it and accept it as a given, in order to develop it later. He can completely redo this pattern of behavior, in case of some extreme situation he won't have to think, but what should I do, he will remember - yeah, my dad did this, or my mom did this, you can do it like this. He will do this, and then he will think, what is best for me? He will do what is best for him. That is, just in terms of conversations, all this is being formed. That is, the coach said that in reality martial arts are very connected with personal perception, and you cannot teach martial arts if you just come, roughly speaking, once a week to training and just somehow teach the technique. He said that all the masters, they lived with the teacher and seemed to watch him, how he behaved in the natural environment. They seemed to adopt the image, likeness, and mind of the teacher, how he behaves in a normal environment, outside of training, outside of sports. And this is the difference between a coach and a teacher. I can call my coach a teacher. For example, I can't call a judo coach a teacher, because his task was to make athletes out of us, well, he made athletes out of us, he is a professional in this matter, but there were no such life conversations. Because that was not his task. My karate coach had a different task. He talked to us a lot. Accordingly, well, somehow he is still just a teacher by training, I'll tell you what, at the university, so he knows what to talk about and how to teach. So it was very interesting and educational. And it seems to me that it was karate that revealed a certain personality in me, in terms of absence, removed some edges, that I can live calmly, socially, not be afraid to meet someone, do something else, because I don't know, how it happened, but I'm sure it was after karate, because martial arts associated with a certain kind of combat, they greatly change the worldview, perception, and seem to remove some kind of framework, boundaries. You become more confident. And quite independent. You could say. Because you are not afraid to defend your point of view in some dispute, knowing, like, that if someone comes at you, you will be able to give him an answer and give him a very good answer. M1. About the karate teacher and dash teacher, I was wondering if he has an education, you said that he teaches at a university, but what does he teach? M1. He is the dean of the physical education department of our Ryazan State University. M. How did you even get into his section? M1. This is also a very interesting story, I... (..) I just somehow saw how training was going on, in karate, in a completely different place, in a completely separate place, in the Krasnodar region, when I was with my grandparents in away, I saw how the training was going on. I was so impressed by this, because at that moment I was doing boxing, and I lacked some kind of morality, some kind of cultural core, when before training everyone bowed, sat down, closed their eyes, well, not just prayed, but they just sat down as if getting ready for training. After training, a bow, ritual phrases, when there is movement and intelligence, not just a straight punch, a side kick, but some beautiful Japanese names, Japanese language, culture, kimono, that's it, equipment, I really liked it, it went in, and I I started looking for this section in my city, I found a section in my city. It was another coach there, who reported, in fact, to the head coach, whom I spoke of as a teacher, I went to him first, but then this coach said, that there was an opportunity to go to some training

camp, go to another building, with this head coach, so I went and somehow it happened that the coach liked me and he decided that I would work with him in the training camp team, national team . And I began to study with him. And I spent, in about six months, actually, I actually studied for six months, I went along the path to a black belt, in fact, because I worked a lot, plowed, studied, learned new things. This is my story of meeting this karate trainer. Now we don’t communicate, but I remember him very often, that is, in some conversations, and I am very glad that such a person appeared on my life’s path.
Well, this is what happens in life, in general, it always happens, for example, if there was a need to get answers to some questions and close your own questions, whether for business or personal reasons, I found a good psychologist, went to a psychologist, solved the questions, closed questions. Somehow like this. Should I answer in this format? Is that normal? Is it clear now?
M. But in general, this is the period before yours, before your state now, right? M. Yes, there was also the military registration and enlistment office story. M. Tell me more about this. M. Well, I was in such a disgusting state of mind that the doctors and the military commissar said, no dear, you are somehow really bad, let us observe you in the hospital and sort of decide whether you need to go there or not. In general, I spent 7 unforgettable days in the Gannushkin Design Bureau, well, I lay there, I just lived there, like, I don’t know, in a hotel. Here. And... Well, I was declared unfit, given category “B”, I never left to serve anywhere. M. How would you characterize this period of your life? M. Well, these 7 days, it would seem, should have been quite difficult, but the only difficult thing was the fact of being there. The stay itself, in fact, gave me some kind of respite or something, because it was even harder at home then. Here. M. So it was some kind of escape? From what was going on at home? M. Escape, escape. M. What happened, how did you react later when you were declared unfit? M. Well, it’s a relief, because if I left, I would feel even worse.
M. Yes, then you can move on to the next point. M. Choice of lifestyle and leisure. Well, this is where it gets more interesting, by the way, because I decided to change my lifestyle. He was like this, he is like this now, more sedentary, not very active, I found myself in such a rather interesting situation that after my previous job and previous company, all my interests simply died, because all my time was taken up by this sweatshop work and gatherings there with drinking there and so on. The rest just somehow fell away. And now I think what is interesting to me, what is not interesting to me, I restored my physical activity to a minimum there, in the future I will... In the future I will return to sports. I’ve even already decided what exactly it will be, what it will be.
M. And what will it be? M. I want to go to boxing. It's very necessary, in fact. M. Why boxing? M. Well, because it is he who has a great psychological influence on people. Well, according to my observations. This is both confidence and the ability to stand up for yourself, which is very important to me.
M. Completely, I don’t remember the last time I drank. In fact, with addictions everything is very interesting, I can, as if I don’t want to and I don’t. I also tried smoking then, but I didn’t understand this joke at all, to be honest, I just started and ended. Moreover, alcohol also makes me feel bad. Because alcohol is such a depressant or something. That I won't go back there at all. But I didn’t have time to develop alcoholism, I... I don’t have that.
And I started, that’s how I played computer games for 16 hours, in the same way, for 16 hours, I read literature and classics of literature. And of course I tried to write poetry. Here. And then, in all the blogs that I kept on games, I began to write about the fact that games are, of course, cool, (npt) but it is necessary, well, the trick is that they greatly, they very much limit the space in which you're cool. And I really love being cool, still do. Here. And how could I, of course, be the top one in the game, but if I go for a walk around Moscow, it will give me little. Here. I wanted to develop something with more generally accepted values, in some more generally accepted values. Well, that is, for example, knowledge of foreign languages, erudition, career prospects, and so on. So, at

the age of 16, at 16, yes, at 16 and a half, in short, somewhere there, at the end of 11th grade, I realized this and began to work in this direction.
V. Yes. Yes, I wouldn't even call it stress. I kind of liked this state of competition again, another one, here. Well, so did the entrance exams, and I was like, oh, I entered Moscow State University without a basic philosophical education, that's fine, now I'm also a translator. In general, this energizes me. So, in short, I always loved this state of competition, and as a child, when you are not competing in the professional market, this is probably one of the only niches where you can compete, these are games, well, such games, board, computer, or sports games. Here, by the way, I would actually dwell here in a little more detail, because I love, since I love this whole business, I did both, and the third, that is, I always played board games with pleasure, a la cards and sports games. I say, I played chess as a child and table tennis. Here. But... But just at the age of about 12 years old, that is, at 11, I started playing computer games, and at the age of 12, I left sports and stayed only in computer games. Here. I wonder why this happened, well, I think I don't know. (laughter)
In general, in principle, I don't like, I don't think that anyone knows better than me, especially in pedagogy, in matters of education, because I have a psychologist's education, pedagogical, I know perfectly well that no one knows my educational does not diagnose needs. Moreover, no one has as much knowledge as I do about which method of presenting material is suitable for me, so this education system does not give me a choice, well, let's say this happens often, the curriculum says that there are many disciplines by choice, but in fact there is nothing there anywhere, plus the budget needs to be distributed correctly, in the end there is one, well, at most two, you have a choice. In general, I don't like this all the time, I like it when I build this educational route for myself. M. Well, that is, studying at the psychology department helped you understand yourself? Q. I don't think it was specifically my training at the psychology department, although... I think that it structured my thinking, of course, certainly my thinking and worldview. In principle, psychology, pedagogy, and philosophy, which I studied, help me understand myself better.
V. Yes, well, accordingly, I created my own group, and spent the whole last year working with this group. And now I want to try again, now I really don't know, but I still hope to somehow go to study in France, so here I have the same problem, that I'm not a psychologist, that I'm a psychologist-educator, and France is more strict system in terms of changing qualifications, so I need a year at a Russian university. Here. Therefore, I want to study at a Russian university for a year at the Russian State University for the Humanities, and then try to transfer to... Well, there is the city of Caen and there is training for translators in three languages - Russian, English, French, and so on.
M. Tell me, do you remember the transition from your state of complete hopelessness to the fact that I can do something and in general, well, I can be responsible for my life and can take some action? So what was this connected with? Any external circumstances or more of your internal transformation? V. Tax. (...) Now, I'm trying to remember. So so so. (...) Well, at first, yes, I would probably describe it this way: at first I was just in shock, I didn't understand what was happening at all. Here. Then I began to feel the consequences, that I began to gain weight, and then I began to worry. Well, that everything is not going according to the best scenario that could have been hoped for. Here. Then, it's not like there was some kind of sudden event, it's just me, this question bothered me, it's like, I like to talk to myself, so. According to the method, like these two chairs, like what are you experiencing. How would I do something like this? So, this guy talked, so, okay, what do we want? We want to worry, or we want something, not to worry. Here. Let's think about what we can do. So, well, we, I mean, I just address myself like that, (laughter) here. M. Well, maybe some parts of you. V. Yes, yes, we are... M. They decided to act together. V. Yes, yes, yes.

Well, yes, we are me, torn apart by the contradictions that exist within me. Here. As a result, I talked to myself, came to the conclusion that I... Yes, another thing... Yes, I remembered, I was very worried, really very much, and it always scared me... And I was even worried not so much because of what happened, but because I didn’t understand what to do next. Here.
Well, a good choice, a good choice, I would probably attribute to my first university, that is, psychological and pedagogical education. And again, this is one of those choices that I made, guided by one criteria, and with the hope of one result, but it turned out to be good, that is, the results turned out to be different, but better. Than I could have imagined. That is, I chose a psychological-pedagogical course, because I knew biology, chemistry, mathematics, Russian, with these subjects I could enroll either in some kind of biological-chemical thing, like there you could go to Timiryazevka to study genetics, or to honey didn't pass. Well, in short, there was an option, either to go for something like that, biological, I was basically interested in genetics, at school I loved problems on genetics, but on the other hand, the option was to go to a psychologist, a psychologist-teacher, that’s it. And, in fact, I thought, damn, now I’ll go see this geneticist. Although I like solving these problems, is it that I will spend my whole life developing new plant varieties, animal breeds and stamps, strains of microorganisms? This is like the definition of genetics, actually. (npt) I think, God forbid, this is boring. I want to communicate with people. And I went to become a psychologist-educator. And why is this still a good choice, because I really liked my university, the Moscow State Pedagogical University, it’s not very overloading, I can, as a person who has already studied at a bunch of universities, and who has many friends from different universities, I know that it is so, moderately stressful. And there was a little more tension in the first year, and then just not at all, not very much. And I really liked it, because I think that if I had studied at another university, I would not have been able to simultaneously learn French, for example, to a high level, because I would have to complete some endless tasks there. And I would not have been able to gain work experience either at Artek or at Detsky Mir, or as a chess teacher, I was still working. In general, I would not have gained much work experience. I would describe the choice of university as successful, but now let’s talk about your criteria. So, when this happened - in 2015, who was among the participants in this situation? Well, I can say that naturally I was the main actor, but here my parents were naturally involved, like the parents of many children after the 11th grade, so. They gave me some advice there. I probably made this choice at the dacha. I was just wondering where, well, I was looking. I sat with the lists and in the end decided that, well, I went to different ones, and decided that psychological and pedagogical education, it still sounds universal, like a psychologist and a teacher. Of course, this is actually a trap, if anything, it’s a trap. In fact, you won’t be able to work as both a teacher and a psychologist (laughter) because you don’t have a subject to teach. (laughter) And the psychologist is also only a school psychologist. Here. (laughter) But I didn’t know that then.
M. Yes, Dima, thank you for the detailed story. I have a few clarifying questions. Firstly, I notice, well, naturally, here there is a moment of professional sociological education, albeit lasting one year, that you use a lot of terms, and with them you seem to indicate some kind of processes that arise in your life, but according to Basically, I have the following question, sometimes you say, for example, that there’s this, this stupid procrastination, or... That is, you give some kind of assessments, and in connection with this it’s interesting, but you’re with someone... Are you discussing it now, or have you discussed those difficulties, including psychological ones, that you have encountered, and sometimes, well, naturally, they arise in life? D. I discuss them, but I discuss them mainly with friends. With my family, (.) I don’t have the habit of sharing anything in particular and asking for advice, for some reason I have a big prejudice in this regard. It just seems to me that older people, my mother is a provincial teacher there, she is unlikely to understand (.) the things that I can tell her about. And you don’t discuss it with friends, but only after the fact.

Now here, with newfound acquaintances, I can discuss what I went through, but every time I go directly through a crisis, I usually don't share it with anyone, well, also probably because I don't think that anyone something can help me with this. Therefore, this is only after the fact.
About training, professional development - I decided to engage in self-development in the field of programming, I selected courses for myself that, in an amicable way, I would need to take. Because, in fact, I personally plan to work in the field of (NPT). Well, that's probably all. Health, I'm in... Six months ago, when my dark streak of procrastination ended, I started playing sports again, going to the gym. And during that period, I tried to arrange healthy meals for myself in the hostel. I stopped subsisting on some fast food and other semi-finished products and started cooking for myself. choose a diet.
I try to be kinder to myself and to others. Excessive self-flagellation and excess severity towards your friends, it rarely bears fruit, I try to refuse it. (...)
B. Well, it's quite hard for me to say about my peers, but I don't think that everyone actually thinks about this in any specific way, well, that is, it's still some kind of process that happens one way or another, in most cases, on its own with myself. Personally, I'm just getting this from, in principle, this is part of why I decided to take part in this interview, I'm interested in the topic of psychology, I have a good friend who is studying at Moscow State University of Psychology and Education, if you know, to become a (NPT) psychotherapist.
B. I personally find out about this from some sources, like Telegram channels, Instagram of some well-known psychotherapy services that run, some of them make short posts on some topics, that's it. Well, sometimes I read some articles, but just considering that I don't seem to know much, I approach it carefully. But in general, this is probably the most important source of information about this topic. Well, about other, some psychological topics too.
M. Where do you study and for whom? M1. I'm studying at the Higher School of Economics to become a psychologist.
Well, perhaps, when my neighbor became ill, she began to choke, and... That is, a person is suffocating, he doesn't know what to do, this is his first time, and it's like... That is, I called an ambulance, there I woke up everyone , she said that yes, we started googling something together, what to do and so on, that is, as if she had something like this, nervous, panicky, that's it. But then she went to the doctor, well, it was like, you know, an independent decision that at the moment I should help the person. Because she can hardly help herself. M. This was probably a very stressful event and somehow you reflected on the moment when - now I'm doing it, or did you just start doing it yourself because you realized that the responsibility was only on you at this moment? M1. I guess I just started doing it on my own. M. Do you think this situation played any role for your independence, or did you simply show what you already had? M1. I think I just manifested what I already had, because my sister is often sick, (laughter) more precisely constantly, so I kind of got used to packing things in bags when I have to go to the hospital, that is, for me it's a familiar situation, so it was sudden in terms of its appearance, but in terms of (..) how should I say it, in terms of factors, it was similar to many situations in life.
M1. Now I'll think about it. (...) So, well, one successful one. (..) I was in the eleventh, no, tenth grade, I wanted a dog, and at that moment I already understood that I definitely (xx) would not stay in my hometown, in Rostov, that I would definitely move. It doesn't matter which college I go to, but I'll definitely move. And... But I really wanted a dog. And as if we were looking for a dog, we found it, we had already paid a deposit, well, like, in a few days I have to go pick it up, and I understand that how can this be, then I will leave the dog with my parents, because I will live in a hostel , well, for sure, I had a choice either not to get a dog, and let's say, not to fulfill my dream, because I never had my own dog, we have a lot of dogs in our family, but we didn't have our own.

And no one knows when I would be able to get myself a dog. Or cancel everything, let’s put it this way, and simply not burden the parents. And that was the choice. Either you blindly follow your dream, then you burden other people because they need to take care of your animal, or you care about other people more than your desires. This happened in the tenth grade, I was probably 17 years old at the time, and me, my mom and my dad were involved in this situation. Because my sister doesn’t live with us, therefore she won’t take care of her. Where this happened, this happened in my hometown, this situation made me feel like I needed to decide something, that I needed to do something and that I was afraid of making a mistake. I’m afraid of ruining the dog’s life there, I don’t know, causing some kind of trauma to myself, or burdening my parents with this too. That is, the situation was not pleasant. The choice was not a pleasant one. But now, (..) I treat her calmly, I can’t say that she directly somehow excites something in me, probably, I just remember with some pleasantness all this then... All These are my decisions that led to the fact that I now have a dog. (..) It evoked different thoughts. (...) Don't even know. I think, probably, I was just very afraid to hang some kind of responsibility on another person, I was very afraid that the animal would forget its owner... And it’s not very pleasant for me either. (NPT) kind of like for myself, but like for others, too, so. Now this gives me (..) also mixed feelings. Well, that is, I don’t know why I did this then and decided to get a dog after all, and I don’t know what I would do with this situation now, well, that is, it’s like you just decide in the moment, yes or no, and probably Then I just let go of all the consequences and lived in this moment, that yes, now I want a dog, now let’s live in this gorgeous moment, and then I will accept the consequences and decide what to do with them. Here. But I think that it turned out well, because I love her very much, I have a gorgeous friend who will always hug you, lick something, and so on, make you laugh, play with you, that is, how These would be very pleasant moments, memories, so I think that I made a good choice.

probably my sister also has health problems, so she, a little like this, somehow maximally, (sigh) I don’t know, reaches maximum heights during education, and therefore, probably, this is also a little bit of my responsibility, (laughter) also everyone, and they also pay a lot of attention to her education, even more than mine, because it was more difficult for her to study, but certain hopes were probably pinned on me in this regard, but I’m not exactly (npt) on this topic of approval, that you should do something there, always do everything well, I don’t know. (laughter) Well, yes, in general, this was also important to my grandparents, but what my grandparents on my father’s side, basically, my grandmother was more likely to just say, go ahead, well, it was important to her that I do in basically anything, and I moved somewhere forward, and managed to do everything. But it didn’t matter to her in what area I was doing it. Here. Probably so. M. But it turns out that both pairs of grandparents have higher education? I. Yes, everyone has a higher education, yes. M. I’ll carefully ask about my sister: what health problems did she have, were and still have, what is the reason for this? I. She has autism spectrum disorder. But she is quite highly functional, that is, there is no such thing that she, I don’t know, it’s probably not very noticeable, in terms of... (..) For people who are unfamiliar with this, they probably won’t immediately understand that something is wrong specifically. That is, they may think that she communicates in some strange way, or something, I don’t know. Well, in general, not so much that she couldn’t study, for example, but in life it interferes with her. M. But you said that due to the fact that your sister has such difficulties, sometimes they even paid less attention to you, and so you, well, how much did this bother you? That is, did it evoke any emotions? I. In general, no, probably (laughter) only because they always paid a lot of attention to me, and sometimes, I would say that it was even some kind of overprotection, so for me, I don’t know, it’s just very difficult to assess the effect and the cause , that, for example, they looked after me a lot, because my sister has difficulties, for example, for a very long time I was not allowed to go anywhere on my own, (..) well, on the subway, or on some

public transport, and they were always driven, and it annoyed me because I wanted to travel on my own. And I don't know what this is connected with, maybe because my sister couldn't drive for a long time, and I started driving earlier, my parents were afraid to let me go. It seems to me that some of my parents' fears, as it were, related to my sister's health, are transferred to me. Well, at least it was like that before. Probably not anymore. But maybe, maybe I'm just thinking, in general, I've never had the feeling that they pay more attention to my sister, because I also received a lot of attention. And vice versa, probably at some moments it was good that this attention did not go to me and I, I had more freedom.
M. Did you feel older precisely because she had just such health difficulties? I. Yes, because for quite a long time, but even now, she still doesn't have it so strongly, for a very long time she behaved as if she were much younger. Therefore, at some point, well, I remember, right when I realized that she had some peculiarities, I asked my mother, by the way, I even remember this moment, I was driving, we were going either to a dance or with dances, and my mother began to tell me, I was about eight or nine, and she gave me a book to read, well, it's such a cool book, "Autism through the eyes of a sister," it's like from the point of view of a girl whose brother has autism and she tells . I think yes, it's quite similar, because some patterns of behavior are described there very, very similarly. Here. And when I realized this, I somehow began to relate to her easier, because when you know why she behaves so strangely and it annoys you, and it's not clear what to do about it, when you know it, it's easier. But still, I noticed that my sister acted as if she were younger, as if she were younger than me. Therefore, I didn't have the feeling that she was older, and that somehow... In general, I was probably ahead in terms of development after all.
M. But it seems as if you have done a lot of work compared to the way you talk about your adolescence and the way you behave now. You are very open, you are very emotional, and talking about such experiences is a great courage. In fact. N. (laughter) I have chosen a psychotherapist several times and now I finally go to a normal one, and everything, plus or minus, yes, is being worked out, so it's just... Well, that's exactly what I'm talking about, about the fact that from the age of eighteen, I can finally make choices myself, and I can, as it were, in an emotional way, that is, choose what I feel, what I don't feel, and what, I can behave this way and be like this, that is, in some moral things, and in some actions. That is, I decide where I will do the actions one day, and I chose this, and I will, well, kind of lead you like this. And be like that. This is also like a choice, in my head, it's considered, because, well, just then, all these things, they were very strong, I cried there for months, and in general, I felt even more downtrodden, but fortunately, some things started there. then a fairly adequate relationship that lasted quite a long time, and this somehow made me feel that I was not at all... There is someone who loves me, someone who appreciates me, the same one like this... Replacing unconditional love with parental love, that's it. And probably, in parallel with all this, probably in parallel with this, there were also elections in the 11th grade... M. Can I have a second? Tell me, do you attribute your transformation to a greater extent to psychotherapy, or perhaps there was also some event that made you look at yourself differently and begin to behave differently. N. More likely, to a lesser extent with psychotherapy, but that is, I started adequate psychotherapy literally four months ago, because before that I just went to psychotherapists, and somehow, well, I couldn't find someone with whom I would be comfortable, Here. So I associate it rather with the fact that the most important thing is that now I can, since I was eighteen, I do not depend on my parents, that is, if before that my parents could forbid me something and somehow control me, now, well, this is absolutely not the case, and we have some kind of, well, adequate distance, Well, you know, there is an expression, healthy indifference, something like this, probably with us. It's clear that I love them and this is unconditional and absolute, but how could I...

M. And after 18 years, what important elections did you have? Besides work. Maybe in some other areas? Independent travel, I heard this spring, maybe something else? Choosing a psychotherapist? N. Yes, choosing a psychotherapist. That's for sure. (laughter) An important choice, I'm very glad that I found my current therapist, she's very strong there. Yes, independent travel, because at first I started traveling around Russia, it was also funny, of course, because I turned 18 and Covid began. Like hello, thank you. Nastya, who had never been abroad before, or even in any other cities, here. Yes. So I myself began to travel slowly around Russia, sometimes in a company, sometimes on my own, and then I decided - damn it, I've never been abroad. Here. And yes, in general, I'm very glad that I went to Istanbul, so. Because I went there myself too, that's it. That is, completely alone, not knowing much (laughter) English, that's it. But it was great and I'm very, very glad that I went after all, because well, that's exactly my trip, I bought tickets there a few weeks before February 24, and the tickets that I repurchased were canceled 10 thousand times , and I was already thinking, damn, how am I going to go in such an atmosphere, but I went anyway. And I'm very glad, because... Well, at least this ticked some box, because you still felt as if you were in some kind of geographical cage and (npt) get me out. And this summer I also went to Dagestan, on my own. And this was also wonderful, it seemed to slightly support your own feeling that you are doing something with your life, and you are sort of solving not only some work or romantic processes, so. But you also allow yourself to receive some kind of pleasure. Here. Yes, what other elections, well, probably a partner, we still have to talk about it, because this is my first breakup with that young man with whom we dated for two and a half years, this is probably an important decision that I accepted. And after that we dated for a year, because I, in fact, I left him for another partner, with whom we were in a relationship for a year, it was such a very cinematic relationship, with... I think you know the expression Dead Inside, here, (laughter) this is it. (laughter) So, in the end, we parted ways, well, that is, I can generally say that he abandoned me there... Because of his own pens, because he was somehow against me projected, as they say, and this was also a rather difficult period, just around March - April, all this was also layered on February 24, and (..) there I did not make any choices, (laughter) this is exactly there was a moment when I directly felt that I had lost some control, and because of this, well, it was really very difficult. Well, it's also related to this, that is, I found a psychotherapist right on the same day. Here. And thanks to the fact that I decided to find her at all, and because I found her, I somehow, well, generally survived all these moments, because of course the first separation was very difficult, well, actually without your will, which was done. Here. Well, then there, after some time, it seems that I met a young man with whom we now have, well, some kind of relationship begins, this can also be considered some kind of, well, choice in the romantic part is important, because Well, there is 50 percent of work that calms you down, and there is also, well, it's clear that it's not 50, there are other breakdowns, probably 30 percent of work, 30 percent of relationships, 30 percent of friends, and then there's 10 percent, this is some kind of totality , oh well, the family still needs to give some part of the interest, so. Well, that is, I just remember that just when I was in Dagestan, I felt very happy, because finally everything in my life, plus or minus, seemed to work out, and this, well, if you look at all these segments, Well, they're pretty good. Here. That's why...
N. Well, then, I think that I can go back to the choice that I made when I was 16, when I was in the summer, when I decided to go to the young man with whom, who hinted to me for sex, for some kind of romantic relationship, that's it. And to his friend's house. Well, accordingly, I was 16, that is, what year was it, 2018, yes, it seems. Yes, summer 2018. Here. (..) Accordingly, I was there, this young man was there, and his friend was there. And so I ran away from home to spend time with them, because it seemed to me that it would be cool and that I would probably get closer to him, and indeed, he would kind of like me as a person, and that's how he would like me probably appreciate it more. Well, in principle, I liked the feeling that he was paying some attention to me,

that a person seemed to appear who appreciated me, at least for something. Here. Maybe sexual desire, and it seemed to me that, well, in general, I could probably somehow, well, show interest, and he would appreciate me even more, but for something else. Here. And all this took place in the Moscow region, at his dacha, in his house. And... Well, it happened, (laughter) how to describe it, I just don’t want to go into any super details, but (..) in the end, after we first talked with his friend, who told me what - there with these vodkas, so, and then this friend himself came, oh, not a friend, but the young man himself, with whom we had some kind of fuss, so, and he took me upstairs, and then I continued I remember it very well, (..) just in very small snatches, here. But it was all connected with some kind of (sigh) (..) well, sexual actions. In the end, I don’t know how he would have entered me or not, but he tried, let’s say so. Here. And what kind of feelings this situation caused then, obviously - not the most pleasant ones, well, that is, I was probably in some kind of teenage age, so that everyone, I was just, I was just worried about everything. Well, it seems to me that everything was just finishing me off gradually, that is, it wasn’t so much the situation itself that was finishing me off, that is, the fact that... Although, well, in general, it’s difficult for me to separate this for me, the fact that we then stopped communicating with him , that is, he seemed to write to me at the beginning, well, that is, maybe he wrote there twice, so. And that’s all for me... (..) Well, sort of, the fact that he’s no longer interested in me at all, and the fact that he absolutely doesn’t need me, and the fact that no one loves me, doesn’t appreciate me, I I felt absolute loneliness. I remember that I imagined that I, firstly, well, that is, I just had pictures in my head, like I was alone in the middle of a white sheet of paper, and in general there was no one close to me, as if my parents don’t give a fuck, my friends and I are so close We don't communicate, that's it. And, well, that is, it just felt like such total loneliness, rather, this whole situation led to these feelings, so. And what feelings now, (sigh) does this situation evoke, now, for a second. (...) (sigh) Well, probably (..) I just thought it through, thought it over 10 million, billion times, turned it over in my head, and somehow the psychotherapist and I seemed to have worked through it, give or take. .. And now I just feel some kind of, I don’t know, sadness, sympathy, probably for myself, but it’s global, because, well, obviously none of this would have happened if I felt somehow there much better, that is, as confident and calm as she is now.
N. (sob) Thank you, it’s very nice to hear all this. It’s just that now it’s actually much easier for me to tell all this, because it’s all already agreed upon with my loved ones, and well, my friends, my former partner, or my current partner. And to the psychotherapist, here. That is, like another year or two after what happened, of course it would have been more difficult for me to tell all this, but now it’s like... Well, you just understand that... Well, somehow in general, it’s all rational, meaningful and... But thank you very much for the words of support, it’s very (..) nice every time, me too...
M. I really like the way you praise yourself, it’s really great that you acknowledge your victories, understand and build it into your story, you understand that yes, I really did it right, and now I get what I wanted, and even unsuccessful elections in the past become not so unsuccessful; on the contrary, I am very glad that I made a different decision then. N. Well, I think that this is all kind of connected, plus or minus, with the fact that I turned eighteen, I began, plus or minus, to somehow adequately perceive myself and the world, and then again, now it’s like I’ve added to all this psychotherapy, and but I just, as if my emotional intelligence is growing a little, you can, well, somehow listen to what you feel and already determine what you feel, and not just feel and be like - oh, I feel bad, (laughter) here.
M. Yes, of course. If you think that's it, move on. A. Relationships with parents. This is a very difficult question, because... Well, I have always had a very (..) (h) great attachment with my parents, because they literally did not let me go anywhere, and it was this year that I decided that I

would completely separate from them, I worked with a psychologist about this. And... (..) And I tried to distance myself from them as much as possible, because I understood that I was already independent, I was already old enough to make decisions on my own. And, (..) well, not even listen to any advice, realizing that my experience, conditionally, of living in Moscow, studying and working is completely different, like theirs, in another city, and since we are completely different generations , because, well, my parents are adults. Here. And in this regard, it turns out that relations with parents may have cooled somewhat, and they are not as active as they were before. (...) Are there any additional questions on this point?
M. Please tell us in more detail how it happened that you wanted to start volunteering? A. I accidentally saw an announcement about holding a marathon, in general, in my city, I had to run it, but there was some kind of large fee, because, well, I used to do athletics, and I thought that I could run, but there there was some kind of large contribution, and I thought that well, I don't really want to ask my parents for money, and I saw that volunteers were needed there, and I simply wrote to the post office. And after 2 months they answered me, here, come. And then again, again, again, again, and so on, now several projects a month. (laughter) Or maybe even a week, and in general, now no longer as a volunteer, and not only there on all-Russian, but also on international projects. Even now, no longer as a volunteer, but as a manager of volunteer projects. M. Great. Is this related to sports, are these different projects, or is it one direction? A. We did this with sports, but now sports are a big, well, sports projects, they are a big part of my life. And the rest of the projects are of different directions, just the very fact of volunteering, the very fact of helping, it remains unchanged. M. Can you tell me what feelings the situation caused you to decide to volunteer? A. Then it was very interesting to me, because this is new communication, this is a new community, these are new opportunities, because I really missed communication exactly in adolescence, then it caused a feeling of such, perhaps instant admiration, that is happiness is in the moment, now this also happens from time to time, precisely at some event, but you just realize that you have something to do, and you can go to any event and go help someone. M. How do you feel about this now? A. Now I'm completely delighted, because you live this, you love doing this, when you're at some event that you really like, you just almost cry with happiness that you're there.
And probably, I also really wanted to do rhythmic gymnastics, I watched a lot of all sorts of videos, there were performances at the Olympics, at some sports competitions. And I kept asking, asking my parents to bring me to the gymnastics group, and then I still don't know, maybe my persuasion somehow influenced my family, maybe something else, that's it, but I still They brought me in and I started playing sports. And I understand that it's probably sport, it has played a lot in my life and in my character, too, a lot from sport. I have there, regarding the achievement of any goals that I set for myself, that I can gather there, even if I understand that it's hard for me, bad, or something else, I understand what I have after all, there's this kind of core that probably doesn't allow you to give up when you just need to pull yourself together and do something, come up with something, that's it. Or somehow cope with the situation that exists. Here. And probably the fact that in my family, in general, (..) there is such a moment of complete freedom. That is, I chose, after school I entered a technical school, because I did not enter the university, and my parents said that you choose a profession and direction yourself, because we want you to be a good person. And of course, perhaps, I think that this was bad advice, because when you are 16 years old, and you want to get specific, well, some steps, how to choose, what to do, maybe how to look at the test results, according to career guidance, which shows you 16 areas of activity, and you don't understand what to choose. Here. But it seems to me that this is my path in education, it seemed to be a little in different directions, but it seemed like it was almost cultural, almost literary, probably. Therefore, somehow everything comes together, as if like a puzzle, here you are,

seemingly from different places, but you find puzzles that your picture is missing. It's probably something like this.
And it was six months of hell, and some kind of moral torment, when they just constantly call you, I don’t know, a person who doesn’t know how to do anything, can’t do anything, and that everyone has already written a diploma, but you haven’t, and it was just what Here's a psychological panic for six months, that's it.
M. Tell me, how did the desire to go to study journalism come about? Why did you decide? L. Well, in general, until the seventh grade I wanted to be a psychologist, so. But... M. How interesting. L. Yes, my dad is just a psychologist, that’s it. But in short, there was such a story that I began to find out how to enter the psychology department, and found out that I had to take biology there. M. Biology, yes. L. And biology was generally my favorite subject at school, and I say - no, I’ll look for biology (npt) for something else. And so I started buying all sorts of magazines there, these ones, for girls, (npt) then later, I became interested in all kinds of fashion journalism, and then it means, I don’t know, there in the eighth grade, in the ninth, I became more interested in politics, I wanted to be a journalist who travels to all sorts of hot spots, that is, something like that, little by little it became more and more boring, then I just wanted to be some kind of observer, that’s it. Well, that is, it was just some kind of interest of mine, based on some of my predispositions. That is, I was always good at writing texts, in principle, my language was so good, well, I understood that this is what I am passionate about. Here. M. That is, but it was as if it was a choice from the opposite, that if not a psychologist, then I would be a journalist. L. Well, I wouldn’t say that this is probably a choice from the contrary, that is, well, my interest in psychology never went away there, my dad always gave me some books to read, he conducted all sorts of tests with me, that’s it. It’s just, well, maybe I’m also partly burned out, because, well, I’m basically the kind of person who, if I really want something, I’ll hurt myself, but I’ll achieve it. Well, that means it wasn’t such a really great goal, because if this goal had been great, well, I would have learned biology. Well, what can you do? (laughter) M. Why did you want to become a psychologist? Because dad was a psychologist or is it somehow different? L. No, I actually found out that my dad is a psychologist after I decided to become one. But this, you know, is such a story that you never know what your parents do, and then you find out. Here. Well, in my opinion, it all started because of the series “Lie to Me”, I was very interested in this topic there, like body language here (npt) that’s all, it was so cool, then I read that There are sports psychologists, I thought that was also interesting. I wanted to be a sports psychologist. And then I began to get interested in serial killers, just reading their biographies, and I wanted to be a psychologist who works with all sorts of criminals, that is, he understands why they commit such terrible things. Well, maybe I also watched enough of some TV series there, like “The Crypt”, where I remember going to the dacha, watching with my grandmothers in the evenings, where a criminal is also sitting there, and a psychologist is working with him. Here. Well, somehow it attracted me because it was such a very socially significant profession, which, in general, was aimed at a good cause. M. At what age did you decide to become a psychologist? L. Oh, well, it seems to me when I was maybe 10-11 years old, that is, before that I wanted to be an actress, well, let’s say, many girls in elementary school want to, and then I think - no, it’s too fickle, a psychologist - that’s the most That.
Well, not a profession, but probably just a hobby, I just understand that in fact all my energy is there, and when I step over myself, well, I forget to do this creativity at all because I have no time, but I begin to suffer from it. Sometimes I have some kind of blues there, I can even get sick, psychosomatics starts to turn on. Well, in general, these are the moments. M. Katya, tell me, have

you ever had a consultation with a psychotherapist? K. Yes, by the way, it took place at the Higher School of Economics.
M. Yes, but where, as it seems, maybe you or your peers can get information about what independence is and what an independent person is? M1. Well, probably more from... From psychology, but from some kind of popular psychology, because it is more accessible and understandable. And very popular now.
M. I see. Great. Do you have any hobbies or hobbies now, do you work in the summer or in general? N. Well, I worked, but I worked in Yandex, in Yandex.Textbook, in an educational project, then I left there because it was... I worked in support, and in computer science in Yandex.Textbook, Well, in computer science, I left there because I had family circumstances there, and I had to write a diploma and everything else fell in, well, just because of stress and plus the manager changed there, so it didn't suit me and I left. So, I was in Moscow as a psychological internship in my fourth year, I did it for a charity foundation in "Shalash", so, I don't know whether you know or not? M.
M. Well, about this and that. N. Well, in general, I'm interested in the sphere of parent-child relationships and the sphere... Well, in short, working with children, with teenagers, as if I, which in my experience was not very clear, seems to understand what to do as a teenager, so I kind of do everything... Well, I just like this age, that the worldview is formed there, and so on, and so I kind of want... I would like, for example, to have such a person, to To whom I would come and somehow tell everything without judgment, for example, he or she would simply listen to me. Well, in general, I would like, for example, to have such a person, if anything, like a school psychologist, so. At my school everything was like that, well, very like that, not humane, that's why... Well, in particular, that's why I went to the psychology department. And in general, I want to be more careful with children and teenagers, so that they can be treated, and, well, treat them in general. That's why it's like this.
And there's this very thing, I don't know when there will be grandchildren, when I'll get married, that is, there's this very insistence on a certain way of life, there are some expectations from the parents, from society, and I feel them, but at the same time I kind of went through psychology and psychotherapy, that is, it all seems to help me, somehow my beliefs, to live according to my own beliefs, and not social ones. But I still feel like it's frowned upon. And for example, when you don't listen to someone, it's condemned. There, when you are, as it were, in the minority, this is also condemned, that is, it seems even to me now, this situation, which is the war with Ukraine, that, as it were (.) some people who are vehemently opposed, or something like- then, in general, some people stand out from the masses, they are always, well, they are always perceived negatively, it seems to me. Here. And when, for example, you are different, it's just somehow scary as if people can be different, and this seems to me to be such a problem that it happens because of this condemnation that you are somehow different. Here.
M. It turns out that the Department of Psychology helped me to be more independent, if I heard correctly? N. Yes, it seems to me that yes, and it seems to me that it's just plus the people there, my friends and my husband, it's like, that is, we somehow switched to a more healthy (npt), healthier kind of then the atmosphere, somewhere there it is normal to experience some emotions, well, emotions such as anger or hatred, or... Well, in short, that is, which previously seemed to me that they should not be experienced, or that it is bad to experience them, or something else. Well, in short, there is no plus about family (npt) there (npt), that these are all sorts of non-adaptive patterns and so on and so forth, but how very upset I would have been at first when I took this subject, that this is how bad everything is and all that, but then I thought that, in general, this happens not only in my family, it happens even much worse than in my family, for example, and that parents, they just seem to try as hard as they can, that is, they seem to they also draw conclusions for themselves,

based on those who raised them, based on life experience, and what they learned is what they passed on to us, so, and we can then also take it out and pass it on, and so on it will always be like this, and therefore it seems to me that this is, in general, a positive dynamic, that it is, in principle, enough, and not what you say there - this is bad, this is not the same, this is not adaptive, this is something else... That.
And then, and also the fact that I was probably not afraid to take specialized mathematics, because to become a psychologist you need specialized mathematics, and it was like, it was very difficult for me, and every time it ended... Andrey, my husband, he seemed very He’s good at math, but he tried to explain it to me, I didn’t understand, it ended in tears every time and all these tutors and all that, I didn’t like it at all and I literally forced myself to learn it. But I’m like, I want to take psychology, I’ll pass and I’ll never need it again. Well, how important it was for me to pass it well and then I passed it, and I, I had such, well, it seems to me that such a low score for admission, but then I was very... At first I was upset, then I was very I was happy because it was an entrance exam for psychology and that I was going to get into it after all. And I was very happy about this, and my decision, and the fact that I passed these points, and my decision to go to the open day and to enter the Higher School of Economics, although I did not see other universities, that is, I did not go there and didn’t scout anything. I sort of decided that I needed to go there.
N. I would rate it a 10, to be honest, because my parents, my dad, were very much against me going to the psychology department, he wanted me to go to law school, I have a mother, her first education was in medicine, then she studied in psychology. went, but she was like, well, it’s like I want to be like her, she thought, here. But I didn’t go there because of her, I was just interested, as if about the people, there, like with the people, what’s in their heads, how to understand it all, that is, it was somehow for me it was interesting about behavior and emotions, so I kind of... And so my mother says something to me: go to these courses, go to these courses, there’s something else, and I say no, I won’t go, I’ll decide for myself what courses I should take, or my dad says, I need to go to a master’s program somewhere to become a lawyer, or something like that, and I say like, no, I don’t want to, or whatever Here I am this year, well, in the next academic year, for example, I won’t study, because, well, like my parents, they would rather have me go to a master’s program, but I just understand that I’m tired of studying (laughter) that I can’t, (laughter) I need rest. (laughter) And that I just don’t understand where, what kind of master’s program I would like to go to, I didn’t understand, and therefore I didn’t go anywhere, and how would I make this decision for myself, how would I want to work and understand maybe there's a better place for me to go. About the job, I would probably rate it a seven, because my first job was in my dad’s sales office, that is, my dad, he owns a construction company, and I worked in his sales office. But I wanted to work myself, as if I wanted it myself. And I kind of came there every day, stayed there for some time, they already told me like - go home, but I was like - no, I still need to do this, and I tried to come up with something there, whatever didn't come up with it. Here.
M. What about making everyday and important decisions? Here, in fact, we are, of course, slightly duplicating what we have already discussed. N. Yes, yes, I understood. (..) Well, I think, it seems to me, let it also be seven, because I, well, in general, it seems to me that this is a process that there are no correct ones... In general, I’m kind of really worried about there right, wrong there, or how it will affect others, or something like that... Well, in short, I worry about this in every possible way, and it seems that the more I work there with a psychologist, well, the more I see, that no matter how people react normally to my decisions, the more calm I am about this, the calmer I begin to feel about it, so how would I consider that it’s seven, there I’ll just be there later, well, what’s my attitude specifically towards making a decision , it’s very similar, it’s quite saturated with anxiety, so I wouldn’t say that it comes too easily to me, but I kind of understand that I always make

adequate decisions, plus or minus, I just have problems making decisions, that’s it. And the choice of lifestyle, leisure, well, right now I would probably say that (..) six, because I think that (..) well, I just feel that I’m a little burnt out and tired after studying there, after school, when things weren’t very good there either, at the university, when there were some difficult moments there, that’s it. And it seems to me that I just need to recover now, just somehow settle down, that sometimes I get shaky, like how I behave there, or how I need to do or something else, well, like a lifestyle, and for me, I’m just now focusing on the fact that I need to rest, and otherwise be more careful with myself. Here. And about leisure, I don’t really like spending time in company, I rather like one-on-one communication, and well, I kind of, (.) well, I don’t do any supernatural leisure, like, for example, I knit there, or maybe I have some kind of hobby that is very accessible, well, drums are not very accessible. That is, I disturb everyone, and it happens that they irritate everyone, but as if I could occupy myself with something, I don’t feel like I’m very bored, that is, in general, in every possible way... I sometimes scold myself myself for rest, that is, it’s as if I can’t rest, because I’m not tired and all that. But this is again a balance, there is a work life balance and therefore... (...) Well, about leisure, I meet with friends, I read books there, watch films, some educational things there, well, educational videos on various there platforms. Well, in general, I do, it seems to me, what I want, what interests me, and it seems to me that this is enough. Here. Romantic relationship. (laughter)
M. What do you mean, what didn’t coincide? I didn’t quite understand? They said that it was possible, but... N. No, rather that they... Well, for example, dad said that you should, for example, not lie, that it was better to be honest, and then it turns out that he has a second family there, that he him there... Well, in short, (laughter) some secrets from us, roughly speaking, that is, that he says one thing and does another. And that is, as it were... It doesn’t work out, then there is no clear picture, you still understand that there is this duality, and not... (..) Well, what is it... And you begin to think like about some of your actions, that’s it. That’s what I’m talking about, that there weren’t any where, for example, they told me - go and do it, for example, my mother said - go there, do something, take an interest there, something else, if only I had such opportunities as you have, then I would have done this, that, that, but she, despite the fact that she seemed to have opportunities, she practically sat at home with us all the time, that is, she developed, some She worked through her own problems there, with the help of psychology she studied there, but she didn’t seem to really realize herself and it was also like this was a call, like go do something there, realize yourself, but she herself didn’t do that. And it was as if I was expecting that if my parents said something, then they would do it, but that didn’t happen, and so I had a question, like, how can this be, like, people say one thing, but do another, And I had such a conflict. Here.
M. Do you have any hobbies? Besides work. A. Well, probably yes, part of it is the field of education, psychology, everything connected with tutoring, with... (...) Drawing, exhibitions, studying, well, I don’t know, the work of artists, this is also connected with literature, Let’s say now, the main focus of interests is the book (npt), Annie Lee Lang, which describes art, artists, and, in principle, the concept of loneliness. And how artists reflected it, how it is depicted in culture, in what images. Here. Something like that. M. Great.
A. So, training and professional development, I already said that. Health choices. If we talk about the second point, then for the last year, but probably I have had more changes in six months, because there was a change of place of residence and somehow I concentrate more on it. Because the choices that were made in the previous six months were not in favor of my health, but there the only important option, or rather there was a choice, was to go to a psychologist. To deal with this destructive story with the leader, with (sigh) (..) change of position. Here. And if you look more towards the physical rather than the psychological, then this is probably a choice in favor of taking care (.) of some kind about yourself, to searching for, well, understanding in general your health

there through the body, changing a little nutrition, attitude towards sports, and so on Further. Well, something like that, I returned there again, to proactive history. Job. (..) One important thing, well, it seemed to happen to itself, it so happened that for a new position, again over the last six months, first there was the building of horizontal communication, despite the fact that there is a manager and subordinates. Yes, I am the director there, but these are still subordinates, a turquoise organization was built. There was this, well, how it was clearly articulated, said internally in the collective, in the team.

M. Basically from the elections. A. Well, after all, what is still very much unclear and difficult for me is the case of accepting responsibility for the whole, the position of a manager. M. Yes, I remember you talked about this in great detail. Yeah, that's what happened. A. This is really very complicated... Well, it seems to have been partially worked out, this situation, in psychotherapy, but not yet fully resolved on some emotional level, it also seems to me that this has created some kind of trauma, (laughter) here. In terms of what was wrong, why was it wrong, right? M. Yes, why the wrong choice, do you think? A. Because, again, it was probably from the position of - well, who else? Well, very often I have such a choice when there is no other outcome, well, that is, some kind of critical point. And only then do I, as it were, completely regroup, into some kind of monster, make this decision, well, that is, I say this - yes, I can, we'll do it this way, I'll pull it off, but somehow, as if at a critical peak, I take upon myself the whole responsibility that is possible. Well, as if this were directly noticed, and then it usually works out. (xxx) But in this situation, which was and where my choice was, as it were, wrong, I underestimated it. Well, that is, the gap in this critical situation, the gap between what I can do and what definitely won't work out, it, well, happened more. Revaluation. (..) Here. M. Yes, but how do you feel about this situation now? What thoughts or feelings might arise about her? A. Well, it's cool that it was the first thing. Second, an important decision was made that in the next couple of years, I don't know, I won't take a management position at gunpoint. Not yet, well, no, I won't go there again for now. Just now I started, well, I kind of started to sniff out, if possible, from this state when you, well, got a very strong electric shock, well, you just intuitively won't get into it anymore. Here.

Another important choice, which in general, if in the future 10 years, then (..) it's probably closer to the family. Well, that is, as it were, a choice in favor of (..) building long-term relationships, and another related choice - health. Well, that is, as if I don't want to waste what I already have, because our body is only depleted, but I want to somehow fill it up, maintain balance, different areas of life that affect health, ultimately well-being, and the feeling of happiness when you feel good. This is a choice in favor of, well, taking care of yourself in different areas. Well, that is, starting there, I don't know, with some kind of tests, check-ups, regular support for physical fitness, psychological health, such a minimum of some kind of psychological hygiene, then yes, this is directly connected for me with family relationships, because in Ultimately, if you make a choice, then for now I have the feeling that I am not childfree, but I still want some kind of (.) continuation of the family and continuation of the clan, if you can call it that. Again, I want healthy children, a healthy child, and this means that I must be healthy. And the other person must be healthy. Therefore, this is a long-term construction, with certain resulting requirements for a person. (..) And probably not by requirements at all, but by similarity of beliefs. When they look in one direction. Well, probably for 10 years, well, another important one, this is first, in the short term, somewhere up to 5 years, a period of time to take, then this is a set of expertise and experience, packaging it and going out not to work for someone, but for myself.

M. You can go straight through the list, you don't have to arrange them. K. Training and professional development is... Probably there will be seven, because (..) you always have to learn everything, and sometimes it's very difficult to allocate your resources and brains, (laughter) let's

put it this way. And do one thing. Health is five, (laughter) it's either there or it's not, work is also six or seven, because... M. Wait, let's go back to health, making specific decisions. In terms of health. K. Regarding my health, everything seems to be fine, I feel good, but sometimes there are times when I get sick, (laughter) Covid and so on. And I try to recover as much as possible. But if I get sick, then I don't do anything, it's immediately minus work, (laughter) minus friendships, only the place of residence will be there then. (laughter) At maximum. M. What about choices, such as choosing which doctor you need to go to, or even understanding whether you need to seek help or not? K. Choosing a doctor, I don't know. I somehow... M. How difficult or easy is it for you? K. No, it's easy for me to choose a doctor. I just see, either from reviews, or using word of mouth, that this doctor, we went to see him, he's good. I'm so good, I'll go to him too and everything will be fine. In principle, this is how it turns out, so I don't have such a difficult choice here to choose a specific doctor. Well, usually, I just probably didn't get sick with anything serious, no matter where it depended on me... The doctor's choice is between life and death. Therefore... M. In general, you said that you rate your health as a five, then what is the difficulty in making decisions regarding health? K. Health, difficulty making decisions, (laughter) giving injections. This is where it's difficult. (laughter) Or force yourself to go... It's just that when you're sick, you don't really want to go out. M. So it's difficult for you to understand whether you need help or not? K. Yes, I can't, it's sometimes difficult to assess the extent of my illness, it just seems to me that even with a temperature of 38 you can still do something there and at work, everything is a doctor (NPT) in principle (NPT) and you can live.
M. And now I will ask you to tell me about one story of a successful choice, and also, please, in accordance with this layout from the chat. K. Successful... (..) Now, we need to think. (...) Something... (...) I thought, I think it's good that I changed one job for another. Before that, I worked in another place, not even at a university, but in a clinic, well, in a hospital. And this is very, well, at first it seemed to me that this is very cool and great, because the equipment is new, but then I realized that this does not give me any career growth and if I stay in this position, I'll be there (npt). Then, but this also happened at school, then they gave me... Work, a feeling of extreme fatigue, every day. And it's like (npt) Groundhog Day. And I realized that this had to stop somehow, otherwise I would simply burn out at this job. M. So you also seemed to be guided by your emotions? K. (..) Yes. M. It seems to me that you have someone else there? K. Yes, now there are students again, we should have opened another classroom for them. All. (..) Well, it seems like I told you everything. M. No, more about feelings and thoughts? K. Oh, (sigh) feelings, then this situation, (laughter) it seems to me that this whole situation sucked out any emotions and feelings from me at that moment, because working in a hospital is very energy-intensive. And now I'm looking at it too, the same thing, the only thing I've gained is experience, put it in my work book and just to fill out my resume. M. Tell me, Karina, how do you determine which choice is successful for you and which is unsuccessful? How do you separate them? K. (..) Successful, if I feel some kind of return, I feel needed. And unsuccessfully, if I feel some kind of return, I feel needed. And it's unsuccessful if I feel like I'm wasting time and wasting it.
want to be the kind of person who (..) takes responsibility for all the negative events that happen to him, and for example (..) does not ask, well, that is, it's normal to ask for help, but when you really need it, that's it. I (npt) did this, and I started to practice it now, and if I feel bad, I don't call right away, but before I immediately picked up the phone, immediately dialed and cried. (laughter) And now, first I'll cry to myself, listen to music, maybe talk with friends about some abstract topics, and then, when I've calmed down, I'll share. Like this.
M. But you also said that mom changed a little, softened her views. What does this have to do with? L. Oh, (laughter) with her life experience, probably. Well, oh, in general, yes... (..) Well, she looks, reads these psychological books, and something else. In general, it develops itself, so we

plant it like that. (...) Because she doesn’t like her life, she wants to change something in it. M. Lisa, was it really noticeable to you that suddenly she changed there and became softer? Or how? L. Yes, yes, she just talks a lot, and since she has no friends, in our city, well, they were, they all went to Tyumen. (laughter) And a new friend appeared, and she also left for Tyumen, (npt) And so it always turned out that she was talking to me and all these topics, well, I don’t know, she talks a lot in general, as if about herself, then about how she lived there (sigh) then she speaks about herself, about her changes. Or it says that you can reprimand me there if you see that I’m going too far there. Well, in general it was not difficult for her. (laughter)
..) Rather, this is how it is, somehow significant, this is health. (...) So, health. (laughter) (...) I would probably classify it more for me as some kind of sport, some kind of sport, (..) they are connected. And, (npt) I also said that alcohol, (laughter) (npt) let’s say, refuse (npt). That is, I don’t want this one (npt) yet, but even before that, this is the last one. And as for sports, I, I have a new kind of sport, that is, always, (NPT) development, I liked it, there was a subscription for eight classes. This is something new for me. But as they say, well, jumping on a trampoline, doing (npt) or something like that, in my opinion. (npt) Then work. Work, well, work, here I chose, “Yandex.Food”. I was choosing between Yandex.Food and Delivery Club, well, it seemed that in Yandex.Food, well, how to work there, on the contrary (npt), with large orders, but since I could, I had initial information... M. So, now everything is turned on, now, I hope, everything will be heard well and there will be no interruptions. L. Well, yes, I said that I was choosing between Delivery Club and Yandex.Food, and there was more difference, that is, I went to Yandex.Food. Oh, and then I also worked at Pyaterochka, but I initially went to the order area, since it turned out to be there from morning to evening, and I needed (..) evening part-time work, or for a number of hours. But, I said, they offered me a cashier job, and I, I had a choice... M. Yes, Lisa, something went wrong. Yes, about the order picker, and you were offered cashiers. Here in these words. L. Yes, I had a choice whether to agree or refuse. And I thought, okay, I’ll agree, I’ll try, why not. Although it was a little scary and exciting, in the end everything, so to speak, worked out, I was trained and I worked calmly, so to speak. Here. Later, but then I had to leave, so I quit. But I warned that I wouldn’t be around for long and, in principle, (laughter) since they didn’t quite suit me, (laughter) and it would have been a choice in general, (laughter) probably to quit. And, so, (npt) in my opinion, (..) I would roughly give an example, like choosing a vacation. Since it’s difficult for me to get from Rostov to Nizhnevartovsk, so to speak, at the moment, since the airport is closed, and you can get there by roundabout routes, but it’s expensive. Because from Moscow (laughter) to us one way it’s 10 thousand, but I just had a direct flight, I could fly (npt) when I was lucky in principle to get it and when you already flew for 3, and for 5, and for 7, then you don’t want this, well, yes, you don’t want to go one way for 10. But in principle, it happens, it’s a lot, so to speak, well, I would come to my city, but there’s not much there, there is nothing to do, and in general, my mother and I agreed that we would meet in the summer, but not in the city, but somewhere, so to speak, for us on neutral territory. Well, we went to Sochi, that is, she (npt) flew, and I had to get there by train, and... (...) Well, we generally chose tickets, well, where is the best place to go, we chose these ones, where Sochi is. But since I was already there, a year ago, for a few days, it was somehow very, there is such an example, but... (sigh) So, (..) friendly relations. (...) Well, choice. (..) Well, here, probably, who would you like to be a friend, (laughter) and who would not. Don't know. Or, let’s say, where there is a person like this, I would like someone... (sigh) (...) So... (laughter) In general, they offered me to take a walk there, and I, (..) let’s say, I didn't want it in general. I don’t like this person, so I don’t want him to be (laughter) my friend. And you probably... Well, I kind of refuse. Don't want. Don't know. (laughter) What’s a better example to give, honestly. Well, I don’t know how I could have one friend here, (..) I don’t even know. I'm

moving on for now. Place of residence, regarding the place of residence, by the way, I live in a dormitory, and initially I didn't want to live in a dormitory, because, well, it seems to me that here, well, all sorts of events are held, as if there was a place to sit, let's say I had to film apartment or something like that. And plus I have a hostel, it's very comfortable, well, I just won a competition, (laughter) and the hostel in which I live, it's like, well, it's an apartment type, and that's why (laughter) it's here for me in general, not only that , that I'm like this in a hostel, where I have connections, but it's also almost home. (laughter) But, of course, I didn't know which one I would end up in, but initially I wanted to go to the hostel, so that there would be all sorts of these events in which I could participate. That is, renting in one there, in an apartment, is not interesting, it is boring. Here. (...) Everyday and important decisions. (sigh) (..) Well, about important decisions, everyday ones, probably just like where, where to go, if everyday, where to go, to have fun, to see. Well, also, for example, I took part in the race there in May, for me it was such an important decision. In such a major event, because it is not held here, and I have never participated. The Russian Federation race, which took place in May, I... Well, it costs money, and you kind of have to decide on it. (laughter) I took the plunge, bought it and didn't regret it. And also (..) I liked it, I finished the race (npt) I also decided, I realized that I liked it, that (laughter) I need to train for it, and that, well, more.
M. If in terms of the degree of difficulty of the choice, how would you rate it between one and ten? Well, you actually talked about a series of elections, but on average, how do you evaluate your passage of these forks? You chose between a bachelor's degree and a bachelor's degree, this advanced one, you chose not to work there, but you chose to work there. J. It seems to me, seven, approximately. M. Thank you. J. Health, I don't even know what I can say about this. Because I've always tried somehow... (..) Well, I just have very good health, some kind of good immunity, I've never really... In terms of paying some attention to my health, I make a choice in favor of some kind of regular check-up. I go to doctors, donate blood, take vitamins, and exercise. I'm trying. Sometimes I get very lazy, but I'm trying to instill this habit in myself; in fact, I still really want it to be some kind of regular. Also, I go to the doctor, well, that is, I don't have the position where I will never go to the doctor in my life, like some people, they don't like it at all, no, I'm somehow very sensitive to myself, to my health. It's better to go get checked there, once again, than not. M. And how would you rate the severity of choosing these, developing habits, even laziness, but it's important to go to the doctors, get checked, get tested, how easy or difficult is this for you? G. Four. M. Four yeah. Thank you. J. (..) And work, for me it's a little bit like that, for now... (..) Well, I'm still studying there in general, what suits me, what doesn't, I don't understand yet, I generally... At the fact that it seemed to me that I was a rather diligent person and that I could somehow do some monotonous work for a long time, but it turned out that not as long as I thought. And I'm still feeling out in general what type of activity I'm comfortable doing for a long time, because somehow, well, in my head it's as if there should be one activity for, well, a long period of time. But I always somehow get tired after a couple of months, I need some kind of change of activity, or some kind of development. That is, right in one place, something is the same, like accounting, for example, or in a bakery, doing something the same every day, I start to get tired and fall into some kind of despondency, which is like this it will happen, I immediately have some thoughts that it will be like this all my life. It's the same there every day. That is, I also realized for myself that in this regard I need some kind of variety, and I want... Well, my dad, for example, never worked in an office, maybe that's why I subconsciously somehow chose a profession similar to his . Because he travels around objects all the time, doing something, something like this is closer to me, some kind of active activity. Even in terms of studying, I could never sit like this and constantly listen to something and that's it, that is, when we receive knowledge, we immediately put it into practice, some kind of more creative training or something. And apparently I'm inclined

to have a job, I also need some kind of work, well, more active or creative, I don’t know. I'm still searching. Choosing a job is actually quite easy for me, I don’t know, maybe (..) 6... I somehow start easily, finish easily. (...) Well, six, probably not, let's give five. Let's put it at five.
J. Because it’s such a difficult topic, because I still... (..) Well, that is, I still live, for example, with my mom and dad, I communicate every day, I’m still building some kind of relationship with them. I myself go to a psychologist, somehow I’m already trying, from the side of some more... Well, not a teenager anymore, but some more adult person, I’m trying to look at any situations from both sides. Both from my position and from the position of my parents. Before that, at school, I always had some kind of very blaming position. That is, I thought that the way I think is exactly like that, and I never really wanted to consider any other points of view on this situation. Not how I see it from my side, but how other people could see it. Well, my parents. And that’s why it’s always been very difficult for me to understand them. And I was like that, a rebellious child inside, that is, on the outside I was quite calm, but I always disagreed with them, with something, I always somehow kept my opinion about you. And now I’m just trying to take it easier, it’s easier to accept that someone may have a different opinion and that’s normal. Other views on some situations. I don’t know, I don’t understand at all what choice means in terms of relationships with parents, but (..) in any case, it was always somehow very difficult for me, I... We always had good and bad, then good again. It was such a thorny road.
M. I think that this decision is also very difficult, and somehow very worthy. But you speak warmly about your father, and about your similarity with him, in principle, in part. J. Well, yes. Well, I went, as soon as I had the opportunity, I immediately went to a psychologist. And I still go to him. We have a free psychologist at our faculty. They did it for us. This turned out to be a very popular service. A lot of guys go. And this is really very cool. Yes, I’m working on my relationship with both my dad and my mom, how it all affected me, and where in this whole situation I am, and where are my parents’ opinions about me, (npt).
Somehow it’s always been (...) hard for me to open up. Yes, it’s probably some kind of, well, childhood trauma. I’m still working with this, with a psychologist, and it’s hard for me to open up to people, because it’s as if... (...) It’s as if, when I open up, I start to take everything too close to my heart, and then it’s very difficult I'm experiencing this. Therefore, in terms of friendly relations, choosing some new friend for me, well, probably an eight.
Now, I have some kind of frantic energy, I want to meet someone all the time, learn something new, go somewhere new, to theaters, museums, concerts, walks, etc. whatever. I feel like I'm being filled up by this, I don't know. So I went, when I went to a psychologist, somehow after that I became more open, and then people themselves began to reach out, after that. (..) Oh, romantic relationships, that’s also a ten. I have a very hard time with this, because it’s also about opening up, you have to open up to people, and at some point I start to quickly, quickly close myself off, as if when I understand that this is some kind of... Well, it’s not like that anymore, (..) when I realize that I am becoming a little vulnerable, I, (..) my brain seems to automatically begin to push away, that is, it is so much so that I even (..) at first may not notice it, and only when I analyze it later, that is, everything is so quirky that it seems to me at the moment when this happens that the reason that I chose is really important, but in fact then I understand that I just had some kind of fear and (..) I just, well, sort of decided to run away. Now I’m actively working with this, but this one is hard. For ten.
K. Metaphorical associative cards are a tool for a psychologist so that a person associates his situations with the help of pictures. That is, it’s easier to sort of reveal it all, from different sides, from different points of view.

M. Did you decide to do this yourself, or... How did this idea come about to do this? K. So I was on an educational shift here, at the Republican Center. And just like that, the psychologist worked with us, and she talked about these cards, which are associative psychological. I bought them because I was interested in all this, I bought them and they are generally so beautiful, (laughter) and I began to sort of rummage within myself, with the help of them, and then I realized that I was more interested in going deeper into all of this. climb, and this just became popular, these Tarot cards and so on.
Well, firstly, I didn't want to lie, I never had a need to lie or hide something, and (.) secondly, it's always obvious from me if I'm lying. Since I apparently didn't learn this, my mother always knew that something was wrong, even if I kept silent about something. She says - so, do you want to tell me something? She always (xxxx) saw and read me. Right always. Well, at the age of 12 I had a rebellion, at 13, maybe it lasted until I was 15, and then it started that I don't want to tell my mother this, she will scold me there or she forbade me to do this How can I tell her about this? After this, this situation, which lasted maybe a year or a year and a half, with that boy, no more situations like this were repeated, that is, this was enough for me, I completely lost all her trust, it didn't suit me, I stopped feeling this connection , so that I needed it, that is, she stopped believing me, stopped supporting me, it became hard for me. And this was not a reason for me to stop communicating with him, I just matured a little and realized that he really was something strange. What did I even find in him? (laughter) Here. And then I realized that I still regretted what I did. And from that moment on, I no longer deceive in any way. (.) And that is, my mother and I discussed this, I say, like it happens, my girlfriends drink, and she says - okay, like if they drink, then wait a little, from 16 I will officially allow you, come on, (. .) whatever... (..) Only you will warn me, write and, as it were, you must come home, you don't have to go anywhere, and so on. Well, okay, that is, there were no secrets from my mother at all.
but initiative can be punished, because (.) I studied excellently, I had to, well, I wanted to participate in various competitions, Olympics and others, but I simply didn't have enough time in the day, I didn't have time to be good everywhere. And sometimes I felt bad because of this, I had periods, I don't know, now you can call it burnout, but I just felt bad, I was starting to get sick, that is, apparently this is how my body reacts, I don't pay attention to tired, but I pay attention when I start to get sick. And I just went into some kind of illness like the flu or something else, I might get dizzy, I would faint and bleed. These are the stories I have, but I just don't notice it. That is, this can affect your health, this initiative, because you are ready to be everywhere, but there is not enough time and energy.
M. But if we talk about, for example, a dance studio, a school and then a university, there, inside these institutions, they somehow tried to cultivate your independence? A. (.) At the dances... (.) We developed... We had such a strict teacher, I studied mostly classical dances, and training was constant, and there the teacher developed for us... (.) Probably character. That is... But I can say that it's probably... (.) Well, yes, on the one hand, I'm very disciplined, that is, in this regard, dancing gave me discipline just 10 out of 10, that's because there was such a teacher. And there was a certain atmosphere there, but (xx) it destroyed other traits of my character in me, that is, I could not develop as a person, because there, for example, there was criticism all the time, it was squeezing, that is, constant pressure. (.) So we also had a group, there were only girls, it's very difficult, especially when you're 14-15 years old, and everyone somehow had slightly different interests, well, in general... And I didn't feel like there in some cool company in which I feel good, so on the one hand I really loved it, but now I already understand, looking at this, that yes, the discipline turned out great for me, of course, but there are certain mental , I still have psychological traumas.

D. And I, you know... Yes, I have friends. I have very close girlfriends, and my boyfriends are also close. I also decided to take care of my mental health, since I can’t go to a psychologist, since we have one free psychologist in the city, and I went, I didn’t like it, because it was at the level of simply abstracting from problems. Here. That is, sometimes it helps, yes, so as not to overextend yourself, but sometimes not. Here. That’s why my friend gave me a book for New Year... (..) “The child in you must find a home.” This is some German psychologist, Stephanie Stahl, here. There are so many exercises, why we do this, that is, in general, it all comes from childhood, and inside us there is a child who... Ah, a gloomy child and a sunny child. And all our tantrums, or when we get angry, it’s all a gloomy child, that’s it. And we need to find a common language with him, and we also have an inner adult who sometimes succumbs to the whims of this gloomy child. Here. And there are a lot of interesting exercises there. I try to do this sometimes.
M. Yes, in general, undoubtedly. Are your relationships with your parents too distant, distant? Or somehow you quarrel, or... D. Sometimes they are very close, and most often these are just situations when we become close, which is extremely rare now, they make me feel guilty that I’m so bad and I offend them. But in general, well, I don’t know, they certainly didn’t do much harm to me, but purely morally, they did. That is, there is some kind of financial support at all. (..) But psychologically, of course, it’s very (.) difficult. (..) Here. Varya, I'm a little busy. They are shorter in the kitchen. OK. In short... (..) So what should I tell you? M. I asked about relationships, about support or about conflicts, well, a little, in general terms, in order to somehow understand. D. My father drinks often, and as if before it didn’t directly affect him very much, it was limited to the fact that we were walking along the streets, he was drunk, and just did all sorts of bullshit, I was ashamed of him. And then, in the ninth grade, when I began preparing for the Olympics, I studied them seriously for the first time, I almost didn’t leave the room, because I wanted to seriously prepare. And my parents said that you sit so much and seem to be moving away from us, well, they fought because of this. And my dad also came home drunk, and my mother asked me to sit with him in the kitchen and talk every evening, he drove me to hysterics, and in ninth grade I can’t even imagine how I pulled it out, but it was... Emotionally it was difficult . Here. And with my mother, well, I don’t know, in general I’ve always been my father’s daughter, well, I thought so, but for some reason I don’t have a particularly good relationship with my dad now either. Mom sometimes, sometimes she somehow tries, like, when she sees that I’m really really bad, and that’s not always the case. Here. But she doesn't take my problems seriously. Well, yeah, she doesn't take my problems seriously. Yes, they don't take my problems seriously. That is, if I say - I’m tired, I worked, they’re like - why are you tired, what problems can you have at 18 years old? Yes, it’s true that there are none. Here. Well, in short, not being perceived as an adult also depresses me. Here. (...) So, it’s very difficult now all at once, in fact, the relationship with parents is quite complicated, that’s it. And now I’ve simply protected myself from them. When they are at home, I practically don’t leave my room. Now my mother is offended by me, and I gave up, I don’t care what she was offended by, I don’t even want to find out, let her really be offended, if she doesn’t want to enter into dialogue, it’s her problem. Somewhere in the middle of the tenth grade, I tried with her, no, or at the beginning of the tenth grade, I tried to talk and say that I need support, at least just come and say that you’re great, you can handle it, you’ll succeed. And that's it, I don't need anything else. And this dialogue boiled down to the fact that I’m ungrateful, they spend a lot of money on me, they spend almost their last money on me, and I’m ungrateful. Then I got sick of them saying that they were spending a lot of money on me, so in April I went to work. That is, school and work were certainly difficult, but I managed. And since April, I haven’t asked them for any money, I’ve bought clothes and something to eat here, well, I’ve gone to the neighboring town, all with my own money. And then they say something like this to me - why don’t you go to the village with us, like

you’re at work again, you’re cutting yourself off from us. Are we not giving you enough money? Something like that. Well, well, yes, I don’t have enough money. So I went to work. Here. (..) In general, they are (..) children. M. Children? D. I can honestly say, they are not trying to change themselves, they are not trying to hear (..) me, that’s it. It's quite difficult. M. Yes, I’m very sorry, what you’re saying is very sad to listen to. And it’s even so paradoxical, that is, well, it’s clear that you’re trying very hard and learning, and it’s true, a lot of things are working out, and so, when they tell you that you’re studying too much, and maybe you need to stop and go do something else, yes, I think it’s very disappointing to hear. D. Yes, and when I wrote the Olympiad poorly and walked around upset, they told me that I just didn’t study enough. Here. Something like this... M. Well, yes, in such conditions, of course, it is difficult to get support. Apparently we really need to look for it somewhere else. Please tell me, do you like being so independent, do you want to be more independent, or do you want to share this responsibility with someone? D. I began to notice that... Apparently I don’t know at what level this works, but here... (..) somewhere inside I am still a child and in most situations, sometimes stressful, I begin to behave like a child. And then I, well, kind of think about it and... Well, I analyze it, and I understand that (.) did not behave like an adult in this situation. But it was necessary. Here. Well, in short, this independence from childhood, it actually affects me. Because even more so sports, they also tried like professional sports, all the work, and there, well (..) no study, no personal life, only sports. In short, this is it. You also need to be responsible. And now sometimes it takes its toll. Here.
M. I see. Are there some situations that you want to hide, on purpose and in general so that they don’t find out and maybe no one finds out at all? D. (.) Probably self-harm. M. Do you do self-harm? D. (.) Not now, but through sports I can. Come to the gym and just... (..) To the point where I'm just falling. Here. M. And does this help you in any way? D. Well, (.) not to think about something, I don’t know. Just to finish myself off, probably. I don’t know how to come home, go to bed and that’s it. M. It’s a pity, this is of course an inappropriate way, but as you say, it seems to me that you seem to have more different available tools in order to somehow deal with your condition. She showed me the book, told me about the exercises, and told me about her friends. D. Well, I’m trying, I’m trying to somehow get myself out of all this, in fact, I’m trying.
M. Thank you very much. Please tell me, you are in Rostov-on-Don now, right? And I understand that you are working, while we were agreeing, you wrote that you need to catch up after work, are you studying or just working, how is your life in general now? L. Yes, then I will answer in such a multi-layered way, I received my first education this year, a bachelor’s degree, at DSTU University. In the Don State. Here. That's where I work. Here. It’s like we’ve even gone a little towards individualization, here. I’m studying, I’ve now entered two master’s programs, the first is my 39th, this is social work as well, and the second, 44/2, is in psychology and pedagogy. M. Ah, colleague that is. It's clear. Is the bachelor's degree also in psychology? L. Bachelor's degree 39, this is social work. M. Social work. L. Yes. I became interested in psychology in the second year, in the third, the therapeutic direction was closer, I studied there and realized that I also wanted to take two master’s degrees and one of them first. M. So you will study in both at once? L. Yes. That's what I thought... M. Wow. And work a little more? L. Yes, and in different universities. Well, that is, in my 35th, also in DSTU, and I went to study psychology at the Southern Federal. M. So, the Southern Federal is Krasnodar? L. No, this is the Southern Federal District, this is also Rostov. M. Oh, I got it mixed up, yes. It's clear. Do you have any other hobbies or interests, if you have time for them? L. From a hobby, if it’s an activity, yes, I really like to organize and come up with all sorts of different thematic events, fortunately, the university also loves when it is offered to do something, I probably won’t name any right now, psychological hobbies, hobbies, but for me I like some practices, well, for which I am conditionally admitted, I mean going through, conducting training, of course that’s it. I wouldn’t call my work a hobby, although I enjoy it.

M. Can you think of, remember some example, from there, I don’t know, from a movie, from a TV series, for example, I don’t know, from another work, from a book, of a person who is independent and not independent? Maybe some celebrities, someone famous, or some bloggers, someone in plain sight? L. So, (...) I don’t know directly whether this can be such an example, that is, interpret... (..) And in the work, well, Bukowski, there is Henry Chanaski, as his alter ego, the work “ Women" is called, "Post Office" too, so, I remember the main character, in my opinion he is very infantile, he also lives the life that he wanted there, he is rather an example of this, he works there at the post office, with a huge number of sexual relations, beer, and it’s as if he has nothing else in life. He didn’t escape anywhere, and he didn’t have to do anything. And if he’s just so independent... (...) What’s his name, I watched some interview today, I don’t know, I remember, Mark Guarden, I think his name is. Something like that. March... Some psychologist, I listened to his podcast today. Some kind of person, I don’t know... (...) I can give examples, so I look, I don’t know, the rector at my university is an independent person, but I don’t know (npt) work or not.

M. Did something happen then? L. Yes, yes, yes. And there... She fixed herself in this position. M. Well, somehow you can ask the question - what? Or don't want to answer you? L. It’s possible, it’s possible. I worked through my injuries, you can ask. (laughter) In my family, well, like in a family, there was a stepfather, a case of domestic violence that lasted for 6 years, from 10 to 16. Here. And it was very difficult to fight back, I was little, then I grew a little, so. And at the final point, well, at the age of 16, I immediately understood that I had to do something, somehow fight for myself, well, yes. And, thank God, it worked out, as they say. Yes. And at that moment I immediately realized that oh, oh, oh how you have to be independent (xxxx). (laughter) Oh, oh, oh, how necessary this is. And right then it was clearly formulated and it didn’t go back from that point, thank God. M. But you moved after 3 years, as far as I understand? L. Have you moved? M. No? L. But no, just, well, it turns out that I have my dad, wonderful, beloved, but we (npt) when I was 7. Then I had a stepfather, from 10 to 16, not a very good person, (laughter) Here. Now my mother is also married, but now I have a wonderful stepfather, I respect him very much, I love him, I appreciate him, he is kind, caring and, well, my dad too. Wonderful person. I don’t know what happened to that person. And I already moved when my mother was... I already had, now it turns out, a current stepfather, I already moved away from them. I didn’t have it there, but everything was there (npt) it all ended just then (..) then, my last trial was at 16 with him. That's all. So we (..) said goodbye and... And I directly cemented my position as an independent lady. M. I’m very sorry about domestic violence, of course, and I’m glad that you and your mother are no longer there. If I hear correctly. L. No. M. And you came up with a plan? L. I’m now... Now I’m (npt) taking pictures of the experience, it was yes, thank you, I might not have been so strong. Who knows.

And after sixteen, yes, the first independent decision I made, I remember, it was on nerves, consciously, I don’t know why, why, it didn’t happen, I lit a cigarette. I came straight away, and I didn’t have a situation where I was wandering around somewhere, in garages, I came openly and said, like this. Here. Naturally, my mother didn’t like this, of course, but due to the traumatic experience there, she somehow turned a blind eye to it then. There she said - I'm sure it will fall off you, but now, okay. Well, we’ll sort of assume that these aren’t some kind of steam locomotives, it’s not necessary. She is the only, it seems to me, the wisest thing that she told me, she says - please, you can, just don’t buy some crap for yourself there, it’s better to let them lie there at home, if you want, take it, like. Well, this is something normal. Because again, she says - what’s the point, well, I would take it away from you, shout at you, so that you would stop smoking? Nothing would change. I would (npt) where is it, well, I would be hiding somewhere. No, and it fell off. I don’t remember, I went there for two years, I worked through the traumatic experience with a psychologist, and one day I just said, no, I don’t need this anymore. I wasn’t so drawn to it when it

was like this - well, you want it, right? Please. How (npt) is it normal, let’s not just somewhere, over there at home on the balcony, you want, well, that’s it, so that I can be calm.
M. That is, after you, I don’t even know how to describe it, when you broke off relations with this man who showed domestic violence, with your stepfather, when he disappeared from the family, as I understand it, or he was imprisoned, I don't know. You had some kind of surge in all sorts of independent decisions and some kind of activity, right? L. Yes, well, yes, but rather it was probably, I don’t even know how to classify it. Well, because yes, then it turns out that for 6 years you seem to live in a social cage anyway, there you don’t think about how you can choose or think about something, but I don’t know how it is conventionally, what jacket to wear today, so that there are no visible bruises. Anyway, this stupid childish trait - I won’t show it, it lived, here. And this was the only thing in which you made (laughter) any independent decisions. Previously, you already go into some kind of, it seems to me, even aggression, in relation to those decisions in which you were limited, and you are already trying to grab everything that you couldn’t take there before. Well, here I need to give credit, again, probably to my mother, but she somehow directed it all correctly, what are these, well, that is, my kind of eccentric, some kind of emotional outbursts, I don’t know, or what - radical decisions, like, I don’t know, smoking the same thing, were not met with aggression on her part, which would make me even more willing to do something against. She manipulated it all so much that you were like, so what. And that’s all there is to it, and that’s okay. Yes, well, thank you, okay. Fine. Fine. Well, then, but I remember, yes, she gave... Well, the start date, oh, now, as if I didn’t tear off my charger. I'll put it in. But before the start of, yes, my first year, it turns out that the first year and everything negative fell away from me.

3.4 Table 4. Subjective health evaluations (physical health)

Citations
Yes, it turns out that after that I trained for three more years, I also went to different competitions, in different cities, I already understood, well, since I refused that chance, that chance, a second one most likely won’t come to me, but I’ve already trained, let’s say So, semi-professionally, I played for our academy, CSKA, but I (.) didn’t dream of going there to some Wimbledon and so on. And one day, when I went to a competition in Taganrog, I was fighting for first place, the last one turned out, the last game was for first place, and I fell on my back, so. I injured my back, then I couldn’t walk at all for a week, I was thinking that it was a fracture, or that it was a bruise. They took a picture and said that it was just a spinal bruise, well, it would go away soon, but after that I already quit tennis, because even after rehabilitation there, about six months, when I returned to tennis, the indicators were completely different and, accordingly, , guys who were plus or minus my age, they continued to increase the pace while I was rehabilitating there and so on. Well, basically, I was, roughly speaking, no longer needed, even in this, at this level of tennis, and I decided to leave.
M. Yes, I think I’ll quit, to be honest, now I’m already trying cigarettes, I don’t smoke, I’ve switched to alternative, let’s say, methods of obtaining nicotine, like IQOS, well, for example, something like that, it’s still unknown of course, which is less harmful, but at least warms the soul, which they say may not be so harmful. And I think yes, and I’m gradually starting to come to the point of quitting smoking, completely, because I’m starting to realize that very often I smoke there, when, for example, I’m either bored or need to occupy myself with something there, any stressful situations, and so on. And when this is not such a strong need, then it is quite possible that this can be easily abandoned. And therefore, I think soon (.) I will quit. Here. Anything else to say on this topic? Or (.) can we move on?

M. How do you like it, how, I don't know... M. Well, I decided for health reasons, I think it's necessary in principle, all my life I was an athlete, a tennis player, I tried a lot of sports after tennis, then I tried again, football, volleyball. .. M. As far as I remember, swimming, you said? M. Basketball, swimming, water polo, billiards, if you can call it a sport, I tried a lot of things, and when the time came for college, there was much less time for such activities, but I decided that at least just go to the gym there for I need some kind of general health, some kind of physical development, so I went to the gym, probably already about... (..) Well, I've been going for about a year now, (..) every week three times. I also made this decision quite easily, since I, in principle, connected my whole life with sports, some kind of unnecessary activity in my life, on the contrary, it was only a plus for me, my desire for this was there, therefore, too, the choice to go there was rude speaking, go to the gym, don't go, one, probably in terms of difficulty. Or something like that. Well, I probably can't say more about my health (..), well, I think there's nothing to highlight.
As a result, I smoked from the second to the fourth grade, that is, I had been smoking for almost 2 years, being generally a schoolboy who had his head on the table (npt) himself because he was running. But it turned out that I had to quit for two reasons. First, and most importantly, health problems began. I'm sitting there, this is all for me, I've played enough, I won't.
M. But I'll immediately clarify this episode, when you already felt that you, well, smoking began to affect your health, and now your parents burned you, this moment, in general, you wanted your parents to burn you in these activities, or did you not care at all? I. On the contrary, I wanted them not to burn me, (laughter) to receive this strongly, and then for them to know, in principle. Yes, it's just that my father, in my earlier time, probably around the sixth grade, had big problems with alcohol, very big ones. (..) And there it got to the point where I was just sitting, he told me - look, cigarettes, smoke the whole pack there, so that you can get more, so that you feel that this is not the same, and don't smoke there anymore. Well, the like. Well, basically, since I was in school, my father probably doesn't drink anymore. That is, I don't know how many years have passed there, maybe even 7 years for sure. (..) Yes, in general, it's been more than that, probably almost 10 years or something, since my father doesn't even drink anything strong. Well, similar. He didn't even seem to imply that smoking a pack of cigarettes would just be like, well, a pleasure. Here. But no, I didn't want my parents to burn me at all, it happened by accident, a classmate burned me, that is, she complained. Oh, I forgot to specify, so that my parents wouldn't burn me, I sprayed cologne into my mouth. It was an old children's cologne, I don't remember, it was unlikely that it was triple aged, because it was a regular children's cologne. But it was definitely alcohol-based, and I even remember it was "Pirates of the Caribbean", then these colognes were also coming out. And I just poured cologne into my mouth. Here. It was terrible, but what can you do, you had to make some sacrifices.
About health, with health... (..) The story is that, let's say, as soon as I received normal money, I finally went to have my teeth treated. I had a big problem with my teeth and I passed, then I took a genetic test to check for some congenital diseases and discovered several congenital diseases. Here I would not say that I was unlucky, on the contrary, it was good, I saw there and saw some predispositions in terms of hormonal ones too. I went to the endocrinologist and there they also developed, drank some zinc and so on. Here. Well, plus I had a dental operation that didn't go very well, they pulled out a tooth, it turned out to be the sixth, chewable one, and he had a cyst on the root, and the cyst ate a layer into the sinus. And now between, well, where the tooth was, I have a hole in my sinus. That is, such a good message, air can go there, back and forth from the sinus to the mouth, water can go into it, so. And this, in principle, can only be healed with an implant. This, well, with health, it means that it was necessary, well, it was necessary, well, most activities were

prohibited. But now, for example, it's literally been a week here, and after work I'm going to go, well, recover in the gym. Because now I couldn't go to the gym for a while.
M. And now there are those moments on which you consult with your parents? I. (sigh) (h) (...) Well, probably for health reasons, I always consult with my mother, because she is a nurse. Here. That is, if I suddenly start to get sick there somehow, in a way that I have never treated myself before, that is, if I start some kind of ARVI or something there, I can basically cope with it myself. Here. And when it comes to some symptoms that I haven't had before, I call my mother and talk, if they don't help, then I go straight to the doctor. Here. (..)
M. Well, yes, it seems that you hear yourself very well and understand what you need and what your needs are. Tell me, with your activities, what you called a hobby, did you also listen to yourself or perhaps it was imposed by your parents? M. Regarding sports, I probably always liked to do something like that, to feel some kind of power over my body, that is, when you grow, including over yourself. Including remembering, as they say, (laughter) the classics of fight club, development through self-destruction, that's also a wonderful thing. This is also wrong, but I probably came to this too.
M. (laughter) Yes. Here. Therefore, I probably don't particularly value my health, and making decisions here is quite simple for me, I still try to control my health and so on, in the sense that, well, I try to see something in myself if something hurts me and so on, but otherwise, it's as if I've never had such a thing that I have to go straight to the doctor, that is, it seems to me that I'm more than healthy. I recently took tests, and they told me that they could at least launch me into space.
Health - ten, it's very difficult to make a choice, I have some kind of masculine fear, I don't know, or in general it's a universal fear of going somewhere, or arranging an appointment with a doctor, going there, I don't know, to the dentist once every six months. Well, in general, it's just some ten, and I don't know what's stopping me.
A. Oh, you know, this is very interesting, I had no thoughts. Well, that is, if we are talking about some kind of rational reflection, and I mean perspective reflection, that is, when we assess risks, when we, we don't know, analyze a situation that could happen, it in general (xxxx) (laughter) there was none. Well, that is, it's purely based on some kind of emotion, some kind of euphoria, it's probably possible, I didn't use psychoactive substances very much, I smoked marijuana twice, but it seems to me that this is something similar to what people experience under psychoactive substances, such an absolute frenzy and absolute lack of self-control. Here.
Next, health. (..) Yeah, health, health. Here, you know, there is a rather difficult aspect, because for probably several years my health has fluctuated, (laughter) let's say, like the exchange rate of the ruble on the stock exchange. Probably until 9-10 grade, I was quite (.) a chubby boy. And I probably made the choice in favor of a healthy lifestyle on the basis of some, let's say, personal oppression and personal motivations. But for the most part this was due to teenage conflicts. And, accordingly... (..) Any problems. M. Did someone offend you? I didn't quite understand about oppression. A. Let's just say, (..) at some point it began to seem to me that at school, on the part of friends, on the part of teachers, at some sharp moment the attitude towards me changed. That is, they stopped noticing me and (.) paying any attention to me. Although I didn't seem to do anything wrong. Nevertheless, I didn't really understand what happened, but at that moment it was probably quite difficult for me to communicate with people, because I didn't understand what this attitude was connected with. That is, if I had a teacher with whom I was in close contact, and (.) with whom, in principle, I was conducting some kind of project activity, then at that moment it happened, I don't know how some day came and as if at the snap of a finger What happened was that this person's entire attention switched to another audience. And essentially, you know, it's like a child who has been sharply deprived of attention. You want it, but they don't give it to you. And I probably began at that moment to look for disadvantages in myself and in my appearance, in my

health. I began to think that this was probably connected with some characteristics of my body or characteristics of my thoughts. And then I probably started soul-searching. (.) Therefore, the choice in favor of health, it was probably given to me, let's say, through a battle, due to some experiences, through an attempt to prove to myself and many others that everything could be different, that I can do something... something else. Therefore, in terms of severity, probably (..) well, let it be a ten. At work... M. So you connected this deterioration in your relationship with the fact that you somehow gained weight, or somehow didn't look the way it seemed right, right? Because you were talking about the love for sweets, and about being chubby, you said? Or did I misunderstand you? A. Well, for the most part, I've been pretty chubby since childhood, and I probably stayed that way until the 9th grade. Therefore, purely physically, I didn't change much, due to weight gain or loss. There was simply, let's say, one consistency of the body, which was maintained as it grew. Therefore, I don't know, I probably connected this not with an increase or change in weight, but with the fact that at some point some skills and shortcomings that people did not want to take into account simply became more noticeable. M. Did you say something to you, did you somehow become yourself, did you say about choosing a healthy lifestyle? Something like this. A. Yes, I decided that... The Unified State Exam had just ended... the Unified State Exam, in the 9th grade, I thought that since such a situation was happening, I needed to rethink myself, I needed to somehow start taking care of myself, to show myself, well, to change something in myself, not only mentally, but also physically, so I probably made the choice in favor of a healthy lifestyle, in terms of athletics. That is, I got up at about 5 in the morning, slammed the doors, everyone was scared of what was happening so early in the morning, and I just left the house and started running at the stadium, ran to some park and spent my time there.
really don't like this, so I'm trying to gradually return to my usual way of life. And somehow connect sports to your activities. But for the most part, probably due to my (..) fear of people (xxx), some kind of tightness, I don't always succeed. That is, I cannot, relatively speaking, sit in a hostel, do something, get up and start doing push-ups there. Or, I don't know, stand in the plank. Because, (.) you know, this is an internal experience when you look stupid in someone's eyes...
M. Well, yes, I understand. A. Yes, for the most part that's why I got up early in the morning and ran away earlier, to get away from prying eyes. M. Well, yes, very clear. A. Now, well, basically, what you can do is go out late in the evening, we have a small park, a student park, and do a few laps of running there if you have free time. If not, then I try to diversify my diet and make it, let's say, less high in calories.
And so, if your hobby is sports, crossfit, cycling, snowboarding and generally active recreation.
T. And I remember myself probably from the age of one... Well, probably from the age of six, from five, somewhere like that, directly consciously, so to speak. Well, as a child, I initially wanted to become... (..) To become a policeman, that is, at that time, a policeman, and, in principle, my parents did the same, if we talk about independence, that is, they always signed me up somewhere, to some clubs, some sections, that is, since childhood I was kind of fond of sports, that is, I was such an activist, that is, both at school and in some extracurricular activities, social events, that is, always for the most part, they attracted me, too, and (..) it seems so, and some leadership qualities were probably emerging, so this is where the roots probably go, maybe some of my independence in making some decisions. That is, as I have already said, the sphere, so to speak, of my activity is quite wide, that is, starting from the public... That is, this has been going on since childhood, that is, now I, let's say at work, am the chairman of the council of young scientists specialists, these leadership qualities that have been going on since childhood also manifested themselves. And I finished school, that is, until the eleventh grade, passed the exam, and entered the university. At the university, of course, it's also a little less, since after school, for some reason, I began to devote

more... More time to studying, not in the periphery, but specifically to studying, and therefore, a little less, I had classes that were, so to speak, excellent from studies. But still, no matter how I gave it up, I also took part in all sporting events and social events. So I studied and studied, and naturally, after that, after that, I went to work. And at work, from the first, as they say, days, that is, he showed himself as a fairly active person, went out with his colleagues somewhere, that is, he was always, so to speak, easy-going. And, that is, after that the management noticed and, as they say, I am still, so to speak, in plain sight. That is, just like the leadership, as it was before, like at the university, like at school, and now, let's say, the strategy can be action, that is, just independence, leadership qualities, and so on, then are present at the moment. Here. In principle, I think, more or less answered. (laughter)
T. Well, I have this character trait: sometimes I follow the majority. That is, in order, as they say, to go against the flow, namely, my closest circle. That is, my environment was quite like this, just sports, that is, we were engaged in dancing, and naturally this was vocals. I was, I looked at other people, I looked at how they were, whether they were achieving success in this regard, and then I tried it on myself, whether I was even interested in the same activities that were popular at that time, and I already made a decision. And if, so to speak, in percentage terms, they probably have their own opinion and people's opinion, well, in general, the opinion of the current, the opinion of society, so to speak, is probably about 50/50. Well, maybe 60/40, in my favor. In this plan.
M. And if we talk about health, then when you make decisions in this area, what helps you do this, for example, a consultation with a doctor or how do you even choose a dentist? T. Well, it turns out that in this regard I'm probably suspicious about my health, that is, if some kind of sore pops up, I start there, well, not exactly panicking, but it's faster, faster to take some action to remove this pain. Just with teeth, and everyone knows that this is a rather expensive procedure, namely dental treatment. And therefore, finances did not quite allow, or rather allowed, but there were other, well, one might say, demanding, that is, aspects that required an urgent investment of money. And that's why I put off just about the teeth. Here. And therefore, in principle, since I have already said that I am suspicious, I quickly, quickly tried to fix all this as quickly as possible, so as not to start all this. Then don't pour in even more finances, already eliminating the consequences of all this. M. But I'm even asking more about how you understand who is a good dentist there and who is bad, about this. So, where do you get this? T. Well, here again, already through acquaintances, that is, I found out from people who treated there, and about prices, and so on. There was no such thing here that, yeah, I typed dentists there into Google, and went to the first link that came up, and immediately went there. That is, naturally, at first there was some (..) research, so to speak, on this matter, that is, I found out from friends there who was doing good work and, accordingly, I went there.
T. Well, in principle, all my successful decisions, which were made at the junctions of the most important stages in my life, were at school, that is, the choice, I don't know, not just there, I don't know, walking around the entrances, smoking and so on, have a drink there, namely, go in for sports, that is, some kind of social, some kind of personal development. At the university, that is, to enter exactly the university that you are now, well, in which you naturally studied, (..) it is precisely the choice of the field that is most promising in our country. And at work. That is, I work in a fairly large company and this, in principle, makes me happy. I don't think that somewhere in these probably most important stages for myself, in my life, I made some wrong decisions.
I. So health... What elections, elections, elections, elections, elections, well, it seems to me that with my health pah-pah-pah, everything is fine, I try not to get sick, (laughter) here. It seems to me, well, if we take health in general, well, my mother just had a stroke there. I had to make certain choices there, I don't know, about apartments, about inheritance, and so on and so forth. This all also falls on me, in any case, so for me, well, it was hard for me, it's still hard for me to resolve all these issues, because in many areas, with many of these things, I'm meeting for the first time, there

the insurance company, the hospital, I don't know, they have to process certain documents, it's incredibly difficult for me. As a child, my health was bad. As a child, after childhood bronchitis, I had very severe bronchial asthma, I was sick for a very long time. I rarely, I didn't go to kindergarten at all, and I went to school, but I was sick very often, of course. (..) It was very difficult for me as a child, so it seems to me that I would rate my health as exactly this, I would rate it as an eight
M1. Oh, I'll start now from the earliest, it's just that my mother has such a perception of life that she was against kindergarten, due to negative experiences in childhood, that she remembers by her example that teachers are very like that, not so much, and may not monitor the health and so on of the child. And she always sent me to some private development centers, and if you take me from a very early age, there are speech therapists with me, reading, something else, that is, in addition to the fact that my mother worked with me, that she is like me in fact, she taught me everything, before that at school, I went to such educational programs, and when I became more conscious, as an adult, probably at 4-5 years old or something, well, at 4-5 probably, yes, at 5 somewhere, I went to the children's center, combined with a fitness center for adults, and there were all sorts of sections like judo, fencing, and all sorts of other things, that is, I kind of went, it just sounds cool, interesting, some kind of dancing, gymnastics, acrobatics, absolutely went different. Accordingly, later, when I became even older, my range of interests was outlined, these are creativity and sports. Here. As a sport I went to judo most of my life, then 2 years to boxing, 2 years to karate. And I probably practiced judo for 10 years, or 12, I don't remember. I graduated from music school with honors, so in terms of interests, you can say, well, little by little, let's say take a swimming pool, creativity is a guitar, some kind of drawing, singing in different sections, that is, I actually did drawing in many different sections. I won't say that it was some kind of cool level, but just purely for myself, to communicate, to spend time, it was. M. And at school, it turns out that you also managed to study very well, in addition to these sections? M1. Yes, I can say that I, this is not my assessment, this is the assessment of others, that I am smart, and I had an interesting situation, that I understood that I could easily be an excellent student, I am not a fool, I can prepare, I can do everything, but I was just kind of lazy to spend so much time, and this is probably not very good, but I never had problems at school, any difficult subjects, the most difficult subject for me is chemistry. Somehow, out of interest, I just took it and decided, I'd better learn the topic, and then I didn't study it anymore, this topic, because it wasn't interesting to me, that is, I understood that I was doing things that were more useful for myself . Accordingly, I had no problems at school; if you look at the boys, I was among the smartest, that is, in terms of grades, and everything was fine. And this despite the fact that my gymnasium is one of the best schools in my city, where studying is quite difficult.
That is, my schedule is like this: at school I communicate with them, after school I go to the music room, there I communicate with these people, and from the music room, let's say I go to sports, there I communicate with these people. And this is how it went cyclically for me. That is, I communicated with many, but I won't say that all of them were my very good friends, they just communicated well, well, in some respects they were friends, naturally, in each section I had one or two people with whom I was most communicated, I think these are adequate things. Well, something like this.
M. But about the karate trainer, tell us how he helped you develop independence, since you speak about him so warmly and in detail. M1. Yes, I speak very warmly about him, about the coach, because, well, this overlaps with the fact that I spent a lot of time in training, a lot of time, from the tenth grade I did karate, it got so bad that I had, let's say Monday, Wednesday, Friday, two workouts a day, 2 hours each, and the remaining time, let's say one. Each training session lasts

somewhere for 2-3 hours and I was with him all the time, like listening to how much he loved to share some stories, how he not only enlightened us, but simply talked about all sorts of life topics, I heard him talk about it, I drew some conclusions for myself, what I can do, how I can do, similar things. Here. In general, just in my opinion, even in terms of education with a child, the main thing is to talk to him stupidly. Just talk. Tell some moments, life situations, how he got out of these situations, so that the child has some kind of template example that he could use in the event of some new situation. And then impose your own, having some kind of behavior model. That is, it seems to me that it is from conversations that a certain model of behavior is formed so that the child can use it and accept it as a given, in order to develop it later. He can completely redo this pattern of behavior, in case of some extreme situation he won't have to think, but what should I do, he will remember - yeah, my dad did this, or my mom did this, you can do it like this. He will do this, and then he will think, what is best for me? He will do what is best for him. That is, just in terms of conversations, all this is being formed. That is, the coach said that in reality martial arts are very connected with personal perception, and you cannot teach martial arts if you just come, roughly speaking, once a week to training and just somehow teach the technique. He said that all the masters, they lived with the teacher and seemed to watch him, how he behaved in the natural environment. They seemed to adopt the image, likeness, and mind of the teacher, how he behaves in a normal environment, outside of training, outside of sports. And this is the difference between a coach and a teacher. I can call my coach a teacher. For example, I can't call a judo coach a teacher, because his task was to make athletes out of us, well, he made athletes out of us, he is a professional in this matter, but there were no such life conversations. Because that was not his task. My karate coach had a different task. He talked to us a lot. Accordingly, well, somehow he is still just a teacher by training, I'll tell you what, at the university, so he knows what to talk about and how to teach. So it was very interesting and educational. And it seems to me that it was karate that revealed a certain personality in me, in terms of absence, removed some edges, that I can live calmly, socially, not be afraid to meet someone, do something else, because I don't know, how it happened, but I'm sure it was after karate, because martial arts associated with a certain kind of combat, they greatly change the worldview, perception, and seem to remove some kind of framework, boundaries. You become more confident. And quite independent. You could say. Because you are not afraid to defend your point of view in some dispute, knowing, like, that if someone comes at you, you will be able to give him an answer and give him a very good answer. M1. About the karate teacher and dash teacher, I was wondering if he has an education, you said that he teaches at a university, but what does he teach? M1. He is the dean of the physical education department of our Ryazan State University. M. How did you even get into his section? M1. This is also a very interesting story, I... (..) I just somehow saw how training was going on, in karate, in a completely different place, in a completely separate place, in the Krasnodar region, when I was with my grandparents in away, I saw how the training was going on. I was so impressed by this, because at that moment I was doing boxing, and I lacked some kind of morality, some kind of cultural core, when before training everyone bowed, sat down, closed their eyes, well, not just prayed, but they just sat down as if getting ready for training. After training, a bow, ritual phrases, when there is movement and intelligence, not just a straight punch, a side kick, but some beautiful Japanese names, Japanese language, culture, kimono, that's it, equipment, I really liked it, it went in, and I I started looking for this section in my city, I found a section in my city. It was another coach there, who reported, in fact, to the head coach, whom I spoke of as a teacher, I went to him first, but then this coach said, that there was an opportunity to go to some training camp, go to another building, with this head coach, so I went and somehow it happened that the coach liked me and he decided that I would work with him in the training camp team, national team . And I began to study with him. And I spent, in about six months, actually, I actually studied

for six months, I went along the path to a black belt, in fact, because I worked a lot, plowed, studied, learned new things. This is my story of meeting this karate trainer. Now we don't communicate, but I remember him very often, that is, in some conversations, and I am very glad that such a person appeared on my life's path.
M. Listen, but about dependence on someone, you say that you don't like to be dependent on someone, but why, that is, what do you put into this dependence? M1. Well, I had an offer from my coach to become a personal student, in Japanese traditions, as it were, that is, roughly speaking, to live with him, that is, to do what he does... As if he essentially becomes a certain , not exactly a master, but kind of dictates to you what you need to do in order to develop. Yes, I would become an athlete, but again I understand that I might not become if I had some kind of injury. That is, I did not want to put everything on this card. I didn't like this concept, that you are completely dependent on a person, that... For some, this is good, you don't have to think about what to do tomorrow, but for me it's not good because I kind of control myself and do what what will I need. That is, as much as I don't like being limited by someone. Any limitation here is not very good. That is, there you go. I like the Higher School of Economics because, by the way, there are no restrictions on studying, that is, you don't even have to attend lectures, that is, you are, as it were, responsible for who you will be. That is, there is no such strict control, so what - oh, you need to learn. If you don't study, you'll fly out. (laughter) As if only this fact stops you and that's all. There are no mentors there to control them, no micro-management...
M. What brings such warmth to memories of school? In primary school? M. There was somehow more friendly communication there, with the team, but the only thing was that I was very far behind in sports. When they played football, volleyball and so on, I usually sat on the bench. M. Did you want to continue, did you want to play together? Or were there some reasons why you didn't play? M. To be honest, it's difficult to answer, because on the one hand, it seems like you want it, but you don't seem to want it, this. M. Was it your choice to sit and not play? M. (..) No, I think my lack of skills played (..) in this.
M. Yes, then you can move on to the next point. M. Choice of lifestyle and leisure. Well, this is where it gets more interesting, by the way, because I decided to change my lifestyle. He was like this, he is like this now, more sedentary, not very active, I found myself in such a rather interesting situation that after my previous job and previous company, all my interests simply died, because all my time was taken up by this sweatshop work and gatherings there with drinking there and so on. The rest just somehow fell away. And now I think what is interesting to me, what is not interesting to me, I restored my physical activity to a minimum there, in the future I will... In the future I will return to sports. I've even already decided what exactly it will be, what it will be.
M. And what will it be? M. I want to go to boxing. It's very necessary, in fact. M. Why boxing? M. Well, because it is he who has a great psychological influence on people. Well, according to my observations. This is both confidence and the ability to stand up for yourself, which is very important to me.
V. No, there was no such question, but my mother tried to compensate for this so that I would somehow interact with society, so I went to an English language club, to drawing, in general, they took me to all sorts of different events where there were other children, Mom often went out with her friends who had children, so it cannot be said that I was deprived of communication with children, just food, catering, my mother did all this at home, that's it.
V. Yes. Yes, I wouldn't even call it stress. I kind of liked this state of competition again, another one, here. Well, so did the entrance exams, and I was like, oh, I entered Moscow State University without a basic philosophical education, that's fine, now I'm also a translator. In general, this energizes me. So, in short, I always loved this state of competition, and as a child, when you are

not competing in the professional market, this is probably one of the only niches where you can compete, these are games, well, such games, board, computer, or sports games. Here, by the way, I would actually dwell here in a little more detail, because I love, since I love this whole business, I did both, and the third, that is, I always played board games with pleasure, a la cards and sports games. I say, I played chess as a child and table tennis. Here. But... But just at the age of about 12 years old, that is, at 11, I started playing computer games, and at the age of 12, I left sports and stayed only in computer games. Here. I wonder why this happened, well, I think I don't know. (laughter)
V. Well, yes, with health, well, I'm generally not the healthiest right now, I have allergies, and a bunch of other diseases, but our medical system, it's usually in no hurry to help, and usually, until you get completely ill , somehow they're just trying to tell you, like, go drink oak bark there and everything will be fine with you. In general, my health taught me that no one except me will worry about my health. Therefore, you need to show maximum will to get a referral, to get the tests that are needed to get a referral to a research institute, and so on. And there are elections, that is, here, too, it's probably difficult to implement, that is, to withstand the doctor's pressure, because the doctor has authority, that is, like, are you going to teach me, puppy? You (npt) like me, did not undergo residency training. But, you need to show pressure, I then wrote the first complaint in my life to healthcare, to the Ministry of Health, here. This is just such a space of struggle for me, health, so I can say that this is... M
A. This means that what happened, well, I had certain health problems, (..) they are related to reproductive function. She was questionable to me. Accordingly, I needed to get a referral for surgery, this is why the health sector is such an important thing. So, I needed to get a referral for surgery and it's very difficult to do, it was, well, this whole story began in 2019, that is, the diagnosis came to light. So, I tried for 3 years to get a referral, and at one time I received it, but then I got sick, it expired, and they didn't want to give me a new one, so. Who were the participants in this situation? Well, the main participant in this situation was me, naturally, and doctors were involved in this situation, but they were more like passive observers than participants. I would say my mother participated more, because she asked some doctors she knew there and so on. My girl too. Where did this happen? So, okay, let me tell you a little further, in general, I tried to get a referral, I didn't succeed, then, in one private clinic... Well, in general it turns out that this operation is very rarely done without any additional shady payments, that's it. And I, I have a principled position that I don't give bribes for anything, well, in general, I don't want to violate this life principle. Here. Well, in the end I decided to do this operation for a fee and it was unsuccessful. And if... Which I perceive, in fact, as a wrong choice, that I probably should have taken a more careful approach to choosing a doctor.
Q. A year ago. A year ago I had this operation, here. Then I felt pretty bad for six months, well, it was very bad for probably three months, so then I made a decision for myself that, well, sort of... Well, first of all, I don't blame myself for this, for the fact that I there may not be enough... First of all, there is no guarantee that if I tried another doctor it would be better. M. Well, yes, there's no way to check it. V. Yes, there is no way to check. Here. And I was guided by the recommendation that was given to me, that is, in principle, I'm not just like - oops, I'll go to any doctor. I've really come a long way, but the fact that it ended in failure does not mean that the path was wrong. M. Well, yes, it's not your responsibility. You did, you approached this situation responsibly, as far as I hear. That is, they did everything. V. Well, yes. So, in this regard, I calmed down. At first I kind of thought, damn, maybe it could have been better, then I thought - no, better, well, much better, as if I did everything I could, for my part. This is the first moment. And then, then I decided that I needed to get a good examination now, that I could... And then I kind of asked myself - what can I do now, that's what I need to get examined again now, I started knocking out all sorts of directions,

there same level of hormones, what can be done about it? Here. And I began to move in this direction, received a referral to doctors, so. And then I started dating the girl I'm dating now. When I was just going through the referrals, and before I started dating her, that is, well, it was as if we had already started to have some kind of romantic relationship, so. And I tell her that well, right now I'm not at all sure about any sexual things that I can give you, so I say that let me first go to the doctors, they will prescribe me some kind of adequate treatment, and.. It's just that if I'm in a relationship, then I will feel uncomfortable, that maybe I'm not giving something that I should give in terms of my ideas about relationships. Here. And in general, somehow in the end I... By the way, this motivated me to speed up in that direction, to move, specifically to go to doctors faster, and to get directions with greater intensity, that's it. Well, somehow we continued to communicate with her, with this girl, and in the end all my functions returned to normal on their own. Here. Yes, but this happens, there are paired organs, the functions of one are taken over by the other. Here.
M. So the uncertainty was scary? V. Yes, I didn't understand, that is, which doctors to contact, where to go, that's it. Both then and there there was still such a problem that everything is very slow in our healthcare system, that is, there, for example, they give a referral, but the appointment is only a month and a half later. I'm like damn, what am I supposed to do for a month and a half? I, I'm very worried about this, but I can't do anything other than this recording. M. Yes, it's terrible. V. Well, that's when I started writing complaints, this greatly speeds up our healthcare system, by the way (laughter). Here. Moreover, even those to whom I did not write, you just tell them that you have already written one complaint, immediately become more and more talkative. Here.
Well, next is probably health. A year ago, well, a little over a year ago, (..) I had an operation there on my leg, to straighten my toes, that too, it was a necessary operation, and after which there was six months of recovery. Here. And... M. Necessary, that is, it really interfered with your life? Has this problem been around for a long time, or has something happened? K. Well, about two years ago, even 3 years ago already, it appeared, at first I didn't do anything, it went away after some time, there after ointment or some other such exercises, but after that the condition began to worsen, and It got to the point where it was painful to walk. Here. And so a decision was made there, here. And this summer I also thought about having the same operation on the other leg, but now all this is being postponed for me. Perhaps next year, perhaps even the next year. Because there are (..) various issues, including financial, time, because you definitely need to spend a week there under observation in the hospital, and after that you need to wear special shoes, and now it will soon be quite cold. It will be uncomfortable to walk in such shoes, so I am putting off solving this problem.
Here. Finance. Well, here the choice is probably only within the framework of finances, it's about health and about resolving some issues in this regard. M. Is this about leg surgery? K. Yes, including, plus other various problems, there in the summer I decided to do dental treatment, so. Also when it started to become quite expensive, and then other ailments appeared that needed to be urgently addressed medically, so. Therefore, dental treatment there, too, has been postponed and is being postponed there indefinitely.
About training, professional development - I decided to engage in self-development in the field of programming, I selected courses for myself that, in an amicable way, I would need to take. Because, in fact, I personally plan to work in the field of (NPT). Well, that's probably all. Health, I'm in... Six months ago, when my dark streak of procrastination ended, I started playing sports again, going to the gym. And during that period, I tried to arrange healthy meals for myself in the hostel. I stopped subsisting on some fast food and other semi-finished products and started cooking for myself. choose a diet.

Okay, I'll try now. Training, professional development, probably six, making a choice may not be that hard, easy to say (npt) here to understand. But it is difficult to implement this choice. Health, probably three, but in fact, the gym and healthy eating take away almost everything from me...
K. (.) Yes, these are some restrictions on the part of the parents, because of vision, for example, you couldn’t play on the computer for more than one hour a day, there are some restrictions on the time spent on the street, that is, when to take a walk there , restrictions...
M. I understand. And here’s another thing about your vision, has it changed somehow now, or what’s wrong with it at the lyceum? K. Well, in high school it got worse, but there it’s also hereditary, and because I read a lot (.) in the wrong position, it got worse, and it’s still bad now. (.) For now.
M. Did you have any prohibitions in childhood that you definitely shouldn’t, both in childhood and in adolescence? S. Only if you consider it an allergy, that’s all. M. That means taking care of your health, right? S. Yes. M. Was this somehow instilled in you by your parents and did you want to break it, or how did it happen? S. No, it’s just sometimes... Well, it’s like I’m allergic to nuts, but I really like nuts. But I understand that... What will be the consequences. And, well, like, as a child, I somehow didn’t attach much importance to this, but I go to camps, let’s say some sanatoriums, my mother always tells me - do you remember that you can’t have nuts? Do you remember what will happen? And then I, here. And in terms of alcohol and smoking, I was somehow not prohibited at all, but I also had no desire. Here. And my parents forbade many of my friends and now they are just having a nightmare there. M. That is, on the contrary, the ban gave rise to... S. Yes. Because with this ban, they seemed to have more and more desire to try. They didn’t forbid me, my mother always told me that you can try, but it’s not a fact that you will like it. Here. I tried alcohol for the first time at the age of 18, at graduation. M. That is, quite recently, right? S. Yes. And somehow, I don’t know, now I drink a maximum of champagne, wine, and on holidays. All. Smoking, I have it in general, I don’t even have the desire, I’ve been offered to try it so many times, I say, I don’t know, I don’t want to. There is no desire at all.
decided to carry it myself. (..) Well, I went up to the guys, near the tents, to the boys, I said - boys, can you please help me carry the suitcase, I can’t do it myself. They told me - no, we can't do it now. I say, okay, okay. I'll carry it myself. Here. And somewhere in the middle of the journey, I already realized that I simply couldn’t cope, that this rib was starting to ache a lot, there was pain. And I wrote to the chat with the guys, I said - guys, the boys really need you now, please help. Here. And no one reacted at all. Here. And I just walked, roared, carried this suitcase, so I carried it. But most of all, I don’t know, what made me laugh was that I was walking, and so I met many young people, they were just walking like - damn, she’s so cool, really, strong, independent, carrying a suitcase herself, in general. I walk and think... M. And it was very painful for you. S. And I walk, roar and think - you didn’t think of offering help, somehow, I don’t know. I even, I don’t know, I always somehow offer help... (..) I, I don’t know, I’m used to doing this, so. Then I just went to the doctor, they told me a lot of things about how I shouldn’t carry heavy things, but I went and did all this, that’s it. So. Do you often think about it, about this situation? (.) Well, recently, often, because it happened recently. (laughter) Here. I told my parents, my mother said that everything can be expected from me, and what emotions arise? The emotions are so mixed. M. Can you explain? S. Well, in the sense that yes, I myself was able to convey all this, but it was to my own detriment, but also that, as it were, I don’t know, we were always told from childhood that boys should help girls and all that. And then they just go like this...
M. That is, it’s like the ability to solve your problems, the ability to choose how you want to spend your leisure time. Tell me, in setting your goals, do you show independence, focus primarily on yourself, or does someone influence you? M1. Yes, of course, I primarily focus on myself, that is, for example, with the same boxing, as it were... (..) I really, like, all the girls are like that - boxing,

why, why do you need boxing? I'm like this - I want to go boxing, that's it. (laughter) I went, found it, went, liked it, and now I go, and that's it, everything suits me. If it concerns some goals of others, then yes, I can set goals for myself, I can fulfill them on my own, but here it all comes down to financial dependence, sometimes I cannot achieve a goal due to the fact that I do not have the funds for it implementation.
Well, perhaps, when my neighbor became ill, she began to choke, and... That is, a person is suffocating, he doesn't know what to do, this is his first time, and it's like... That is, I called an ambulance, there I woke up everyone , she said that yes, we started googling something together, what to do and so on, that is, as if she had something like this, nervous, panicky, that's it. But then she went to the doctor, well, it was like, you know, an independent decision that at the moment I should help the person. Because she can hardly help herself. M. This was probably a very stressful event and somehow you reflected on the moment when - now I'm doing it, or did you just start doing it yourself because you realized that the responsibility was only on you at this moment? M1. I guess I just started doing it on my own. M. Do you think this situation played any role for your independence, or did you simply show what you already had? M1. I think I just manifested what I already had, because my sister is often sick, (laughter) more precisely constantly, so I kind of got used to packing things in bags when I have to go to the hospital, that is, for me it's a familiar situation, so it was sudden in terms of its appearance, but in terms of (..) how should I say it, in terms of factors, it was similar to many situations in life.
Regarding health, there are probably no solutions. At least nothing comes to mind. There, every year you just go to the doctor, and he tells you what to do and that's it. (laughter) M. So you are generally calm about your health and therefore somehow don't even remember? I. Well, I probably haven't had anything like serious health problems lately, if something happens to me, I go to the doctor, but I don't know there, I recently had poisoning, I go to the doctor, he me right away (npt) (laughter) but there were no such, well, particularly important ones, but probably if I had had any health problems lately, I would have remembered it better, but so, (laughter) nothing there was no specific one.
As for health, well, as such, well, firstly, I chose to do a health check-up in order to identify some... Some areas in which there are some problems, this was already a big step, because I couldn't get to a regular doctor. And so I chose not to put off some moments. Of course, now, with a new workload, it's been postponed for some time, but I think I'll get back to it soon. (..) And I will start again the procedure that I was doing. M. And what about regular health checks? How regularly will this happen? A. Regularly - this is six months. (..) And about work, work... M. Sorry, please, can I ask you more, about your decision about regular health checks? A. Yes, of course. M. How did you decide to do this? How did you make this decision? A. (..) Well, since we now had Covid, I... And I never had confirmed Covid, I decided to go and find out if everything was fine with my lungs and everything else, and since now there are pavilions in Moscow, health pavilions, and there is one of them literally next to my house, I thought that it would take an hour, an hour at most, and I have an hour of free time, I can go there and do it. And also, well, when... (..) When you underwent medical examination at work, mandatory, it's also like a choice that we are not given, (..) what you are obliged to do, and also this, well, some your own feelings that something is wrong and you should go to the doctor, and he will refer you where you need to, according to your needs.
And probably, I also really wanted to do rhythmic gymnastics, I watched a lot of all sorts of videos, there were performances at the Olympics, at some sports competitions. And I kept asking, asking my parents to bring me to the gymnastics group, and then I still don't know, maybe my persuasion somehow influenced my family, maybe something else, that's it, but I still They brought me in and I started playing sports. And I understand that it's probably sport, it has played a

lot in my life and in my character, too, a lot from sport. I have there, regarding the achievement of any goals that I set for myself, that I can gather there, even if I understand that it's hard for me, bad, or something else, I understand what I have after all, there's this kind of core that probably doesn't allow you to give up when you just need to pull yourself together and do something, come up with something, that's it. Or somehow cope with the situation that exists. Here. And probably the fact that in my family, in general, (..) there is such a moment of complete freedom. That is, I chose, after school I entered a technical school, because I did not enter the university, and my parents said that you choose a profession and direction yourself, because we want you to be a good person. And of course, perhaps, I think that this was bad advice, because when you are 16 years old, and you want to get specific, well, some steps, how to choose, what to do, maybe how to look at the test results, according to career guidance, which shows you 16 areas of activity, and you don't understand what to choose. Here. But it seems to me that this is my path in education, it seemed to be a little in different directions, but it seemed like it was almost cultural, almost literary, probably. Therefore, somehow everything comes together, as if like a puzzle, here you are, seemingly from different places, but you find puzzles that your picture is missing. It's probably something like this.
stopped playing sports at the age of 16, and probably now, after a certain number of years, I would tell myself that I still need to continue to exercise, even a little, even a little bit at a time, maybe a couple of times a week , but still do it. Because at that moment I had completely finished studying, that is, I had some activities, not much at all, but still, here you go. But I wouldn't want to quit. This is probably the choice I regret. M. But if you go deeper, when exactly, how old were you when this happened? P. I was 16 years old, it turns out that I graduated from school, began studying at a technical school, and I simply did not study in the area in which I live, and not in the area, and not even close to the area in which I played sports. And plus it so happened that at that time the group in which I was studying was disbanded, and then I thought that, well, the coach and I also discussed that it would probably be better for me to look at some university or technical school somewhere. activity, because I trained with girls who are younger than me and from a sports point of view, this is kind of a step back. And significant, because if you train with those who are on the same level as you, or a level or two higher, that's good. And when you train with those who are kind of far behind you, it's not good. Because you, too, will roll back to approximately their level. Here. And then, yes, at that moment I thought that yes, it would probably be difficult to travel, because school would end anyway, it would be very late and there was still a road to class, and classes too, and it would be difficult. Here. Well, then I bought a membership, a membership to the fitness room, that's it. But it was also difficult with him, because he was also not so close, but still. Here. And I went and worked out, but I understood that this was not enough for me, and I just wanted gymnastic activity at that moment. M. But you said that at that moment, well, as an adviser, well, the coach was present in this matter, and who else? Who else was involved in this situation? P. No, just me, me and the coach. So, it turns out, I told her (..) my situation, and so, well, she suggested this option. Of course, she wouldn't have refused me if I had said that I would like to continue, so. But she outlined the situation to me, as she sees it, as a professional, as a coach. M. But if, in contrast to unsuccessful ones, we talk about successful elections, what elections do you think were successful in your life? P. I would probably highlight the choice of sports, gymnastics, as especially successful. I understand that it's mine, and I understand that it was very important and great that I liked it. And I'm probably lucky in that this sport is interesting not only to me, but also to my family, and my family really supported me in this, then the choice... (..)
M. Where do you work? M1. I work in a private children's correctional center as a speech therapist.

Well, that is, yes, you will go to medical school, Yes, doctors, doctors, this is always needed, well, they are like that, one of the pillars of society that will always come in handy, you will always need to treat people. Here. But it is very difficult to find, unlearn, firstly, spend a lot. And then, I couldn't answer why I needed this, so somehow, not finding answers, I slowly walked away from it. Well, it's a plus due to the fact that then my mother, grandmother is a pharmacist, my mother is there in the field of obstetrics, and somehow, knowing this whole kitchen, I didn't go there, so this is also an important, it seems to me, such a stage, when I reoriented myself, but it was as if it was unclear where. Further, my interests developed - social studies, history, well, closer and closer and closer I seemed to come to education and the humanities, to the humanitarian sphere.
Another important choice, which in general, if in the future 10 years, then (..) it's probably closer to the family. Well, that is, as it were, a choice in favor of (..) building long-term relationships, and another related choice - health. Well, that is, as if I don't want to waste what I already have, because our body is only depleted, but I want to somehow fill it up, maintain balance, different areas of life that affect health, ultimately well-being, and the feeling of happiness when you feel good. This is a choice in favor of, well, taking care of yourself in different areas. Well, that is, starting there, I don't know, with some kind of tests, check-ups, regular support for physical fitness, psychological health, such a minimum of some kind of psychological hygiene, then yes, this is directly connected for me with family relationships, because in Ultimately, if you make a choice, then for now I have the feeling that I am not childfree, but I still want some kind of (.) continuation of the family and continuation of the clan, if you can call it that. Again, I want healthy children, a healthy child, and this means that I must be healthy. And the other person must be healthy. Therefore, this is a long-term construction, with certain resulting requirements for a person. (..) And probably not by requirements at all, but by similarity of beliefs. When they look in one direction. Well, probably for 10 years, well, another important one, this is first, in the short term, somewhere up to 5 years, a period of time to take, then this is a set of expertise and experience, packaging it and going out not to work for someone, but for myself.
M. You can go straight through the list, you don't have to arrange them. K. Training and professional development is... Probably there will be seven, because (..) you always have to learn everything, and sometimes it's very difficult to allocate your resources and brains, (laughter) let's put it this way. And do one thing. Health is five, (laughter) it's either there or it's not, work is also six or seven, because... M. Wait, let's go back to health, making specific decisions. In terms of health. K. Regarding my health, everything seems to be fine, I feel good, but sometimes there are times when I get sick, (laughter) Covid and so on. And I try to recover as much as possible. But if I get sick, then I don't do anything, it's immediately minus work, (laughter) minus friendships, only the place of residence will be there then. (laughter) At maximum. M. What about choices, such as choosing which doctor you need to go to, or even understanding whether you need to seek help or not? K. Choosing a doctor, I don't know. I somehow... M. How difficult or easy is it for you? K. No, it's easy for me to choose a doctor. I just see, either from reviews, or using word of mouth, that this doctor, we went to see him, he's good. I'm so good, I'll go to him too and everything will be fine. In principle, this is how it turns out, so I don't have such a difficult choice here to choose a specific doctor. Well, usually, I just probably didn't get sick with anything serious, no matter where it depended on me... The doctor's choice is between life and death. Therefore... M. In general, you said that you rate your health as a five, then what is the difficulty in making decisions regarding health? K. Health, difficulty making decisions, (laughter) giving injections. This is where it's difficult. (laughter) Or force yourself to go... It's just that when you're sick, you don't really want to go out. M. So it's difficult for you to understand whether you need help or not? K. Yes, I can't, it's sometimes difficult to assess the extent of my illness, it just seems to me that even with a

temperature of 38 you can still do something there and at work, everything is a doctor (NPT) in principle (NPT) and you can live.
D. It turns out that at the age of 11 I was told that I had the fourth degree of scoliosis, idiopathic, and that this could no longer be treated, perhaps I should have started going to some massages there a couple of years ago, doing something, but I had already encountered with a severe form of this scoliosis, and they told me that... Well, I was again given a choice, I remember how my mother seriously decided to talk to me, at the age of 11, that Dasha, you have such a situation, you can (..) live with this scoliosis, that is, it's like, well, I would have developed a hump, very strongly, it had already begun to appear, or I would have to undergo surgery, and they would put me in a metal structure for life. Into the spine. And when you're 11, you don't really understand that they're going to do something to you now, and you'll have to live with it, well, for the rest of your life, and I made up my mind, and basically, my mother told me that I walked this path very steadfastly , and lay in the ward after the operation. The girls who were 10 years older than me were there, they cried, it was hard for them, and my mother said that I went through it all with courage (laughter). Here. This is probably also an important path. M. Yes, I really sympathize, well, it's really a choice, and it's such a significant one, and I think that you are probably faced with some of its consequences. Does this have anything to do with tennis or not? It seems like... D. My mother thinks yes, that it's because of tennis, but I think that... (..) Perhaps, but there is a reason, there is a reason. Perhaps this somehow prompted, well, aggravated the situation, but obviously it was not the reason. Most likely it's something genetic, perhaps I had crooked feet from birth, and they tried to correct them for me somehow, here. Did not work out. And you understand, yes, that when the weight is unevenly distributed, then all sorts of scoliosis results. That's probably because of this.
Then health. Well, from the last one, probably... But this is probably some kind of funny situation, there's not much choice here, I, I felt bad, not so long ago, a month ago, and I called an ambulance, I didn't want to call it for a very long time, because that it always seems to me that my situation is not serious enough to call an ambulance, for someone it is now perhaps more important, more necessary, so. And when we arrived, it turned out that the situation was very serious, I had some kind of intestinal inflammation. And they just gave me a choice - to be hospitalized or not. (laughter) And I, too, somehow without really thinking, decided that it was necessary, let them check me and all that. And it was also an unpleasant situation that I was out of work there for a week, and I was ashamed, but I thought that if this, (..) if I don't get cured now and then it all somehow piles up, and it will be like like some kind of lump of snow, just like that. Well, in general, some more pain will be added, and then the consequences will be more difficult to deal with than now. I hope I made myself clear. (laughter)
How could it be, what else? (laughter) (..) I think, I think. (laughter) I still have something from childhood, like, let's say, principle, choice, I never drank alcohol, well, cigarettes, that's understandable. And even when I... Well, that's how it is in Russia, they are always trying to offer something to you, and as if in theory, the majority drinks and considers it normal. (laughter) Yes, and when you don't drink, in general, they are all so surprised, well, come on, well, try it, (laughter) but I always had a clear motive, this has been since childhood, that I am not going to drink and I will never. Well, as an example. Well, even when, relatively speaking, I was moving into a hostel, the neighbors somehow, well, there are three of us, and they suggested how to celebrate a housewarming, they asked me - will you? I'm like, no, I won't. (laughter) And they were like - well, okay. But I always hear this phrase - you will start someday anyway. That is, I don't drink at all. Not for the holidays, well, it doesn't matter. Just never and not at all. Here. This, so to speak, (sigh) is my principle and choice. (laughter) (...) So, I don't know how many minutes have already passed there. (laughter) It just happens, you don't remember everything right away. (..) Damn, I guess I, I can't think of anything yet, it's hard to come up with, that is, come up with.

..) Rather, this is how it is, somehow significant, this is health. (...) So, health. (laughter) (...) I would probably classify it more for me as some kind of sport, some kind of sport, (..) they are connected. And, (npt) I also said that alcohol, (laughter) (npt) let’s say, refuse (npt). That is, I don’t want this one (npt) yet, but even before that, this is the last one. And as for sports, I, I have a new kind of sport, that is, always, (NPT) development, I liked it, there was a subscription for eight classes. This is something new for me. But as they say, well, jumping on a trampoline, doing (npt) or something like that, in my opinion. (npt) Then work. Work, well, work, here I chose, “Yandex.Food”. I was choosing between Yandex.Food and Delivery Club, well, it seemed that in Yandex.Food, well, how to work there, on the contrary (npt), with large orders, but since I could, I had initial information... M. So, now everything is turned on, now, I hope, everything will be heard well and there will be no interruptions. L. Well, yes, I said that I was choosing between Delivery Club and Yandex.Food, and there was more difference, that is, I went to Yandex.Food. Oh, and then I also worked at Pyaterochka, but I initially went to the order area, since it turned out to be there from morning to evening, and I needed (..) evening part-time work, or for a number of hours. But, I said, they offered me a cashier job, and I, I had a choice... M. Yes, Lisa, something went wrong. Yes, about the order picker, and you were offered cashiers. Here in these words. L. Yes, I had a choice whether to agree or refuse. And I thought, okay, I’ll agree, I’ll try, why not. Although it was a little scary and exciting, in the end everything, so to speak, worked out, I was trained and I worked calmly, so to speak. Here. Later, but then I had to leave, so I quit. But I warned that I wouldn’t be around for long and, in principle, (laughter) since they didn’t quite suit me, (laughter) and it would have been a choice in general, (laughter) probably to quit. And, so, (npt) in my opinion, (..) I would roughly give an example, like choosing a vacation. Since it’s difficult for me to get from Rostov to Nizhnevartovsk, so to speak, at the moment, since the airport is closed, and you can get there by roundabout routes, but it’s expensive. Because from Moscow (laughter) to us one way it’s 10 thousand, but I just had a direct flight, I could fly (npt) when I was lucky in principle to get it and when you already flew for 3, and for 5, and for 7, then you don’t want this, well, yes, you don’t want to go one way for 10. But in principle, it happens, it’s a lot, so to speak, well, I would come to my city, but there’s not much there, there is nothing to do, and in general, my mother and I agreed that we would meet in the summer, but not in the city, but somewhere, so to speak, for us on neutral territory. Well, we went to Sochi, that is, she (npt) flew, and I had to get there by train, and... (...) Well, we generally chose tickets, well, where is the best place to go, we chose these ones, where Sochi is. But since I was already there, a year ago, for a few days, it was somehow very, there is such an example, but... (sigh) So, (..) friendly relations. (...) Well, choice. (..) Well, here, probably, who would you like to be a friend, (laughter) and who would not. Don't know. Or, let’s say, where there is a person like this, I would like someone... (sigh) (...) So... (laughter) In general, they offered me to take a walk there, and I, (..) let’s say, I didn't want it in general. I don’t like this person, so I don’t want him to be (laughter) my friend. And you probably... Well, I kind of refuse. Don't want. Don't know. (laughter) What’s a better example to give, honestly. Well, I don’t know how I could have one friend here, (..) I don’t even know. I'm moving on for now. Place of residence, regarding the place of residence, by the way, I live in a dormitory, and initially I didn’t want to live in a dormitory, because, well, it seems to me that here, well, all sorts of events are held, as if there was a place to sit, let’s say I had to film apartment or something like that. And plus I have a hostel, it’s very comfortable, well, I just won a competition, (laughter) and the hostel in which I live, it’s like, well, it’s an apartment type, and that’s why (laughter) it’s here for me in general, not only that , that I’m like this in a hostel, where I have connections, but it’s also almost home. (laughter) But, of course, I didn’t know which one I would end up in, but initially I wanted to go to the hostel, so that there would be all sorts of these events

in which I could participate. That is, renting in one there, in an apartment, is not interesting, it is boring. Here. (...) Everyday and important decisions. (sigh) (..) Well, about important decisions, everyday ones, probably just like where, where to go, if everyday, where to go, to have fun, to see. Well, also, for example, I took part in the race there in May, for me it was such an important decision. In such a major event, because it is not held here, and I have never participated. The Russian Federation race, which took place in May, I... Well, it costs money, and you kind of have to decide on it. (laughter) I took the plunge, bought it and didn’t regret it. And also (..) I liked it, I finished the race (npt) I also decided, I realized that I liked it, that (laughter) I need to train for it, and that, well, more.
M. Yes, but do you have any decisions in life that you consider unsuccessful? L. Solutions... Health related? M. In general, not only related to health, but are there any choices that you consider to be completely unsuccessful? L. Yes. The elections were unsuccessful. Well, (..) probably yes, yes. (laughter) But for me everything is connected with sports. (laughter) In the eighth grade, no, not even in the eighth, but earlier, in the sixth grade, they offered me to go to... (npt) To the All-Russian Olympiad for schoolchildren in physical education, but I refused then, and in the seventh they offered me, but I She also refused, or rather, at first she agreed, then for some reason she refused. In the eighth, (sigh) I also refused, and in the ninth, (laughter) and only in the tenth I came, and even then I agreed. And... I regret that I refused before, because I really wanted to go to the final stage, that is, to Russia, which took place then in the eleventh grade, in Kursk, that is, you go through the regional, well, first the school, then municipal, regional, and final. And (laughter) in general, two years of preparation is not enough, and if in the tenth grade I failed there, then in the eleventh, in the region, although I was a prize-winner, I took first place, but I was 17 hundredths short of Russia, and I was very I wanted to. And in general, I regretted then that I had not agreed to another school earlier. And in general, yes, and the fact that in addition to physical education there, let’s say I would also take part in mathematics, I somehow even thought about it... Well, I didn’t think about what the Olympiads are like, and what they give, and what in general is that? In general, then it was a bad choice that I refused. (sigh) In this regard. M. So you consider those elections unsuccessful, where you missed some opportunity? L. Well, probably yes. M. But out of these several situations with physical education Olympiads, which of these, well, which of these situations is the most unfortunate, in your opinion? L. (...) Well, what’s the worst? (laughter) The situation, well, (laughter) I didn’t have enough points, (laughter) for Russia, these hundredths... Hundredths, well, it’s just literally the whole point, or (npt) in gymnastics, or a second of running. (laughter) M. And you feel like you’re blaming yourself a little for not pushing through? L. Yes. I just wanted, and even more than to pass the Unified State Exam well, that is, (laughter) well, damn it, I still can’t come to terms with it. It's a shame. (laughter)
So I realized that as a result, you have not yet enrolled in any specific business specialty in order to do quests. But as for sports, why wouldn’t it become some kind of professional field, well, in which you would become, well, a professional athlete, why not this? L. Yes, it’s interesting, I mean, in general it’s, well, firstly, we don’t have that many sports in our city, and if it’s all over there (npt), then basically, in principle, then in our regions... Well, even if not only all kinds of sports, Moscow has everything, I recently made such conclusions, but in the regions, in one there is no such thing, in another there is no such thing, in a third this is not the case, and since we have a lot of sports, I started somewhere with basketball, then I liked it, before I was banned anymore (npt), then I went to sports tourism for 2 years, it’s not like It’s just mountaineering, but that’s the point, there’s a program there, on the ropes like that. In principle, I liked it, but for some reason I got tired of it. I don't know. (laughter) Honestly, I got tired of it and just quit. (laughter) The same about athletics. And athletics, it's even more boring. Well, again, it’s a cyclical sport, where you only do the same thing, and that is, we only have running and nothing else. There is no throwing or

jumping, just running. And I'm not interested in running. (laughter) It’s the same thing, in general, I’m truly not an athlete. I understand this because, well, I don't like it. Well, I don’t know, and somehow other sports, I didn’t try much, then I just somehow in the ninth, or even the eighth grade, (laughter) forgot that I was an athlete, and to some extent a sport was absent from my life, and then, in the tenth grade, I somehow remembered this, and here, when we were just preparing for the Olympiad in physical education, we have, in addition to, well, theory, gymnastics and running. And since I liked gymnastics, it’s clear that it’s not like real, professional, sports gymnasts, there’s a lot of other things, and I (laughter) by the way, I regretted that damn, it’s a pity I don’t do this, but they only take from childhood. And my mother told me that she somehow brought me when I was 5 years old, but they refused me. But I don’t remember this, they kind of told me that I was too big. (laughter) But I kind of liked it, and because of this I kept looking in Rostov for some kind of acrobatics for adults, gymnastics for adults, that’s it. And trampoline jumping, but it is combined with gymnastics for adults. But the problem is that damn, it takes a day. (laughter) Well, yes, sort of. And so, well, I didn’t manage to go to any professional sport, because I couldn’t find one for myself. M. That is, if, perhaps, when you were younger, you would have been sent, well, sent somewhere... L. Yes, perhaps.
Z. Yes, yes, yes. Here. Choice of lifestyle and leisure. I really want (laughter) for this area of activity to change for me, because over the past year, all I did was study. True, I don’t have time for anything else, I didn’t have time. I’m trying to change, I’m trying to change this moment, I really want to do... Well, in general, I would like to do yoga, go to group classes, I realized that I really like it when (..) probably a new team appears, and there are new people, meeting new people, it’s very interesting, it’s something new and unusual. I would really like this area of mine to improve, because again, all I’m doing right now is learning. And this drains you and you need to recharge somewhere. Where can I get it? Well, I realized for myself that I would like to go to a slightly different space, to other people, and replenish my resources with the help of sports, yoga, etc.
M. Okay, let's now take a closer look at your elections, and now I want to ask about the elections that you may consider unsuccessful in your life, were there any? And why do you consider them unsuccessful? Z. (...) Yes, it was a bad choice in terms of health, so I already told you that I turned to a surgeon, and... Well, I regretted it. I had an operation that was not required at all, that is, in vain, they completely removed my nail plate just like that. Why this was done is unclear. When I came to a highly qualified specialist, a podiatrist, they told me that this was a complete disgrace, it was impossible to do this, and now we are correcting this whole problem. Here. That’s why for me this is such an important choice, a health experience. And now I think this is very important. When they wish you health, now I don’t just, well, health, happiness. Happiness, health - yes, this is what I need. (laughter)
M. Great, great. Yes, it seems we have now discussed some better choice. Maybe you can remember, for example, how a bad choice was made? For example, with an ingrown toenail, maybe you can remember your thoughts and feelings while you were going for surgery? Z. Yes, yes, of course. At that time, I was at home, not in Rostov, and we went to the hospital. In general, it turned out that I was immediately sent to a surgeon to solve this problem. I come to the surgeon and he looks at me slightly and immediately says - that’s it, operate. I'm like - what? And I had never had any health problems before. Everything was fine, no surgeries at all. Well, it was a shock for me, I was admitted to the hospital right away, that’s it, they left me, my parents brought my things, and the next day I was waiting for the operation, so. So it turns out (..) day X comes, the time approaches, they take me to the operating room, perform the operation, and the worst thing was that after the operation on the nail, well, it turns out that the nail plate was completely

removed, and a bandage was wrapped around this, on this skin. It’s very tender and soft there, and it’s still a little unhealed, because an inflammatory process began from this nail, it was painful. Here. And they wrap this bandage around me and say, well, that’s it, now lie down and rest. Everything is fine. The next day it was necessary to do (..) soak the nail in a special solution so that it, the nail... The finger in the solution so that it heals. Here. And for this it was necessary to remove this bandage. I start to unwind it, but it won’t come off. He was completely stuck to this super soft, unhealed spot, it was just terrible. The time was probably just right... (..) Well, there was a day, I was sitting, I tried to soak this bandage in different solutions, a couple of hours passed, it didn’t help, we just started trying, well, tearing it off a little, that didn’t work either helped. It was terrible. We probably sat like that until very late at night, with the nurses, they tried to take it off for me, nothing worked, it hurts, it’s a super soft, tender place... In the end they told me, let’s do this to you. In general, well, it’s like a bandage, it’s even, applied to this finger, here. They cut off the entire part of the bandage and left only the piece that was glued to my finger. And in general, they tried to remove it with tweezers, pulling out each one of these... (laughter) A piece of lint from the bandage. It was terrible. It's just a nightmare. This also didn’t help, and I’m already in tears, of course, all upset, nothing is working out... I’m also a very suspicious person, (laughter) I’ve already thought of a bunch of things, that’s it, now he’s dried up for life, I’m like with him I’ll live, (laughter) upset, that’s it. Well, they told me to wait for the doctor in the morning. But that’s all that was left to do, and I ended up sleeping through the night with this bandage, and the next morning the surgeon came and in the operating room he made me some kind of, I don’t know, glued some kind of plate on me, and just tore it off with a very sharp movement. Yes, from this soft place, they simply tore off the completely dried bandage. It was very painful, oh well, and it was all bearable, it all went away, that’s it. I thought that was it, my problem would end there, thank God, everything was cured, everything is fine. So six months pass, the nail grows back, it grows very ugly, ugly, really, ugly, yellow, clumsy, all like this... I turn to the surgeon again, already here in Rostov, he says that Well, we need to do an operation and rip it out again. I think - what kind of person are you, really? Here. I’m thinking, well, are there any more humane methods of treatment, maybe it’s possible to even it out somehow? They tell me that such treatment is done only in private clinics, this is a paid treatment, but they said - try it, maybe it will suit you. And I thought that I didn’t want to suffer anymore, I wanted to know something specific from a specialist, some exact answer as to why this was happening, not just to rip it off every time, but to find out the reason. And what to do with it. And I turned to a podiatrist, in a private clinic. And now this doctor and I are still aligning my nail plate, everything is fine. Here. Of course, I was upset that I had experienced all this, but probably without this I would not have understood that it is very important to go to, well, really good doctors and it is better to pay a little for your treatment, and be sure that they will really help you and correct you your problem, than every time, as they suggested to me, tearing off a nail. Here. M. What did you think, what did you learn... What do you think, what did this situation possibly teach you? Z. (..) Taught? Well, probably after all (..) until this moment I didn’t really think about the qualifications of doctors, that is, you come to the hospital, doctors, they’re all doctors, they probably know everything, smart people. And now, you look at it from a slightly different angle, well, at least I do. I pay attention to certificates, I pay attention to where I studied, what merits I have. Well, in general, for qualifications and I think this is still very important. And also thank you that we live in the modern world, we have the Internet, we have the opportunity to look at people’s reviews, at a certain doctor, for example, or a clinic. And now, depending on the reviews, you can make a decision whether or not to go to this specialist. Here. Probably, after all, yes. The situation taught me to take health more seriously, this is not a joke, and to pay attention to who will treat you. This is also very important.

Then I went to school, and in the first grade I did synchronized swimming. All first grade. And then, the workload became so heavy that my mother suggested that I either change schools, or that I study in the second shift and be able to devote all the remaining time to training. Or stay in school and quit swimming. And she didn’t decide this for me, she said that it was entirely my choice. And then, during that period, I decided that I would quit swimming. And I'll stay in school. Well, it seems to me that then I chose friends and some kind of social life, it seems to me, at the age I was, as if friends were more important, and sports had never been some kind of meaning of life for me. I went, I basically liked it, but he was never a priority for me. But now I think that... Now I don’t think at all, but I used to think that if I had stayed, I would now be some kind of master of sports or take part in competitions, be an athlete. And life would have turned out completely differently. But nevertheless, I never regret the decisions I made, in any way. I studied the same way, in the same school from first to eleventh grade. (..) I was like that, not very much, but that is, not a completely withdrawn child, but also not one who directly craves attention, that is, I was afraid of public speaking, but at the same time I communicated well with everyone, well, I was the kind of person who He seems to communicate with everyone, but only a few people are good there. But everyone treated me well. Then, the elections, where... In principle, even when I was in school, my parents always put the whole choice on me, they didn’t decide anything for me. Which sections should I go to? I also tried a lot of different sports, only because I wanted it myself, and I left the same way because I wanted it myself. And it seems to me that this... Well, to some extent it’s probably correct, perhaps those who... Those parents who want their child to actively engage in some kind of sport, who have their own ambitions, they somehow insist, but I, on the other hand, have tried a lot of different sports throughout my life, and I realized what I like to do. I still swim, for example, like it was with synchronized swimming, I realized that I like swimming, I still go to the pool. And when I started doing athletics, I realized that I like to run, but kind of (..) not... But I only like to run, for example. I don't like everything else we did. And I just left, just doing it for myself. Well, that is, it seems to me that we need to somehow give the child a choice, although they already asked me then... (...) What... (...)
M. If in terms of the degree of difficulty of the choice, how would you rate it between one and ten? Well, you actually talked about a series of elections, but on average, how do you evaluate your passage of these forks? You chose between a bachelor’s degree and a bachelor’s degree, this advanced one, you chose not to work there, but you chose to work there. J. It seems to me, seven, approximately. M. Thank you. J. Health, I don’t even know what I can say about this. Because I’ve always tried somehow... (..) Well, I just have very good health, some kind of good immunity, I’ve never really... In terms of paying some attention to my health, I make a choice in favor of some kind of regular check-up. I go to doctors, donate blood, take vitamins, and exercise. I'm trying. Sometimes I get very lazy, but I’m trying to instill this habit in myself; in fact, I still really want it to be some kind of regular. Also, I go to the doctor, well, that is, I don’t have the position where I will never go to the doctor in my life, like some people, they don’t like it at all, no, I’m somehow very sensitive to myself, to my health. It’s better to go get checked there, once again, than not. M. And how would you rate the severity of choosing these, developing habits, even laziness, but it’s important to go to the doctors, get checked, get tested, how easy or difficult is this for you? G. Four. M. Four yeah. Thank you. J. (..) And work, for me it’s a little bit like that, for now... (..) Well, I’m still studying there in general, what suits me, what doesn’t, I don’t understand yet, I generally... At the fact that it seemed to me that I was a rather diligent person and that I could somehow do some monotonous work for a long time, but it turned out that not as long as I thought. And I’m still feeling out in general what type of activity I’m comfortable doing for a long time, because somehow, well, in my head it’s as if there should be one activity for, well, a long period of

time. But I always somehow get tired after a couple of months, I need some kind of change of activity, or some kind of development. That is, right in one place, something is the same, like accounting, for example, or in a bakery, doing something the same every day, I start to get tired and fall into some kind of despondency, which is like this it will happen, I immediately have some thoughts that it will be like this all my life. It's the same there every day. That is, I also realized for myself that in this regard I need some kind of variety, and I want... Well, my dad, for example, never worked in an office, maybe that's why I subconsciously somehow chose a profession similar to his . Because he travels around objects all the time, doing something, something like this is closer to me, some kind of active activity. Even in terms of studying, I could never sit like this and constantly listen to something and that's it, that is, when we receive knowledge, we immediately put it into practice, some kind of more creative training or something. And apparently I'm inclined to have a job, I also need some kind of work, well, more active or creative, I don't know. I'm still searching. Choosing a job is actually quite easy for me, I don't know, maybe (..) 6... I somehow start easily, finish easily. (...) Well, six, probably not, let's give five. Let's put it at five.
M. What if you give an example of some recent situation where you acted completely independently, and this situation stuck in your mind? K. (...) Well, for example, now my decision was completely independent - to get braces. And yesterday I installed them. That is, my mother told me that it's your business, do what you want. I ask her: Mom, is it worth it or not? She says do what you want. But she also seems to already understand that I often ask her for advice, and please think with me, And she seems to be starting to quietly separate me too, saying that please make the decision yourself. That is, I have personal accumulated money for work, and I myself, first I had my teeth removed, then now I got braces, this is completely my decision.
but initiative can be punished, because (.) I studied excellently, I had to, well, I wanted to participate in various competitions, Olympics and others, but I simply didn't have enough time in the day, I didn't have time to be good everywhere. And sometimes I felt bad because of this, I had periods, I don't know, now you can call it burnout, but I just felt bad, I was starting to get sick, that is, apparently this is how my body reacts, I don't pay attention to tired, but I pay attention when I start to get sick. And I just went into some kind of illness like the flu or something else, I might get dizzy, I would faint and bleed. These are the stories I have, but I just don't notice it. That is, this can affect your health, this initiative, because you are ready to be everywhere, but there is not enough time and energy.
I immediately signed up for the dances, as if to continue, because I liked it there and the team that was there, so I came and then stayed. That is, I thought that for an hour and a half twice a week, nothing would come of it, I would remain in shape, I mean physically, that's it. And in general, studying...
M. That's why you chose a different trajectory now, right? D. Well, not because of this, in general I just have a lot of problems with my physical health, I realized that I definitely wouldn't go into professional sports, well, science was interesting to me, that's why too.
And then, in short, school ended, I went to the school playground for a month, then on July 1, my grandmother and I went to the dentist, that is, also with my grandmother.
And on July 2, they left me alone. And in short, I went for a walk with my friend from the entrance. I really loved swinging on the horizontal bar, letting go and grabbing. In general, I once thought - like, how long, how long can I stay in the air without getting caught? Well, I fell on my face. And in general, it was a very cool day, so eventful, I rode in an ambulance, and then I probably walked around for a very long time with a blue face. Well, that's how my independent life began. (laughter) M. Yeah, what do you think this story says about you? How does this story characterize you? How do you think? How do you think about her and about you now? D. I don't know, it's just funny to me. M. But then it probably hurt a lot? D. No, by the way, I don't

remember being in pain at all, I just remember that I fell and lay there for a while, then I thought that I needed to get up, and I felt discomfort in the area of my face, I wiped it and saw blood on my hands. I think I'll go home and wash myself. And in our case, in short, then they were repairing the roof, and there were guys standing at the entrance, they saw me and they lifted me up to the fourth floor, called an ambulance, and called my mother. Now, if they weren't there, what would have happened? Or if these men turned out to be inadequate, what would have happened? Interest Ask. But everything turned out well actually, no concussions, nothing, I didn't break my nose. M. Yes, but what did the parents and grandmother say when they came home and found out all this? D. Well, grandma was at home, mom came running, scared of course. Some guys called her and said that your daughter is covered in blood, that's it. M. Nightmare. And on July 3rd they left you alone again? D. I don't remember anymore. Maybe. Mom works, dad works, and of course. Well, sometimes my grandmother went to the garden, probably... I don't remember. But I'm still living, so it's okay. (laughter)
M. Did you end up in the hospital because of this? D. (..) No. But... Well, yes, I was at Sirius this summer, and in short I was slightly ill, I had a sore throat and a runny nose. Well, I didn't go right away, but I went to the first-aid post, they prescribed antibiotics, I took it for 4 days, that's all, I didn't go to the first-aid post and then say that everything was fine with me. I just didn't want to go there anymore, that's it. And in the end, after 2 days I felt very bad, I could not tilt my head, my sinuses hurt, everything was wrong, I was afraid that I had sinusitis. I had sinusitis, that's it. And I decided to go. They scolded me very much there, they said that this was impossible, they took me to the hospital, in the end they wanted to admit me, but everything worked out, and in the end I was on antibiotics for another two weeks. That is, half of the entire shift that I was there, I was on antibiotics. Now I have stomach problems because of this. And all because I didn't go and tell him again that everything didn't work out for me. Here.
M. Is someone else watching him, do they remind you? Or, well, just... D. Well, they remind me, but they don't make sure that I'm taken right by the hand to the hospital, because after all, I'm already 17 years old, I'm not a child. Here. And even more so, there was such a situation that since childhood I've been in hospitals, I don't know, I've had a lot of different things, I also have tuberculosis, some kind of problem, that is, that I have this mantu and Diaskintest are always big, something was stupidly infected there or something like that. She was registered. Then a cardiac surgeon, oh, a surgeon, (laughter) we also went to a cardiologist, I had problems when I started playing sports normally, in the fourth grade. Then, when I started playing sports even better, in the seventh grade, I walked like a disabled person because of my knees, and we also went to another city. Well, in short, a lot of things. And this is not a complete list. That's why I'm very tired of this, I don't really like going to the doctors and wouldn't like to at all, but somehow I wasn't particularly lucky with my health, to be honest.
M. Did something happen then? L. Yes, yes, yes. And there... She fixed herself in this position. M. Well, somehow you can ask the question - what? Or don't want to answer you? L. It's possible, it's possible. I worked through my injuries, you can ask. (laughter) In my family, well, like in a family, there was a stepfather, a case of domestic violence that lasted for 6 years, from 10 to 16. Here. And it was very difficult to fight back, I was little, then I grew a little, so. And at the final point, well, at the age of 16, I immediately understood that I had to do something, somehow fight for myself, well, yes. And, thank God, it worked out, as they say. Yes. And at that moment I immediately realized that oh, oh, oh how you have to be independent (xxxx). (laughter) Oh, oh, oh, how necessary this is. And right then it was clearly formulated and it didn't go back from that point, thank God. M. But you moved after 3 years, as far as I understand? L. Have you moved? M. No? L. But no, just, well, it turns out that I have my dad, wonderful, beloved, but we (npt) when I was

7. Then I had a stepfather, from 10 to 16, not a very good person, (laughter) Here. Now my mother is also married, but now I have a wonderful stepfather, I respect him very much, I love him, I appreciate him, he is kind, caring and, well, my dad too. Wonderful person. I don't know what happened to that person. And I already moved when my mother was... I already had, now it turns out, a current stepfather, I already moved away from them. I didn't have it there, but everything was there (npt) it all ended just then (..) then, my last trial was at 16 with him. That's all. So we (..) said goodbye and... And I directly cemented my position as an independent lady. M. I'm very sorry about domestic violence, of course, and I'm glad that you and your mother are no longer there. If I hear correctly. L. No. M. And you came up with a plan? L. I'm now... Now I'm (npt) taking pictures of the experience, it was yes, thank you, I might not have been so strong. Who knows.
M. Are there some things that you prefer to hide from your parents, maybe you don't tell them from everyone? You mentioned tattoos, but that was quite a long time ago. L. Yes, well, what can I say, now I wouldn't hide it, now I would come proudly (npt), to my mother and say - here it is, new. I just don't want to. I guess sometimes I can hide some of my emotional anxieties from my mother, because in general I'm such an emotional person, I can be sad, or just emotional... The only thing is, I try really hard, but I never succeed straight hide it from my mother, when I catch a cold there, or something like that, in short, it happens, I know that she will be nervous, and I don't like it so much, and I'm always trying to do something, somehow - yes, everything is fine , Fine. (laughter) These are things, yes, I try not to tell my parents, because they will worry, well, that is, I don't know, ordinary ARVI, but mom already - oh God, you live there alone, who will give you tea? do? Now, well, that is, these are not some kind of terrible things, I just (npt) don't want (npt) only dad. No one will lose anything from this, I just don't know, they will save 1000 nerve cells by doing this (npt). Without thinking about the tea I made for myself or not.

3.5 Table 5. Self-care practices (types of practices)

**Citations**
Yes, it turns out that after that I trained for three more years, I also went to different competitions, in different cities, I already understood, well, since I refused that chance, that chance, a second one most likely won't come to me, but I've already trained, let's say So, semi-professionally, I played for our academy, CSKA, but I (.) didn't dream of going there to some Wimbledon and so on. And one day, when I went to a competition in Taganrog, I was fighting for first place, the last one turned out, the last game was for first place, and I fell on my back, so. I injured my back, then I couldn't walk at all for a week, I was thinking that it was a fracture, or that it was a bruise. They took a picture and said that it was just a spinal bruise, well, it would go away soon, but after that I already quit tennis, because even after rehabilitation there, about six months, when I returned to tennis, the indicators were completely different and, accordingly, , guys who were plus or minus my age, they continued to increase the pace while I was rehabilitating there and so on. Well, basically, I was, roughly speaking, no longer needed, even in this, at this level of tennis, and I decided to leave.
Yeah, health. Until I was 17 years old, well, I was an athlete, and so on, and so for a very long time, well, until I was 17 years old, I always told myself that there is no smoking, no drinking, I will never be there, it's not for me, especially for me Dad smokes there, I think no. But then, at the age of 17, after (.) the death of my friend, my second, I started smoking. I still smoke to this day, I reacted at first... I still have thoughts, why did I do it? Maybe it's worth quitting? But then I think that, well, for now this, for example, brings me pleasure and some kind of outlet, in its own way, so I think for now I'll make a choice in this direction, and while it develops in this way, well, I'll

drink there, I don’t know, there’s a glass of wine on holidays, roughly speaking. That is, not a fan. The choice to start smoking was conscious; I purposefully wanted to try smoking and start smoking. That is, I don’t know how to describe it, one day I think - I want and will smoke, and that’s it. And I kind of tried it, and that’s what I wanted. M. It worked. M. Yes. (laughter) That's how it all happened. Therefore, the choice was also probably easy, I probably don’t really regret this choice, well, although sometimes, perhaps some thoughts slip through there again, but in terms of complexity this choice was, I also think there were about three or four, probably maybe, something like that.
M. Yes, I think I’ll quit, to be honest, now I’m already trying cigarettes, I don’t smoke, I’ve switched to alternative, let’s say, methods of obtaining nicotine, like IQOS, well, for example, something like that, it’s still unknown of course, which is less harmful, but at least warms the soul, which they say may not be so harmful. And I think yes, and I’m gradually starting to come to the point of quitting smoking, completely, because I’m starting to realize that very often I smoke there, when, for example, I’m either bored or need to occupy myself with something there, any stressful situations, and so on. And when this is not such a strong need, then it is quite possible that this can be easily abandoned. And therefore, I think soon (.) I will quit. Here. Anything else to say on this topic? Or (.) can we move on?
M. How do you like it, how, I don’t know... M. Well, I decided for health reasons, I think it’s necessary in principle, all my life I was an athlete, a tennis player, I tried a lot of sports after tennis, then I tried again, football, volleyball. .. M. As far as I remember, swimming, you said? M. Basketball, swimming, water polo, billiards, if you can call it a sport, I tried a lot of things, and when the time came for college, there was much less time for such activities, but I decided that at least just go to the gym there for I need some kind of general health, some kind of physical development, so I went to the gym, probably already about... (..) Well, I’ve been going for about a year now, (..) every week three times. I also made this decision quite easily, since I, in principle, connected my whole life with sports, some kind of unnecessary activity in my life, on the contrary, it was only a plus for me, my desire for this was there, therefore, too, the choice to go there was rude speaking, go to the gym, don’t go, one, probably in terms of difficulty. Or something like that. Well, I probably can’t say more about my health (..), well, I think there’s nothing to highlight.
As a result, I smoked from the second to the fourth grade, that is, I had been smoking for almost 2 years, being generally a schoolboy who had his head on the table (npt) himself because he was running. But it turned out that I had to quit for two reasons. First, and most importantly, health problems began. I’m sitting there, this is all for me, I’ve played enough, I won’t.
And in the end, the actions reached the point where the teachers all scolded me for a very long time, and were about to label me as, well, like, not a street child, but whatever it is, well, in short, register as a child. Here. But there it turned out to be somehow simple, I was simply put on the internal school register. And they said that if something happens again, then we will have the same horror. A little time passed, a week later the fight started again and then they started beating me, probably about seven people beat me, on the street, it was in winter. But at some point, I kind of just lay there angry, I don’t know, something confused me, I abruptly shoved everyone who was standing, fought off with steps, quickly jumped up, kicked someone else there and caught up with one, and just I took the snow that was lying, and then there was some kind of ice and I decided, I thought that I would just wash his face with snow, it so happened that I took a piece of ice and it accidentally hit him in the temple. Nothing terrible happened there, the ice just cut the skin a little, that is, not even a cut, it was so strong. But in the end, I was still scared too, that’s it, I won’t go to school anymore. Let's go to some doctor and say that blah blah blah, let's check the child, I'm there, I don't remember exactly what happened. Well, in short, the doctors there determined that there

was some kind of prolonged depression, they prescribed the child, well, fifth grade, antidepressants, and they forced him to sit on antidepressants. I spent a long time on them, on the course, I don't really know, me, well, not systematically, but at times, my mother seemed to say to them - let's continue to drink this. And these were, I really don't remember, either some very mild tranquilizers, or antidepressants, I remember... They were probably called something, I've already forgotten. But the point was that my studies failed because of this, I just stood at the blackboard like a vegetable. I didn't have a single thought in my head and the teachers often looked sideways at me, and to themselves, that is, like this drug addict, to the narcologist... What's going on there. Carefully. I'm sorry, the girl dropped something. Here. We went to a narcologist, where he told me that, buddy, everything is fine, they showed him a prescription for these antidepressants, and for a long time they refused. But somewhere around the eighth grade, I realized that there was a huge difference in interaction between me and my classmates, that is, more and more of the time that I sat at my desk, I simply watched how my classmates' adolescence was passing. And at some point, like in some "Rocky," I stood up in my room and said - I won't drink, that's it. I refused. And somewhere from the eighth to the ninth and eleventh grades, I began a time of real rehabilitation, when I, well, there my first interactions began, just like those who were more mature. Here. There's a story with friends, one there, others, the fourth, it still didn't work out with the girls, that's it. But somewhere around the tenth or eleventh grade, I was already completely rehabilitated, both as if just on my own, and in the eyes of my peers, who saw me both in the sixth and seventh grade. And we kind of fell into the same sort of path, that is, the same groups, and on our own we seemed to communicate normally. And this, this dynasty that I carried with me from the bad events that were covered by my pills, somehow left. Then it so happened that even by my first year it all continued; I began to experience a simply stormy, simply huge, stormy life as a freshman. And I slept, it was so that I slept at night every other night, one night. That is, I worked in the morning, afternoon and evening on projects at the university, at night we went to parties, the next day I still worked in the morning, afternoon and evening, and at night I slept. There were nights when I didn't sleep, not because there were parties, but because I didn't have time to complete projects by the deadline, and I also had to work at night, because, of course, there were some situations, they just calculated it wrong , one of the teammates did not fulfill his duties there, and so on. Here. And I, as the leader of the project, just needed it to be completed. No matter what a person does, I will try for him now. Here. And my classmates, whom I later met, joked that I was speedrunning my life. Speedrun, this is true, just in case, for those who will decipher this later, this means accelerated, accelerated passage. That is, among the players there are those who compete to see who can complete the game faster. This is called speedrun. I was told that I was in the speedrun of my life. That is, in terms of (..) everything that they felt there during the entire time when they studied there from the fifth to the eleventh grade, I felt literally in 3 years, and even further, what they had not yet had time to experience, I already felt it. That is, I was carried away somewhere there.
M. Ilya, very detailed, thank you. In general, I'll start with your first phrase, when you started speaking, you said that reflection is always welcome. And as if from your story, it also came up a couple of times that you have, as it were, experience communicating with different psychologists. But tell me, tell me, are you, in general, inclined to reflect on your own, or how did this experience influence you, why are you talking about yourself in such detail, analytically? I. It's like two in one. Like these non-cafés. But it turned out like, the story itself from the 8th grade there, when I came off these antidepressants, well, it turned out that in order to understand this, it was necessary, of course, to reflect there. I have minimal communication with a psychologist, well, except for this psychologist I just talked about, 27 years old. Because every time I went to a psychologist, it ended, well, there were one, two, three or four trips. And they told me what I already know. And most of the psychologists that I just went to said, "I don't need to go to a psychologist, I calmly

delve into my own head." That is, I will scatter if I can. The only times when I actually went to a psychologist with benefit was when I went for IQ tests. This was when I went, I sat there for an hour with him, he solved the test, then yes, this was my only useful trip. And the rest, if they didn't just interfere, somehow changed life for the worse. Although it is also unclear which one, good or bad. Probably a good one after all. Then the rest were useless. Well, it probably turned out that I myself am a reflective person, I even think at the Skolkovo university the moderators told me that at times I was even hyper-reflective, let me look for meaning where there is simply none. (..) But it so happened that the element in which we learn, it implies reflection after the end of each activity, project. Necessarily. That is, we have to sort everything out, what, why, but they didn't teach it in a very good way. But when I practiced with the Skolkovo team in game technology, a prerequisite there was, as it were, reflection itself, game technology, and in detail, that is, I had to write a plan, with the tools that I used, that is, I said what I want to try on the guys there such and such, such and such a method, there is interaction between them, maybe there (npt) according to Weldon a couple of people there can move from an idea generator to an executor, and the like. And then come back and write a reflection. It didn't work out because like this, like this, like this. You need to try this, maybe something like this. So much so that I needed to read the reflections of those guys whom I sort of moderate myself. Therefore, it's like I'm reflective myself, and it so happened that I also needed to practice this intensively.
M. But I'll immediately clarify this episode, when you already felt that you, well, smoking began to affect your health, and now your parents burned you, this moment, in general, you wanted your parents to burn you in these activities, or did you not care at all? I. On the contrary, I wanted them not to burn me, (laughter) to receive this strongly, and then for them to know, in principle. Yes, it's just that my father, in my earlier time, probably around the sixth grade, had big problems with alcohol, very big ones. (..) And there it got to the point where I was just sitting, he told me - look, cigarettes, smoke the whole pack there, so that you can get more, so that you feel that this is not the same, and don't smoke there anymore. Well, the like. Well, basically, since I was in school, my father probably doesn't drink anymore. That is, I don't know how many years have passed there, maybe even 7 years for sure. (..) Yes, in general, it's been more than that, probably almost 10 years or something, since my father doesn't even drink anything strong. Well, similar. He didn't even seem to imply that smoking a pack of cigarettes would just be like, well, a pleasure. Here. But no, I didn't want my parents to burn me at all, it happened by accident, a classmate burned me, that is, she complained. Oh, I forgot to specify, so that my parents wouldn't burn me, I sprayed cologne into my mouth. It was an old children's cologne, I don't remember, it was unlikely that it was triple aged, because it was a regular children's cologne. But it was definitely alcohol-based, and I even remember it was "Pirates of the Caribbean", then these colognes were also coming out. And I just poured cologne into my mouth. Here. It was terrible, but what can you do, you had to make some sacrifices.
About health, with health... (..) The story is that, let's say, as soon as I received normal money, I finally went to have my teeth treated. I had a big problem with my teeth and I passed, then I took a genetic test to check for some congenital diseases and discovered several congenital diseases. Here I would not say that I was unlucky, on the contrary, it was good, I saw there and saw some predispositions in terms of hormonal ones too. I went to the endocrinologist and there they also developed, drank some zinc and so on. Here. Well, plus I had a dental operation that didn't go very well, they pulled out a tooth, it turned out to be the sixth, chewable one, and he had a cyst on the root, and the cyst ate a layer into the sinus. And now between, well, where the tooth was, I have a hole in my sinus. That is, such a good message, air can go there, back and forth from the sinus to the mouth, water can go into it, so. And this, in principle, can only be healed with an implant. This,

well, with health, it means that it was necessary, well, it was necessary, well, most activities were prohibited. But now, for example, it’s literally been a week here, and after work I’m going to go, well, recover in the gym. Because now I couldn’t go to the gym for a while.

M. And now there are those moments on which you consult with your parents? I. (sigh) (h) (...) Well, probably for health reasons, I always consult with my mother, because she is a nurse. Here. That is, if I suddenly start to get sick there somehow, in a way that I have never treated myself before, that is, if I start some kind of ARVI or something there, I can basically cope with it myself. Here. And when it comes to some symptoms that I haven’t had before, I call my mother and talk, if they don’t help, then I go straight to the doctor. Here. (..)

M. I hear some kind of conflict between what you think and how you act. And how is it generally given to you, is it easy to make a choice in such a situation or not? That is, why do the scales still start to tip in one direction every time? M. (sigh) It's hard to say. Probably because... (..) How to say... Well, again, probably there is a point in psychology that people often treat others the way they want to be treated. And among other things, I give people, other people, a chance because I would like to be given a chance one day if I stumbled. This is exactly why I give people there some kind of warmth and so on, that is, everything, all these moments, because I myself lack it. That is, what happens is, let’s say, the opposite situation. That is, I give what I lack. Here. M. But this is generally interesting. How did you come to such an understanding, to such an experience? And to your own behavior? M. I unconsciously stumbled upon all sorts of psychological things a lot. (NPT) I unknowingly came across all sorts of psychological things, I don’t know, because you scroll through your VK feed and there, for example, there are some posts about psychology, something else, in the same TikTok you come across a lot of all sorts of psychological analyzes, oh behavioral types of a person and so on. So it all stuck to me like that and, in principle, some kind of awareness came. And that includes still coming. Here. And so... M. So, that is, you are not a naive respondent, right? And pumped up.

M. As if your experience of depression was for you also such a marker of where my friends are and what choices can I make in relation to them? And as if you, despite the fact that you are used to giving people a second chance, decided that you will not give them this chance? M. Well, I just, again, it seems to me that I have some kind of innate probably psychological things, well, that is, I can read people to some extent, I understand how they behave, how they will behave throughout to lead for a certain time, that is, it seems to me that I have some kind of psychological habits in terms of reading a person and so on. And all this. Understanding other people, including yourself. And based on this, I probably, well, probably don’t give these people a chance. That is, I understand that they will not change.

M. Well, yes, it seems that you hear yourself very well and understand what you need and what your needs are. Tell me, with your activities, what you called a hobby, did you also listen to yourself or perhaps it was imposed by your parents? M. Regarding sports, I probably always liked to do something like that, to feel some kind of power over my body, that is, when you grow, including over yourself. Including remembering, as they say, (laughter) the classics of fight club, development through self-destruction, that’s also a wonderful thing. This is also wrong, but I probably came to this too.

M. Well, great. That is, in addition to your profession, you also have a hobby that gives you pleasure. And you seem to have mentioned something else in your hobbies, music, right? M. Yes. Yes. Here. Well, it’s more... There is some kind of story in me that I want to learn how to play well, and so on, but I’m probably too lazy for this, and even more so, it seems to me that I don’t have good potential for this . That is, I can play some banal things there, play something on chords, sort of a little strumming, that is, I can play, and... Well, it’s not like I studied there, that is, it’s more by instinct it sort of went, I liked it there, how I typed some kind of melody, I started to

continue there. Here I am... M. How old were you when you started playing music? M. (sigh) (..) Oh, probably 18-19 somewhere. In this regard. M. And it was also your choice, right? Or... M. Yes, yes, it was my choice, completely. It's just that at one point I realized that... Well, again, I'm probably a depressive psychotype, I don't remember, if that's what it's called, so. And I bought myself a musical instrument simply because sometimes I'm in such a state that I just want to sit in the dark and strum something sad to myself. This somehow makes it easier. M. But it's cool that you have such a resource that you know what to do in such conditions. M. Well, before, I used to, as they say, beat myself up with sad music, but still doing this, as they say, this is probably my favorite self-flagellation and so on. Well, it's probably not self-flagellation, it's probably just some kind of self-harming. Here. First I bought a ukulele, realized that it didn't sound sad enough, and bought a guitar. (laughter) Here. There's more bass, more sadness can be created, let's say. Here. Well, sort of in this format. M. Yes, it's interesting, that is, you can take this sadness out from the outside and create it yourself. That's great too. M. Yes. (laughter) M. No, it's really great that sadness comes out of you and is expressed in creativity. This is great. M. Well, including... Oh, yes, by the way, I remembered another hobby, you said, expressing yourself in creativity, also not so long ago, a year ago, I think, I started writing a poem, here. Also, as they say, sadness had a very strong influence on this, along with depression, so. Because there is not a single funny poem that (laughter) I wrote. Because, it seems to me that the strongest emotion is the worst emotion. And based on this, here comes, well, let's say, what I do.

M. The choice of a doctor, the choice to seek medical help or not. M. (..) (h) Probably (..) (laughter) two or three. Here. Because, in principle, I practically don't get sick, I'm a follower of a very strange method, the one that does everything on its own, but there is such a joke that a man goes to the doctor only when a piece of a spear in his back prevents him from sleeping. On the back. Here. Therefore... I probably have a lot of them, by the way, yes, it will probably be important to record that I have a lot of things taken from my personal stereotypes that I have formed of what a man should be. A lot of things come from this too, that is, I literally have a list of men there, too, to be like this, like this, like that. That is, well, I understand that stereotypes about men, in principle, stereotypes are a very disastrous story, but I myself, here is my list, (laughter) I should be like this. That's how it is in this regard. This also affects a lot of things in my life, so... Yes, regarding health, in principle I probably don't really value my health, so it's easy for me to make decisions in this regard. Here. I kind of understand that I do sports there and so on, all the things, but at the same time I can drink there calmly, well, I try to know my limits and so on. It's as if I understand that if I drink a lot, then (laughter) I will die in one moment.

M. (laughter) Yes. Here. Therefore, I probably don't particularly value my health, and making decisions here is quite simple for me, I still try to control my health and so on, in the sense that, well, I try to see something in myself if something hurts me and so on, but otherwise, it's as if I've never had such a thing that I have to go straight to the doctor, that is, it seems to me that I'm more than healthy. I recently took tests, and they told me that they could at least launch me into space.

A. Yes, I work in an IT company, well, it's an ordinary development studio, I'm a backender. M. Tell me, do you have any hobbies, interests, besides study and work? A. Yes, my scientific interests are research in the field of education, I am mainly engaged in the history of the philosophy of university education. Psychology a little, I also do origami, sports are athletics, that's it. Just like that. How long have you been doing sports? Two years. Well, intermittently, I did a lot of sports as a child, well, I consciously came to this (npt).

Health. Health is the area that suffers greatly in my life, I'm sick right now, right now. And I recently took a Covid test there, yesterday, and I'm waiting to see whether it will happen or not, but it seems that it won't, I already feel fine. But, damn it, I haven't been doing much about my

health lately, but I just support it with sports and that’s it. But I would like to undergo some kind of examination, the fact is that the right side of my head has been hurting all my life, and it’s such a noise, it’s just as if, I can describe this pain as a noise. I've already gotten used to it, because I know that damn it, this never happens, once in the tenth grade I did an examination that did not give any intelligible results, they told me that I was healthy, I'm into it I don't really believe it. (laughter) Here. Still, something is wrong. Otherwise, health...

Health - ten, it’s very difficult to make a choice, I have some kind of masculine fear, I don’t know, or in general it’s a universal fear of going somewhere, or arranging an appointment with a doctor, going there, I don’t know, to the dentist once every six months. Well, in general, it’s just some ten, and I don’t know what’s stopping me.

Damn it, it’s difficult to take up sports on a systematic basis

A. Oh, you know, this is very interesting, I had no thoughts. Well, that is, if we are talking about some kind of rational reflection, and I mean perspective reflection, that is, when we assess risks, when we, we don’t know, analyze a situation that could happen, it in general (xxxx) (laughter) there was none. Well, that is, it’s purely based on some kind of emotion, some kind of euphoria, it’s probably possible, I didn’t use psychoactive substances very much, I smoked marijuana twice, but it seems to me that this is something similar to what people experience under psychoactive substances, such an absolute frenzy and absolute lack of self-control. Here.

Next, health. (..) Yeah, health, health. Here, you know, there is a rather difficult aspect, because for probably several years my health has fluctuated, (laughter) let’s say, like the exchange rate of the ruble on the stock exchange. Probably until 9-10 grade, I was quite (.) a chubby boy. And I probably made the choice in favor of a healthy lifestyle on the basis of some, let’s say, personal oppression and personal motivations. But for the most part this was due to teenage conflicts. And, accordingly... (..) Any problems. M. Did someone offend you? I didn’t quite understand about oppression. A. Let's just say, (..) at some point it began to seem to me that at school, on the part of friends, on the part of teachers, at some sharp moment the attitude towards me changed. That is, they stopped noticing me and (.) paying any attention to me. Although I didn't seem to do anything wrong. Nevertheless, I didn’t really understand what happened, but at that moment it was probably quite difficult for me to communicate with people, because I didn’t understand what this attitude was connected with. That is, if I had a teacher with whom I was in close contact, and (.) with whom, in principle, I was conducting some kind of project activity, then at that moment it happened, I don’t know how some day came and as if at the snap of a finger What happened was that this person’s entire attention switched to another audience. And essentially, you know, it's like a child who has been sharply deprived of attention. You want it, but they don’t give it to you. And I probably began at that moment to look for disadvantages in myself and in my appearance, in my health. I began to think that this was probably connected with some characteristics of my body or characteristics of my thoughts. And then I probably started soul-searching. (.) Therefore, the choice in favor of health, it was probably given to me, let’s say, through a battle, due to some experiences, through an attempt to prove to myself and many others that everything could be different, that I can do something... something else. Therefore, in terms of severity, probably (..) well, let it be a ten. At work... M. So you connected this deterioration in your relationship with the fact that you somehow gained weight, or somehow didn’t look the way it seemed right, right? Because you were talking about the love for sweets, and about being chubby, you said? Or did I misunderstand you? A. Well, for the most part, I’ve been pretty chubby since childhood, and I probably stayed that way until the 9th grade. Therefore, purely physically, I didn’t change much, due to weight gain or loss. There was simply, let’s say, one consistency of the body, which was maintained as it grew. Therefore, I don’t know, I probably connected this not with an increase or change in weight, but with the fact that at some point some skills and shortcomings that people did not want to take into account

simply became more noticeable. M. Did you say something to you, did you somehow become yourself, did you say about choosing a healthy lifestyle? Something like this. A. Yes, I decided that... The Unified State Exam had just ended... the Unified State Exam, in the 9th grade, I thought that since such a situation was happening, I needed to rethink myself, I needed to somehow start taking care of myself, to show myself, well, to change something in myself, not only mentally, but also physically, so I probably made the choice in favor of a healthy lifestyle, in terms of athletics. That is, I got up at about 5 in the morning, slammed the doors, everyone was scared of what was happening so early in the morning, and I just left the house and started running at the stadium, ran to some park and spent my time there.

I really don't like this, so I'm trying to gradually return to my usual way of life. And somehow connect sports to your activities. But for the most part, probably due to my (..) fear of people (xxx), some kind of tightness, I don't always succeed. That is, I cannot, relatively speaking, sit in a hostel, do something, get up and start doing push-ups there. Or, I don't know, stand in the plank. Because, (.) you know, this is an internal experience when you look stupid in someone's eyes...

M. Well, yes, I understand. A. Yes, for the most part that's why I got up early in the morning and ran away earlier, to get away from prying eyes. M. Well, yes, very clear. A. Now, well, basically, what you can do is go out late in the evening, we have a small park, a student park, and do a few laps of running there if you have free time. If not, then I try to diversify my diet and make it, let's say, less high in calories.

M. Interesting. Do you have any hobbies or interests? I. During training, my hobby is only sports. M. What kind of sports do you do? I. Basketball, but without any activities, just by myself.

And so, if your hobby is sports, crossfit, cycling, snowboarding and generally active recreation.

A. (..) Well, in general, in order to get straight on where to go, they told me banal things about don't drink, don't smoke, love your mother and everything like that. But the smaller parts, somehow I don't even remember this, but it's already on my mind, that is, not to forget our neighbors, those who are nearby, to help them not to forget. Here. These are the things. And if we talk specifically about independence, then somehow... Well, I don't even remember, they just said that you will grow up, you will be independent, you will do everything for yourself, cook soups and all that. Here. Well, they just taught me and I remembered it, I put it into practice and then I cook borscht for myself now. Here. M. Is this specifically about borscht, about this kind of food, or about some other things too? A. Well, besides this, yes, for example, hammering the same nail into the wall is understandable, my father taught me this too, it was also not without bruised fingers. Here. So... Besides this there is a lot of other things.

T. And I remember myself probably from the age of one... Well, probably from the age of six, from five, somewhere like that, directly consciously, so to speak. Well, as a child, I initially wanted to become... (..) To become a policeman, that is, at that time, a policeman, and, in principle, my parents did the same, if we talk about independence, that is, they always signed me up somewhere, to some clubs, some sections, that is, since childhood I was kind of fond of sports, that is, I was such an activist, that is, both at school and in some extracurricular activities, social events, that is, always for the most part, they attracted me, too, and (..) it seems so, and some leadership qualities were probably emerging, so this is where the roots probably go, maybe some of my independence in making some decisions. That is, as I have already said, the sphere, so to speak, of my activity is quite wide, that is, starting from the public... That is, this has been going on since childhood, that is, now I, let's say at work, am the chairman of the council of young scientists specialists, these leadership qualities that have been going on since childhood also manifested themselves. And I finished school, that is, until the eleventh grade, passed the exam, and entered the university. At the university, of course, it's also a little less, since after school, for some reason, I began to devote

more... More time to studying, not in the periphery, but specifically to studying, and therefore, a little less, I had classes that were, so to speak, excellent from studies. But still, no matter how I gave it up, I also took part in all sporting events and social events. So I studied and studied, and naturally, after that, after that, I went to work. And at work, from the first, as they say, days, that is, he showed himself as a fairly active person, went out with his colleagues somewhere, that is, he was always, so to speak, easy-going. And, that is, after that the management noticed and, as they say, I am still, so to speak, in plain sight. That is, just like the leadership, as it was before, like at the university, like at school, and now, let's say, the strategy can be action, that is, just independence, leadership qualities, and so on, then are present at the moment. Here. In principle, I think, more or less answered. (laughter)
T. Well, I have this character trait: sometimes I follow the majority. That is, in order, as they say, to go against the flow, namely, my closest circle. That is, my environment was quite like this, just sports, that is, we were engaged in dancing, and naturally this was vocals. I was, I looked at other people, I looked at how they were, whether they were achieving success in this regard, and then I tried it on myself, whether I was even interested in the same activities that were popular at that time, and I already made a decision. And if, so to speak, in percentage terms, they probably have their own opinion and people's opinion, well, in general, the opinion of the current, the opinion of society, so to speak, is probably about 50/50. Well, maybe 60/40, in my favor. In this plan.
M. In general, well, now it turns out that you are discussing with someone some things that may be difficult for you, well, be it some psychologist, I don't know, a close person? T. Well, yes, there were moments, that is, when I visited a psychologist, that is, in principle, there were moments when I was just put in a leadership position, and I, having worked for only 2 years, yes, about two years , one and a half, in general at work, that is, I was put in a leadership position, naturally, there was stress. In general, there was a lot of stress and I needed some kind of psychological help, (..) it turns out to be qualified. Yes, I also consulted a psychologist. And in principle, this gave its micro-fruits, that is, well, perhaps, including the fact that I have already gotten used to it, that is, this so-called adaptation period has passed, and now I am more or less in a normal, so to speak, psychological rut and everything is fine.
M. And if we talk about health, then when you make decisions in this area, what helps you do this, for example, a consultation with a doctor or how do you even choose a dentist? T. Well, it turns out that in this regard I'm probably suspicious about my health, that is, if some kind of sore pops up, I start there, well, not exactly panicking, but it's faster, faster to take some action to remove this pain. Just with teeth, and everyone knows that this is a rather expensive procedure, namely dental treatment. And therefore, finances did not quite allow, or rather allowed, but there were other, well, one might say, demanding, that is, aspects that required an urgent investment of money. And that's why I put off just about the teeth. Here. And therefore, in principle, since I have already said that I am suspicious, I quickly, quickly tried to fix all this as quickly as possible, so as not to start all this. Then don't pour in even more finances, already eliminating the consequences of all this. M. But I'm even asking more about how you understand who is a good dentist there and who is bad, about this. So, where do you get this? T. Well, here again, already through acquaintances, that is, I found out from people who treated there, and about prices, and so on. There was no such thing here that, yeah, I typed dentists there into Google, and went to the first link that came up, and immediately went there. That is, naturally, at first there was some (..) research, so to speak, on this matter, that is, I found out from friends there who was doing good work and, accordingly, I went there.
T. Well, in principle, all my successful decisions, which were made at the junctions of the most important stages in my life, were at school, that is, the choice, I don't know, not just there, I don't know, walking around the entrances, smoking and so on, have a drink there, namely, go in for sports, that is, some kind of social, some kind of personal development. At the university, that is, to

enter exactly the university that you are now, well, in which you naturally studied, (..) it is precisely the choice of the field that is most promising in our country. And at work. That is, I work in a fairly large company and this, in principle, makes me happy. I don't think that somewhere in these probably most important stages for myself, in my life, I made some wrong decisions.
T. Well, yes, that is, before, I'll interrupt, I'm sorry, I just had a problem before in that sense, that I, well, when you see a cat trying to attack a mouse, that is, it first prepares itself there, sits for a long time, waits, and at some point pounces. I've always had this in anticipation and preparation, just before communicating with a psychologist and so on, that is, I've always had this for a very long time, that is, I'm trying to calculate every little thing that is impossible to calculate, and sometimes just you miss your goals. This is exactly what is needed, it seems to me, we simply mean to wait, but not to wait out in general, roughly speaking. That is, to evaluate yes, more or less seriously, (npt) you act as you see fit. Here. Something like this.
I. So health... What elections, elections, elections, elections, elections, well, it seems to me that with my health pah-pah-pah, everything is fine, I try not to get sick, (laughter) here. It seems to me, well, if we take health in general, well, my mother just had a stroke there. I had to make certain choices there, I don't know, about apartments, about inheritance, and so on and so forth. This all also falls on me, in any case, so for me, well, it was hard for me, it's still hard for me to resolve all these issues, because in many areas, with many of these things, I'm meeting for the first time, there the insurance company, the hospital, I don't know, they have to process certain documents, it's incredibly difficult for me. As a child, my health was bad. As a child, after childhood bronchitis, I had very severe bronchial asthma, I was sick for a very long time. I rarely, I didn't go to kindergarten at all, and I went to school, but I was sick very often, of course. (..) It was very difficult for me as a child, so it seems to me that I would rate my health as exactly this, I would rate it as an eight
when everything seems to be bad, but I always keep the thought that life, this is also such a clichéd concept, that life is like a zebra, that is, there is a black stripe, there is a white stripe. And I began to notice it too, it seems to me that maybe this is self-hypnosis, maybe, I don't know, it's more like you, as a psychologist, will say. (laughter) I also began to notice that yes, there are some lows in life, but there are always ups and downs in life. And... (..) And the same goes for good and bad things, for me too, that is, I have neutrality here, but in life, yes, there are some good and bad moments, just even bad ones moments, I never call them bad, I just think that this means now is the time, some kind of quiet, calm life, or doing nothing, or I don't know, it means that life is telling you - rest there, devote time , I don't know, some family issues, don't engage in public affairs. There and then next week you will have five new acquaintances there and ten new projects will arrive. And this is how it usually happens, I don't know, I was there (..) about two weeks ago, I think somehow, and when autumn began, probably at the beginning of September, I was just moping, moping, I think - nothing It turns out that it still doesn't seem to work and it seems like my colleagues and friends also have problems, somehow they need to be helped, no one helps them, and I can't help them either. And now, October has arrived and there are just so many different projects and you're thinking, how can I get rid of them as quickly as possible, from all these projects, because somehow they all just landed on your head, and you don't understand what to do with them? . (laughter)
M1. What was prohibited, damn it, no such thing actually happened. That is, I don't know, well, it was forbidden to steal, yes, to do something else, but no, even some kind of morality, alcohol and cigarettes, we just don't seem to discuss these topics, in general, there was no such thing as it's impossible, smoking is harmful, I don't seem to know, through conversations maybe, through some other information, I realized that it's kind of harmful and I didn't do it, about cigarettes,

never in my life, that alcohol is just something to try, understand that that's it, that's all. Well. We just didn't talk about this topic, that's all. It's like I didn't study, I kind of understood that they wouldn't be very happy if I smoked and so on, I kind of thought, why do I need this? Well, plus, I'm very frugal, and I understand that cigarettes are a huge waste of money, and so is alcohol, so why bother with it? Oh, I'll give you an example, here's one pack of incomprehensible cigarettes, then let's say you can buy a certain amount of sweets with this money. I'm like, candy, it's cool. That is, it's like, well, everything is measured by equivalents, even this, as I was taught financial literacy, that - mom, mom, I want Kinder, I want Kinder. Mom tells me that look, it costs so many rubles, but you can go to the store and buy so many candies by weight. I'm like, wow, let's have better candy. And I sort of understood that.
M1. Oh, I'll start now from the earliest, it's just that my mother has such a perception of life that she was against kindergarten, due to negative experiences in childhood, that she remembers by her example that teachers are very like that, not so much, and may not monitor the health and so on of the child. And she always sent me to some private development centers, and if you take me from a very early age, there are speech therapists with me, reading, something else, that is, in addition to the fact that my mother worked with me, that she is like me in fact, she taught me everything, before that at school, I went to such educational programs, and when I became more conscious, as an adult, probably at 4-5 years old or something, well, at 4-5 probably, yes, at 5 somewhere, I went to the children's center, combined with a fitness center for adults, and there were all sorts of sections like judo, fencing, and all sorts of other things, that is, I kind of went, it just sounds cool, interesting, some kind of dancing, gymnastics, acrobatics, absolutely went different. Accordingly, later, when I became even older, my range of interests was outlined, these are creativity and sports. Here. As a sport I went to judo most of my life, then 2 years to boxing, 2 years to karate. And I probably practiced judo for 10 years, or 12, I don't remember. I graduated from music school with honors, so in terms of interests, you can say, well, little by little, let's say take a swimming pool, creativity is a guitar, some kind of drawing, singing in different sections, that is, I actually did drawing in many different sections. I won't say that it was some kind of cool level, but just purely for myself, to communicate, to spend time, it was. M. And at school, it turns out that you also managed to study very well, in addition to these sections? M1. Yes, I can say that I, this is not my assessment, this is the assessment of others, that I am smart, and I had an interesting situation, that I understood that I could easily be an excellent student, I am not a fool, I can prepare, I can do everything, but I was just kind of lazy to spend so much time, and this is probably not very good, but I never had problems at school, any difficult subjects, the most difficult subject for me is chemistry. Somehow, out of interest, I just took it and decided, I'd better learn the topic, and then I didn't study it anymore, this topic, because it wasn't interesting to me, that is, I understood that I was doing things that were more useful for myself . Accordingly, I had no problems at school; if you look at the boys, I was among the smartest, that is, in terms of grades, and everything was fine. And this despite the fact that my gymnasium is one of the best schools in my city, where studying is quite difficult.
That is, my schedule is like this: at school I communicate with them, after school I go to the music room, there I communicate with these people, and from the music room, let's say I go to sports, there I communicate with these people. And this is how it went cyclically for me. That is, I communicated with many, but I won't say that all of them were my very good friends, they just communicated well, well, in some respects they were friends, naturally, in each section I had one or two people with whom I was most communicated, I think these are adequate things. Well, something like this.
M. But about the karate trainer, tell us how he helped you develop independence, since you speak about him so warmly and in detail. M1. Yes, I speak very warmly about him, about the coach,

because, well, this overlaps with the fact that I spent a lot of time in training, a lot of time, from the tenth grade I did karate, it got so bad that I had, let's say Monday, Wednesday, Friday, two workouts a day, 2 hours each, and the remaining time, let's say one. Each training session lasts somewhere for 2-3 hours and I was with him all the time, like listening to how much he loved to share some stories, how he not only enlightened us, but simply talked about all sorts of life topics, I heard him talk about it, I drew some conclusions for myself, what I can do, how I can do, similar things. Here. In general, just in my opinion, even in terms of education with a child, the main thing is to talk to him stupidly. Just talk. Tell some moments, life situations, how he got out of these situations, so that the child has some kind of template example that he could use in the event of some new situation. And then impose your own, having some kind of behavior model. That is, it seems to me that it is from conversations that a certain model of behavior is formed so that the child can use it and accept it as a given, in order to develop it later. He can completely redo this pattern of behavior, in case of some extreme situation he won't have to think, but what should I do, he will remember - yeah, my dad did this, or my mom did this, you can do it like this. He will do this, and then he will think, what is best for me? He will do what is best for him. That is, just in terms of conversations, all this is being formed. That is, the coach said that in reality martial arts are very connected with personal perception, and you cannot teach martial arts if you just come, roughly speaking, once a week to training and just somehow teach the technique. He said that all the masters, they lived with the teacher and seemed to watch him, how he behaved in the natural environment. They seemed to adopt the image, likeness, and mind of the teacher, how he behaves in a normal environment, outside of training, outside of sports. And this is the difference between a coach and a teacher. I can call my coach a teacher. For example, I can't call a judo coach a teacher, because his task was to make athletes out of us, well, he made athletes out of us, he is a professional in this matter, but there were no such life conversations. Because that was not his task. My karate coach had a different task. He talked to us a lot. Accordingly, well, somehow he is still just a teacher by training, I'll tell you what, at the university, so he knows what to talk about and how to teach. So it was very interesting and educational. And it seems to me that it was karate that revealed a certain personality in me, in terms of absence, removed some edges, that I can live calmly, socially, not be afraid to meet someone, do something else, because I don't know, how it happened, but I'm sure it was after karate, because martial arts associated with a certain kind of combat, they greatly change the worldview, perception, and seem to remove some kind of framework, boundaries. You become more confident. And quite independent. You could say. Because you are not afraid to defend your point of view in some dispute, knowing, like, that if someone comes at you, you will be able to give him an answer and give him a very good answer. M1. About the karate teacher and dash teacher, I was wondering if he has an education, you said that he teaches at a university, but what does he teach? M1. He is the dean of the physical education department of our Ryazan State University. M. How did you even get into his section? M1. This is also a very interesting story, I... (..) I just somehow saw how training was going on, in karate, in a completely different place, in a completely separate place, in the Krasnodar region, when I was with my grandparents in away, I saw how the training was going on. I was so impressed by this, because at that moment I was doing boxing, and I lacked some kind of morality, some kind of cultural core, when before training everyone bowed, sat down, closed their eyes, well, not just prayed, but they just sat down as if getting ready for training. After training, a bow, ritual phrases, when there is movement and intelligence, not just a straight punch, a side kick, but some beautiful Japanese names, Japanese language, culture, kimono, that's it, equipment, I really liked it, it went in, and I I started looking for this section in my city, I found a section in my city. It was another coach there, who reported, in fact, to the head coach, whom I spoke of as a teacher, I

went to him first, but then this coach said, that there was an opportunity to go to some training camp, go to another building, with this head coach, so I went and somehow it happened that the coach liked me and he decided that I would work with him in the training camp team, national team . And I began to study with him. And I spent, in about six months, actually, I actually studied for six months, I went along the path to a black belt, in fact, because I worked a lot, plowed, studied, learned new things. This is my story of meeting this karate trainer. Now we don't communicate, but I remember him very often, that is, in some conversations, and I am very glad that such a person appeared on my life's path.
Because money affects everything, without money you will not have the opportunity to live in a place where you have good health, eat well, pursue interests, that is, any hobby comes down to the issue of money. That is, I like to do martial arts, but with what money should I buy a sword, tritely, with what money should I buy equipment? That is, money decides everything in my opinion.
Well, here's an example. That is, well, really, communicate with people. Well, I'm proud that I was able to defend this Olympics, the championship, I'm proud that I have some skills, I'm proud that I found an internship, I found it myself, yes, I'm proud of that. I'm proud that I was able to achieve certain things in sports, just when I wanted to do karate myself. I'm proud of this too. That is, here.
M. Listen, but about dependence on someone, you say that you don't like to be dependent on someone, but why, that is, what do you put into this dependence? M1. Well, I had an offer from my coach to become a personal student, in Japanese traditions, as it were, that is, roughly speaking, to live with him, that is, to do what he does... As if he essentially becomes a certain , not exactly a master, but kind of dictates to you what you need to do in order to develop. Yes, I would become an athlete, but again I understand that I might not become if I had some kind of injury. That is, I did not want to put everything on this card. I didn't like this concept, that you are completely dependent on a person, that... For some, this is good, you don't have to think about what to do tomorrow, but for me it's not good because I kind of control myself and do what what will I need. That is, as much as I don't like being limited by someone. Any limitation here is not very good. That is, there you go. I like the Higher School of Economics because, by the way, there are no restrictions on studying, that is, you don't even have to attend lectures, that is, you are, as it were, responsible for who you will be. That is, there is no such strict control, so what - oh, you need to learn. If you don't study, you'll fly out. (laughter) As if only this fact stops you and that's all. There are no mentors there to control them, no micro-management...
Well, this is what happens in life, in general, it always happens, for example, if there was a need to get answers to some questions and close your own questions, whether for business or personal reasons, I found a good psychologist, went to a psychologist, solved the questions, closed questions. Somehow like this. Should I answer in this format? Is that normal? Is it clear now?
A. For health reasons. Well, in fact, in terms of health, what can you say, what can you say in terms of your health... Well, I've had problems with health for quite a long time, it was decided that health needs to be dealt with systematically, and the decision was precisely on the advice of on the advice of parents. I adhere to the following approach - once a year you need to do a complete Check-Up of your health, and regularly take tests and monitor them. And this decision was made very simply, because there was advice from my parents, accordingly, after the advice was received, I understood why this was needed, why you need to regularly track the indicators of your body, I realized how important the energy that you have is, to exist well, and there would be enough of it for what you do, what you do. Well, that's why I regularly take care of my health, track and live happily and energetically.
M. I think that you not only check, but you said about surfing and probably, well, you look very athletic from here, at least it seems, I think that in addition to checking, you also do things, well, you said, go to the gym, right? To the hall. A. Well, yes, because I realized quite early that sport is

energy, well, more precisely, at least for me, that’s it. And in general, I just can’t live without it, because... Well, no matter what your condition is, if you go to a training session, or go surfing, or go jogging, then you get a huge boost of energy, and in general I feed on this. Well, that is, sport is one of the key ways to get energy, to recharge in order to do those energy-consuming things that we do either in business or in other aspects of life. Here.
M. What brings such warmth to memories of school? In primary school? M. There was somehow more friendly communication there, with the team, but the only thing was that I was very far behind in sports. When they played football, volleyball and so on, I usually sat on the bench. M. Did you want to continue, did you want to play together? Or were there some reasons why you didn't play? M. To be honest, it’s difficult to answer, because on the one hand, it seems like you want it, but you don’t seem to want it, this. M. Was it your choice to sit and not play? M. (..) No, I think my lack of skills played (..) in this.
M. Yes, then you can move on to the next point. M. Choice of lifestyle and leisure. Well, this is where it gets more interesting, by the way, because I decided to change my lifestyle. He was like this, he is like this now, more sedentary, not very active, I found myself in such a rather interesting situation that after my previous job and previous company, all my interests simply died, because all my time was taken up by this sweatshop work and gatherings there with drinking there and so on. The rest just somehow fell away. And now I think what is interesting to me, what is not interesting to me, I restored my physical activity to a minimum there, in the future I will... In the future I will return to sports. I’ve even already decided what exactly it will be, what it will be.
M. And what will it be? M. I want to go to boxing. It's very necessary, in fact. M. Why boxing? M. Well, because it is he who has a great psychological influence on people. Well, according to my observations. This is both confidence and the ability to stand up for yourself, which is very important to me.
M. Completely, I don’t remember the last time I drank. In fact, with addictions everything is very interesting, I can, as if I don’t want to and I don’t. I also tried smoking then, but I didn’t understand this joke at all, to be honest, I just started and ended. Moreover, alcohol also makes me feel bad. Because alcohol is such a depressant or something. That I won't go back there at all. But I didn’t have time to develop alcoholism, I... I don’t have that.
This means health is seven out of ten, it’s more likely just a dislike for specific healthcare institutions. Otherwise, I can calmly go to the specialist I need.
Q. I changed, listen, I changed, and I changed a lot of times. I studied to become a psychologist-educator during my undergraduate degree. M. Oh, how similar. V. Yes. I studied to become a psychologist-teacher, entered the Faculty of Philosophy, well, the School of Philosophy, in general, the Higher School of Economics, so (NPT) himself is not very far away.
V. No, there was no such question, but my mother tried to compensate for this so that I would somehow interact with society, so I went to an English language club, to drawing, in general, they took me to all sorts of different events where there were other children, Mom often went out with her friends who had children, so it cannot be said that I was deprived of communication with children, just food, catering, my mother did all this at home, that’s it.
And I started, that’s how I played computer games for 16 hours, in the same way, for 16 hours, I read literature and classics of literature. And of course I tried to write poetry. Here. And then, in all the blogs that I kept on games, I began to write about the fact that games are, of course, cool, (npt) but it is necessary, well, the trick is that they greatly, they very much limit the space in which you're cool. And I really love being cool, still do. Here. And how could I, of course, be the top one in the game, but if I go for a walk around Moscow, it will give me little. Here. I wanted to develop something with more generally accepted values, in some more generally accepted values. Well,

that is, for example, knowledge of foreign languages, erudition, career prospects, and so on. So, at the age of 16, at 16, yes, at 16 and a half, in short, somewhere there, at the end of 11th grade, I realized this and began to work in this direction.
V. Yes. Yes, I wouldn't even call it stress. I kind of liked this state of competition again, another one, here. Well, so did the entrance exams, and I was like, oh, I entered Moscow State University without a basic philosophical education, that's fine, now I'm also a translator. In general, this energizes me. So, in short, I always loved this state of competition, and as a child, when you are not competing in the professional market, this is probably one of the only niches where you can compete, these are games, well, such games, board, computer, or sports games. Here, by the way, I would actually dwell here in a little more detail, because I love, since I love this whole business, I did both, and the third, that is, I always played board games with pleasure, a la cards and sports games. I say, I played chess as a child and table tennis. Here. But... But just at the age of about 12 years old, that is, at 11, I started playing computer games, and at the age of 12, I left sports and stayed only in computer games. Here. I wonder why this happened, well, I think I don't know. (laughter)
In general, in principle, I don't like, I don't think that anyone knows better than me, especially in pedagogy, in matters of education, because I have a psychologist's education, pedagogical, I know perfectly well that no one knows my educational does not diagnose needs. Moreover, no one has as much knowledge as I do about which method of presenting material is suitable for me, so this education system does not give me a choice, well, let's say this happens often, the curriculum says that there are many disciplines by choice, but in fact there is nothing there anywhere, plus the budget needs to be distributed correctly, in the end there is one, well, at most two, you have a choice. In general, I don't like this all the time, I like it when I build this educational route for myself. M. Well, that is, studying at the psychology department helped you understand yourself? Q. I don't think it was specifically my training at the psychology department, although... I think that it structured my thinking, of course, certainly my thinking and worldview. In principle, psychology, pedagogy, and philosophy, which I studied, help me understand myself better.
V. Yes, well, accordingly, I created my own group, and spent the whole last year working with this group. And now I want to try again, now I really don't know, but I still hope to somehow go to study in France, so here I have the same problem, that I'm not a psychologist, that I'm a psychologist-educator, and France is more strict system in terms of changing qualifications, so I need a year at a Russian university. Here. Therefore, I want to study at a Russian university for a year at the Russian State University for the Humanities, and then try to transfer to... Well, there is the city of Caen and there is training for translators in three languages - Russian, English, French, and so on.
V. Well, yes, with health, well, I'm generally not the healthiest right now, I have allergies, and a bunch of other diseases, but our medical system, it's usually in no hurry to help, and usually, until you get completely ill , somehow they're just trying to tell you, like, go drink oak bark there and everything will be fine with you. In general, my health taught me that no one except me will worry about my health. Therefore, you need to show maximum will to get a referral, to get the tests that are needed to get a referral to a research institute, and so on. And there are elections, that is, here, too, it's probably difficult to implement, that is, to withstand the doctor's pressure, because the doctor has authority, that is, like, are you going to teach me, puppy? You (npt) like me, did not undergo residency training. But, you need to show pressure, I then wrote the first complaint in my life to healthcare, to the Ministry of Health, here. This is just such a space of struggle for me, health, so I can say that this is... M
A. This means that what happened, well, I had certain health problems, (..) they are related to reproductive function. She was questionable to me. Accordingly, I needed to get a referral for

surgery, this is why the health sector is such an important thing. So, I needed to get a referral for surgery and it's very difficult to do, it was, well, this whole story began in 2019, that is, the diagnosis came to light. So, I tried for 3 years to get a referral, and at one time I received it, but then I got sick, it expired, and they didn't want to give me a new one, so. Who were the participants in this situation? Well, the main participant in this situation was me, naturally, and doctors were involved in this situation, but they were more like passive observers than participants. I would say my mother participated more, because she asked some doctors she knew there and so on. My girl too. Where did this happen? So, okay, let me tell you a little further, in general, I tried to get a referral, I didn't succeed, then, in one private clinic... Well, in general it turns out that this operation is very rarely done without any additional shady payments, that's it. And I, I have a principled position that I don't give bribes for anything, well, in general, I don't want to violate this life principle. Here. Well, in the end I decided to do this operation for a fee and it was unsuccessful. And if... Which I perceive, in fact, as a wrong choice, that I probably should have taken a more careful approach to choosing a doctor.
Q. A year ago. A year ago I had this operation, here. Then I felt pretty bad for six months, well, it was very bad for probably three months, so then I made a decision for myself that, well, sort of... Well, first of all, I don't blame myself for this, for the fact that I there may not be enough... First of all, there is no guarantee that if I tried another doctor it would be better. M. Well, yes, there's no way to check it. V. Yes, there is no way to check. Here. And I was guided by the recommendation that was given to me, that is, in principle, I'm not just like - oops, I'll go to any doctor. I've really come a long way, but the fact that it ended in failure does not mean that the path was wrong. M. Well, yes, it's not your responsibility. You did, you approached this situation responsibly, as far as I hear. That is, they did everything. V. Well, yes. So, in this regard, I calmed down. At first I kind of thought, damn, maybe it could have been better, then I thought - no, better, well, much better, as if I did everything I could, for my part. This is the first moment. And then, then I decided that I needed to get a good examination now, that I could... And then I kind of asked myself - what can I do now, that's what I need to get examined again now, I started knocking out all sorts of directions, there same level of hormones, what can be done about it? Here. And I began to move in this direction, received a referral to doctors, so. And then I started dating the girl I'm dating now. When I was just going through the referrals, and before I started dating her, that is, well, it was as if we had already started to have some kind of romantic relationship, so. And I tell her that well, right now I'm not at all sure about any sexual things that I can give you, so I say that let me first go to the doctors, they will prescribe me some kind of adequate treatment, and.. It's just that if I'm in a relationship, then I will feel uncomfortable, that maybe I'm not giving something that I should give in terms of my ideas about relationships. Here. And in general, somehow in the end I... By the way, this motivated me to speed up in that direction, to move, specifically to go to doctors faster, and to get directions with greater intensity, that's it. Well, somehow we continued to communicate with her, with this girl, and in the end all my functions returned to normal on their own. Here. Yes, but this happens, there are paired organs, the functions of one are taken over by the other. Here.
M. Tell me, do you remember the transition from your state of complete hopelessness to the fact that I can do something and in general, well, I can be responsible for my life and can take some action? So what was this connected with? Any external circumstances or more of your internal transformation? V. Tax. (...) Now, I'm trying to remember. So so so. (...) Well, at first, yes, I would probably describe it this way: at first I was just in shock, I didn't understand what was happening at all. Here. Then I began to feel the consequences, that I began to gain weight, and then I began to worry. Well, that everything is not going according to the best scenario that could have been hoped for. Here. Then, it's not like there was some kind of sudden event, it's just me, this question

bothered me, it’s like, I like to talk to myself, so. According to the method, like these two chairs, like what are you experiencing. How would I do something like this? So, this guy talked, so, okay, what do we want? We want to worry, or we want something, not to worry. Here. Let's think about what we can do. So, well, we, I mean, I just address myself like that, (laughter) here. M. Well, maybe some parts of you. V. Yes, yes, we are... M. They decided to act together. V. Yes, yes, yes. Well, yes, we are me, torn apart by the contradictions that exist within me. Here. As a result, I talked to myself, came to the conclusion that I... Yes, another thing... Yes, I remembered, I was very worried, really very much, and it always scared me... And I was even worried not so much because of what happened, but because I didn’t understand what to do next. Here.

M. So the uncertainty was scary? V. Yes, I didn’t understand, that is, which doctors to contact, where to go, that’s it. Both then and there there was still such a problem that everything is very slow in our healthcare system, that is, there, for example, they give a referral, but the appointment is only a month and a half later. I’m like damn, what am I supposed to do for a month and a half? I, I’m very worried about this, but I can’t do anything other than this recording. M. Yes, it's terrible. V. Well, that’s when I started writing complaints, this greatly speeds up our healthcare system, by the way (laughter). Here. Moreover, even those to whom I did not write, you just tell them that you have already written one complaint, immediately become more and more talkative. Here.

Well, a good choice, a good choice, I would probably attribute to my first university, that is, psychological and pedagogical education. And again, this is one of those choices that I made, guided by one criteria, and with the hope of one result, but it turned out to be good, that is, the results turned out to be different, but better. Than I could have imagined. That is, I chose a psychological-pedagogical course, because I knew biology, chemistry, mathematics, Russian, with these subjects I could enroll either in some kind of biological-chemical thing, like there you could go to Timiryazevka to study genetics, or to honey didn't pass. Well, in short, there was an option, either to go for something like that, biological, I was basically interested in genetics, at school I loved problems on genetics, but on the other hand, the option was to go to a psychologist, a psychologist-teacher, that’s it. And, in fact, I thought, damn, now I’ll go see this geneticist. Although I like solving these problems, is it that I will spend my whole life developing new plant varieties, animal breeds and stamps, strains of microorganisms? This is like the definition of genetics, actually. (npt) I think, God forbid, this is boring. I want to communicate with people. And I went to become a psychologist-educator. And why is this still a good choice, because I really liked my university, the Moscow State Pedagogical University, it’s not very overloading, I can, as a person who has already studied at a bunch of universities, and who has many friends from different universities, I know that it is so, moderately stressful. And there was a little more tension in the first year, and then just not at all, not very much. And I really liked it, because I think that if I had studied at another university, I would not have been able to simultaneously learn French, for example, to a high level, because I would have to complete some endless tasks there. And I would not have been able to gain work experience either at Artek or at Detsky Mir, or as a chess teacher, I was still working. In general, I would not have gained much work experience. I would describe the choice of university as successful, but now let’s talk about your criteria. So, when this happened - in 2015, who was among the participants in this situation? Well, I can say that naturally I was the main actor, but here my parents were naturally involved, like the parents of many children after the 11th grade, so. They gave me some advice there. I probably made this choice at the dacha. I was just wondering where, well, I was looking. I sat with the lists and in the end decided that, well, I went to different ones, and decided that psychological and pedagogical education, it still sounds universal, like a psychologist and a teacher. Of course, this is actually a trap, if anything, it’s a trap. In fact, you won’t be able to work as both a teacher and a psychologist (laughter) because you

don’t have a subject to teach. (laughter) And the psychologist is also only a school psychologist. Here. (laughter) But I didn’t know that then.
M. Do you have any hobbies or interests? K. Yes, there is, this is football. (?) I used to play and watch, but now I just watch. There for health reasons. Problems have arisen, which is why there are various injuries that are now preventing me from playing football. Here. From sports I would probably say table tennis, in addition to this, reading, various things, including scientific literature, geography, waste sorting and the environmental agenda. Then I’ll probably say, (..) well, let’s put it this way, yes, the fight against domestic violence.
M. Would you be comfortable telling us in more detail what you are doing in the fight against domestic violence? If you feel uncomfortable, you don't have to talk. K. I can, but now, for the most part, this is support for foundations and coverage of their activities there, that is, reposts somewhere, somewhere to talk about various actions, about what the foundations do in principle, for example about “Violence.No”, I visited them several times, well, at the foundation itself, there, and I regularly post something, that is, about what they directly do, as possible, and who you can contact there in case of cases of domestic violence, as possible with to help with this, where to go for help, what other resources are there to help such people, and well, in principle, helping yourself in such situations. M. And again, yes, such an interesting, sensitive topic, if I ask some question and he is uncomfortable, please feel free to refuse to answer. OK? K. Yes, yes. Yes. M. Yes, I would also like to ask, what made you support these funds? Maybe there was something that made you make this choice? K. Well, there wasn’t any specific trigger, I’m a lawyer by profession, and I had a legal education at a university, and before that, when I was studying law on my own for the Olympiad, I more than once came across the problem of domestic violence, including , when there were amendments to the criminal code, when certain offenses were excluded and, let’s say, beatings were not punished. Here. And at that moment I misperceived this problem there, and there I did not really perceive it as a problem later. It seems to me that I have several friends who also covered this problem, this topic. Also on various social networks. And I myself decided to look deeper into this and realized that everything was much worse than I thought. And, (..) well, then somehow it started to grow like this.
Well, next is probably health. A year ago, well, a little over a year ago, (..) I had an operation there on my leg, to straighten my toes, that too, it was a necessary operation, and after which there was six months of recovery. Here. And... M. Necessary, that is, it really interfered with your life? Has this problem been around for a long time, or has something happened? K. Well, about two years ago, even 3 years ago already, it appeared, at first I didn’t do anything, it went away after some time, there after ointment or some other such exercises, but after that the condition began to worsen, and It got to the point where it was painful to walk. Here. And so a decision was made there, here. And this summer I also thought about having the same operation on the other leg, but now all this is being postponed for me. Perhaps next year, perhaps even the next year. Because there are (..) various issues, including financial, time, because you definitely need to spend a week there under observation in the hospital, and after that you need to wear special shoes, and now it will soon be quite cold. It will be uncomfortable to walk in such shoes, so I am putting off solving this problem.
Here. Finance. Well, here the choice is probably only within the framework of finances, it’s about health and about resolving some issues in this regard. M. Is this about leg surgery? K. Yes, including, plus other various problems, there in the summer I decided to do dental treatment, so. Also when it started to become quite expensive, and then other ailments appeared that needed to be urgently addressed medically, so. Therefore, dental treatment there, too, has been postponed and is being postponed there indefinitely.

M. Yes, Dima, thank you for the detailed story. I have a few clarifying questions. Firstly, I notice, well, naturally, here there is a moment of professional sociological education, albeit lasting one year, that you use a lot of terms, and with them you seem to indicate some kind of processes that arise in your life, but according to Basically, I have the following question, sometimes you say, for example, that there’s this, this stupid procrastination, or... That is, you give some kind of assessments, and in connection with this it’s interesting, but you’re with someone... Are you discussing it now, or have you discussed those difficulties, including psychological ones, that you have encountered, and sometimes, well, naturally, they arise in life? D. I discuss them, but I discuss them mainly with friends. With my family, (.) I don’t have the habit of sharing anything in particular and asking for advice, for some reason I have a big prejudice in this regard. It just seems to me that older people, my mother is a provincial teacher there, she is unlikely to understand (.) the things that I can tell her about. And you don’t discuss it with friends, but only after the fact. Now here, with newfound acquaintances, I can discuss what I went through, but every time I go directly through a crisis, I usually don’t share it with anyone, well, also probably because I don’t think that anyone something can help me with this. Therefore, this is only after the fact.
About training, professional development - I decided to engage in self-development in the field of programming, I selected courses for myself that, in an amicable way, I would need to take. Because, in fact, I personally plan to work in the field of (NPT). Well, that's probably all. Health, I’m in... Six months ago, when my dark streak of procrastination ended, I started playing sports again, going to the gym. And during that period, I tried to arrange healthy meals for myself in the hostel. I stopped subsisting on some fast food and other semi-finished products and started cooking for myself. choose a diet.
I try to be kinder to myself and to others. Excessive self-flagellation and excess severity towards your friends, it rarely bears fruit, I try to refuse it. (...)
y, the choice of lifestyle and leisure, well, perhaps nothing has changed here, except that I began to drink less, because the first six months of my studies, when you constantly sit in the dormitory... Here, in fact, besides (..) drunken pastime, You don’t have any special choices anymore.
Okay, I'll try now. Training, professional development, probably six, making a choice may not be that hard, easy to say (npt) here to understand. But it is difficult to implement this choice. Health, probably three, but in fact, the gym and healthy eating take away almost everything from me...
B. Well, it’s quite hard for me to say about my peers, but I don’t think that everyone actually thinks about this in any specific way, well, that is, it’s still some kind of process that happens one way or another, in most cases, on its own with myself. Personally, I’m just getting this from, in principle, this is part of why I decided to take part in this interview, I’m interested in the topic of psychology, I have a good friend who is studying at Moscow State University of Psychology and Education, if you know, to become a (NPT) psychotherapist.
B. I personally find out about this from some sources, like Telegram channels, Instagram of some well-known psychotherapy services that run, some of them make short posts on some topics, that’s it. Well, sometimes I read some articles, but just considering that I don’t seem to know much, I approach it carefully. But in general, this is probably the most important source of information about this topic. Well, about other, some psychological topics too.
K. Yes, it’s probably a sport, now, probably for the last three years, due to health problems I haven’t been able to play, but before I played, studied, within the framework of the university, I managed to play and... Well, this is football, then probably tabletop tennis, also a sport, now
K. (.) Yes, these are some restrictions on the part of the parents, because of vision, for example, you couldn’t play on the computer for more than one hour a day, there are some restrictions on the time spent on the street, that is, when to take a walk there , restrictions...

decided to carry it myself. (..) Well, I went up to the guys, near the tents, to the boys, I said - boys, can you please help me carry the suitcase, I can’t do it myself. They told me - no, we can't do it now. I say, okay, okay. I'll carry it myself. Here. And somewhere in the middle of the journey, I already realized that I simply couldn’t cope, that this rib was starting to ache a lot, there was pain. And I wrote to the chat with the guys, I said - guys, the boys really need you now, please help. Here. And no one reacted at all. Here. And I just walked, roared, carried this suitcase, so I carried it. But most of all, I don’t know, what made me laugh was that I was walking, and so I met many young people, they were just walking like - damn, she’s so cool, really, strong, independent, carrying a suitcase herself, in general. I walk and think... M. And it was very painful for you. S. And I walk, roar and think - you didn’t think of offering help, somehow, I don’t know. I even, I don’t know, I always somehow offer help... (..) I, I don’t know, I’m used to doing this, so. Then I just went to the doctor, they told me a lot of things about how I shouldn’t carry heavy things, but I went and did all this, that’s it. So. Do you often think about it, about this situation? (.) Well, recently, often, because it happened recently. (laughter) Here. I told my parents, my mother said that everything can be expected from me, and what emotions arise? The emotions are so mixed. M. Can you explain? S. Well, in the sense that yes, I myself was able to convey all this, but it was to my own detriment, but also that, as it were, I don’t know, we were always told from childhood that boys should help girls and all that. And then they just go like this...
M. Where do you study and for whom? M1. I'm studying at the Higher School of Economics to become a psychologist.
M. That is, it’s like the ability to solve your problems, the ability to choose how you want to spend your leisure time. Tell me, in setting your goals, do you show independence, focus primarily on yourself, or does someone influence you? M1. Yes, of course, I primarily focus on myself, that is, for example, with the same boxing, as it were... (..) I really, like, all the girls are like that - boxing, why, why do you need boxing? I’m like this - I want to go boxing, that’s it. (laughter) I went, found it, went, liked it, and now I go, and that’s it, everything suits me. If it concerns some goals of others, then yes, I can set goals for myself, I can fulfill them on my own, but here it all comes down to financial dependence, sometimes I cannot achieve a goal due to the fact that I do not have the funds for it implementation.
M. Now I will ask you to give an example of a situation that you especially remember when you showed independence. I will send the main points to the chat, which I will ask you to indicate. Please look at them and tell us what kind of situation it was and then down the list. M1. Okay, now, I'll think about it. (...) Well, we can say that this is admission to HSE, the Higher School of Economics, because many questions arise when you go to study psychology at the Higher School of Economics. Here. Because I am studying commerce, but I also submitted my documents to RANEPA, the institute of the President of the Russian Federation, and there I got into the budget. And how it would have been, one might say, a year ago, during the period of admission, and I decided that I wanted to go to HSE, because there is really strong psychology there, there are cool subjects, teachers and in general I like all this, and at RANEPA I I don't really want to. And who was with me... Well, I decided it myself, I just brought the decision to the family table, so to speak, and that’s it. Well, parents. Well, that is, they didn’t press, they just said - choose where you want, and that’s it. And how did I understand that I was doing it on my own? Well, probably because many were unhappy with this decision, well because probably, for example, my grandmother did not understand why I was entering some kind of Higher School of Economics, although I could go to a university under the President of the Russian Federation. And so on. M. Didn’t that stop you? M1. No, that is, like grandmothers, they are generally like that, you know, well, they’ll say something, I’m not much different there, because after all, it’s a different generation, and somehow

I'm very loyal to them, so I just said - Yes, I'm such a fool. The situation says something about me... (.) Well. probably that I am following the goals that I wanted, that is, in the 11th grade in September, I opened the top psychology universities, saw HSE in first place, and that's it, I went there. And she achieved what she wanted. Do you often think about her... Yes, like boasting, of course, that you could go to the RANEPA budget, yes, I love it, and my grandmother often talks about it, remembers it because she would like it. But life turned out differently. Yes, I tell my parents, well, as if they know everything, sometimes I tell my friends, because in principle the situation is cool and interesting. They said... Well, I said about parents, relatives, but friends, they usually, well, somehow just laughed, like something like that and that's probably all. That is, there was no violent reaction there, because choosing a university, as it were, is a situation that every one of my peers has encountered, so they basically understand and there is no condemnation here and so on, despite the fact that I like it Studying at HSE is the opposite - well done for choosing a university that you still like. Emotions... Well, probably pride, joy and some kind of nostalgia for those times, (..) probably these are the main emotions.
M1. Now I'll think about it. (...) So, well, one successful one. (..) I was in the eleventh, no, tenth grade, I wanted a dog, and at that moment I already understood that I definitely (xx) would not stay in my hometown, in Rostov, that I would definitely move. It doesn't matter which college I go to, but I'll definitely move. And... But I really wanted a dog. And as if we were looking for a dog, we found it, we had already paid a deposit, well, like, in a few days I have to go pick it up, and I understand that how can this be, then I will leave the dog with my parents, because I will live in a hostel , well, for sure, I had a choice either not to get a dog, and let's say, not to fulfill my dream, because I never had my own dog, we have a lot of dogs in our family, but we didn't have our own. And no one knows when I would be able to get myself a dog. Or cancel everything, let's put it this way, and simply not burden the parents. And that was the choice. Either you blindly follow your dream, then you burden other people because they need to take care of your animal, or you care about other people more than your desires. This happened in the tenth grade, I was probably 17 years old at the time, and me, my mom and my dad were involved in this situation. Because my sister doesn't live with us, therefore she won't take care of her. Where this happened, this happened in my hometown, this situation made me feel like I needed to decide something, that I needed to do something and that I was afraid of making a mistake. I'm afraid of ruining the dog's life there, I don't know, causing some kind of trauma to myself, or burdening my parents with this too. That is, the situation was not pleasant. The choice was not a pleasant one. But now, (..) I treat her calmly, I can't say that she directly somehow excites something in me, probably, I just remember with some pleasantness all this then... All These are my decisions that led to the fact that I now have a dog. (..) It evoked different thoughts. (...) Don't even know. I think, probably, I was just very afraid to hang some kind of responsibility on another person, I was very afraid that the animal would forget its owner... And it's not very pleasant for me either. (NPT) kind of like for myself, but like for others, too, so. Now this gives me (..) also mixed feelings. Well, that is, I don't know why I did this then and decided to get a dog after all, and I don't know what I would do with this situation now, well, that is, it's like you just decide in the moment, yes or no, and probably Then I just let go of all the consequences and lived in this moment, that yes, now I want a dog, now let's live in this gorgeous moment, and then I will accept the consequences and decide what to do with them. Here. But I think that it turned out well, because I love her very much, I have a gorgeous friend who will always hug you, lick something, and so on, make you laugh, play with you, that is, how These would be very pleasant moments, memories, so I think that I made a good choice.
probably my sister also has health problems, so she, a little like this, somehow maximally, (sigh) I don't know, reaches maximum heights during education, and therefore, probably, this is also a

little bit of my responsibility, (laughter) also everyone, and they also pay a lot of attention to her education, even more than mine, because it was more difficult for her to study, but certain hopes were probably pinned on me in this regard, but I'm not exactly (npt) on this topic of approval, that you should do something there, always do everything well, I don't know. (laughter) Well, yes, in general, this was also important to my grandparents, but what my grandparents on my father's side, basically, my grandmother was more likely to just say, go ahead, well, it was important to her that I do in basically anything, and I moved somewhere forward, and managed to do everything. But it didn't matter to her in what area I was doing it. Here. Probably so. M. But it turns out that both pairs of grandparents have higher education? I. Yes, everyone has a higher education, yes. M. I'll carefully ask about my sister: what health problems did she have, were and still have, what is the reason for this? I. She has autism spectrum disorder. But she is quite highly functional, that is, there is no such thing that she, I don't know, it's probably not very noticeable, in terms of... (..) For people who are unfamiliar with this, they probably won't immediately understand that something is wrong specifically. That is, they may think that she communicates in some strange way, or something, I don't know. Well, in general, not so much that she couldn't study, for example, but in life it interferes with her. M. But you said that due to the fact that your sister has such difficulties, sometimes they even paid less attention to you, and so you, well, how much did this bother you? That is, did it evoke any emotions? I. In general, no, probably (laughter) only because they always paid a lot of attention to me, and sometimes, I would say that it was even some kind of overprotection, so for me, I don't know, it's just very difficult to assess the effect and the cause , that, for example, they looked after me a lot, because my sister has difficulties, for example, for a very long time I was not allowed to go anywhere on my own, (..) well, on the subway, or on some public transport, and they were always driven, and it annoyed me because I wanted to travel on my own. And I don't know what this is connected with, maybe because my sister couldn't drive for a long time, and I started driving earlier, my parents were afraid to let me go. It seems to me that some of my parents' fears, as it were, related to my sister's health, are transferred to me. Well, at least it was like that before. Probably not anymore. But maybe, maybe I'm just thinking, in general, I've never had the feeling that they pay more attention to my sister, because I also received a lot of attention. And vice versa, probably at some moments it was good that this attention did not go to me and I, I had more freedom.	
M. Did you feel older precisely because she had just such health difficulties? I. Yes, because for quite a long time, but even now, she still doesn't have it so strongly, for a very long time she behaved as if she were much younger. Therefore, at some point, well, I remember, right when I realized that she had some peculiarities, I asked my mother, by the way, I even remember this moment, I was driving, we were going either to a dance or with dances, and my mother began to tell me, I was about eight or nine, and she gave me a book to read, well, it's such a cool book, "Autism through the eyes of a sister," it's like from the point of view of a girl whose brother has autism and she tells . I think yes, it's quite similar, because some patterns of behavior are described there very, very similarly. Here. And when I realized this, I somehow began to relate to her easier, because when you know why she behaves so strangely and it annoys you, and it's not clear what to do about it, when you know it, it's easier. But still, I noticed that my sister acted as if she were younger, as if she were younger than me. Therefore, I didn't have the feeling that she was older, and that somehow... In general, I was probably ahead in terms of development after all.	
Regarding health, there are probably no solutions. At least nothing comes to mind. There, every year you just go to the doctor, and he tells you what to do and that's it. (laughter) M. So you are generally calm about your health and therefore somehow don't even remember? I. Well, I probably haven't had anything like serious health problems lately, if something happens to me, I go to the	

doctor, but I don’t know there, I recently had poisoning, I go to the doctor, he me right away (npt) (laughter) but there were no such, well, particularly important ones, but probably if I had had any health problems lately, I would have remembered it better, but so, (laughter) nothing there was no specific one.
M. But it seems as if you have done a lot of work compared to the way you talk about your adolescence and the way you behave now. You are very open, you are very emotional, and talking about such experiences is a great courage. In fact. N. (laughter) I have chosen a psychotherapist several times and now I finally go to a normal one, and everything, plus or minus, yes, is being worked out, so it’s just... Well, that’s exactly what I’m talking about, about the fact that from the age of eighteen, I can finally make choices myself, and I can, as it were, in an emotional way, that is, choose what I feel, what I don’t feel, and what, I can behave this way and be like this, that is, in some moral things, and in some actions. That is, I decide where I will do the actions one day, and I chose this, and I will, well, kind of lead you like this. And be like that. This is also like a choice, in my head, it’s considered, because, well, just then, all these things, they were very strong, I cried there for months, and in general, I felt even more downtrodden, but fortunately, some things started there. then a fairly adequate relationship that lasted quite a long time, and this somehow made me feel that I was not at all... There is someone who loves me, someone who appreciates me, the same one like this... Replacing unconditional love with parental love, that's it. And probably, in parallel with all this, probably in parallel with this, there were also elections in the 11th grade... M. Can I have a second? Tell me, do you attribute your transformation to a greater extent to psychotherapy, or perhaps there was also some event that made you look at yourself differently and begin to behave differently. N. More likely, to a lesser extent with psychotherapy, but that is, I started adequate psychotherapy literally four months ago, because before that I just went to psychotherapists, and somehow, well, I couldn’t find someone with whom I would be comfortable, Here. So I associate it rather with the fact that the most important thing is that now I can, since I was eighteen, I do not depend on my parents, that is, if before that my parents could forbid me something and somehow control me, now, well, this is absolutely not the case, and we have some kind of, well, adequate distance, Well, you know, there is an expression, healthy indifference, something like this, probably with us. It’s clear that I love them and this is unconditional and absolute, but how could I...
M. And after 18 years, what important elections did you have? Besides work. Maybe in some other areas? Independent travel, I heard this spring, maybe something else? Choosing a psychotherapist? N. Yes, choosing a psychotherapist. That's for sure. (laughter) An important choice, I’m very glad that I found my current therapist, she’s very strong there. Yes, independent travel, because at first I started traveling around Russia, it was also funny, of course, because I turned 18 and Covid began. Like hello, thank you. Nastya, who had never been abroad before, or even in any other cities, here. Yes. So I myself began to travel slowly around Russia, sometimes in a company, sometimes on my own, and then I decided - damn it, I’ve never been abroad. Here. And yes, in general, I’m very glad that I went to Istanbul, so. Because I went there myself too, that’s it. That is, completely alone, not knowing much (laughter) English, that’s it. But it was great and I’m very, very glad that I went after all, because well, that’s exactly my trip, I bought tickets there a few weeks before February 24, and the tickets that I repurchased were canceled 10 thousand times , and I was already thinking, damn, how am I going to go in such an atmosphere, but I went anyway. And I’m very glad, because... Well, at least this ticked some box, because you still felt as if you were in some kind of geographical cage and (npt) get me out. And this summer I also went to Dagestan, on my own. And this was also wonderful, it seemed to slightly support your own feeling that you are doing something with your life, and you are sort of solving not only some work or romantic processes, so. But you also allow yourself to receive some kind of pleasure. Here. Yes, what other elections,

well, probably a partner, we still have to talk about it, because this is my first breakup with that young man with whom we dated for two and a half years, this is probably an important decision that I accepted. And after that we dated for a year, because I, in fact, I left him for another partner, with whom we were in a relationship for a year, it was such a very cinematic relationship, with... I think you know the expression Dead Inside, here, (laughter) this is it. (laughter) So, in the end, we parted ways, well, that is, I can generally say that he abandoned me there... Because of his own pens, because he was somehow against me projected, as they say, and this was also a rather difficult period, just around March - April, all this was also layered on February 24, and (..) there I did not make any choices, (laughter) this is exactly there was a moment when I directly felt that I had lost some control, and because of this, well, it was really very difficult. Well, it’s also related to this, that is, I found a psychotherapist right on the same day. Here. And thanks to the fact that I decided to find her at all, and because I found her, I somehow, well, generally survived all these moments, because of course the first separation was very difficult, well, actually without your will, which was done. Here. Well, then there, after some time, it seems that I met a young man with whom we now have, well, some kind of relationship begins, this can also be considered some kind of, well, choice in the romantic part is important, because Well, there is 50 percent of work that calms you down, and there is also, well, it’s clear that it’s not 50, there are other breakdowns, probably 30 percent of work, 30 percent of relationships, 30 percent of friends, and then there’s 10 percent, this is some kind of totality , oh well, the family still needs to give some part of the interest, so. Well, that is, I just remember that just when I was in Dagestan, I felt very happy, because finally everything in my life, plus or minus, seemed to work out, and this, well, if you look at all these segments, Well, they're pretty good. Here. That's why...

M. No, on the contrary, one is easy, but ten is difficult. So then you have three in training, right? N. Yes, yes, yes then for training three, and (..) health - (..) one. That is... M. Easy? N. Yes, well, you go to the doctor, make an appointment there (laughter) if something worries you.

N. Well, then, I think that I can go back to the choice that I made when I was 16, when I was in the summer, when I decided to go to the young man with whom, who hinted to me for sex, for some kind of romantic relationship, that’s it. And to his friend's house. Well, accordingly, I was 16, that is, what year was it, 2018, yes, it seems. Yes, summer 2018. Here. (..) Accordingly, I was there, this young man was there, and his friend was there. And so I ran away from home to spend time with them, because it seemed to me that it would be cool and that I would probably get closer to him, and indeed, he would kind of like me as a person, and that’s how he would like me probably appreciate it more. Well, in principle, I liked the feeling that he was paying some attention to me, that a person seemed to appear who appreciated me, at least for something. Here. Maybe sexual desire, and it seemed to me that, well, in general, I could probably somehow, well, show interest, and he would appreciate me even more, but for something else. Here. And all this took place in the Moscow region, at his dacha, in his house. And... Well, it happened, (laughter) how to describe it, I just don’t want to go into any super details, but (..) in the end, after we first talked with his friend, who told me what - there with these vodkas, so, and then this friend himself came, oh, not a friend, but the young man himself, with whom we had some kind of fuss, so, and he took me upstairs, and then I continued I remember it very well, (..) just in very small snatches, here. But it was all connected with some kind of (sigh) (..) well, sexual actions. In the end, I don’t know how he would have entered me or not, but he tried, let’s say so. Here. And what kind of feelings this situation caused then, obviously - not the most pleasant ones, well, that is, I was probably in some kind of teenage age, so that everyone, I was just, I was just worried about everything. Well, it seems to me that everything was just finishing me off gradually, that is, it wasn’t so much the situation itself that was finishing me off, that is, the fact that... Although, well, in general, it’s difficult for me to

separate this for me, the fact that we then stopped communicating with him , that is, he seemed to write to me at the beginning, well, that is, maybe he wrote there twice, so. And that's all for me... (..) Well, sort of, the fact that he's no longer interested in me at all, and the fact that he absolutely doesn't need me, and the fact that no one loves me, doesn't appreciate me, I I felt absolute loneliness. I remember that I imagined that I, firstly, well, that is, I just had pictures in my head, like I was alone in the middle of a white sheet of paper, and in general there was no one close to me, as if my parents don't give a fuck, my friends and I are so close We don't communicate, that's it. And, well, that is, it just felt like such total loneliness, rather, this whole situation led to these feelings, so. And what feelings now, (sigh) does this situation evoke, now, for a second. (...) (sigh) Well, probably (..) I just thought it through, thought it over 10 million, billion times, turned it over in my head, and somehow the psychotherapist and I seemed to have worked through it, give or take. .. And now I just feel some kind of, I don't know, sadness, sympathy, probably for myself, but it's global, because, well, obviously none of this would have happened if I felt somehow there much better, that is, as confident and calm as she is now.
N. (sob) Thank you, it's very nice to hear all this. It's just that now it's actually much easier for me to tell all this, because it's all already agreed upon with my loved ones, and well, my friends, my former partner, or my current partner. And to the psychotherapist, here. That is, like another year or two after what happened, of course it would have been more difficult for me to tell all this, but now it's like... Well, you just understand that... Well, somehow in general, it's all rational, meaningful and... But thank you very much for the words of support, it's very (..) nice every time, me too...
M. I really like the way you praise yourself, it's really great that you acknowledge your victories, understand and build it into your story, you understand that yes, I really did it right, and now I get what I wanted, and even unsuccessful elections in the past become not so unsuccessful; on the contrary, I am very glad that I made a different decision then. N. Well, I think that this is all kind of connected, plus or minus, with the fact that I turned eighteen, I began, plus or minus, to somehow adequately perceive myself and the world, and then again, now it's like I've added to all this psychotherapy, and but I just, as if my emotional intelligence is growing a little, you can, well, somehow listen to what you feel and already determine what you feel, and not just feel and be like - oh, I feel bad, (laughter) here.
As for health, well, as such, well, firstly, I chose to do a health check-up in order to identify some... Some areas in which there are some problems, this was already a big step, because I couldn't get to a regular doctor. And so I chose not to put off some moments. Of course, now, with a new workload, it's been postponed for some time, but I think I'll get back to it soon. (..) And I will start again the procedure that I was doing. M. And what about regular health checks? How regularly will this happen? A. Regularly - this is six months. (..) And about work, work... M. Sorry, please, can I ask you more, about your decision about regular health checks? A. Yes, of course. M. How did you decide to do this? How did you make this decision? A. (..) Well, since we now had Covid, I... And I never had confirmed Covid, I decided to go and find out if everything was fine with my lungs and everything else, and since now there are pavilions in Moscow, health pavilions, and there is one of them literally next to my house, I thought that it would take an hour, an hour at most, and I have an hour of free time, I can go there and do it. And also, well, when... (..) When you underwent medical examination at work, mandatory, it's also like a choice that we are not given, (..) what you are obliged to do, and also this, well, some your own feelings that something is wrong and you should go to the doctor, and he will refer you where you need to, according to your needs.
M. Yes, of course. If you think that's it, move on. A. Relationships with parents. This is a very difficult question, because... Well, I have always had a very (..) (h) great attachment with my parents, because they literally did not let me go anywhere, and it was this year that I decided that I

would completely separate from them, I worked with a psychologist about this. And... (..) And I tried to distance myself from them as much as possible, because I understood that I was already independent, I was already old enough to make decisions on my own. And, (..) well, not even listen to any advice, realizing that my experience, conditionally, of living in Moscow, studying and working is completely different, like theirs, in another city, and since we are completely different generations , because, well, my parents are adults. Here. And in this regard, it turns out that relations with parents may have cooled somewhat, and they are not as active as they were before. (...) Are there any additional questions on this point?
M. Please tell us in more detail how it happened that you wanted to start volunteering? A. I accidentally saw an announcement about holding a marathon, in general, in my city, I had to run it, but there was some kind of large fee, because, well, I used to do athletics, and I thought that I could run, but there there was some kind of large contribution, and I thought that well, I don't really want to ask my parents for money, and I saw that volunteers were needed there, and I simply wrote to the post office. And after 2 months they answered me, here, come. And then again, again, again, again, and so on, now several projects a month. (laughter) Or maybe even a week, and in general, now no longer as a volunteer, and not only there on all-Russian, but also on international projects. Even now, no longer as a volunteer, but as a manager of volunteer projects. M. Great. Is this related to sports, are these different projects, or is it one direction? A. We did this with sports, but now sports are a big, well, sports projects, they are a big part of my life. And the rest of the projects are of different directions, just the very fact of volunteering, the very fact of helping, it remains unchanged. M. Can you tell me what feelings the situation caused you to decide to volunteer? A. Then it was very interesting to me, because this is new communication, this is a new community, these are new opportunities, because I really missed communication exactly in adolescence, then it caused a feeling of such, perhaps instant admiration, that is happiness is in the moment, now this also happens from time to time, precisely at some event, but you just realize that you have something to do, and you can go to any event and go help someone. M. How do you feel about this now? A. Now I'm completely delighted, because you live this, you love doing this, when you're at some event that you really like, you just almost cry with happiness that you're there.
And probably one of the things that helps me very, very much is that I have been involved in sports since childhood, at first I went to a circus studio, probably from the age of 4 to 9, I really liked it. And I liked the fact that there is freedom of creativity, and there seems to be some kind of framework, in which you have a coach, you have your leader, and you, for example, put on a number with him, that's it. We also traveled a lot and participated in some international events, and I probably even received my first salary, well, as I believe, I received it abroad. And it was interesting, because these (.) were moments that relieved the fears of the unknown, because for example, we performed in front of a huge audience, there on the streets and did some kind of performances, despite the fact that we were all small, we in fact, they were very shy, that's it. We somehow overcame ourselves, and it was somehow unobtrusive or something, because we perceived it all as a game. And now I understand that I can also use this approach in some life situations.
And I kept asking, asking my parents to bring me to the gymnastics group, and then I still don't know, maybe my persuasion somehow influenced my family, maybe something else, that's it, but I still They brought me in and I started playing sports. And I understand that it's probably sport, it has played a lot in my life and in my character, too, a lot from sport. I have there, regarding the achievement of any goals that I set for myself, that I can gather there, even if I understand that it's hard for me, bad, or something else, I understand what I have after all, there's this kind of core that probably doesn't allow you to give up when you just need to pull yourself together and do

something, come up with something, that’s it. Or somehow cope with the situation that exists. Here. And probably the fact that in my family, in general, (..) there is such a moment of complete freedom. That is, I chose, after school I entered a technical school, because I did not enter the university, and my parents said that you choose a profession and direction yourself, because we want you to be a good person. And of course, perhaps, I think that this was bad advice, because when you are 16 years old, and you want to get specific, well, some steps, how to choose, what to do, maybe how to look at the test results, according to career guidance, which shows you 16 areas of activity, and you don’t understand what to choose. Here. But it seems to me that this is my path in education, it seemed to be a little in different directions, but it seemed like it was almost cultural, almost literary, probably. Therefore, somehow everything comes together, as if like a puzzle, here you are, seemingly from different places, but you find puzzles that your picture is missing. It's probably something like this.
P. Health, oh, I had to choose several clinics, here. And I also read there, looked at it, asked some people who live there for some advice, what’s what, what’s best to choose, that’s it. And that’s probably all there was in terms of health. M. But where do you usually get advice regarding clinics there, and the health sector in general? P. Here, as advisers, I probably have experienced guys from the dormitory where I lived, because (..) they, well, I asked the guys from the second year, and I asked, because we had several clinics around the dormitory, which is better, is the university clinic better, and so on. Here. Plus, I looked at the reviews, and looked at more reviews on the main website of the clinics. Here. Well, I chose and I'm happy with the choice. Because everything somehow turned out very cool there. Well, just one piece of advice that I was hooked on, I read it, and I realized that this is a great clinic, and now I’m really happy
stopped playing sports at the age of 16, and probably now, after a certain number of years, I would tell myself that I still need to continue to exercise, even a little, even a little bit at a time, maybe a couple of times a week , but still do it. Because at that moment I had completely finished studying, that is, I had some activities, not much at all, but still, here you go. But I wouldn’t want to quit. This is probably the choice I regret. M. But if you go deeper, when exactly, how old were you when this happened? P. I was 16 years old, it turns out that I graduated from school, began studying at a technical school, and I simply did not study in the area in which I live, and not in the area, and not even close to the area in which I played sports. And plus it so happened that at that time the group in which I was studying was disbanded, and then I thought that, well, the coach and I also discussed that it would probably be better for me to look at some university or technical school somewhere. activity, because I trained with girls who are younger than me and from a sports point of view, this is kind of a step back. And significant, because if you train with those who are on the same level as you, or a level or two higher, that’s good. And when you train with those who are kind of far behind you, it’s not good. Because you, too, will roll back to approximately their level. Here. And then, yes, at that moment I thought that yes, it would probably be difficult to travel, because school would end anyway, it would be very late and there was still a road to class, and classes too, and it would be difficult. Here. Well, then I bought a membership, a membership to the fitness room, that’s it. But it was also difficult with him, because he was also not so close, but still. Here. And I went and worked out, but I understood that this was not enough for me, and I just wanted gymnastic activity at that moment. M. But you said that at that moment, well, as an adviser, well, the coach was present in this matter, and who else? Who else was involved in this situation? P. No, just me, me and the coach. So, it turns out, I told her (..) my situation, and so, well, she suggested this option. Of course, she wouldn’t have refused me if I had said that I would like to continue, so. But she outlined the situation to me, as she sees it, as a professional, as a coach. M. But if, in contrast to unsuccessful ones, we talk about successful elections, what elections do you think were successful in your life? P. I would probably highlight

the choice of sports, gymnastics, as especially successful. I understand that it's mine, and I understand that it was very important and great that I liked it. And I'm probably lucky in that this sport is interesting not only to me, but also to my family, and my family really supported me in this, then the choice... (..)
M. Tell me, how did the desire to go to study journalism come about? Why did you decide? L. Well, in general, until the seventh grade I wanted to be a psychologist, so. But... M. How interesting. L. Yes, my dad is just a psychologist, that's it. But in short, there was such a story that I began to find out how to enter the psychology department, and found out that I had to take biology there. M. Biology, yes. L. And biology was generally my favorite subject at school, and I say - no, I'll look for biology (npt) for something else. And so I started buying all sorts of magazines there, these ones, for girls, (npt) then later, I became interested in all kinds of fashion journalism, and then it means, I don't know, there in the eighth grade, in the ninth, I became more interested in politics, I wanted to be a journalist who travels to all sorts of hot spots, that is, something like that, little by little it became more and more boring, then I just wanted to be some kind of observer, that's it. Well, that is, it was just some kind of interest of mine, based on some of my predispositions. That is, I was always good at writing texts, in principle, my language was so good, well, I understood that this is what I am passionate about. Here. M. That is, but it was as if it was a choice from the opposite, that if not a psychologist, then I would be a journalist. L. Well, I wouldn't say that this is probably a choice from the contrary, that is, well, my interest in psychology never went away there, my dad always gave me some books to read, he conducted all sorts of tests with me, that's it. It's just, well, maybe I'm also partly burned out, because, well, I'm basically the kind of person who, if I really want something, I'll hurt myself, but I'll achieve it. Well, that means it wasn't such a really great goal, because if this goal had been great, well, I would have learned biology. Well, what can you do? (laughter) M. Why did you want to become a psychologist? Because dad was a psychologist or is it somehow different? L. No, I actually found out that my dad is a psychologist after I decided to become one. But this, you know, is such a story that you never know what your parents do, and then you find out. Here. Well, in my opinion, it all started because of the series "Lie to Me", I was very interested in this topic there, like body language here (npt) that's all, it was so cool, then I read that There are sports psychologists, I thought that was also interesting. I wanted to be a sports psychologist. And then I began to get interested in serial killers, just reading their biographies, and I wanted to be a psychologist who works with all sorts of criminals, that is, he understands why they commit such terrible things. Well, maybe I also watched enough of some TV series there, like "The Crypt", where I remember going to the dacha, watching with my grandmothers in the evenings, where a criminal is also sitting there, and a psychologist is working with him. Here. Well, somehow it attracted me because it was such a very socially significant profession, which, in general, was aimed at a good cause. M. At what age did you decide to become a psychologist? L. Oh, well, it seems to me when I was maybe 10-11 years old, that is, before that I wanted to be an actress, well, let's say, many girls in elementary school want to, and then I think - no, it's too fickle, a psychologist - that's the most That.
M. Yes, there is such a thing. Health? L. Health. Yours? (laughter) M. Yes. What were you even thinking about? L. Well, it often happens to me that I force my loved ones to take care of their health. At the same time, I don't pay that much attention to it. Because for example, there, I don't know, I think when I lived with my parents, someone's blood pressure would rise, and I almost called an ambulance, but they didn't do it. And in the end I understand that I did everything right. But as for mine, I'm like, damn, again I feel sorry for the money for the dentist. Well, let's probably say here then... (..) Probably a five, that is, somewhere in the middle, because, well, as far

as it concerns words, I understand everything perfectly well. But when it turns into business, it is much more difficult. For example, I couldn't get to one doctor for 3 years. But when I got down to it, I immediately realized that thank God. M. Okay. Job?
Well, not a profession, but probably just a hobby, I just understand that in fact all my energy is there, and when I step over myself, well, I forget to do this creativity at all because I have no time, but I begin to suffer from it. Sometimes I have some kind of blues there, I can even get sick, psychosomatics starts to turn on. Well, in general, these are the moments. M. Katya, tell me, have you ever had a consultation with a psychotherapist? K. Yes, by the way, it took place at the Higher School of Economics.
M. Where do you work? M1. I work in a private children's correctional center as a speech therapist.
M. Do you have any hobbies? Besides work. M1. Yes. I like to play sports, different ones, it could be yoga, dancing and I like to embroider.
M. Yes, but where, as it seems, maybe you or your peers can get information about what independence is and what an independent person is? M1. Well, probably more from... From psychology, but from some kind of popular psychology, because it is more accessible and understandable. And very popular now.
M. What is the best way to contact you now, how old are you, what city do you live in now? N. So, Nastya, you can, I'm 22 years old, I'm still studying. Well, that is, I graduated from HSE - Higher School of Economics, Faculty of Psychology, this year, bachelor's degree, and now I am in Omsk, because I myself moved from Omsk to Moscow to study, so, and at the moment I live... Well, I'm just visiting my parents now, but in general I live in Moscow. M. I see. And will you be a psychologist? N. Well, yes. This is by design.
And there's this very thing, I don't know when there will be grandchildren, when I'll get married, that is, there's this very insistence on a certain way of life, there are some expectations from the parents, from society, and I feel them, but at the same time I kind of went through psychology and psychotherapy, that is, it all seems to help me, somehow my beliefs, to live according to my own beliefs, and not social ones. But I still feel like it's frowned upon. And for example, when you don't listen to someone, it's condemned. There, when you are, as it were, in the minority, this is also condemned, that is, it seems even to me now, this situation, which is the war with Ukraine, that, as it were (.) some people who are vehemently opposed, or something like- then, in general, some people stand out from the masses, they are always, well, they are always perceived negatively, it seems to me. Here. And when, for example, you are different, it's just somehow scary as if people can be different, and this seems to me to be such a problem that it happens because of this condemnation that you are somehow different. Here.
M. It turns out that the Department of Psychology helped me to be more independent, if I heard correctly? N. Yes, it seems to me that yes, and it seems to me that it's just plus the people there, my friends and my husband, it's like, that is, we somehow switched to a more healthy (npt), healthier kind of then the atmosphere, somewhere there it is normal to experience some emotions, well, emotions such as anger or hatred, or... Well, in short, that is, which previously seemed to me that they should not be experienced, or that it is bad to experience them, or something else. Well, in short, there is no plus about family (npt) there (npt), that these are all sorts of non-adaptive patterns and so on and so forth, but how very upset I would have been at first when I took this subject, that this is how bad everything is and all that, but then I thought that, in general, this happens not only in my family, it happens even much worse than in my family, for example, and that parents, they just seem to try as hard as they can, that is, they seem to they also draw conclusions for themselves, based on those who raised them, based on life experience, and what they learned is what they passed on to us, so, and we can then also take it out and pass it on, and so on it will always be like this, and therefore it seems to me that this is, in general, a positive dynamic, that it is, in principle,

enough, and not what you say there - this is bad, this is not the same, this is not adaptive, this is something else... That.

And then, and also the fact that I was probably not afraid to take specialized mathematics, because to become a psychologist you need specialized mathematics, and it was like, it was very difficult for me, and every time it ended... Andrey, my husband, he seemed very He’s good at math, but he tried to explain it to me, I didn’t understand, it ended in tears every time and all these tutors and all that, I didn’t like it at all and I literally forced myself to learn it. But I’m like, I want to take psychology, I’ll pass and I’ll never need it again. Well, how important it was for me to pass it well and then I passed it, and I, I had such, well, it seems to me that such a low score for admission, but then I was very... At first I was upset, then I was very I was happy because it was an entrance exam for psychology and that I was going to get into it after all. And I was very happy about this, and my decision, and the fact that I passed these points, and my decision to go to the open day and to enter the Higher School of Economics, although I did not see other universities, that is, I did not go there and didn’t scout anything. I sort of decided that I needed to go there.

N. I would rate it a 10, to be honest, because my parents, my dad, were very much against me going to the psychology department, he wanted me to go to law school, I have a mother, her first education was in medicine, then she studied in psychology. went, but she was like, well, it’s like I want to be like her, she thought, here. But I didn’t go there because of her, I was just interested, as if about the people, there, like with the people, what’s in their heads, how to understand it all, that is, it was somehow for me it was interesting about behavior and emotions, so I kind of... And so my mother says something to me: go to these courses, go to these courses, there’s something else, and I say no, I won’t go, I’ll decide for myself what courses I should take, or my dad says, I need to go to a master’s program somewhere to become a lawyer, or something like that, and I say like, no, I don’t want to, or whatever Here I am this year, well, in the next academic year, for example, I won’t study, because, well, like my parents, they would rather have me go to a master’s program, but I just understand that I’m tired of studying (laughter) that I can’t, (laughter) I need rest. (laughter) And that I just don’t understand where, what kind of master’s program I would like to go to, I didn’t understand, and therefore I didn’t go anywhere, and how would I make this decision for myself, how would I want to work and understand maybe there's a better place for me to go. About the job, I would probably rate it a seven, because my first job was in my dad’s sales office, that is, my dad, he owns a construction company, and I worked in his sales office. But I wanted to work myself, as if I wanted it myself. And I kind of came there every day, stayed there for some time, they already told me like - go home, but I was like - no, I still need to do this, and I tried to come up with something there, whatever didn't come up with it. Here.

M. What about making everyday and important decisions? Here, in fact, we are, of course, slightly duplicating what we have already discussed. N. Yes, yes, I understood. (..) Well, I think, it seems to me, let it also be seven, because I, well, in general, it seems to me that this is a process that there are no correct ones... In general, I’m kind of really worried about there right, wrong there, or how it will affect others, or something like that... Well, in short, I worry about this in every possible way, and it seems that the more I work there with a psychologist, well, the more I see, that no matter how people react normally to my decisions, the more calm I am about this, the calmer I begin to feel about it, so how would I consider that it’s seven, there I’ll just be there later, well, what’s my attitude specifically towards making a decision , it’s very similar, it’s quite saturated with anxiety, so I wouldn’t say that it comes too easily to me, but I kind of understand that I always make adequate decisions, plus or minus, I just have problems making decisions, that’s it. And the choice of lifestyle, leisure, well, right now I would probably say that (..) six, because I think that (..) well, I just feel that I’m a little burnt out and tired after studying there, after school, when things weren’t

very good there either, at the university, when there were some difficult moments there, that's it. And it seems to me that I just need to recover now, just somehow settle down, that sometimes I get shaky, like how I behave there, or how I need to do or something else, well, like a lifestyle, and for me, I'm just now focusing on the fact that I need to rest, and otherwise be more careful with myself. Here. And about leisure, I don't really like spending time in company, I rather like one-on-one communication, and well, I kind of, (.) well, I don't do any supernatural leisure, like, for example, I knit there, or maybe I have some kind of hobby that is very accessible, well, drums are not very accessible. That is, I disturb everyone, and it happens that they irritate everyone, but as if I could occupy myself with something, I don't feel like I'm very bored, that is, in general, in every possible way... I sometimes scold myself myself for rest, that is, it's as if I can't rest, because I'm not tired and all that. But this is again a balance, there is a work life balance and therefore... (...) Well, about leisure, I meet with friends, I read books there, watch films, some educational things there, well, educational videos on various there platforms. Well, in general, I do, it seems to me, what I want, what interests me, and it seems to me that this is enough. Here. Romantic relationship. (laughter)
M. What do you mean, what didn't coincide? I didn't quite understand? They said that it was possible, but... N. No, rather that they... Well, for example, dad said that you should, for example, not lie, that it was better to be honest, and then it turns out that he has a second family there, that he him there... Well, in short, (laughter) some secrets from us, roughly speaking, that is, that he says one thing and does another. And that is, as it were... It doesn't work out, then there is no clear picture, you still understand that there is this duality, and not... (..) Well, what is it... And you begin to think like about some of your actions, that's it. That's what I'm talking about, that there weren't any where, for example, they told me - go and do it, for example, my mother said - go there, do something, take an interest there, something else, if only I had such opportunities as you have, then I would have done this, that, that, but she, despite the fact that she seemed to have opportunities, she practically sat at home with us all the time, that is, she developed, some She worked through her own problems there, with the help of psychology she studied there, but she didn't seem to really realize herself and it was also like this was a call, like go do something there, realize yourself, but she herself didn't do that. And it was as if I was expecting that if my parents said something, then they would do it, but that didn't happen, and so I had a question, like, how can this be, like, people say one thing, but do another, And I had such a conflict. Here.
N. Well, it's like in an interview, where do you see yourself in 10 years? M. Which one? N. Well... So, now, I'll think about it. Well... I see myself, that here I am doing something, something that is important to me, there I have some kind of project, some kind of brainchild that I like, which I want to develop in every possible way, and what I like I would, well, that is, I'm the same with my husband, I really hope so, that everything will be fine, and that I have the same friends that I have now, the same relationships with people, which is like -that's it... (..) Well, that I'm probably happier than now, maybe that I treat myself kinder, that maybe I have some kind of, that's when... (h) Okay, now I'll think about it some more. (..) Well, that I somehow help people, well, that is, either this is a consultation, or these are some other projects to help someone. But it's just that I help someone, I do someone's life is better there... This is how I would like to do it. Here. Well, and accordingly, so that I have some kind of balance in this and that I am healthy, that is, so that I do not have workaholism, because I see that my dad has this workaholism, so that I have some kind of there was a balance between personal life, some kind of family life, and work and fulfillment. This is some kind of ideal picture for me now, well, some kind of dream, here. I hope that's what I meant, as if I answered the question in general.
M. Do you have any hobbies? Besides work. A. Well, probably yes, part of it is the field of education, psychology, everything connected with tutoring, with... (...) Drawing, exhibitions,

studying, well, I don't know, the work of artists, this is also connected with literature, Let's say now, the main focus of interests is the book (npt), Annie Lee Lang, which describes art, artists, and, in principle, the concept of loneliness. And how artists reflected it, how it is depicted in culture, in what images. Here. Something like that. M. Great.

A. So, training and professional development, I already said that. Health choices. If we talk about the second point, then for the last year, but probably I have had more changes in six months, because there was a change of place of residence and somehow I concentrate more on it. Because the choices that were made in the previous six months were not in favor of my health, but there the only important option, or rather there was a choice, was to go to a psychologist. To deal with this destructive story with the leader, with (sigh) (..) change of position. Here. And if you look more towards the physical rather than the psychological, then this is probably a choice in favor of taking care (.) of some kind about yourself, to searching for, well, understanding in general your health there through the body, changing a little nutrition, attitude towards sports, and so on Further. Well, something like that, I returned there again, to proactive history. Job. (..) One important thing, well, it seemed to happen to itself, it so happened that for a new position, again over the last six months, first there was the building of horizontal communication, despite the fact that there is a manager and subordinates. Yes, I am the director there, but these are still subordinates, a turquoise organization was built. There was this, well, how it was clearly articulated, said internally in the collective, in the team.

M. Basically from the elections. A. Well, after all, what is still very much unclear and difficult for me is the case of accepting responsibility for the whole, the position of a manager. M. Yes, I remember you talked about this in great detail. Yeah, that's what happened. A. This is really very complicated... Well, it seems to have been partially worked out, this situation, in psychotherapy, but not yet fully resolved on some emotional level, it also seems to me that this has created some kind of trauma, (laughter) here. In terms of what was wrong, why was it wrong, right? M. Yes, why the wrong choice, do you think? A. Because, again, it was probably from the position of - well, who else? Well, very often I have such a choice when there is no other outcome, well, that is, some kind of critical point. And only then do I, as it were, completely regroup, into some kind of monster, make this decision, well, that is, I say this - yes, I can, we'll do it this way, I'll pull it off, but somehow, as if at a critical peak, I take upon myself the whole responsibility that is possible. Well, as if this were directly noticed, and then it usually works out. (xxx) But in this situation, which was and where my choice was, as it were, wrong, I underestimated it. Well, that is, the gap in this critical situation, the gap between what I can do and what definitely won't work out, it, well, happened more. Revaluation. (..) Here. M. Yes, but how do you feel about this situation now? What thoughts or feelings might arise about her? A. Well, it's cool that it was the first thing. Second, an important decision was made that in the next couple of years, I don't know, I won't take a management position at gunpoint. Not yet, well, no, I won't go there again for now. Just now I started, well, I kind of started to sniff out, if possible, from this state when you, well, got a very strong electric shock, well, you just intuitively won't get into it anymore. Here.

Another important choice, which in general, if in the future 10 years, then (..) it's probably closer to the family. Well, that is, as it were, a choice in favor of (..) building long-term relationships, and another related choice - health. Well, that is, as if I don't want to waste what I already have, because our body is only depleted, but I want to somehow fill it up, maintain balance, different areas of life that affect health, ultimately well-being, and the feeling of happiness when you feel good. This is a choice in favor of, well, taking care of yourself in different areas. Well, that is, starting there, I don't know, with some kind of tests, check-ups, regular support for physical fitness, psychological health, such a minimum of some kind of psychological hygiene, then yes,

this is directly connected for me with family relationships, because in Ultimately, if you make a choice, then for now I have the feeling that I am not childfree, but I still want some kind of (.) continuation of the family and continuation of the clan, if you can call it that. Again, I want healthy children, a healthy child, and this means that I must be healthy. And the other person must be healthy. Therefore, this is a long-term construction, with certain resulting requirements for a person. (..) And probably not by requirements at all, but by similarity of beliefs. When they look in one direction. Well, probably for 10 years, well, another important one, this is first, in the short term, somewhere up to 5 years, a period of time to take, then this is a set of expertise and experience, packaging it and going out not to work for someone, but for myself.
K. At first I wanted to go to become a military doctor, but in the tenth grade, when I found out that girls were not accepted into the military academy, I decided to go to the Lev Nikolaevich Tolstoy University, because a friend of mine studied there, and she responded very well about this university, I decided to go there too. I just have a similar direction, working with children, with youth, and this is closer to me. Well, it just happened that I love chemistry and biology, so I decided to go there.
M. You can go straight through the list, you don't have to arrange them. K. Training and professional development is... Probably there will be seven, because (..) you always have to learn everything, and sometimes it's very difficult to allocate your resources and brains, (laughter) let's put it this way. And do one thing. Health is five, (laughter) it's either there or it's not, work is also six or seven, because... M. Wait, let's go back to health, making specific decisions. In terms of health. K. Regarding my health, everything seems to be fine, I feel good, but sometimes there are times when I get sick, (laughter) Covid and so on. And I try to recover as much as possible. But if I get sick, then I don't do anything, it's immediately minus work, (laughter) minus friendships, only the place of residence will be there then. (laughter) At maximum. M. What about choices, such as choosing which doctor you need to go to, or even understanding whether you need to seek help or not? K. Choosing a doctor, I don't know. I somehow... M. How difficult or easy is it for you? K. No, it's easy for me to choose a doctor. I just see, either from reviews, or using word of mouth, that this doctor, we went to see him, he's good. I'm so good, I'll go to him too and everything will be fine. In principle, this is how it turns out, so I don't have such a difficult choice here to choose a specific doctor. Well, usually, I just probably didn't get sick with anything serious, no matter where it depended on me... The doctor's choice is between life and death. Therefore... M. In general, you said that you rate your health as a five, then what is the difficulty in making decisions regarding health? K. Health, difficulty making decisions, (laughter) giving injections. This is where it's difficult. (laughter) Or force yourself to go... It's just that when you're sick, you don't really want to go out. M. So it's difficult for you to understand whether you need help or not? K. Yes, I can't, it's sometimes difficult to assess the extent of my illness, it just seems to me that even with a temperature of 38 you can still do something there and at work, everything is a doctor (NPT) in principle (NPT) and you can live.
M. And now I will ask you to tell me about one story of a successful choice, and also, please, in accordance with this layout from the chat. K. Successful... (..) Now, we need to think. (...) Something... (...) I thought, I think it's good that I changed one job for another. Before that, I worked in another place, not even at a university, but in a clinic, well, in a hospital. And this is very, well, at first it seemed to me that this is very cool and great, because the equipment is new, but then I realized that this does not give me any career growth and if I stay in this position, I'll be there (npt). Then, but this also happened at school, then they gave me... Work, a feeling of extreme fatigue, every day. And it's like (npt) Groundhog Day. And I realized that this had to stop somehow, otherwise I would simply burn out at this job. M. So you also seemed to be guided by your emotions? K. (..) Yes. M. It seems to me that you have someone else there? K. Yes, now there

are students again, we should have opened another classroom for them. All. (..) Well, it seems like I told you everything. M. No, more about feelings and thoughts? K. Oh, (sigh) feelings, then this situation, (laughter) it seems to me that this whole situation sucked out any emotions and feelings from me at that moment, because working in a hospital is very energy-intensive. And now I'm looking at it too, the same thing, the only thing I've gained is experience, put it in my work book and just to fill out my resume. M. Tell me, Karina, how do you determine which choice is successful for you and which is unsuccessful? How do you separate them? K. (..) Successful, if I feel some kind of return, I feel needed. And unsuccessfully, if I feel some kind of return, I feel needed. And it's unsuccessful if I feel like I'm wasting time and wasting it.
D. It turns out that at the age of 11 I was told that I had the fourth degree of scoliosis, idiopathic, and that this could no longer be treated, perhaps I should have started going to some massages there a couple of years ago, doing something, but I had already encountered with a severe form of this scoliosis, and they told me that... Well, I was again given a choice, I remember how my mother seriously decided to talk to me, at the age of 11, that Dasha, you have such a situation, you can (..) live with this scoliosis, that is, it's like, well, I would have developed a hump, very strongly, it had already begun to appear, or I would have to undergo surgery, and they would put me in a metal structure for life. Into the spine. And when you're 11, you don't really understand that they're going to do something to you now, and you'll have to live with it, well, for the rest of your life, and I made up my mind, and basically, my mother told me that I walked this path very steadfastly , and lay in the ward after the operation. The girls who were 10 years older than me were there, they cried, it was hard for them, and my mother said that I went through it all with courage (laughter). Here. This is probably also an important path. M. Yes, I really sympathize, well, it's really a choice, and it's such a significant one, and I think that you are probably faced with some of its consequences. Does this have anything to do with tennis or not? It seems like... D. My mother thinks yes, that it's because of tennis, but I think that... (..) Perhaps, but there is a reason, there is a reason. Perhaps this somehow prompted, well, aggravated the situation, but obviously it was not the reason. Most likely it's something genetic, perhaps I had crooked feet from birth, and they tried to correct them for me somehow, here. Did not work out. And you understand, yes, that when the weight is unevenly distributed, then all sorts of scoliosis results. That's probably because of this.
Then health. Well, from the last one, probably... But this is probably some kind of funny situation, there's not much choice here, I, I felt bad, not so long ago, a month ago, and I called an ambulance, I didn't want to call it for a very long time, because that it always seems to me that my situation is not serious enough to call an ambulance, for someone it is now perhaps more important, more necessary, so. And when we arrived, it turned out that the situation was very serious, I had some kind of intestinal inflammation. And they just gave me a choice - to be hospitalized or not. (laughter) And I, too, somehow without really thinking, decided that it was necessary, let them check me and all that. And it was also an unpleasant situation that I was out of work there for a week, and I was ashamed, but I thought that if this, (..) if I don't get cured now and then it all somehow piles up, and it will be like like some kind of lump of snow, just like that. Well, in general, some more pain will be added, and then the consequences will be more difficult to deal with than now. I hope I made myself clear. (laughter)
When I went to the hospital, I didn't immediately tell my parents, only when I felt good there
want to be the kind of person who (..) takes responsibility for all the negative events that happen to him, and for example (..) does not ask, well, that is, it's normal to ask for help, but when you really need it, that's it. I (npt) did this, and I started to practice it now, and if I feel bad, I don't call right away, but before I immediately picked up the phone, immediately dialed and cried. (laughter) And

now, first I’ll cry to myself, listen to music, maybe talk with friends about some abstract topics, and then, when I’ve calmed down, I’ll share. Like this.
How could it be, what else? (laughter) (..) I think, I think. (laughter) I still have something from childhood, like, let’s say, principle, choice, I never drank alcohol, well, cigarettes, that’s understandable. And even when I... Well, that’s how it is in Russia, they are always trying to offer something to you, and as if in theory, the majority drinks and considers it normal. (laughter) Yes, and when you don’t drink, in general, they are all so surprised, well, come on, well, try it, (laughter) but I always had a clear motive, this has been since childhood, that I am not going to drink and I will never. Well, as an example. Well, even when, relatively speaking, I was moving into a hostel, the neighbors somehow, well, there are three of us, and they suggested how to celebrate a housewarming, they asked me - will you? I'm like, no, I won't. (laughter) And they were like - well, okay. But I always hear this phrase - you will start someday anyway. That is, I don’t drink at all. Not for the holidays, well, it doesn’t matter. Just never and not at all. Here. This, so to speak, (sigh) is my principle and choice. (laughter) (...) So, I don’t know how many minutes have already passed there. (laughter) It just happens, you don’t remember everything right away. (..) Damn, I guess I, I can’t think of anything yet, it’s hard to come up with, that is, come up with.
M. But you also said that mom changed a little, softened her views. What does this have to do with? L. Oh, (laughter) with her life experience, probably. Well, oh, in general, yes... (..) Well, she looks, reads these psychological books, and something else. In general, it develops itself, so we plant it like that. (...) Because she doesn’t like her life, she wants to change something in it. M. Lisa, was it really noticeable to you that suddenly she changed there and became softer? Or how? L. Yes, yes, she just talks a lot, and since she has no friends, in our city, well, they were, they all went to Tyumen. (laughter) And a new friend appeared, and she also left for Tyumen, (npt) And so it always turned out that she was talking to me and all these topics, well, I don’t know, she talks a lot in general, as if about herself, then about how she lived there (sigh) then she speaks about herself, about her changes. Or it says that you can reprimand me there if you see that I’m going too far there. Well, in general it was not difficult for her. (laughter)
..) Rather, this is how it is, somehow significant, this is health. (...) So, health. (laughter) (...) I would probably classify it more for me as some kind of sport, some kind of sport, (..) they are connected. And, (npt) I also said that alcohol, (laughter) (npt) let’s say, refuse (npt). That is, I don’t want this one (npt) yet, but even before that, this is the last one. And as for sports, I, I have a new kind of sport, that is, always, (NPT) development, I liked it, there was a subscription for eight classes. This is something new for me. But as they say, well, jumping on a trampoline, doing (npt) or something like that, in my opinion. (npt) Then work. Work, well, work, here I chose, “Yandex.Food”. I was choosing between Yandex.Food and Delivery Club, well, it seemed that in Yandex.Food, well, how to work there, on the contrary (npt), with large orders, but since I could, I had initial information... M. So, now everything is turned on, now, I hope, everything will be heard well and there will be no interruptions. L. Well, yes, I said that I was choosing between Delivery Club and Yandex.Food, and there was more difference, that is, I went to Yandex.Food. Oh, and then I also worked at Pyaterochka, but I initially went to the order area, since it turned out to be there from morning to evening, and I needed (..) evening part-time work, or for a number of hours. But, I said, they offered me a cashier job, and I, I had a choice... M. Yes, Lisa, something went wrong. Yes, about the order picker, and you were offered cashiers. Here in these words. L. Yes, I had a choice whether to agree or refuse. And I thought, okay, I’ll agree, I’ll try, why not. Although it was a little scary and exciting, in the end everything, so to speak, worked out, I was trained and I worked calmly, so to speak. Here. Later, but then I had to leave, so I quit. But I warned that I wouldn’t be around for long and, in principle, (laughter) since they didn’t quite suit me, (laughter) and it would have been a choice in general, (laughter) probably to quit. And, so, (npt) in my

opinion, (..) I would roughly give an example, like choosing a vacation. Since it's difficult for me to get from Rostov to Nizhnevartovsk, so to speak, at the moment, since the airport is closed, and you can get there by roundabout routes, but it's expensive. Because from Moscow (laughter) to us one way it's 10 thousand, but I just had a direct flight, I could fly (npt) when I was lucky in principle to get it and when you already flew for 3, and for 5, and for 7, then you don't want this, well, yes, you don't want to go one way for 10. But in principle, it happens, it's a lot, so to speak, well, I would come to my city, but there's not much there, there is nothing to do, and in general, my mother and I agreed that we would meet in the summer, but not in the city, but somewhere, so to speak, for us on neutral territory. Well, we went to Sochi, that is, she (npt) flew, and I had to get there by train, and... (...) Well, we generally chose tickets, well, where is the best place to go, we chose these ones, where Sochi is. But since I was already there, a year ago, for a few days, it was somehow very, there is such an example, but... (sigh) So, (..) friendly relations. (...) Well, choice. (..) Well, here, probably, who would you like to be a friend, (laughter) and who would not. Don't know. Or, let's say, where there is a person like this, I would like someone... (sigh) (...) So... (laughter) In general, they offered me to take a walk there, and I, (..) let's say, I didn't want it in general. I don't like this person, so I don't want him to be (laughter) my friend. And you probably... Well, I kind of refuse. Don't want. Don't know. (laughter) What's a better example to give, honestly. Well, I don't know how I could have one friend here, (..) I don't even know. I'm moving on for now. Place of residence, regarding the place of residence, by the way, I live in a dormitory, and initially I didn't want to live in a dormitory, because, well, it seems to me that here, well, all sorts of events are held, as if there was a place to sit, let's say I had to film apartment or something like that. And plus I have a hostel, it's very comfortable, well, I just won a competition, (laughter) and the hostel in which I live, it's like, well, it's an apartment type, and that's why (laughter) it's here for me in general, not only that , that I'm like this in a hostel, where I have connections, but it's also almost home. (laughter) But, of course, I didn't know which one I would end up in, but initially I wanted to go to the hostel, so that there would be all sorts of these events in which I could participate. That is, renting in one there, in an apartment, is not interesting, it is boring. Here. (...) Everyday and important decisions. (sigh) (..) Well, about important decisions, everyday ones, probably just like where, where to go, if everyday, where to go, to have fun, to see. Well, also, for example, I took part in the race there in May, for me it was such an important decision. In such a major event, because it is not held here, and I have never participated. The Russian Federation race, which took place in May, I... Well, it costs money, and you kind of have to decide on it. (laughter) I took the plunge, bought it and didn't regret it. And also (..) I liked it, I finished the race (npt) I also decided, I realized that I liked it, that (laughter) I need to train for it, and that, well, more.
M. Yes, but do you have any decisions in life that you consider unsuccessful? L. Solutions... Health related? M. In general, not only related to health, but are there any choices that you consider to be completely unsuccessful? L. Yes. The elections were unsuccessful. Well, (..) probably yes, yes. (laughter) But for me everything is connected with sports. (laughter) In the eighth grade, no, not even in the eighth, but earlier, in the sixth grade, they offered me to go to... (npt) To the All-Russian Olympiad for schoolchildren in physical education, but I refused then, and in the seventh they offered me, but I She also refused, or rather, at first she agreed, then for some reason she refused. In the eighth, (sigh) I also refused, and in the ninth, (laughter) and only in the tenth I came, and even then I agreed. And... I regret that I refused before, because I really wanted to go to the final stage, that is, to Russia, which took place then in the eleventh grade, in Kursk, that is, you go through the regional, well, first the school, then municipal, regional, and final. And (laughter) in general, two years of preparation is not enough, and if in the tenth grade I failed there, then in the

eleventh, in the region, although I was a prize-winner, I took first place, but I was 17 hundredths short of Russia, and I was very I wanted to. And in general, I regretted then that I had not agreed to another school earlier. And in general, yes, and the fact that in addition to physical education there, let’s say I would also take part in mathematics, I somehow even thought about it... Well, I didn’t think about what the Olympiads are like, and what they give, and what in general is that? In general, then it was a bad choice that I refused. (sigh) In this regard. M. So you consider those elections unsuccessful, where you missed some opportunity? L. Well, probably yes. M. But out of these several situations with physical education Olympiads, which of these, well, which of these situations is the most unfortunate, in your opinion? L. (...) Well, what’s the worst? (laughter) The situation, well, (laughter) I didn’t have enough points, (laughter) for Russia, these hundredths... Hundredths, well, it’s just literally the whole point, or (npt) in gymnastics, or a second of running. (laughter) M. And you feel like you’re blaming yourself a little for not pushing through? L. Yes. I just wanted, and even more than to pass the Unified State Exam well, that is, (laughter) well, damn it, I still can’t come to terms with it. It's a shame. (laughter)
So I realized that as a result, you have not yet enrolled in any specific business specialty in order to do quests. But as for sports, why wouldn’t it become some kind of professional field, well, in which you would become, well, a professional athlete, why not this? L. Yes, it’s interesting, I mean, in general it’s, well, firstly, we don’t have that many sports in our city, and if it’s all over there (npt), then basically, in principle, then in our regions... Well, even if not only all kinds of sports, Moscow has everything, I recently made such conclusions, but in the regions, in one there is no such thing, in another there is no such thing, in a third this is not the case, and since we have a lot of sports, I started somewhere with basketball, then I liked it, before I was banned anymore (npt), then I went to sports tourism for 2 years, it’s not like It’s just mountaineering, but that’s the point, there’s a program there, on the ropes like that. In principle, I liked it, but for some reason I got tired of it. I don't know. (laughter) Honestly, I got tired of it and just quit. (laughter) The same about athletics. And athletics, it's even more boring. Well, again, it’s a cyclical sport, where you only do the same thing, and that is, we only have running and nothing else. There is no throwing or jumping, just running. And I'm not interested in running. (laughter) It’s the same thing, in general, I’m truly not an athlete. I understand this because, well, I don't like it. Well, I don’t know, and somehow other sports, I didn’t try much, then I just somehow in the ninth, or even the eighth grade, (laughter) forgot that I was an athlete, and to some extent a sport was absent from my life, and then, in the tenth grade, I somehow remembered this, and here, when we were just preparing for the Olympiad in physical education, we have, in addition to, well, theory, gymnastics and running. And since I liked gymnastics, it’s clear that it’s not like real, professional, sports gymnasts, there’s a lot of other things, and I (laughter) by the way, I regretted that damn, it’s a pity I don’t do this, but they only take from childhood. And my mother told me that she somehow brought me when I was 5 years old, but they refused me. But I don’t remember this, they kind of told me that I was too big. (laughter) But I kind of liked it, and because of this I kept looking in Rostov for some kind of acrobatics for adults, gymnastics for adults, that’s it. And trampoline jumping, but it is combined with gymnastics for adults. But the problem is that damn, it takes a day. (laughter) Well, yes, sort of. And so, well, I didn’t manage to go to any professional sport, because I couldn’t find one for myself. M. That is, if, perhaps, when you were younger, you would have been sent, well, sent somewhere... L. Yes, perhaps.
Z. Yes, yes, yes. Here. Choice of lifestyle and leisure. I really want (laughter) for this area of activity to change for me, because over the past year, all I did was study. True, I don’t have time for anything else, I didn’t have time. I’m trying to change, I’m trying to change this moment, I really want to do... Well, in general, I would like to do yoga, go to group classes, I realized that I really like it when (..) probably a new team appears, and there are new people, meeting new

people, it’s very interesting, it’s something new and unusual. I would really like this area of mine to improve, because again, all I’m doing right now is learning. And this drains you and you need to recharge somewhere. Where can I get it? Well, I realized for myself that I would like to go to a slightly different space, to other people, and replenish my resources with the help of sports, yoga, etc.
M. Okay, let's now take a closer look at your elections, and now I want to ask about the elections that you may consider unsuccessful in your life, were there any? And why do you consider them unsuccessful? Z. (...) Yes, it was a bad choice in terms of health, so I already told you that I turned to a surgeon, and... Well, I regretted it. I had an operation that was not required at all, that is, in vain, they completely removed my nail plate just like that. Why this was done is unclear. When I came to a highly qualified specialist, a podiatrist, they told me that this was a complete disgrace, it was impossible to do this, and now we are correcting this whole problem. Here. That’s why for me this is such an important choice, a health experience. And now I think this is very important. When they wish you health, now I don’t just, well, health, happiness. Happiness, health - yes, this is what I need. (laughter)
M. Great, great. Yes, it seems we have now discussed some better choice. Maybe you can remember, for example, how a bad choice was made? For example, with an ingrown toenail, maybe you can remember your thoughts and feelings while you were going for surgery? Z. Yes, yes, of course. At that time, I was at home, not in Rostov, and we went to the hospital. In general, it turned out that I was immediately sent to a surgeon to solve this problem. I come to the surgeon and he looks at me slightly and immediately says - that’s it, operate. I'm like - what? And I had never had any health problems before. Everything was fine, no surgeries at all. Well, it was a shock for me, I was admitted to the hospital right away, that’s it, they left me, my parents brought my things, and the next day I was waiting for the operation, so. So it turns out (..) day X comes, the time approaches, they take me to the operating room, perform the operation, and the worst thing was that after the operation on the nail, well, it turns out that the nail plate was completely removed, and a bandage was wrapped around this, on this skin. It’s very tender and soft there, and it’s still a little unhealed, because an inflammatory process began from this nail, it was painful. Here. And they wrap this bandage around me and say, well, that’s it, now lie down and rest. Everything is fine. The next day it was necessary to do (..) soak the nail in a special solution so that it, the nail... The finger in the solution so that it heals. Here. And for this it was necessary to remove this bandage. I start to unwind it, but it won’t come off. He was completely stuck to this super soft, unhealed spot, it was just terrible. The time was probably just right... (..) Well, there was a day, I was sitting, I tried to soak this bandage in different solutions, a couple of hours passed, it didn’t help, we just started trying, well, tearing it off a little, that didn’t work either helped. It was terrible. We probably sat like that until very late at night, with the nurses, they tried to take it off for me, nothing worked, it hurts, it’s a super soft, tender place... In the end they told me, let’s do this to you. In general, well, it’s like a bandage, it’s even, applied to this finger, here. They cut off the entire part of the bandage and left only the piece that was glued to my finger. And in general, they tried to remove it with tweezers, pulling out each one of these... (laughter) A piece of lint from the bandage. It was terrible. It's just a nightmare. This also didn’t help, and I’m already in tears, of course, all upset, nothing is working out... I’m also a very suspicious person, (laughter) I’ve already thought of a bunch of things, that’s it, now he’s dried up for life, I’m like with him I’ll live, (laughter) upset, that’s it. Well, they told me to wait for the doctor in the morning. But that’s all that was left to do, and I ended up sleeping through the night with this bandage, and the next morning the surgeon came and in the operating room he made me some kind of, I don’t know, glued some kind of plate on me, and just tore it off with a very sharp movement. Yes, from this

soft place, they simply tore off the completely dried bandage. It was very painful, oh well, and it was all bearable, it all went away, that's it. I thought that was it, my problem would end there, thank God, everything was cured, everything is fine. So six months pass, the nail grows back, it grows very ugly, ugly, really, ugly, yellow, clumsy, all like this... I turn to the surgeon again, already here in Rostov, he says that Well, we need to do an operation and rip it out again. I think - what kind of person are you, really? Here. I'm thinking, well, are there any more humane methods of treatment, maybe it's possible to even it out somehow? They tell me that such treatment is done only in private clinics, this is a paid treatment, but they said - try it, maybe it will suit you. And I thought that I didn't want to suffer anymore, I wanted to know something specific from a specialist, some exact answer as to why this was happening, not just to rip it off every time, but to find out the reason. And what to do with it. And I turned to a podiatrist, in a private clinic. And now this doctor and I are still aligning my nail plate, everything is fine. Here. Of course, I was upset that I had experienced all this, but probably without this I would not have understood that it is very important to go to, well, really good doctors and it is better to pay a little for your treatment, and be sure that they will really help you and correct you your problem, than every time, as they suggested to me, tearing off a nail. Here. M. What did you think, what did you learn... What do you think, what did this situation possibly teach you? Z. (..) Taught? Well, probably after all (..) until this moment I didn't really think about the qualifications of doctors, that is, you come to the hospital, doctors, they're all doctors, they probably know everything, smart people. And now, you look at it from a slightly different angle, well, at least I do. I pay attention to certificates, I pay attention to where I studied, what merits I have. Well, in general, for qualifications and I think this is still very important. And also thank you that we live in the modern world, we have the Internet, we have the opportunity to look at people's reviews, at a certain doctor, for example, or a clinic. And now, depending on the reviews, you can make a decision whether or not to go to this specialist. Here. Probably, after all, yes. The situation taught me to take health more seriously, this is not a joke, and to pay attention to who will treat you. This is also very important.

Then I went to school, and in the first grade I did synchronized swimming. All first grade. And then, the workload became so heavy that my mother suggested that I either change schools, or that I study in the second shift and be able to devote all the remaining time to training. Or stay in school and quit swimming. And she didn't decide this for me, she said that it was entirely my choice. And then, during that period, I decided that I would quit swimming. And I'll stay in school. Well, it seems to me that then I chose friends and some kind of social life, it seems to me, at the age I was, as if friends were more important, and sports had never been some kind of meaning of life for me. I went, I basically liked it, but he was never a priority for me. But now I think that... Now I don't think at all, but I used to think that if I had stayed, I would now be some kind of master of sports or take part in competitions, be an athlete. And life would have turned out completely differently. But nevertheless, I never regret the decisions I made, in any way. I studied the same way, in the same school from first to eleventh grade. (..) I was like that, not very much, but that is, not a completely withdrawn child, but also not one who directly craves attention, that is, I was afraid of public speaking, but at the same time I communicated well with everyone, well, I was the kind of person who He seems to communicate with everyone, but only a few people are good there. But everyone treated me well. Then, the elections, where... In principle, even when I was in school, my parents always put the whole choice on me, they didn't decide anything for me. Which sections should I go to? I also tried a lot of different sports, only because I wanted it myself, and I left the same way because I wanted it myself. And it seems to me that this... Well, to some extent it's probably correct, perhaps those who... Those parents who want their child to actively engage in some kind of sport, who have their own ambitions, they somehow insist, but I, on the other hand, have tried a lot of different sports throughout my life, and I realized what I like to do. I still swim, for example,

like it was with synchronized swimming, I realized that I like swimming, I still go to the pool. And when I started doing athletics, I realized that I like to run, but kind of (..) not... But I only like to run, for example. I don't like everything else we did. And I just left, just doing it for myself. Well, that is, it seems to me that we need to somehow give the child a choice, although they already asked me then... (...) What... (...)
M. If in terms of the degree of difficulty of the choice, how would you rate it between one and ten? Well, you actually talked about a series of elections, but on average, how do you evaluate your passage of these forks? You chose between a bachelor's degree and a bachelor's degree, this advanced one, you chose not to work there, but you chose to work there. J. It seems to me, seven, approximately. M. Thank you. J. Health, I don't even know what I can say about this. Because I've always tried somehow... (..) Well, I just have very good health, some kind of good immunity, I've never really... In terms of paying some attention to my health, I make a choice in favor of some kind of regular check-up. I go to doctors, donate blood, take vitamins, and exercise. I'm trying. Sometimes I get very lazy, but I'm trying to instill this habit in myself; in fact, I still really want it to be some kind of regular. Also, I go to the doctor, well, that is, I don't have the position where I will never go to the doctor in my life, like some people, they don't like it at all, no, I'm somehow very sensitive to myself, to my health. It's better to go get checked there, once again, than not. M. And how would you rate the severity of choosing these, developing habits, even laziness, but it's important to go to the doctors, get checked, get tested, how easy or difficult is this for you? G. Four. M. Four yeah. Thank you. J. (..) And work, for me it's a little bit like that, for now... (..) Well, I'm still studying there in general, what suits me, what doesn't, I don't understand yet, I generally... At the fact that it seemed to me that I was a rather diligent person and that I could somehow do some monotonous work for a long time, but it turned out that not as long as I thought. And I'm still feeling out in general what type of activity I'm comfortable doing for a long time, because somehow, well, in my head it's as if there should be one activity for, well, a long period of time. But I always somehow get tired after a couple of months, I need some kind of change of activity, or some kind of development. That is, right in one place, something is the same, like accounting, for example, or in a bakery, doing something the same every day, I start to get tired and fall into some kind of despondency, which is like this it will happen, I immediately have some thoughts that it will be like this all my life. It's the same there every day. That is, I also realized for myself that in this regard I need some kind of variety, and I want... Well, my dad, for example, never worked in an office, maybe that's why I subconsciously somehow chose a profession similar to his . Because he travels around objects all the time, doing something, something like this is closer to me, some kind of active activity. Even in terms of studying, I could never sit like this and constantly listen to something and that's it, that is, when we receive knowledge, we immediately put it into practice, some kind of more creative training or something. And apparently I'm inclined to have a job, I also need some kind of work, well, more active or creative, I don't know. I'm still searching. Choosing a job is actually quite easy for me, I don't know, maybe (..) 6... I somehow start easily, finish easily. (...) Well, six, probably not, let's give five. Let's put it at five.
J. Because it's such a difficult topic, because I still... (..) Well, that is, I still live, for example, with my mom and dad, I communicate every day, I'm still building some kind of relationship with them. I myself go to a psychologist, somehow I'm already trying, from the side of some more... Well, not a teenager anymore, but some more adult person, I'm trying to look at any situations from both sides. Both from my position and from the position of my parents. Before that, at school, I always had some kind of very blaming position. That is, I thought that the way I think is exactly like that, and I never really wanted to consider any other points of view on this situation. Not how I see it from my side, but how other people could see it. Well, my parents. And that's why it's

always been very difficult for me to understand them. And I was like that, a rebellious child inside, that is, on the outside I was quite calm, but I always disagreed with them, with something, I always somehow kept my opinion about you. And now I'm just trying to take it easier, it's easier to accept that someone may have a different opinion and that's normal. Other views on some situations. I don't know, I don't understand at all what choice means in terms of relationships with parents, but (..) in any case, it was always somehow very difficult for me, I... We always had good and bad, then good again. It was such a thorny road.
M. I think that this decision is also very difficult, and somehow very worthy. But you speak warmly about your father, and about your similarity with him, in principle, in part. J. Well, yes. Well, I went, as soon as I had the opportunity, I immediately went to a psychologist. And I still go to him. We have a free psychologist at our faculty. They did it for us. This turned out to be a very popular service. A lot of guys go. And this is really very cool. Yes, I'm working on my relationship with both my dad and my mom, how it all affected me, and where in this whole situation I am, and where are my parents' opinions about me, (npt).
Somehow it's always been (...) hard for me to open up. Yes, it's probably some kind of, well, childhood trauma. I'm still working with this, with a psychologist, and it's hard for me to open up to people, because it's as if... (...) It's as if, when I open up, I start to take everything too close to my heart, and then it's very difficult I'm experiencing this. Therefore, in terms of friendly relations, choosing some new friend for me, well, probably an eight.
Now, I have some kind of frantic energy, I want to meet someone all the time, learn something new, go somewhere new, to theaters, museums, concerts, walks, etc. whatever. I feel like I'm being filled up by this, I don't know. So I went, when I went to a psychologist, somehow after that I became more open, and then people themselves began to reach out, after that. (..) Oh, romantic relationships, that's also a ten. I have a very hard time with this, because it's also about opening up, you have to open up to people, and at some point I start to quickly, quickly close myself off, as if when I understand that this is some kind of... Well, it's not like that anymore, (..) when I realize that I am becoming a little vulnerable, I, (..) my brain seems to automatically begin to push away, that is, it is so much so that I even (..) at first may not notice it, and only when I analyze it later, that is, everything is so quirky that it seems to me at the moment when this happens that the reason that I chose is really important, but in fact then I understand that I just had some kind of fear and (..) I just, well, sort of decided to run away. Now I'm actively working with this, but this one is hard. For ten.
K. Metaphorical associative cards are a tool for a psychologist so that a person associates his situations with the help of pictures. That is, it's easier to sort of reveal it all, from different sides, from different points of view.
M. Did you decide to do this yourself, or... How did this idea come about to do this? K. So I was on an educational shift here, at the Republican Center. And just like that, the psychologist worked with us, and she talked about these cards, which are associative psychological. I bought them because I was interested in all this, I bought them and they are generally so beautiful, (laughter) and I began to sort of rummage within myself, with the help of them, and then I realized that I was more interested in going deeper into all of this. climb, and this just became popular, these Tarot cards and so on. And I think - why not? I bought them. And so it went from there. And astrology is always interesting, because from childhood it's like, oh, who is he according to his zodiac sign, do we match with him or not? (laughter) Something like this.
Now I'm asking myself what I want. I also recently found such a method, with a coin, that is, what should I do, do this or that, and instead of going up to my mother or asking my friends, I take a coin and if I'm not satisfied with the answer, it means that I've already decided what I want. For some reason I'm just afraid of getting it out of myself, I don't know why this happens, but I

realized that what happens most often in these cases is that I already knew the answer. Here. It's very interesting, so I say, I watch myself, and I like that I'm growing. In terms of personality, in terms of the fact that it is separation from parents, this worries me first of all. Here.
I immediately signed up for the dances, as if to continue, because I liked it there and the team that was there, so I came and then stayed. That is, I thought that for an hour and a half twice a week, nothing would come of it, I would remain in shape, I mean physically, that's it. And in general, studying...
D. And I, you know... Yes, I have friends. I have very close girlfriends, and my boyfriends are also close. I also decided to take care of my mental health, since I can't go to a psychologist, since we have one free psychologist in the city, and I went, I didn't like it, because it was at the level of simply abstracting from problems. Here. That is, sometimes it helps, yes, so as not to overextend yourself, but sometimes not. Here. That's why my friend gave me a book for New Year... (..) "The child in you must find a home." This is some German psychologist, Stephanie Stahl, here. There are so many exercises, why we do this, that is, in general, it all comes from childhood, and inside us there is a child who... Ah, a gloomy child and a sunny child. And all our tantrums, or when we get angry, it's all a gloomy child, that's it. And we need to find a common language with him, and we also have an inner adult who sometimes succumbs to the whims of this gloomy child. Here. And there are a lot of interesting exercises there. I try to do this sometimes.
M. Yes, in general, undoubtedly. Are your relationships with your parents too distant, distant? Or somehow you quarrel, or... D. Sometimes they are very close, and most often these are just situations when we become close, which is extremely rare now, they make me feel guilty that I'm so bad and I offend them. But in general, well, I don't know, they certainly didn't do much harm to me, but purely morally, they did. That is, there is some kind of financial support at all. (..) But psychologically, of course, it's very (.) difficult. (..) Here. Varya, I'm a little busy. They are shorter in the kitchen. OK. In short... (..) So what should I tell you? M. I asked about relationships, about support or about conflicts, well, a little, in general terms, in order to somehow understand. D. My father drinks often, and as if before it didn't directly affect him very much, it was limited to the fact that we were walking along the streets, he was drunk, and just did all sorts of bullshit, I was ashamed of him. And then, in the ninth grade, when I began preparing for the Olympics, I studied them seriously for the first time, I almost didn't leave the room, because I wanted to seriously prepare. And my parents said that you sit so much and seem to be moving away from us, well, they fought because of this. And my dad also came home drunk, and my mother asked me to sit with him in the kitchen and talk every evening, he drove me to hysterics, and in ninth grade I can't even imagine how I pulled it out, but it was... Emotionally it was difficult . Here. And with my mother, well, I don't know, in general I've always been my father's daughter, well, I thought so, but for some reason I don't have a particularly good relationship with my dad now either. Mom sometimes, sometimes she somehow tries, like, when she sees that I'm really really bad, and that's not always the case. Here. But she doesn't take my problems seriously. Well, yeah, she doesn't take my problems seriously. Yes, they don't take my problems seriously. That is, if I say - I'm tired, I worked, they're like - why are you tired, what problems can you have at 18 years old? Yes, it's true that there are none. Here. Well, in short, not being perceived as an adult also depresses me. Here. (...) So, it's very difficult now all at once, in fact, the relationship with parents is quite complicated, that's it. And now I've simply protected myself from them. When they are at home, I practically don't leave my room. Now my mother is offended by me, and I gave up, I don't care what she was offended by, I don't even want to find out, let her really be offended, if she doesn't want to enter into dialogue, it's her problem. Somewhere in the middle of the tenth grade, I tried with her, no, or at the beginning of the tenth grade, I tried to talk and say that I need support, at least just come and

say that you're great, you can handle it, you'll succeed. And that's it, I don't need anything else. And this dialogue boiled down to the fact that I'm ungrateful, they spend a lot of money on me, they spend almost their last money on me, and I'm ungrateful. Then I got sick of them saying that they were spending a lot of money on me, so in April I went to work. That is, school and work were certainly difficult, but I managed. And since April, I haven't asked them for any money, I've bought clothes and something to eat here, well, I've gone to the neighboring town, all with my own money. And then they say something like this to me - why don't you go to the village with us, like you're at work again, you're cutting yourself off from us. Are we not giving you enough money? Something like that. Well, well, yes, I don't have enough money. So I went to work. Here. (..) In general, they are (..) children. M. Children? D. I can honestly say, they are not trying to change themselves, they are not trying to hear (..) me, that's it. It's quite difficult. M. Yes, I'm very sorry, what you're saying is very sad to listen to. And it's even so paradoxical, that is, well, it's clear that you're trying very hard and learning, and it's true, a lot of things are working out, and so, when they tell you that you're studying too much, and maybe you need to stop and go do something else, yes, I think it's very disappointing to hear. D. Yes, and when I wrote the Olympiad poorly and walked around upset, they told me that I just didn't study enough. Here. Something like this... M. Well, yes, in such conditions, of course, it is difficult to get support. Apparently we really need to look for it somewhere else. Please tell me, do you like being so independent, do you want to be more independent, or do you want to share this responsibility with someone? D. I began to notice that... Apparently I don't know at what level this works, but here... (..) somewhere inside I am still a child and in most situations, sometimes stressful, I begin to behave like a child. And then I, well, kind of think about it and... Well, I analyze it, and I understand that (.) did not behave like an adult in this situation. But it was necessary. Here. Well, in short, this independence from childhood, it actually affects me. Because even more so sports, they also tried like professional sports, all the work, and there, well (..) no study, no personal life, only sports. In short, this is it. You also need to be responsible. And now sometimes it takes its toll. Here.
M. What kind of sport? D. Orienteering. M. Wow. D. I also took up skiing. M. You can do a lot of things, it seems. D. Yes. M. Does this sport experience help you now? Even though you left him forcedly. D. Yes, it helps in the sense that... Well, on the one hand, no, I mean, wait, does it help that I left him, or the fact that I was studying? M. No, no, what did you learn exactly, what were you there and what did you do? D. What I endure until the last moment is a minus. So, let's say I get sick, and I won't go anywhere until the last minute, I'll endure it. I'm working until the end, I'm already feeling bad, I'll still do something, because I need to go to the gym... Or I just went to the gym recently, and I'm thinking like this - I'll do a light workout, because after my illness. But in the end I did it very hard, because somewhere in the middle of the workout I thought that I had worked very little, and that I had not finished myself off, and I need to finish myself off more, that's it. Well, (..) no, sport is just that, I don't know, I made friends because of sport. Here. (...) And in general, orienteering is a cool sport, it makes you think. And in general, the fact that you are running through the forest, I love this sport, in fact, very much, with my soul. Well, but as a hobby, not as a sport. Sport, it destroys people. Professional. And health, both mental and physical. Everything possible.
M. That's why you chose a different trajectory now, right? D. Well, not because of this, in general I just have a lot of problems with my physical health, I realized that I definitely wouldn't go into professional sports, well, science was interesting to me, that's why too.
M. I see. Are there some situations that you want to hide, on purpose and in general so that they don't find out and maybe no one finds out at all? D. (.) Probably self-harm. M. Do you do self-harm? D. (.) Not now, but through sports I can. Come to the gym and just... (..) To the point where I'm just falling. Here. M. And does this help you in any way? D. Well, (.) not to think about

something, I don't know. Just to finish myself off, probably. I don't know how to come home, go to bed and that's it. M. It's a pity, this is of course an inappropriate way, but as you say, it seems to me that you seem to have more different available tools in order to somehow deal with your condition. She showed me the book, told me about the exercises, and told me about her friends. D. Well, I'm trying, I'm trying to somehow get myself out of all this, in fact, I'm trying.
M. Thank you very much. Please tell me, you are in Rostov-on-Don now, right? And I understand that you are working, while we were agreeing, you wrote that you need to catch up after work, are you studying or just working, how is your life in general now? L. Yes, then I will answer in such a multi-layered way, I received my first education this year, a bachelor's degree, at DSTU University. In the Don State. Here. That's where I work. Here. It's like we've even gone a little towards individualization, here. I'm studying, I've now entered two master's programs, the first is my 39th, this is social work as well, and the second, 44/2, is in psychology and pedagogy. M. Ah, colleague that is. It's clear. Is the bachelor's degree also in psychology? L. Bachelor's degree 39, this is social work. M. Social work. L. Yes. I became interested in psychology in the second year, in the third, the therapeutic direction was closer, I studied there and realized that I also wanted to take two master's degrees and one of them first. M. So you will study in both at once? L. Yes. That's what I thought... M. Wow. And work a little more? L. Yes, and in different universities. Well, that is, in my 35th, also in DSTU, and I went to study psychology at the Southern Federal. M. So, the Southern Federal is Krasnodar? L. No, this is the Southern Federal District, this is also Rostov. M. Oh, I got it mixed up, yes. It's clear. Do you have any other hobbies or interests, if you have time for them? L. From a hobby, if it's an activity, yes, I really like to organize and come up with all sorts of different thematic events, fortunately, the university also loves when it is offered to do something, I probably won't name any right now, psychological hobbies, hobbies, but for me I like some practices, well, for which I am conditionally admitted, I mean going through, conducting training, of course that's it. I wouldn't call my work a hobby, although I enjoy it.
M. Can you think of, remember some example, from there, I don't know, from a movie, from a TV series, for example, I don't know, from another work, from a book, of a person who is independent and not independent? Maybe some celebrities, someone famous, or some bloggers, someone in plain sight? L. So, (...) I don't know directly whether this can be such an example, that is, interpret... (..) And in the work, well, Bukowski, there is Henry Chanaski, as his alter ego, the work " Women" is called, "Post Office" too, so, I remember the main character, in my opinion he is very infantile, he also lives the life that he wanted there, he is rather an example of this, he works there at the post office, with a huge number of sexual relations, beer, and it's as if he has nothing else in life. He didn't escape anywhere, and he didn't have to do anything. And if he's just so independent... (...) What's his name, I watched some interview today, I don't know, I remember, Mark Guarden, I think his name is. Something like that. March... Some psychologist, I listened to his podcast today. Some kind of person, I don't know... (...) I can give examples, so I look, I don't know, the rector at my university is an independent person, but I don't know (npt) work or not.
M. A person who enrolled in two master's programs and built his entire professional trajectory? L. Yes, it still is, I have absolutely such a sober argument for this, when I had the opportunity to initially enroll, I was interested, I liked social work, because I read disciplinary work (NPT), I liked the number of courses, and I looked at psychology, psycho-ped. But biology was a mandatory part there. And I thought that I had made up my mind that I didn't want to give it up. (laughter) And that's it, here I am, although I could force myself to do it. But she didn't. Therefore, it doesn't reach ten yet. I... If only I had just defined myself vectorially, yes. Although I don't regret a second, I like everything that happened, but I still think that I screwed up a little there.

And after sixteen, yes, the first independent decision I made, I remember, it was on nerves, consciously, I don't know why, why, it didn't happen, I lit a cigarette. I came straight away, and I didn't have a situation where I was wandering around somewhere, in garages, I came openly and said, like this. Here. Naturally, my mother didn't like this, of course, but due to the traumatic experience there, she somehow turned a blind eye to it then. There she said - I'm sure it will fall off you, but now, okay. Well, we'll sort of assume that these aren't some kind of steam locomotives, it's not necessary. She is the only, it seems to me, the wisest thing that she told me, she says - please, you can, just don't buy some crap for yourself there, it's better to let them lie there at home, if you want, take it, like. Well, this is something normal. Because again, she says - what's the point, well, I would take it away from you, shout at you, so that you would stop smoking? Nothing would change. I would (npt) where is it, well, I would be hiding somewhere. No, and it fell off. I don't remember, I went there for two years, I worked through the traumatic experience with a psychologist, and one day I just said, no, I don't need this anymore. I wasn't so drawn to it when it was like this - well, you want it, right? Please. How (npt) is it normal, let's not just somewhere, over there at home on the balcony, you want, well, that's it, so that I can be calm.
M. That is, after you, I don't even know how to describe it, when you broke off relations with this man who showed domestic violence, with your stepfather, when he disappeared from the family, as I understand it, or he was imprisoned, I don't know. You had some kind of surge in all sorts of independent decisions and some kind of activity, right? L. Yes, well, yes, but rather it was probably, I don't even know how to classify it. Well, because yes, then it turns out that for 6 years you seem to live in a social cage anyway, there you don't think about how you can choose or think about something, but I don't know how it is conventionally, what jacket to wear today, so that there are no visible bruises. Anyway, this stupid childish trait - I won't show it, it lived, here. And this was the only thing in which you made (laughter) any independent decisions. Previously, you already go into some kind of, it seems to me, even aggression, in relation to those decisions in which you were limited, and you are already trying to grab everything that you couldn't take there before. Well, here I need to give credit, again, probably to my mother, but she somehow directed it all correctly, what are these, well, that is, my kind of eccentric, some kind of emotional outbursts, I don't know, or what - radical decisions, like, I don't know, smoking the same thing, were not met with aggression on her part, which would make me even more willing to do something against. She manipulated it all so much that you were like, so what. And that's all there is to it, and that's okay. Yes, well, thank you, okay. Fine. Fine. Well, then, but I remember, yes, she gave... Well, the start date, oh, now, as if I didn't tear off my charger. I'll put it in. But before the start of, yes, my first year, it turns out that the first year and everything negative fell away from me.
M. Are there some things that you prefer to hide from your parents, maybe you don't tell them from everyone? You mentioned tattoos, but that was quite a long time ago. L. Yes, well, what can I say, now I wouldn't hide it, now I would come proudly (npt), to my mother and say - here it is, new. I just don't want to. I guess sometimes I can hide some of my emotional anxieties from my mother, because in general I'm such an emotional person, I can be sad, or just emotional... The only thing is, I try really hard, but I never succeed straight hide it from my mother, when I catch a cold there, or something like that, in short, it happens, I know that she will be nervous, and I don't like it so much, and I'm always trying to do something, somehow - yes, everything is fine , Fine. (laughter) These are things, yes, I try not to tell my parents, because they will worry, well, that is, I don't know, ordinary ARVI, but mom already - oh God, you live there alone, who will give you tea? do? Now, well, that is, these are not some kind of terrible things, I just (npt) don't want (npt) only dad. No one will lose anything from this, I just don't know, they will save 1000 nerve cells by doing this (npt). Without thinking about the tea I made for myself or not.

3.6 Table 6. Self-care practices (goals)

Citations
Yes, it turns out that after that I trained for three more years, I also went to different competitions, in different cities, I already understood, well, since I refused that chance, that chance, a second one most likely won't come to me, but I've already trained, let's say So, semi-professionally, I played for our academy, CSKA, but I (.) didn't dream of going there to some Wimbledon and so on. And one day, when I went to a competition in Taganrog, I was fighting for first place, the last one turned out, the last game was for first place, and I fell on my back, so. I injured my back, then I couldn't walk at all for a week, I was thinking that it was a fracture, or that it was a bruise. They took a picture and said that it was just a spinal bruise, well, it would go away soon, but after that I already quit tennis, because even after rehabilitation there, about six months, when I returned to tennis, the indicators were completely different and, accordingly, , guys who were plus or minus my age, they continued to increase the pace while I was rehabilitating there and so on. Well, basically, I was, roughly speaking, no longer needed, even in this, at this level of tennis, and I decided to leave.
Yeah, health. Until I was 17 years old, well, I was an athlete, and so on, and so for a very long time, well, until I was 17 years old, I always told myself that there is no smoking, no drinking, I will never be there, it's not for me, especially for me Dad smokes there, I think no. But then, at the age of 17, after (.) the death of my friend, my second, I started smoking. I still smoke to this day, I reacted at first... I still have thoughts, why did I do it? Maybe it's worth quitting? But then I think that, well, for now this, for example, brings me pleasure and some kind of outlet, in its own way, so I think for now I'll make a choice in this direction, and while it develops in this way, well, I'll drink there, I don't know, there's a glass of wine on holidays, roughly speaking. That is, not a fan. The choice to start smoking was conscious; I purposefully wanted to try smoking and start smoking. That is, I don't know how to describe it, one day I think - I want and will smoke, and that's it. And I kind of tried it, and that's what I wanted. M. It worked. M. Yes. (laughter) That's how it all happened. Therefore, the choice was also probably easy, I probably don't really regret this choice, well, although sometimes, perhaps some thoughts slip through there again, but in terms of complexity this choice was, I also think there were about three or four, probably maybe, something like that.
M. Yes, I think I'll quit, to be honest, now I'm already trying cigarettes, I don't smoke, I've switched to alternative, let's say, methods of obtaining nicotine, like IQOS, well, for example, something like that, it's still unknown of course, which is less harmful, but at least warms the soul, which they say may not be so harmful. And I think yes, and I'm gradually starting to come to the point of quitting smoking, completely, because I'm starting to realize that very often I smoke there, when, for example, I'm either bored or need to occupy myself with something there, any stressful situations, and so on. And when this is not such a strong need, then it is quite possible that this can be easily abandoned. And therefore, I think soon (.) I will quit. Here. Anything else to say on this topic? Or (.) can we move on?
M. How do you like it, how, I don't know... M. Well, I decided for health reasons, I think it's necessary in principle, all my life I was an athlete, a tennis player, I tried a lot of sports after tennis, then I tried again, football, volleyball. .. M. As far as I remember, swimming, you said? M. Basketball, swimming, water polo, billiards, if you can call it a sport, I tried a lot of things, and when the time came for college, there was much less time for such activities, but I decided that at least just go to the gym there for I need some kind of general health, some kind of physical development, so I went to the gym, probably already about... (..) Well, I've been going for about a

year now, (..) every week three times. I also made this decision quite easily, since I, in principle, connected my whole life with sports, some kind of unnecessary activity in my life, on the contrary, it was only a plus for me, my desire for this was there, therefore, too, the choice to go there was rude speaking, go to the gym, don't go, one, probably in terms of difficulty. Or something like that. Well, I probably can't say more about my health (..), well, I think there's nothing to highlight.
As a result, I smoked from the second to the fourth grade, that is, I had been smoking for almost 2 years, being generally a schoolboy who had his head on the table (npt) himself because he was running. But it turned out that I had to quit for two reasons. First, and most importantly, health problems began. I'm sitting there, this is all for me, I've played enough, I won't.
And in the end, the actions reached the point where the teachers all scolded me for a very long time, and were about to label me as, well, like, not a street child, but whatever it is, well, in short, register as a child. Here. But there it turned out to be somehow simple, I was simply put on the internal school register. And they said that if something happens again, then we will have the same horror. A little time passed, a week later the fight started again and then they started beating me, probably about seven people beat me, on the street, it was in winter. But at some point, I kind of just lay there angry, I don't know, something confused me, I abruptly shoved everyone who was standing, fought off with steps, quickly jumped up, kicked someone else there and caught up with one, and just I took the snow that was lying, and then there was some kind of ice and I decided, I thought that I would just wash his face with snow, it so happened that I took a piece of ice and it accidentally hit him in the temple. Nothing terrible happened there, the ice just cut the skin a little, that is, not even a cut, it was so strong. But in the end, I was still scared too, that's it, I won't go to school anymore. Let's go to some doctor and say that blah blah blah, let's check the child, I'm there, I don't remember exactly what happened. Well, in short, the doctors there determined that there was some kind of prolonged depression, they prescribed the child, well, fifth grade, antidepressants, and they forced him to sit on antidepressants. I spent a long time on them, on the course, I don't really know, me, well, not systematically, but at times, my mother seemed to say to them - let's continue to drink this. And these were, I really don't remember, either some very mild tranquilizers, or antidepressants, I remember... They were probably called something, I've already forgotten. But the point was that my studies failed because of this, I just stood at the blackboard like a vegetable. I didn't have a single thought in my head and the teachers often looked sideways at me, and to themselves, that is, like this drug addict, to the narcologist... What's going on there. Carefully. I'm sorry, the girl dropped something. Here. We went to a narcologist, where he told me that, buddy, everything is fine, they showed him a prescription for these antidepressants, and for a long time they refused. But somewhere around the eighth grade, I realized that there was a huge difference in interaction between me and my classmates, that is, more and more of the time that I sat at my desk, I simply watched how my classmates' adolescence was passing. And at some point, like in some "Rocky," I stood up in my room and said - I won't drink, that's it. I refused. And somewhere from the eighth to the ninth and eleventh grades, I began a time of real rehabilitation, when I, well, there my first interactions began, just like those who were more mature. Here. There's a story with friends, one there, others, the fourth, it still didn't work out with the girls, that's it. But somewhere around the tenth or eleventh grade, I was already completely rehabilitated, both as if just on my own, and in the eyes of my peers, who saw me both in the sixth and seventh grade. And we kind of fell into the same sort of path, that is, the same groups, and on our own we seemed to communicate normally. And this, this dynasty that I carried with me from the bad events that were covered by my pills, somehow left. Then it so happened that even by my first year it all continued; I began to experience a simply stormy, simply huge, stormy life as a freshman. And I slept, it was so that I slept at night every other night, one night. That is, I worked in the morning, afternoon and evening on projects at the university, at night we went to parties, the next day I still worked in the

morning, afternoon and evening, and at night I slept. There were nights when I didn't sleep, not because there were parties, but because I didn't have time to complete projects by the deadline, and I also had to work at night, because, of course, there were some situations, they just calculated it wrong , one of the teammates did not fulfill his duties there, and so on. Here. And I, as the leader of the project, just needed it to be completed. No matter what a person does, I will try for him now. Here. And my classmates, whom I later met, joked that I was speedrunning my life. Speedrun, this is true, just in case, for those who will decipher this later, this means accelerated, accelerated passage. That is, among the players there are those who compete to see who can complete the game faster. This is called speedrun. I was told that I was in the speedrun of my life. That is, in terms of (..) everything that they felt there during the entire time when they studied there from the fifth to the eleventh grade, I felt literally in 3 years, and even further, what they had not yet had time to experience, I already felt it. That is, I was carried away somewhere there.
M. Ilya, very detailed, thank you. In general, I'll start with your first phrase, when you started speaking, you said that reflection is always welcome. And as if from your story, it also came up a couple of times that you have, as it were, experience communicating with different psychologists. But tell me, tell me, are you, in general, inclined to reflect on your own, or how did this experience influence you, why are you talking about yourself in such detail, analytically? I. It's like two in one. Like these non-cafés. But it turned out like, the story itself from the 8th grade there, when I came off these antidepressants, well, it turned out that in order to understand this, it was necessary, of course, to reflect there. I have minimal communication with a psychologist, well, except for this psychologist I just talked about, 27 years old. Because every time I went to a psychologist, it ended, well, there were one, two, three or four trips. And they told me what I already know. And most of the psychologists that I just went to said, "I don't need to go to a psychologist, I calmly delve into my own head." That is, I will scatter if I can. The only times when I actually went to a psychologist with benefit was when I went for IQ tests. This was when I went, I sat there for an hour with him, he solved the test, then yes, this was my only useful trip. And the rest, if they didn't just interfere, somehow changed life for the worse. Although it is also unclear which one, good or bad. Probably a good one after all. Then the rest were useless. Well, it probably turned out that I myself am a reflective person, I even think at the Skolkovo university the moderators told me that at times I was even hyper-reflective, let me look for meaning where there is simply none. (..) But it so happened that the element in which we learn, it implies reflection after the end of each activity, project. Necessarily. That is, we have to sort everything out, what, why, but they didn't teach it in a very good way. But when I practiced with the Skolkovo team in game technology, a prerequisite there was, as it were, reflection itself, game technology, and in detail, that is, I had to write a plan, with the tools that I used, that is, I said what I want to try on the guys there such and such, such and such a method, there is interaction between them, maybe there (npt) according to Weldon a couple of people there can move from an idea generator to an executor, and the like. And then come back and write a reflection. It didn't work out because like this, like this, like this. You need to try this, maybe something like this. So much so that I needed to read the reflections of those guys whom I sort of moderate myself. Therefore, it's like I'm reflective myself, and it so happened that I also needed to practice this intensively.
M. But I'll immediately clarify this episode, when you already felt that you, well, smoking began to affect your health, and now your parents burned you, this moment, in general, you wanted your parents to burn you in these activities, or did you not care at all? I. On the contrary, I wanted them not to burn me, (laughter) to receive this strongly, and then for them to know, in principle. Yes, it's just that my father, in my earlier time, probably around the sixth grade, had big problems with alcohol, very big ones. (..) And there it got to the point where I was just sitting, he told me - look,

cigarettes, smoke the whole pack there, so that you can get more, so that you feel that this is not the same, and don’t smoke there anymore. Well, the like. Well, basically, since I was in school, my father probably doesn’t drink anymore. That is, I don’t know how many years have passed there, maybe even 7 years for sure. (..) Yes, in general, it’s been more than that, probably almost 10 years or something, since my father doesn’t even drink anything strong. Well, similar. He didn’t even seem to imply that smoking a pack of cigarettes would just be like, well, a pleasure. Here. But no, I didn’t want my parents to burn me at all, it happened by accident, a classmate burned me, that is, she complained. Oh, I forgot to specify, so that my parents wouldn’t burn me, I sprayed cologne into my mouth. It was an old children's cologne, I don't remember, it was unlikely that it was triple aged, because it was a regular children's cologne. But it was definitely alcohol-based, and I even remember it was “Pirates of the Caribbean”, then these colognes were also coming out. And I just poured cologne into my mouth. Here. It was terrible, but what can you do, you had to make some sacrifices.
About health, with health... (..) The story is that, let’s say, as soon as I received normal money, I finally went to have my teeth treated. I had a big problem with my teeth and I passed, then I took a genetic test to check for some congenital diseases and discovered several congenital diseases. Here I would not say that I was unlucky, on the contrary, it was good, I saw there and saw some predispositions in terms of hormonal ones too. I went to the endocrinologist and there they also developed, drank some zinc and so on. Here. Well, plus I had a dental operation that didn’t go very well, they pulled out a tooth, it turned out to be the sixth, chewable one, and he had a cyst on the root, and the cyst ate a layer into the sinus. And now between, well, where the tooth was, I have a hole in my sinus. That is, such a good message, air can go there, back and forth from the sinus to the mouth, water can go into it, so. And this, in principle, can only be healed with an implant. This, well, with health, it means that it was necessary, well, it was necessary, well, most activities were prohibited. But now, for example, it’s literally been a week here, and after work I’m going to go, well, recover in the gym. Because now I couldn’t go to the gym for a while.
M. And now there are those moments on which you consult with your parents? I. (sigh) (h) (...) Well, probably for health reasons, I always consult with my mother, because she is a nurse. Here. That is, if I suddenly start to get sick there somehow, in a way that I have never treated myself before, that is, if I start some kind of ARVI or something there, I can basically cope with it myself. Here. And when it comes to some symptoms that I haven’t had before, I call my mother and talk, if they don’t help, then I go straight to the doctor. Here. (..)
M. I hear some kind of conflict between what you think and how you act. And how is it generally given to you, is it easy to make a choice in such a situation or not? That is, why do the scales still start to tip in one direction every time? M. (sigh) It's hard to say. Probably because... (..) How to say... Well, again, probably there is a point in psychology that people often treat others the way they want to be treated. And among other things, I give people, other people, a chance because I would like to be given a chance one day if I stumbled. This is exactly why I give people there some kind of warmth and so on, that is, everything, all these moments, because I myself lack it. That is, what happens is, let’s say, the opposite situation. That is, I give what I lack. Here. M. But this is generally interesting. How did you come to such an understanding, to such an experience? And to your own behavior? M. I unconsciously stumbled upon all sorts of psychological things a lot. (NPT) I unknowingly came across all sorts of psychological things, I don’t know, because you scroll through your VK feed and there, for example, there are some posts about psychology, something else, in the same TikTok you come across a lot of all sorts of psychological analyzes, oh behavioral types of a person and so on. So it all stuck to me like that and, in principle, some kind of awareness came. And that includes still coming. Here. And so... M. So, that is, you are not a naive respondent, right? And pumped up.

M. As if your experience of depression was for you also such a marker of where my friends are and what choices can I make in relation to them? And as if you, despite the fact that you are used to giving people a second chance, decided that you will not give them this chance? M. Well, I just, again, it seems to me that I have some kind of innate probably psychological things, well, that is, I can read people to some extent, I understand how they behave, how they will behave throughout to lead for a certain time, that is, it seems to me that I have some kind of psychological habits in terms of reading a person and so on. And all this. Understanding other people, including yourself. And based on this, I probably, well, probably don’t give these people a chance. That is, I understand that they will not change.
M. Well, yes, it seems that you hear yourself very well and understand what you need and what your needs are. Tell me, with your activities, what you called a hobby, did you also listen to yourself or perhaps it was imposed by your parents? M. Regarding sports, I probably always liked to do something like that, to feel some kind of power over my body, that is, when you grow, including over yourself. Including remembering, as they say, (laughter) the classics of fight club, development through self-destruction, that’s also a wonderful thing. This is also wrong, but I probably came to this too.
M. Well, great. That is, in addition to your profession, you also have a hobby that gives you pleasure. And you seem to have mentioned something else in your hobbies, music, right? M. Yes. Yes. Here. Well, it’s more... There is some kind of story in me that I want to learn how to play well, and so on, but I’m probably too lazy for this, and even more so, it seems to me that I don’t have good potential for this . That is, I can play some banal things there, play something on chords, sort of a little strumming, that is, I can play, and... Well, it’s not like I studied there, that is, it’s more by instinct it sort of went, I liked it there, how I typed some kind of melody, I started to continue there. Here I am... M. How old were you when you started playing music? M. (sigh) (..) Oh, probably 18-19 somewhere. In this regard. M. And it was also your choice, right? Or... M. Yes, yes, it was my choice, completely. It’s just that at one point I realized that... Well, again, I’m probably a depressive psychotype, I don’t remember, if that’s what it’s called, so. And I bought myself a musical instrument simply because sometimes I’m in such a state that I just want to sit in the dark and strum something sad to myself. This somehow makes it easier. M. But it’s cool that you have such a resource that you know what to do in such conditions. M. Well, before, I used to, as they say, beat myself up with sad music, but still doing this, as they say, this is probably my favorite self-flagellation and so on. Well, it’s probably not self-flagellation, it’s probably just some kind of self-harming. Here. First I bought a ukulele, realized that it didn’t sound sad enough, and bought a guitar. (laughter) Here. There's more bass, more sadness can be created, let's say. Here. Well, sort of in this format. M. Yes, it’s interesting, that is, you can take this sadness out from the outside and create it yourself. That's great too. M. Yes. (laughter) M. No, it’s really great that sadness comes out of you and is expressed in creativity. This is great. M. Well, including... Oh, yes, by the way, I remembered another hobby, you said, expressing yourself in creativity, also not so long ago, a year ago, I think, I started writing a poem, here. Also, as they say, sadness had a very strong influence on this, along with depression, so. Because there is not a single funny poem that (laughter) I wrote. Because, it seems to me that the strongest emotion is the worst emotion. And based on this, here comes, well, let’s say, what I do.
M. The choice of a doctor, the choice to seek medical help or not. M. (..) (h) Probably (..) (laughter) two or three. Here. Because, in principle, I practically don’t get sick, I’m a follower of a very strange method, the one that does everything on its own, but there is such a joke that a man goes to the doctor only when a piece of a spear in his back prevents him from sleeping. On the back. Here. Therefore... I probably have a lot of them, by the way, yes, it will probably be

important to record that I have a lot of things taken from my personal stereotypes that I have formed of what a man should be. A lot of things come from this too, that is, I literally have a list of men there, too, to be like this, like this, like that. That is, well, I understand that stereotypes about men, in principle, stereotypes are a very disastrous story, but I myself, here is my list, (laughter) I should be like this. That's how it is in this regard. This also affects a lot of things in my life, so... Yes, regarding health, in principle I probably don't really value my health, so it's easy for me to make decisions in this regard. Here. I kind of understand that I do sports there and so on, all the things, but at the same time I can drink there calmly, well, I try to know my limits and so on. It's as if I understand that if I drink a lot, then (laughter) I will die in one moment.
M. (laughter) Yes. Here. Therefore, I probably don't particularly value my health, and making decisions here is quite simple for me, I still try to control my health and so on, in the sense that, well, I try to see something in myself if something hurts me and so on, but otherwise, it's as if I've never had such a thing that I have to go straight to the doctor, that is, it seems to me that I'm more than healthy. I recently took tests, and they told me that they could at least launch me into space.
A. Yes, I work in an IT company, well, it's an ordinary development studio, I'm a backender. M. Tell me, do you have any hobbies, interests, besides study and work? A. Yes, my scientific interests are research in the field of education, I am mainly engaged in the history of the philosophy of university education. Psychology a little, I also do origami, sports are athletics, that's it. Just like that. How long have you been doing sports? Two years. Well, intermittently, I did a lot of sports as a child, well, I consciously came to this (npt).
Health. Health is the area that suffers greatly in my life, I'm sick right now, right now. And I recently took a Covid test there, yesterday, and I'm waiting to see whether it will happen or not, but it seems that it won't, I already feel fine. But, damn it, I haven't been doing much about my health lately, but I just support it with sports and that's it. But I would like to undergo some kind of examination, the fact is that the right side of my head has been hurting all my life, and it's such a noise, it's just as if, I can describe this pain as a noise. I've already gotten used to it, because I know that damn it, this never happens, once in the tenth grade I did an examination that did not give any intelligible results, they told me that I was healthy, I'm into it I don't really believe it. (laughter) Here. Still, something is wrong. Otherwise, health...
Health - ten, it's very difficult to make a choice, I have some kind of masculine fear, I don't know, or in general it's a universal fear of going somewhere, or arranging an appointment with a doctor, going there, I don't know, to the dentist once every six months. Well, in general, it's just some ten, and I don't know what's stopping me.
A. Oh, you know, this is very interesting, I had no thoughts. Well, that is, if we are talking about some kind of rational reflection, and I mean perspective reflection, that is, when we assess risks, when we, we don't know, analyze a situation that could happen, it in general (xxxx) (laughter) there was none. Well, that is, it's purely based on some kind of emotion, some kind of euphoria, it's probably possible, I didn't use psychoactive substances very much, I smoked marijuana twice, but it seems to me that this is something similar to what people experience under psychoactive substances, such an absolute frenzy and absolute lack of self-control. Here.
Next, health. (..) Yeah, health, health. Here, you know, there is a rather difficult aspect, because for probably several years my health has fluctuated, (laughter) let's say, like the exchange rate of the ruble on the stock exchange. Probably until 9-10 grade, I was quite (.) a chubby boy. And I probably made the choice in favor of a healthy lifestyle on the basis of some, let's say, personal oppression and personal motivations. But for the most part this was due to teenage conflicts. And, accordingly... (..) Any problems. M. Did someone offend you? I didn't quite understand about oppression. A. Let's just say, (..) at some point it began to seem to me that at school, on the part of friends, on the part of teachers, at some sharp moment the attitude towards me changed. That is,

they stopped noticing me and (.) paying any attention to me. Although I didn't seem to do anything wrong. Nevertheless, I didn’t really understand what happened, but at that moment it was probably quite difficult for me to communicate with people, because I didn’t understand what this attitude was connected with. That is, if I had a teacher with whom I was in close contact, and (.) with whom, in principle, I was conducting some kind of project activity, then at that moment it happened, I don’t know how some day came and as if at the snap of a finger What happened was that this person’s entire attention switched to another audience. And essentially, you know, it's like a child who has been sharply deprived of attention. You want it, but they don’t give it to you. And I probably began at that moment to look for disadvantages in myself and in my appearance, in my health. I began to think that this was probably connected with some characteristics of my body or characteristics of my thoughts. And then I probably started soul-searching. (.) Therefore, the choice in favor of health, it was probably given to me, let’s say, through a battle, due to some experiences, through an attempt to prove to myself and many others that everything could be different, that I can do something... something else. Therefore, in terms of severity, probably (..) well, let it be a ten. At work... M. So you connected this deterioration in your relationship with the fact that you somehow gained weight, or somehow didn’t look the way it seemed right, right? Because you were talking about the love for sweets, and about being chubby, you said? Or did I misunderstand you? A. Well, for the most part, I’ve been pretty chubby since childhood, and I probably stayed that way until the 9th grade. Therefore, purely physically, I didn’t change much, due to weight gain or loss. There was simply, let’s say, one consistency of the body, which was maintained as it grew. Therefore, I don’t know, I probably connected this not with an increase or change in weight, but with the fact that at some point some skills and shortcomings that people did not want to take into account simply became more noticeable. M. Did you say something to you, did you somehow become yourself, did you say about choosing a healthy lifestyle? Something like this. A. Yes, I decided that... The Unified State Exam had just ended... the Unified State Exam, in the 9th grade, I thought that since such a situation was happening, I needed to rethink myself, I needed to somehow start taking care of myself, to show myself, well, to change something in myself, not only mentally, but also physically, so I probably made the choice in favor of a healthy lifestyle, in terms of athletics. That is, I got up at about 5 in the morning, slammed the doors, everyone was scared of what was happening so early in the morning, and I just left the house and started running at the stadium, ran to some park and spent my time there.
really don’t like this, so I’m trying to gradually return to my usual way of life. And somehow connect sports to your activities. But for the most part, probably due to my (..) fear of people (xxx), some kind of tightness, I don’t always succeed. That is, I cannot, relatively speaking, sit in a hostel, do something, get up and start doing push-ups there. Or, I don’t know, stand in the plank. Because, (.) you know, this is an internal experience when you look stupid in someone’s eyes...
M. Well, yes, I understand. A. Yes, for the most part that’s why I got up early in the morning and ran away earlier, to get away from prying eyes. M. Well, yes, very clear. A. Now, well, basically, what you can do is go out late in the evening, we have a small park, a student park, and do a few laps of running there if you have free time. If not, then I try to diversify my diet and make it, let’s say, less high in calories.
And so, if your hobby is sports, crossfit, cycling, snowboarding and generally active recreation.
M. I see. How do you help your friends? Well, in fact, were there any similar interesting situations? A. I don’t remember them so often, but most often these are situations related to a psychological and moral point of view, because they fall into some kind of depression, something bad happened to them, I try to support them, I remember sitting at night , talked to them, tried to support them, although he himself took the test in the morning. It was so. Here.

A. (..) Well, in general, in order to get straight on where to go, they told me banal things about don't drink, don't smoke, love your mother and everything like that. But the smaller parts, somehow I don't even remember this, but it's already on my mind, that is, not to forget our neighbors, those who are nearby, to help them not to forget. Here. These are the things. And if we talk specifically about independence, then somehow... Well, I don't even remember, they just said that you will grow up, you will be independent, you will do everything for yourself, cook soups and all that. Here. Well, they just taught me and I remembered it, I put it into practice and then I cook borscht for myself now. Here. M. Is this specifically about borscht, about this kind of food, or about some other things too? A. Well, besides this, yes, for example, hammering the same nail into the wall is understandable, my father taught me this too, it was also not without bruised fingers. Here. So... Besides this there is a lot of other things.
T. And I remember myself probably from the age of one... Well, probably from the age of six, from five, somewhere like that, directly consciously, so to speak. Well, as a child, I initially wanted to become... (..) To become a policeman, that is, at that time, a policeman, and, in principle, my parents did the same, if we talk about independence, that is, they always signed me up somewhere, to some clubs, some sections, that is, since childhood I was kind of fond of sports, that is, I was such an activist, that is, both at school and in some extracurricular activities, social events, that is, always for the most part, they attracted me, too, and (..) it seems so, and some leadership qualities were probably emerging, so this is where the roots probably go, maybe some of my independence in making some decisions. That is, as I have already said, the sphere, so to speak, of my activity is quite wide, that is, starting from the public... That is, this has been going on since childhood, that is, now I, let's say at work, am the chairman of the council of young scientists specialists, these leadership qualities that have been going on since childhood also manifested themselves. And I finished school, that is, until the eleventh grade, passed the exam, and entered the university. At the university, of course, it's also a little less, since after school, for some reason, I began to devote more... More time to studying, not in the periphery, but specifically to studying, and therefore, a little less, I had classes that were, so to speak, excellent from studies. But still, no matter how I gave it up, I also took part in all sporting events and social events. So I studied and studied, and naturally, after that, after that, I went to work. And at work, from the first, as they say, days, that is, he showed himself as a fairly active person, went out with his colleagues somewhere, that is, he was always, so to speak, easy-going. And, that is, after that the management noticed and, as they say, I am still, so to speak, in plain sight. That is, just like the leadership, as it was before, like at the university, like at school, and now, let's say, the strategy can be action, that is, just independence, leadership qualities, and so on, then are present at the moment. Here. In principle, I think, more or less answered. (laughter)
T. Well, I have this character trait: sometimes I follow the majority. That is, in order, as they say, to go against the flow, namely, my closest circle. That is, my environment was quite like this, just sports, that is, we were engaged in dancing, and naturally this was vocals. I was, I looked at other people, I looked at how they were, whether they were achieving success in this regard, and then I tried it on myself, whether I was even interested in the same activities that were popular at that time, and I already made a decision. And if, so to speak, in percentage terms, they probably have their own opinion and people's opinion, well, in general, the opinion of the current, the opinion of society, so to speak, is probably about 50/50. Well, maybe 60/40, in my favor. In this plan.
M. In general, well, now it turns out that you are discussing with someone some things that may be difficult for you, well, be it some psychologist, I don't know, a close person? T. Well, yes, there were moments, that is, when I visited a psychologist, that is, in principle, there were moments when I was just put in a leadership position, and I, having worked for only 2 years, yes, about two years , one and a half, in general at work, that is, I was put in a leadership position, naturally, there

was stress. In general, there was a lot of stress and I needed some kind of psychological help, (..) it turns out to be qualified. Yes, I also consulted a psychologist. And in principle, this gave its micro-fruits, that is, well, perhaps, including the fact that I have already gotten used to it, that is, this so-called adaptation period has passed, and now I am more or less in a normal, so to speak, psychological rut and everything is fine.
M. And if we talk about health, then when you make decisions in this area, what helps you do this, for example, a consultation with a doctor or how do you even choose a dentist? T. Well, it turns out that in this regard I'm probably suspicious about my health, that is, if some kind of sore pops up, I start there, well, not exactly panicking, but it's faster, faster to take some action to remove this pain. Just with teeth, and everyone knows that this is a rather expensive procedure, namely dental treatment. And therefore, finances did not quite allow, or rather allowed, but there were other, well, one might say, demanding, that is, aspects that required an urgent investment of money. And that's why I put off just about the teeth. Here. And therefore, in principle, since I have already said that I am suspicious, I quickly, quickly tried to fix all this as quickly as possible, so as not to start all this. Then don't pour in even more finances, already eliminating the consequences of all this. M. But I'm even asking more about how you understand who is a good dentist there and who is bad, about this. So, where do you get this? T. Well, here again, already through acquaintances, that is, I found out from people who treated there, and about prices, and so on. There was no such thing here that, yeah, I typed dentists there into Google, and went to the first link that came up, and immediately went there. That is, naturally, at first there was some (..) research, so to speak, on this matter, that is, I found out from friends there who was doing good work and, accordingly, I went there.
T. Well, in principle, all my successful decisions, which were made at the junctions of the most important stages in my life, were at school, that is, the choice, I don't know, not just there, I don't know, walking around the entrances, smoking and so on, have a drink there, namely, go in for sports, that is, some kind of social, some kind of personal development. At the university, that is, to enter exactly the university that you are now, well, in which you naturally studied, (..) it is precisely the choice of the field that is most promising in our country. And at work. That is, I work in a fairly large company and this, in principle, makes me happy. I don't think that somewhere in these probably most important stages for myself, in my life, I made some wrong decisions.
T. Well, yes, that is, before, I'll interrupt, I'm sorry, I just had a problem before in that sense, that I, well, when you see a cat trying to attack a mouse, that is, it first prepares itself there, sits for a long time, waits, and at some point pounces. I've always had this in anticipation and preparation, just before communicating with a psychologist and so on, that is, I've always had this for a very long time, that is, I'm trying to calculate every little thing that is impossible to calculate, and sometimes just you miss your goals. This is exactly what is needed, it seems to me, we simply mean to wait, but not to wait out in general, roughly speaking. That is, to evaluate yes, more or less seriously, (npt) you act as you see fit. Here. Something like this.
I. So health... What elections, elections, elections, elections, elections, well, it seems to me that with my health pah-pah-pah, everything is fine, I try not to get sick, (laughter) here. It seems to me, well, if we take health in general, well, my mother just had a stroke there. I had to make certain choices there, I don't know, about apartments, about inheritance, and so on and so forth. This all also falls on me, in any case, so for me, well, it was hard for me, it's still hard for me to resolve all these issues, because in many areas, with many of these things, I'm meeting for the first time, there the insurance company, the hospital, I don't know, they have to process certain documents, it's incredibly difficult for me. As a child, my health was bad. As a child, after childhood bronchitis, I had very severe bronchial asthma, I was sick for a very long time. I rarely, I didn't go to kindergarten at all, and I went to school, but I was sick very often, of course. (..) It was very

difficult for me as a child, so it seems to me that I would rate my health as exactly this, I would rate it as an eight
when everything seems to be bad, but I always keep the thought that life, this is also such a clichéd concept, that life is like a zebra, that is, there is a black stripe, there is a white stripe. And I began to notice it too, it seems to me that maybe this is self-hypnosis, maybe, I don’t know, it’s more like you, as a psychologist, will say. (laughter) I also began to notice that yes, there are some lows in life, but there are always ups and downs in life. And... (..) And the same goes for good and bad things, for me too, that is, I have neutrality here, but in life, yes, there are some good and bad moments, just even bad ones moments, I never call them bad, I just think that this means now is the time, some kind of quiet, calm life, or doing nothing, or I don’t know, it means that life is telling you - rest there, devote time , I don’t know, some family issues, don’t engage in public affairs. There and then next week you will have five new acquaintances there and ten new projects will arrive. And this is how it usually happens, I don’t know, I was there (..) about two weeks ago, I think somehow, and when autumn began, probably at the beginning of September, I was just moping, moping, I think - nothing It turns out that it still doesn’t seem to work and it seems like my colleagues and friends also have problems, somehow they need to be helped, no one helps them, and I can’t help them either. And now, October has arrived and there are just so many different projects and you’re thinking, how can I get rid of them as quickly as possible, from all these projects, because somehow they all just landed on your head, and you don’t understand what to do with them? . (laughter)
M1. What was prohibited, damn it, no such thing actually happened. That is, I don’t know, well, it was forbidden to steal, yes, to do something else, but no, even some kind of morality, alcohol and cigarettes, we just don’t seem to discuss these topics, in general, there was no such thing as it’s impossible, smoking is harmful, I don’t seem to know, through conversations maybe, through some other information, I realized that it’s kind of harmful and I didn’t do it, about cigarettes, never in my life, that alcohol is just something to try, understand that that's it, that's all. Well. We just didn’t talk about this topic, that’s all. It’s like I didn’t study, I kind of understood that they wouldn’t be very happy if I smoked and so on, I kind of thought, why do I need this? Well, plus, I'm very frugal, and I understand that cigarettes are a huge waste of money, and so is alcohol, so why bother with it? Oh, I’ll give you an example, here’s one pack of incomprehensible cigarettes, then let’s say you can buy a certain amount of sweets with this money. I'm like, candy, it's cool. That is, it’s like, well, everything is measured by equivalents, even this, as I was taught financial literacy, that - mom, mom, I want Kinder, I want Kinder. Mom tells me that look, it costs so many rubles, but you can go to the store and buy so many candies by weight. I'm like, wow, let's have better candy. And I sort of understood that.
M1. Oh, I’ll start now from the earliest, it’s just that my mother has such a perception of life that she was against kindergarten, due to negative experiences in childhood, that she remembers by her example that teachers are very like that, not so much, and may not monitor the health and so on of the child. And she always sent me to some private development centers, and if you take me from a very early age, there are speech therapists with me, reading, something else, that is, in addition to the fact that my mother worked with me, that she is like me in fact, she taught me everything, before that at school, I went to such educational programs, and when I became more conscious, as an adult, probably at 4-5 years old or something, well, at 4-5 probably, yes, at 5 somewhere, I went to the children's center, combined with a fitness center for adults, and there were all sorts of sections like judo, fencing, and all sorts of other things, that is, I kind of went, it just sounds cool, interesting, some kind of dancing, gymnastics, acrobatics, absolutely went different. Accordingly, later, when I became even older, my range of interests was outlined, these are creativity and sports. Here. As a sport I went to judo most of my life, then 2 years to boxing, 2 years to karate. And I

probably practiced judo for 10 years, or 12, I don't remember. I graduated from music school with honors, so in terms of interests, you can say, well, little by little, let's say take a swimming pool, creativity is a guitar, some kind of drawing, singing in different sections, that is, I actually did drawing in many different sections. I won't say that it was some kind of cool level, but just purely for myself, to communicate, to spend time, it was. M. And at school, it turns out that you also managed to study very well, in addition to these sections? M1. Yes, I can say that I, this is not my assessment, this is the assessment of others, that I am smart, and I had an interesting situation, that I understood that I could easily be an excellent student, I am not a fool, I can prepare, I can do everything, but I was just kind of lazy to spend so much time, and this is probably not very good, but I never had problems at school, any difficult subjects, the most difficult subject for me is chemistry. Somehow, out of interest, I just took it and decided, I'd better learn the topic, and then I didn't study it anymore, this topic, because it wasn't interesting to me, that is, I understood that I was doing things that were more useful for myself . Accordingly, I had no problems at school; if you look at the boys, I was among the smartest, that is, in terms of grades, and everything was fine. And this despite the fact that my gymnasium is one of the best schools in my city, where studying is quite difficult.
That is, my schedule is like this: at school I communicate with them, after school I go to the music room, there I communicate with these people, and from the music room, let's say I go to sports, there I communicate with these people. And this is how it went cyclically for me. That is, I communicated with many, but I won't say that all of them were my very good friends, they just communicated well, well, in some respects they were friends, naturally, in each section I had one or two people with whom I was most communicated, I think these are adequate things. Well, something like this.
M. But about the karate trainer, tell us how he helped you develop independence, since you speak about him so warmly and in detail. M1. Yes, I speak very warmly about him, about the coach, because, well, this overlaps with the fact that I spent a lot of time in training, a lot of time, from the tenth grade I did karate, it got so bad that I had, let's say Monday, Wednesday, Friday, two workouts a day, 2 hours each, and the remaining time, let's say one. Each training session lasts somewhere for 2-3 hours and I was with him all the time, like listening to how much he loved to share some stories, how he not only enlightened us, but simply talked about all sorts of life topics, I heard him talk about it, I drew some conclusions for myself, what I can do, how I can do, similar things. Here. In general, just in my opinion, even in terms of education with a child, the main thing is to talk to him stupidly. Just talk. Tell some moments, life situations, how he got out of these situations, so that the child has some kind of template example that he could use in the event of some new situation. And then impose your own, having some kind of behavior model. That is, it seems to me that it is from conversations that a certain model of behavior is formed so that the child can use it and accept it as a given, in order to develop it later. He can completely redo this pattern of behavior, in case of some extreme situation he won't have to think, but what should I do, he will remember - yeah, my dad did this, or my mom did this, you can do it like this. He will do this, and then he will think, what is best for me? He will do what is best for him. That is, just in terms of conversations, all this is being formed. That is, the coach said that in reality martial arts are very connected with personal perception, and you cannot teach martial arts if you just come, roughly speaking, once a week to training and just somehow teach the technique. He said that all the masters, they lived with the teacher and seemed to watch him, how he behaved in the natural environment. They seemed to adopt the image, likeness, and mind of the teacher, how he behaves in a normal environment, outside of training, outside of sports. And this is the difference between a coach and a teacher. I can call my coach a teacher. For example, I can't call a judo coach a teacher,

because his task was to make athletes out of us, well, he made athletes out of us, he is a professional in this matter, but there were no such life conversations. Because that was not his task. My karate coach had a different task. He talked to us a lot. Accordingly, well, somehow he is still just a teacher by training, I'll tell you what, at the university, so he knows what to talk about and how to teach. So it was very interesting and educational. And it seems to me that it was karate that revealed a certain personality in me, in terms of absence, removed some edges, that I can live calmly, socially, not be afraid to meet someone, do something else, because I don't know, how it happened, but I'm sure it was after karate, because martial arts associated with a certain kind of combat, they greatly change the worldview, perception, and seem to remove some kind of framework, boundaries. You become more confident. And quite independent. You could say. Because you are not afraid to defend your point of view in some dispute, knowing, like, that if someone comes at you, you will be able to give him an answer and give him a very good answer. M1. About the karate teacher and dash teacher, I was wondering if he has an education, you said that he teaches at a university, but what does he teach? M1. He is the dean of the physical education department of our Ryazan State University. M. How did you even get into his section? M1. This is also a very interesting story, I... (..) I just somehow saw how training was going on, in karate, in a completely different place, in a completely separate place, in the Krasnodar region, when I was with my grandparents in away, I saw how the training was going on. I was so impressed by this, because at that moment I was doing boxing, and I lacked some kind of morality, some kind of cultural core, when before training everyone bowed, sat down, closed their eyes, well, not just prayed, but they just sat down as if getting ready for training. After training, a bow, ritual phrases, when there is movement and intelligence, not just a straight punch, a side kick, but some beautiful Japanese names, Japanese language, culture, kimono, that's it, equipment, I really liked it, it went in, and I I started looking for this section in my city, I found a section in my city. It was another coach there, who reported, in fact, to the head coach, whom I spoke of as a teacher, I went to him first, but then this coach said, that there was an opportunity to go to some training camp, go to another building, with this head coach, so I went and somehow it happened that the coach liked me and he decided that I would work with him in the training camp team, national team . And I began to study with him. And I spent, in about six months, actually, I actually studied for six months, I went along the path to a black belt, in fact, because I worked a lot, plowed, studied, learned new things. This is my story of meeting this karate trainer. Now we don't communicate, but I remember him very often, that is, in some conversations, and I am very glad that such a person appeared on my life's path.
Because money affects everything, without money you will not have the opportunity to live in a place where you have good health, eat well, pursue interests, that is, any hobby comes down to the issue of money. That is, I like to do martial arts, but with what money should I buy a sword, tritely, with what money should I buy equipment? That is, money decides everything in my opinion.
Well, here's an example. That is, well, really, communicate with people. Well, I'm proud that I was able to defend this Olympics, the championship, I'm proud that I have some skills, I'm proud that I found an internship, I found it myself, yes, I'm proud of that. I'm proud that I was able to achieve certain things in sports, just when I wanted to do karate myself. I'm proud of this too. That is, here.
M. Listen, but about dependence on someone, you say that you don't like to be dependent on someone, but why, that is, what do you put into this dependence? M1. Well, I had an offer from my coach to become a personal student, in Japanese traditions, as it were, that is, roughly speaking, to live with him, that is, to do what he does... As if he essentially becomes a certain , not exactly a master, but kind of dictates to you what you need to do in order to develop. Yes, I would become an athlete, but again I understand that I might not become if I had some kind of injury. That is, I did not want to put everything on this card. I didn't like this concept, that you are completely

dependent on a person, that... For some, this is good, you don’t have to think about what to do tomorrow, but for me it’s not good because I kind of control myself and do what what will I need. That is, as much as I don’t like being limited by someone. Any limitation here is not very good. That is, there you go. I like the Higher School of Economics because, by the way, there are no restrictions on studying, that is, you don’t even have to attend lectures, that is, you are, as it were, responsible for who you will be. That is, there is no such strict control, so what - oh, you need to learn. If you don't study, you'll fly out. (laughter) As if only this fact stops you and that’s all. There are no mentors there to control them, no micro-management...
Well, this is what happens in life, in general, it always happens, for example, if there was a need to get answers to some questions and close your own questions, whether for business or personal reasons, I found a good psychologist, went to a psychologist, solved the questions, closed questions. Somehow like this. Should I answer in this format? Is that normal? Is it clear now?
A. For health reasons. Well, in fact, in terms of health, what can you say, what can you say in terms of your health... Well, I’ve had problems with health for quite a long time, it was decided that health needs to be dealt with systematically, and the decision was precisely on the advice of on the advice of parents. I adhere to the following approach - once a year you need to do a complete Check-Up of your health, and regularly take tests and monitor them. And this decision was made very simply, because there was advice from my parents, accordingly, after the advice was received, I understood why this was needed, why you need to regularly track the indicators of your body, I realized how important the energy that you have is, to exist well, and there would be enough of it for what you do, what you do. Well, that’s why I regularly take care of my health, track and live happily and energetically.
M. I think that you not only check, but you said about surfing and probably, well, you look very athletic from here, at least it seems, I think that in addition to checking, you also do things, well, you said, go to the gym, right? To the hall. A. Well, yes, because I realized quite early that sport is energy, well, more precisely, at least for me, that’s it. And in general, I just can’t live without it, because... Well, no matter what your condition is, if you go to a training session, or go surfing, or go jogging, then you get a huge boost of energy, and in general I feed on this. Well, that is, sport is one of the key ways to get energy, to recharge in order to do those energy-consuming things that we do either in business or in other aspects of life. Here.
M. What brings such warmth to memories of school? In primary school? M. There was somehow more friendly communication there, with the team, but the only thing was that I was very far behind in sports. When they played football, volleyball and so on, I usually sat on the bench. M. Did you want to continue, did you want to play together? Or were there some reasons why you didn't play? M. To be honest, it’s difficult to answer, because on the one hand, it seems like you want it, but you don’t seem to want it, this. M. Was it your choice to sit and not play? M. (..) No, I think my lack of skills played (..) in this.
M. But in general, this is the period before yours, before your state now, right? M. Yes, there was also the military registration and enlistment office story. M. Tell me more about this. M. Well, I was in such a disgusting state of mind that the doctors and the military commissar said, no dear, you are somehow really bad, let us observe you in the hospital and sort of decide whether you need to go there or not. In general, I spent 7 unforgettable days in the Gannushkin Design Bureau, well, I lay there, I just lived there, like, I don’t know, in a hotel. Here. And... Well, I was declared unfit, given category “B”, I never left to serve anywhere. M. How would you characterize this period of your life? M. Well, these 7 days, it would seem, should have been quite difficult, but the only difficult thing was the fact of being there. The stay itself, in fact, gave me some kind of respite or something, because it was even harder at home then. Here. M. So it was some kind of escape?

From what was going on at home? M. Escape, escape. M. What happened, how did you react later when you were declared unfit? M. Well, it's a relief, because if I left, I would feel even worse.
M. Yes, then you can move on to the next point. M. Choice of lifestyle and leisure. Well, this is where it gets more interesting, by the way, because I decided to change my lifestyle. He was like this, he is like this now, more sedentary, not very active, I found myself in such a rather interesting situation that after my previous job and previous company, all my interests simply died, because all my time was taken up by this sweatshop work and gatherings there with drinking there and so on. The rest just somehow fell away. And now I think what is interesting to me, what is not interesting to me, I restored my physical activity to a minimum there, in the future I will... In the future I will return to sports. I've even already decided what exactly it will be, what it will be.
M. And what will it be? M. I want to go to boxing. It's very necessary, in fact. M. Why boxing? M. Well, because it is he who has a great psychological influence on people. Well, according to my observations. This is both confidence and the ability to stand up for yourself, which is very important to me.
M. Completely, I don't remember the last time I drank. In fact, with addictions everything is very interesting, I can, as if I don't want to and I don't. I also tried smoking then, but I didn't understand this joke at all, to be honest, I just started and ended. Moreover, alcohol also makes me feel bad. Because alcohol is such a depressant or something. That I won't go back there at all. But I didn't have time to develop alcoholism, I... I don't have that.
This means health is seven out of ten, it's more likely just a dislike for specific healthcare institutions. Otherwise, I can calmly go to the specialist I need.
V. No, there was no such question, but my mother tried to compensate for this so that I would somehow interact with society, so I went to an English language club, to drawing, in general, they took me to all sorts of different events where there were other children, Mom often went out with her friends who had children, so it cannot be said that I was deprived of communication with children, just food, catering, my mother did all this at home, that's it.
And I started, that's how I played computer games for 16 hours, in the same way, for 16 hours, I read literature and classics of literature. And of course I tried to write poetry. Here. And then, in all the blogs that I kept on games, I began to write about the fact that games are, of course, cool, (npt) but it is necessary, well, the trick is that they greatly, they very much limit the space in which you're cool. And I really love being cool, still do. Here. And how could I, of course, be the top one in the game, but if I go for a walk around Moscow, it will give me little. Here. I wanted to develop something with more generally accepted values, in some more generally accepted values. Well, that is, for example, knowledge of foreign languages, erudition, career prospects, and so on. So, at the age of 16, at 16, yes, at 16 and a half, in short, somewhere there, at the end of 11th grade, I realized this and began to work in this direction.
V. Yes. Yes, I wouldn't even call it stress. I kind of liked this state of competition again, another one, here. Well, so did the entrance exams, and I was like, oh, I entered Moscow State University without a basic philosophical education, that's fine, now I'm also a translator. In general, this energizes me. So, in short, I always loved this state of competition, and as a child, when you are not competing in the professional market, this is probably one of the only niches where you can compete, these are games, well, such games, board, computer, or sports games. Here, by the way, I would actually dwell here in a little more detail, because I love, since I love this whole business, I did both, and the third, that is, I always played board games with pleasure, a la cards and sports games. I say, I played chess as a child and table tennis. Here. But... But just at the age of about 12 years old, that is, at 11, I started playing computer games, and at the age of 12, I left sports and stayed only in computer games. Here. I wonder why this happened, well, I think I don't know. (laughter)

In general, in principle, I don’t like, I don’t think that anyone knows better than me, especially in pedagogy, in matters of education, because I have a psychologist’s education, pedagogical, I know perfectly well that no one knows my educational does not diagnose needs. Moreover, no one has as much knowledge as I do about which method of presenting material is suitable for me, so this education system does not give me a choice, well, let’s say this happens often, the curriculum says that there are many disciplines by choice, but in fact there is nothing there anywhere, plus the budget needs to be distributed correctly, in the end there is one, well, at most two, you have a choice. In general, I don’t like this all the time, I like it when I build this educational route for myself. M. Well, that is, studying at the psychology department helped you understand yourself? Q. I don’t think it was specifically my training at the psychology department, although... I think that it structured my thinking, of course, certainly my thinking and worldview. In principle, psychology, pedagogy, and philosophy, which I studied, help me understand myself better.
V. Yes, well, accordingly, I created my own group, and spent the whole last year working with this group. And now I want to try again, now I really don’t know, but I still hope to somehow go to study in France, so here I have the same problem, that I’m not a psychologist, that I’m a psychologist-educator, and France is more strict system in terms of changing qualifications, so I need a year at a Russian university. Here. Therefore, I want to study at a Russian university for a year at the Russian State University for the Humanities, and then try to transfer to... Well, there is the city of Caen and there is training for translators in three languages - Russian, English, French, and so on.
V. Well, yes, with health, well, I’m generally not the healthiest right now, I have allergies, and a bunch of other diseases, but our medical system, it’s usually in no hurry to help, and usually, until you get completely ill , somehow they’re just trying to tell you, like, go drink oak bark there and everything will be fine with you. In general, my health taught me that no one except me will worry about my health. Therefore, you need to show maximum will to get a referral, to get the tests that are needed to get a referral to a research institute, and so on. And there are elections, that is, here, too, it’s probably difficult to implement, that is, to withstand the doctor’s pressure, because the doctor has authority, that is, like, are you going to teach me, puppy? You (npt) like me, did not undergo residency training. But, you need to show pressure, I then wrote the first complaint in my life to healthcare, to the Ministry of Health, here. This is just such a space of struggle for me, health, so I can say that this is... M
A. This means that what happened, well, I had certain health problems, (..) they are related to reproductive function. She was questionable to me. Accordingly, I needed to get a referral for surgery, this is why the health sector is such an important thing. So, I needed to get a referral for surgery and it’s very difficult to do, it was, well, this whole story began in 2019, that is, the diagnosis came to light. So, I tried for 3 years to get a referral, and at one time I received it, but then I got sick, it expired, and they didn’t want to give me a new one, so. Who were the participants in this situation? Well, the main participant in this situation was me, naturally, and doctors were involved in this situation, but they were more like passive observers than participants. I would say my mother participated more, because she asked some doctors she knew there and so on. My girl too. Where did this happen? So, okay, let me tell you a little further, in general, I tried to get a referral, I didn’t succeed, then, in one private clinic... Well, in general it turns out that this operation is very rarely done without any additional shady payments, that’s it. And I, I have a principled position that I don’t give bribes for anything, well, in general, I don’t want to violate this life principle. Here. Well, in the end I decided to do this operation for a fee and it was unsuccessful. And if... Which I perceive, in fact, as a wrong choice, that I probably should have taken a more careful approach to choosing a doctor.

Q. A year ago. A year ago I had this operation, here. Then I felt pretty bad for six months, well, it was very bad for probably three months, so then I made a decision for myself that, well, sort of... Well, first of all, I don't blame myself for this, for the fact that I there may not be enough... First of all, there is no guarantee that if I tried another doctor it would be better. M. Well, yes, there's no way to check it. V. Yes, there is no way to check. Here. And I was guided by the recommendation that was given to me, that is, in principle, I'm not just like - oops, I'll go to any doctor. I've really come a long way, but the fact that it ended in failure does not mean that the path was wrong. M. Well, yes, it's not your responsibility. You did, you approached this situation responsibly, as far as I hear. That is, they did everything. V. Well, yes. So, in this regard, I calmed down. At first I kind of thought, damn, maybe it could have been better, then I thought - no, better, well, much better, as if I did everything I could, for my part. This is the first moment. And then, then I decided that I needed to get a good examination now, that I could... And then I kind of asked myself - what can I do now, that's what I need to get examined again now, I started knocking out all sorts of directions, there same level of hormones, what can be done about it? Here. And I began to move in this direction, received a referral to doctors, so. And then I started dating the girl I'm dating now. When I was just going through the referrals, and before I started dating her, that is, well, it was as if we had already started to have some kind of romantic relationship, so. And I tell her that well, right now I'm not at all sure about any sexual things that I can give you, so I say that let me first go to the doctors, they will prescribe me some kind of adequate treatment, and.. It's just that if I'm in a relationship, then I will feel uncomfortable, that maybe I'm not giving something that I should give in terms of my ideas about relationships. Here. And in general, somehow in the end I... By the way, this motivated me to speed up in that direction, to move, specifically to go to doctors faster, and to get directions with greater intensity, that's it. Well, somehow we continued to communicate with her, with this girl, and in the end all my functions returned to normal on their own. Here. Yes, but this happens, there are paired organs, the functions of one are taken over by the other. Here.

M. Tell me, do you remember the transition from your state of complete hopelessness to the fact that I can do something and in general, well, I can be responsible for my life and can take some action? So what was this connected with? Any external circumstances or more of your internal transformation? V. Tax. (...) Now, I'm trying to remember. So so so. (...) Well, at first, yes, I would probably describe it this way: at first I was just in shock, I didn't understand what was happening at all. Here. Then I began to feel the consequences, that I began to gain weight, and then I began to worry. Well, that everything is not going according to the best scenario that could have been hoped for. Here. Then, it's not like there was some kind of sudden event, it's just me, this question bothered me, it's like, I like to talk to myself, so. According to the method, like these two chairs, like what are you experiencing. How would I do something like this? So, this guy talked, so, okay, what do we want? We want to worry, or we want something, not to worry. Here. Let's think about what we can do. So, well, we, I mean, I just address myself like that, (laughter) here. M. Well, maybe some parts of you. V. Yes, yes, we are... M. They decided to act together. V. Yes, yes, yes. Well, yes, we are me, torn apart by the contradictions that exist within me. Here. As a result, I talked to myself, came to the conclusion that I... Yes, another thing... Yes, I remembered, I was very worried, really very much, and it always scared me... And I was even worried not so much because of what happened, but because I didn't understand what to do next. Here.

M. So the uncertainty was scary? V. Yes, I didn't understand, that is, which doctors to contact, where to go, that's it. Both then and there there was still such a problem that everything is very slow in our healthcare system, that is, there, for example, they give a referral, but the appointment is only a month and a half later. I'm like damn, what am I supposed to do for a month and a half? I, I'm very worried about this, but I can't do anything other than this recording. M. Yes, it's terrible. V. Well, that's when I started writing complaints, this greatly speeds up our healthcare system, by

the way (laughter). Here. Moreover, even those to whom I did not write, you just tell them that you have already written one complaint, immediately become more and more talkative. Here.
Well, a good choice, a good choice, I would probably attribute to my first university, that is, psychological and pedagogical education. And again, this is one of those choices that I made, guided by one criteria, and with the hope of one result, but it turned out to be good, that is, the results turned out to be different, but better. Than I could have imagined. That is, I chose a psychological-pedagogical course, because I knew biology, chemistry, mathematics, Russian, with these subjects I could enroll either in some kind of biological-chemical thing, like there you could go to Timiryazevka to study genetics, or to honey didn't pass. Well, in short, there was an option, either to go for something like that, biological, I was basically interested in genetics, at school I loved problems on genetics, but on the other hand, the option was to go to a psychologist, a psychologist-teacher, that's it. And, in fact, I thought, damn, now I'll go see this geneticist. Although I like solving these problems, is it that I will spend my whole life developing new plant varieties, animal breeds and stamps, strains of microorganisms? This is like the definition of genetics, actually. (npt) I think, God forbid, this is boring. I want to communicate with people. And I went to become a psychologist-educator. And why is this still a good choice, because I really liked my university, the Moscow State Pedagogical University, it's not very overloading, I can, as a person who has already studied at a bunch of universities, and who has many friends from different universities, I know that it is so, moderately stressful. And there was a little more tension in the first year, and then just not at all, not very much. And I really liked it, because I think that if I had studied at another university, I would not have been able to simultaneously learn French, for example, to a high level, because I would have to complete some endless tasks there. And I would not have been able to gain work experience either at Artek or at Detsky Mir, or as a chess teacher, I was still working. In general, I would not have gained much work experience. I would describe the choice of university as successful, but now let's talk about your criteria. So, when this happened - in 2015, who was among the participants in this situation? Well, I can say that naturally I was the main actor, but here my parents were naturally involved, like the parents of many children after the 11th grade, so. They gave me some advice there. I probably made this choice at the dacha. I was just wondering where, well, I was looking. I sat with the lists and in the end decided that, well, I went to different ones, and decided that psychological and pedagogical education, it still sounds universal, like a psychologist and a teacher. Of course, this is actually a trap, if anything, it's a trap. In fact, you won't be able to work as both a teacher and a psychologist (laughter) because you don't have a subject to teach. (laughter) And the psychologist is also only a school psychologist. Here. (laughter) But I didn't know that then.
M. Do you have any hobbies or interests? K. Yes, there is, this is football. (?) I used to play and watch, but now I just watch. There for health reasons. Problems have arisen, which is why there are various injuries that are now preventing me from playing football. Here. From sports I would probably say table tennis, in addition to this, reading, various things, including scientific literature, geography, waste sorting and the environmental agenda. Then I'll probably say, (..) well, let's put it this way, yes, the fight against domestic violence.
M. Would you be comfortable telling us in more detail what you are doing in the fight against domestic violence? If you feel uncomfortable, you don't have to talk. K. I can, but now, for the most part, this is support for foundations and coverage of their activities there, that is, reposts somewhere, somewhere to talk about various actions, about what the foundations do in principle, for example about "Violence.No", I visited them several times, well, at the foundation itself, there, and I regularly post something, that is, about what they directly do, as possible, and who you can contact there in case of cases of domestic violence, as possible with to help with this, where to go

for help, what other resources are there to help such people, and well, in principle, helping yourself in such situations. M. And again, yes, such an interesting, sensitive topic, if I ask some question and he is uncomfortable, please feel free to refuse to answer. OK? K. Yes, yes. Yes. M. Yes, I would also like to ask, what made you support these funds? Maybe there was something that made you make this choice? K. Well, there wasn't any specific trigger, I'm a lawyer by profession, and I had a legal education at a university, and before that, when I was studying law on my own for the Olympiad, I more than once came across the problem of domestic violence, including , when there were amendments to the criminal code, when certain offenses were excluded and, let's say, beatings were not punished. Here. And at that moment I misperceived this problem there, and there I did not really perceive it as a problem later. It seems to me that I have several friends who also covered this problem, this topic. Also on various social networks. And I myself decided to look deeper into this and realized that everything was much worse than I thought. And, (..) well, then somehow it started to grow like this.

Well, next is probably health. A year ago, well, a little over a year ago, (..) I had an operation there on my leg, to straighten my toes, that too, it was a necessary operation, and after which there was six months of recovery. Here. And... M. Necessary, that is, it really interfered with your life? Has this problem been around for a long time, or has something happened? K. Well, about two years ago, even 3 years ago already, it appeared, at first I didn't do anything, it went away after some time, there after ointment or some other such exercises, but after that the condition began to worsen, and It got to the point where it was painful to walk. Here. And so a decision was made there, here. And this summer I also thought about having the same operation on the other leg, but now all this is being postponed for me. Perhaps next year, perhaps even the next year. Because there are (..) various issues, including financial, time, because you definitely need to spend a week there under observation in the hospital, and after that you need to wear special shoes, and now it will soon be quite cold. It will be uncomfortable to walk in such shoes, so I am putting off solving this problem.

Here. Finance. Well, here the choice is probably only within the framework of finances, it's about health and about resolving some issues in this regard. M. Is this about leg surgery? K. Yes, including, plus other various problems, there in the summer I decided to do dental treatment, so. Also when it started to become quite expensive, and then other ailments appeared that needed to be urgently addressed medically, so. Therefore, dental treatment there, too, has been postponed and is being postponed there indefinitely.

M. Yes, Dima, thank you for the detailed story. I have a few clarifying questions. Firstly, I notice, well, naturally, here there is a moment of professional sociological education, albeit lasting one year, that you use a lot of terms, and with them you seem to indicate some kind of processes that arise in your life, but according to Basically, I have the following question, sometimes you say, for example, that there's this, this stupid procrastination, or... That is, you give some kind of assessments, and in connection with this it's interesting, but you're with someone... Are you discussing it now, or have you discussed those difficulties, including psychological ones, that you have encountered, and sometimes, well, naturally, they arise in life? D. I discuss them, but I discuss them mainly with friends. With my family, (.) I don't have the habit of sharing anything in particular and asking for advice, for some reason I have a big prejudice in this regard. It just seems to me that older people, my mother is a provincial teacher there, she is unlikely to understand (.) the things that I can tell her about. And you don't discuss it with friends, but only after the fact. Now here, with newfound acquaintances, I can discuss what I went through, but every time I go directly through a crisis, I usually don't share it with anyone, well, also probably because I don't think that anyone something can help me with this. Therefore, this is only after the fact.

About training, professional development - I decided to engage in self-development in the field of programming, I selected courses for myself that, in an amicable way, I would need to take. Because, in fact, I personally plan to work in the field of (NPT). Well, that's probably all. Health, I’m in... Six months ago, when my dark streak of procrastination ended, I started playing sports again, going to the gym. And during that period, I tried to arrange healthy meals for myself in the hostel. I stopped subsisting on some fast food and other semi-finished products and started cooking for myself. choose a diet.
I try to be kinder to myself and to others. Excessive self-flagellation and excess severity towards your friends, it rarely bears fruit, I try to refuse it. (...)
y, the choice of lifestyle and leisure, well, perhaps nothing has changed here, except that I began to drink less, because the first six months of my studies, when you constantly sit in the dormitory... Here, in fact, besides (..) drunken pastime, You don’t have any special choices anymore.
Okay, I'll try now. Training, professional development, probably six, making a choice may not be that hard, easy to say (npt) here to understand. But it is difficult to implement this choice. Health, probably three, but in fact, the gym and healthy eating take away almost everything from me...
B. Well, it’s quite hard for me to say about my peers, but I don’t think that everyone actually thinks about this in any specific way, well, that is, it’s still some kind of process that happens one way or another, in most cases, on its own with myself. Personally, I’m just getting this from, in principle, this is part of why I decided to take part in this interview, I’m interested in the topic of psychology, I have a good friend who is studying at Moscow State University of Psychology and Education, if you know, to become a (NPT) psychotherapist.
B. I personally find out about this from some sources, like Telegram channels, Instagram of some well-known psychotherapy services that run, some of them make short posts on some topics, that’s it. Well, sometimes I read some articles, but just considering that I don’t seem to know much, I approach it carefully. But in general, this is probably the most important source of information about this topic. Well, about other, some psychological topics too.
K. (.) Yes, these are some restrictions on the part of the parents, because of vision, for example, you couldn’t play on the computer for more than one hour a day, there are some restrictions on the time spent on the street, that is, when to take a walk there , restrictions...
decided to carry it myself. (..) Well, I went up to the guys, near the tents, to the boys, I said - boys, can you please help me carry the suitcase, I can’t do it myself. They told me - no, we can't do it now. I say, okay, okay. I'll carry it myself. Here. And somewhere in the middle of the journey, I already realized that I simply couldn’t cope, that this rib was starting to ache a lot, there was pain. And I wrote to the chat with the guys, I said - guys, the boys really need you now, please help. Here. And no one reacted at all. Here. And I just walked, roared, carried this suitcase, so I carried it. But most of all, I don’t know, what made me laugh was that I was walking, and so I met many young people, they were just walking like - damn, she’s so cool, really, strong, independent, carrying a suitcase herself, in general. I walk and think... M. And it was very painful for you. S. And I walk, roar and think - you didn’t think of offering help, somehow, I don’t know. I even, I don’t know, I always somehow offer help... (..) I, I don’t know, I’m used to doing this, so. Then I just went to the doctor, they told me a lot of things about how I shouldn’t carry heavy things, but I went and did all this, that’s it. So. Do you often think about it, about this situation? (.) Well, recently, often, because it happened recently. (laughter) Here. I told my parents, my mother said that everything can be expected from me, and what emotions arise? The emotions are so mixed. M. Can you explain? S. Well, in the sense that yes, I myself was able to convey all this, but it was to my own detriment, but also that, as it were, I don’t know, we were always told from childhood that boys should help girls and all that. And then they just go like this...

M. Where do you study and for whom? M1. I'm studying at the Higher School of Economics to become a psychologist.
M. That is, it’s like the ability to solve your problems, the ability to choose how you want to spend your leisure time. Tell me, in setting your goals, do you show independence, focus primarily on yourself, or does someone influence you? M1. Yes, of course, I primarily focus on myself, that is, for example, with the same boxing, as it were... (..) I really, like, all the girls are like that - boxing, why, why do you need boxing? I’m like this - I want to go boxing, that’s it. (laughter) I went, found it, went, liked it, and now I go, and that’s it, everything suits me. If it concerns some goals of others, then yes, I can set goals for myself, I can fulfill them on my own, but here it all comes down to financial dependence, sometimes I cannot achieve a goal due to the fact that I do not have the funds for it implementation.
Well, perhaps, when my neighbor became ill, she began to choke, and... That is, a person is suffocating, he doesn’t know what to do, this is his first time, and it’s like... That is, I called an ambulance, there I woke up everyone , she said that yes, we started googling something together, what to do and so on, that is, as if she had something like this, nervous, panicky, that’s it. But then she went to the doctor, well, it was like, you know, an independent decision that at the moment I should help the person. Because she can hardly help herself. M. This was probably a very stressful event and somehow you reflected on the moment when - now I’m doing it, or did you just start doing it yourself because you realized that the responsibility was only on you at this moment? M1. I guess I just started doing it on my own. M. Do you think this situation played any role for your independence, or did you simply show what you already had? M1. I think I just manifested what I already had, because my sister is often sick, (laughter) more precisely constantly, so I kind of got used to packing things in bags when I have to go to the hospital, that is, for me it’s a familiar situation, so it was sudden in terms of its appearance, but in terms of (..) how should I say it, in terms of factors, it was similar to many situations in life.
M1. Now I'll think about it. (...) So, well, one successful one. (..) I was in the eleventh, no, tenth grade, I wanted a dog, and at that moment I already understood that I definitely (xx) would not stay in my hometown, in Rostov, that I would definitely move. It doesn’t matter which college I go to, but I’ll definitely move. And... But I really wanted a dog. And as if we were looking for a dog, we found it, we had already paid a deposit, well, like, in a few days I have to go pick it up, and I understand that how can this be, then I will leave the dog with my parents, because I will live in a hostel , well, for sure, I had a choice either not to get a dog, and let’s say, not to fulfill my dream, because I never had my own dog, we have a lot of dogs in our family, but we didn’t have our own. And no one knows when I would be able to get myself a dog. Or cancel everything, let’s put it this way, and simply not burden the parents. And that was the choice. Either you blindly follow your dream, then you burden other people because they need to take care of your animal, or you care about other people more than your desires. This happened in the tenth grade, I was probably 17 years old at the time, and me, my mom and my dad were involved in this situation. Because my sister doesn’t live with us, therefore she won’t take care of her. Where this happened, this happened in my hometown, this situation made me feel like I needed to decide something, that I needed to do something and that I was afraid of making a mistake. I’m afraid of ruining the dog’s life there, I don’t know, causing some kind of trauma to myself, or burdening my parents with this too. That is, the situation was not pleasant. The choice was not a pleasant one. But now, (..) I treat her calmly, I can’t say that she directly somehow excites something in me, probably, I just remember with some pleasantness all this then... All These are my decisions that led to the fact that I now have a dog. (..) It evoked different thoughts. (...) Don't even know. I think, probably, I was just very afraid to hang some kind of responsibility on another person, I was very afraid that the animal would forget its owner... And it’s not very pleasant for me either. (NPT) kind of like for

myself, but like for others, too, so. Now this gives me (..) also mixed feelings. Well, that is, I don't know why I did this then and decided to get a dog after all, and I don't know what I would do with this situation now, well, that is, it's like you just decide in the moment, yes or no, and probably Then I just let go of all the consequences and lived in this moment, that yes, now I want a dog, now let's live in this gorgeous moment, and then I will accept the consequences and decide what to do with them. Here. But I think that it turned out well, because I love her very much, I have a gorgeous friend who will always hug you, lick something, and so on, make you laugh, play with you, that is, how These would be very pleasant moments, memories, so I think that I made a good choice.
probably my sister also has health problems, so she, a little like this, somehow maximally, (sigh) I don't know, reaches maximum heights during education, and therefore, probably, this is also a little bit of my responsibility, (laughter) also everyone, and they also pay a lot of attention to her education, even more than mine, because it was more difficult for her to study, but certain hopes were probably pinned on me in this regard, but I'm not exactly (npt) on this topic of approval, that you should do something there, always do everything well, I don't know. (laughter) Well, yes, in general, this was also important to my grandparents, but what my grandparents on my father's side, basically, my grandmother was more likely to just say, go ahead, well, it was important to her that I do in basically anything, and I moved somewhere forward, and managed to do everything. But it didn't matter to her in what area I was doing it. Here. Probably so. M. But it turns out that both pairs of grandparents have higher education? I. Yes, everyone has a higher education, yes. M. I'll carefully ask about my sister: what health problems did she have, were and still have, what is the reason for this? I. She has autism spectrum disorder. But she is quite highly functional, that is, there is no such thing that she, I don't know, it's probably not very noticeable, in terms of... (..) For people who are unfamiliar with this, they probably won't immediately understand that something is wrong specifically. That is, they may think that she communicates in some strange way, or something, I don't know. Well, in general, not so much that she couldn't study, for example, but in life it interferes with her. M. But you said that due to the fact that your sister has such difficulties, sometimes they even paid less attention to you, and so you, well, how much did this bother you? That is, did it evoke any emotions? I. In general, no, probably (laughter) only because they always paid a lot of attention to me, and sometimes, I would say that it was even some kind of overprotection, so for me, I don't know, it's just very difficult to assess the effect and the cause , that, for example, they looked after me a lot, because my sister has difficulties, for example, for a very long time I was not allowed to go anywhere on my own, (..) well, on the subway, or on some public transport, and they were always driven, and it annoyed me because I wanted to travel on my own. And I don't know what this is connected with, maybe because my sister couldn't drive for a long time, and I started driving earlier, my parents were afraid to let me go. It seems to me that some of my parents' fears, as it were, related to my sister's health, are transferred to me. Well, at least it was like that before. Probably not anymore. But maybe, maybe I'm just thinking, in general, I've never had the feeling that they pay more attention to my sister, because I also received a lot of attention. And vice versa, probably at some moments it was good that this attention did not go to me and I, I had more freedom.
M. Did you feel older precisely because she had just such health difficulties? I. Yes, because for quite a long time, but even now, she still doesn't have it so strongly, for a very long time she behaved as if she were much younger. Therefore, at some point, well, I remember, right when I realized that she had some peculiarities, I asked my mother, by the way, I even remember this moment, I was driving, we were going either to a dance or with dances, and my mother began to tell me, I was about eight or nine, and she gave me a book to read, well, it's such a cool book,

“Autism through the eyes of a sister,” it’s like from the point of view of a girl whose brother has autism and she tells . I think yes, it’s quite similar, because some patterns of behavior are described there very, very similarly. Here. And when I realized this, I somehow began to relate to her easier, because when you know why she behaves so strangely and it annoys you, and it’s not clear what to do about it, when you know it, it’s easier. But still, I noticed that my sister acted as if she were younger, as if she were younger than me. Therefore, I didn’t have the feeling that she was older, and that somehow... In general, I was probably ahead in terms of development after all.
Regarding health, there are probably no solutions. At least nothing comes to mind. There, every year you just go to the doctor, and he tells you what to do and that’s it. (laughter) M. So you are generally calm about your health and therefore somehow don’t even remember? I. Well, I probably haven’t had anything like serious health problems lately, if something happens to me, I go to the doctor, but I don’t know there, I recently had poisoning, I go to the doctor, he me right away (npt) (laughter) but there were no such, well, particularly important ones, but probably if I had had any health problems lately, I would have remembered it better, but so, (laughter) nothing there was no specific one.
M. But it seems as if you have done a lot of work compared to the way you talk about your adolescence and the way you behave now. You are very open, you are very emotional, and talking about such experiences is a great courage. In fact. N. (laughter) I have chosen a psychotherapist several times and now I finally go to a normal one, and everything, plus or minus, yes, is being worked out, so it’s just... Well, that’s exactly what I’m talking about, about the fact that from the age of eighteen, I can finally make choices myself, and I can, as it were, in an emotional way, that is, choose what I feel, what I don’t feel, and what, I can behave this way and be like this, that is, in some moral things, and in some actions. That is, I decide where I will do the actions one day, and I chose this, and I will, well, kind of lead you like this. And be like that. This is also like a choice, in my head, it’s considered, because, well, just then, all these things, they were very strong, I cried there for months, and in general, I felt even more downtrodden, but fortunately, some things started there. then a fairly adequate relationship that lasted quite a long time, and this somehow made me feel that I was not at all... There is someone who loves me, someone who appreciates me, the same one like this... Replacing unconditional love with parental love, that's it. And probably, in parallel with all this, probably in parallel with this, there were also elections in the 11th grade... M. Can I have a second? Tell me, do you attribute your transformation to a greater extent to psychotherapy, or perhaps there was also some event that made you look at yourself differently and begin to behave differently. N. More likely, to a lesser extent with psychotherapy, but that is, I started adequate psychotherapy literally four months ago, because before that I just went to psychotherapists, and somehow, well, I couldn’t find someone with whom I would be comfortable, Here. So I associate it rather with the fact that the most important thing is that now I can, since I was eighteen, I do not depend on my parents, that is, if before that my parents could forbid me something and somehow control me, now, well, this is absolutely not the case, and we have some kind of, well, adequate distance, Well, you know, there is an expression, healthy indifference, something like this, probably with us. It’s clear that I love them and this is unconditional and absolute, but how could I...
M. And after 18 years, what important elections did you have? Besides work. Maybe in some other areas? Independent travel, I heard this spring, maybe something else? Choosing a psychotherapist? N. Yes, choosing a psychotherapist. That's for sure. (laughter) An important choice, I’m very glad that I found my current therapist, she’s very strong there. Yes, independent travel, because at first I started traveling around Russia, it was also funny, of course, because I turned 18 and Covid began. Like hello, thank you. Nastya, who had never been abroad before, or even in any other cities, here. Yes. So I myself began to travel slowly around Russia, sometimes in a company, sometimes on my

own, and then I decided - damn it, I've never been abroad. Here. And yes, in general, I'm very glad that I went to Istanbul, so. Because I went there myself too, that's it. That is, completely alone, not knowing much (laughter) English, that's it. But it was great and I'm very, very glad that I went after all, because well, that's exactly my trip, I bought tickets there a few weeks before February 24, and the tickets that I repurchased were canceled 10 thousand times , and I was already thinking, damn, how am I going to go in such an atmosphere, but I went anyway. And I'm very glad, because... Well, at least this ticked some box, because you still felt as if you were in some kind of geographical cage and (npt) get me out. And this summer I also went to Dagestan, on my own. And this was also wonderful, it seemed to slightly support your own feeling that you are doing something with your life, and you are sort of solving not only some work or romantic processes, so. But you also allow yourself to receive some kind of pleasure. Here. Yes, what other elections, well, probably a partner, we still have to talk about it, because this is my first breakup with that young man with whom we dated for two and a half years, this is probably an important decision that I accepted. And after that we dated for a year, because I, in fact, I left him for another partner, with whom we were in a relationship for a year, it was such a very cinematic relationship, with... I think you know the expression Dead Inside, here, (laughter) this is it. (laughter) So, in the end, we parted ways, well, that is, I can generally say that he abandoned me there... Because of his own pens, because he was somehow against me projected, as they say, and this was also a rather difficult period, just around March - April, all this was also layered on February 24, and (..) there I did not make any choices, (laughter) this is exactly there was a moment when I directly felt that I had lost some control, and because of this, well, it was really very difficult. Well, it's also related to this, that is, I found a psychotherapist right on the same day. Here. And thanks to the fact that I decided to find her at all, and because I found her, I somehow, well, generally survived all these moments, because of course the first separation was very difficult, well, actually without your will, which was done. Here. Well, then there, after some time, it seems that I met a young man with whom we now have, well, some kind of relationship begins, this can also be considered some kind of, well, choice in the romantic part is important, because Well, there is 50 percent of work that calms you down, and there is also, well, it's clear that it's not 50, there are other breakdowns, probably 30 percent of work, 30 percent of relationships, 30 percent of friends, and then there's 10 percent, this is some kind of totality , oh well, the family still needs to give some part of the interest, so. Well, that is, I just remember that just when I was in Dagestan, I felt very happy, because finally everything in my life, plus or minus, seemed to work out, and this, well, if you look at all these segments, Well, they're pretty good. Here. That's why...
M. No, on the contrary, one is easy, but ten is difficult. So then you have three in training, right? N. Yes, yes, yes then for training three, and (..) health - (..) one. That is... M. Easy? N. Yes, well, you go to the doctor, make an appointment there (laughter) if something worries you.
N. Well, then, I think that I can go back to the choice that I made when I was 16, when I was in the summer, when I decided to go to the young man with whom, who hinted to me for sex, for some kind of romantic relationship, that's it. And to his friend's house. Well, accordingly, I was 16, that is, what year was it, 2018, yes, it seems. Yes, summer 2018. Here. (..) Accordingly, I was there, this young man was there, and his friend was there. And so I ran away from home to spend time with them, because it seemed to me that it would be cool and that I would probably get closer to him, and indeed, he would kind of like me as a person, and that's how he would like me probably appreciate it more. Well, in principle, I liked the feeling that he was paying some attention to me, that a person seemed to appear who appreciated me, at least for something. Here. Maybe sexual desire, and it seemed to me that, well, in general, I could probably somehow, well, show interest, and he would appreciate me even more, but for something else. Here. And all this took place in the

Moscow region, at his dacha, in his house. And... Well, it happened, (laughter) how to describe it, I just don't want to go into any super details, but (..) in the end, after we first talked with his friend, who told me what - there with these vodkas, so, and then this friend himself came, oh, not a friend, but the young man himself, with whom we had some kind of fuss, so, and he took me upstairs, and then I continued I remember it very well, (..) just in very small snatches, here. But it was all connected with some kind of (sigh) (..) well, sexual actions. In the end, I don't know how he would have entered me or not, but he tried, let's say so. Here. And what kind of feelings this situation caused then, obviously - not the most pleasant ones, well, that is, I was probably in some kind of teenage age, so that everyone, I was just, I was just worried about everything. Well, it seems to me that everything was just finishing me off gradually, that is, it wasn't so much the situation itself that was finishing me off, that is, the fact that... Although, well, in general, it's difficult for me to separate this for me, the fact that we then stopped communicating with him , that is, he seemed to write to me at the beginning, well, that is, maybe he wrote there twice, so. And that's all for me... (..) Well, sort of, the fact that he's no longer interested in me at all, and the fact that he absolutely doesn't need me, and the fact that no one loves me, doesn't appreciate me, I I felt absolute loneliness. I remember that I imagined that I, firstly, well, that is, I just had pictures in my head, like I was alone in the middle of a white sheet of paper, and in general there was no one close to me, as if my parents don't give a fuck, my friends and I are so close We don't communicate, that's it. And, well, that is, it just felt like such total loneliness, rather, this whole situation led to these feelings, so. And what feelings now, (sigh) does this situation evoke, now, for a second. (...) (sigh) Well, probably (..) I just thought it through, thought it over 10 million, billion times, turned it over in my head, and somehow the psychotherapist and I seemed to have worked through it, give or take. .. And now I just feel some kind of, I don't know, sadness, sympathy, probably for myself, but it's global, because, well, obviously none of this would have happened if I felt somehow there much better, that is, as confident and calm as she is now.
N. (sob) Thank you, it's very nice to hear all this. It's just that now it's actually much easier for me to tell all this, because it's all already agreed upon with my loved ones, and well, my friends, my former partner, or my current partner. And to the psychotherapist, here. That is, like another year or two after what happened, of course it would have been more difficult for me to tell all this, but now it's like... Well, you just understand that... Well, somehow in general, it's all rational, meaningful and... But thank you very much for the words of support, it's very (..) nice every time, me too...
M. I really like the way you praise yourself, it's really great that you acknowledge your victories, understand and build it into your story, you understand that yes, I really did it right, and now I get what I wanted, and even unsuccessful elections in the past become not so unsuccessful; on the contrary, I am very glad that I made a different decision then. N. Well, I think that this is all kind of connected, plus or minus, with the fact that I turned eighteen, I began, plus or minus, to somehow adequately perceive myself and the world, and then again, now it's like I've added to all this psychotherapy, and but I just, as if my emotional intelligence is growing a little, you can, well, somehow listen to what you feel and already determine what you feel, and not just feel and be like - oh, I feel bad, (laughter) here.
As for health, well, as such, well, firstly, I chose to do a health check-up in order to identify some... Some areas in which there are some problems, this was already a big step, because I couldn't get to a regular doctor. And so I chose not to put off some moments. Of course, now, with a new workload, it's been postponed for some time, but I think I'll get back to it soon. (..) And I will start again the procedure that I was doing. M. And what about regular health checks? How regularly will this happen? A. Regularly - this is six months. (..) And about work, work... M. Sorry, please, can I ask you more, about your decision about regular health checks? A. Yes, of course. M. How

did you decide to do this? How did you make this decision? A. (..) Well, since we now had Covid, I... And I never had confirmed Covid, I decided to go and find out if everything was fine with my lungs and everything else, and since now there are pavilions in Moscow, health pavilions, and there is one of them literally next to my house, I thought that it would take an hour, an hour at most, and I have an hour of free time, I can go there and do it. And also, well, when... (..) When you underwent medical examination at work, mandatory, it's also like a choice that we are not given, (..) what you are obliged to do, and also this, well, some your own feelings that something is wrong and you should go to the doctor, and he will refer you where you need to, according to your needs.
M. Yes, of course. If you think that's it, move on. A. Relationships with parents. This is a very difficult question, because... Well, I have always had a very (..) (h) great attachment with my parents, because they literally did not let me go anywhere, and it was this year that I decided that I would completely separate from them, I worked with a psychologist about this. And... (..) And I tried to distance myself from them as much as possible, because I understood that I was already independent, I was already old enough to make decisions on my own. And, (..) well, not even listen to any advice, realizing that my experience, conditionally, of living in Moscow, studying and working is completely different, like theirs, in another city, and since we are completely different generations , because, well, my parents are adults. Here. And in this regard, it turns out that relations with parents may have cooled somewhat, and they are not as active as they were before. (...) Are there any additional questions on this point?
M. Please tell us in more detail how it happened that you wanted to start volunteering? A. I accidentally saw an announcement about holding a marathon, in general, in my city, I had to run it, but there was some kind of large fee, because, well, I used to do athletics, and I thought that I could run, but there there was some kind of large contribution, and I thought that well, I don't really want to ask my parents for money, and I saw that volunteers were needed there, and I simply wrote to the post office. And after 2 months they answered me, here, come. And then again, again, again, again, and so on, now several projects a month. (laughter) Or maybe even a week, and in general, now no longer as a volunteer, and not only there on all-Russian, but also on international projects. Even now, no longer as a volunteer, but as a manager of volunteer projects. M. Great. Is this related to sports, are these different projects, or is it one direction? A. We did this with sports, but now sports are a big, well, sports projects, they are a big part of my life. And the rest of the projects are of different directions, just the very fact of volunteering, the very fact of helping, it remains unchanged. M. Can you tell me what feelings the situation caused you to decide to volunteer? A. Then it was very interesting to me, because this is new communication, this is a new community, these are new opportunities, because I really missed communication exactly in adolescence, then it caused a feeling of such, perhaps instant admiration, that is happiness is in the moment, now this also happens from time to time, precisely at some event, but you just realize that you have something to do, and you can go to any event and go help someone. M. How do you feel about this now? A. Now I'm completely delighted, because you live this, you love doing this, when you're at some event that you really like, you just almost cry with happiness that you're there.
And probably one of the things that helps me very, very much is that I have been involved in sports since childhood, at first I went to a circus studio, probably from the age of 4 to 9, I really liked it. And I liked the fact that there is freedom of creativity, and there seems to be some kind of framework, in which you have a coach, you have your leader, and you, for example, put on a number with him, that's it. We also traveled a lot and participated in some international events, and I probably even received my first salary, well, as I believe, I received it abroad. And it was interesting, because these (.) were moments that relieved the fears of the unknown, because for example, we performed in front of a huge audience, there on the streets and did some kind of

performances, despite the fact that we were all small, we in fact, they were very shy, that's it. We somehow overcame ourselves, and it was somehow unobtrusive or something, because we perceived it all as a game. And now I understand that I can also use this approach in some life situations.
And I kept asking, asking my parents to bring me to the gymnastics group, and then I still don't know, maybe my persuasion somehow influenced my family, maybe something else, that's it, but I still They brought me in and I started playing sports. And I understand that it's probably sport, it has played a lot in my life and in my character, too, a lot from sport. I have there, regarding the achievement of any goals that I set for myself, that I can gather there, even if I understand that it's hard for me, bad, or something else, I understand what I have after all, there's this kind of core that probably doesn't allow you to give up when you just need to pull yourself together and do something, come up with something, that's it. Or somehow cope with the situation that exists. Here. And probably the fact that in my family, in general, (..) there is such a moment of complete freedom. That is, I chose, after school I entered a technical school, because I did not enter the university, and my parents said that you choose a profession and direction yourself, because we want you to be a good person. And of course, perhaps, I think that this was bad advice, because when you are 16 years old, and you want to get specific, well, some steps, how to choose, what to do, maybe how to look at the test results, according to career guidance, which shows you 16 areas of activity, and you don't understand what to choose. Here. But it seems to me that this is my path in education, it seemed to be a little in different directions, but it seemed like it was almost cultural, almost literary, probably. Therefore, somehow everything comes together, as if like a puzzle, here you are, seemingly from different places, but you find puzzles that your picture is missing. It's probably something like this.
P. Health, oh, I had to choose several clinics, here. And I also read there, looked at it, asked some people who live there for some advice, what's what, what's best to choose, that's it. And that's probably all there was in terms of health. M. But where do you usually get advice regarding clinics there, and the health sector in general? P. Here, as advisers, I probably have experienced guys from the dormitory where I lived, because (..) they, well, I asked the guys from the second year, and I asked, because we had several clinics around the dormitory, which is better, is the university clinic better, and so on. Here. Plus, I looked at the reviews, and looked at more reviews on the main website of the clinics. Here. Well, I chose and I'm happy with the choice. Because everything somehow turned out very cool there. Well, just one piece of advice that I was hooked on, I read it, and I realized that this is a great clinic, and now I'm really happy
stopped playing sports at the age of 16, and probably now, after a certain number of years, I would tell myself that I still need to continue to exercise, even a little, even a little bit at a time, maybe a couple of times a week , but still do it. Because at that moment I had completely finished studying, that is, I had some activities, not much at all, but still, here you go. But I wouldn't want to quit. This is probably the choice I regret. M. But if you go deeper, when exactly, how old were you when this happened? P. I was 16 years old, it turns out that I graduated from school, began studying at a technical school, and I simply did not study in the area in which I live, and not in the area, and not even close to the area in which I played sports. And plus it so happened that at that time the group in which I was studying was disbanded, and then I thought that, well, the coach and I also discussed that it would probably be better for me to look at some university or technical school somewhere. activity, because I trained with girls who are younger than me and from a sports point of view, this is kind of a step back. And significant, because if you train with those who are on the same level as you, or a level or two higher, that's good. And when you train with those who are kind of far behind you, it's not good. Because you, too, will roll back to approximately their level. Here. And then, yes, at that moment I thought that yes, it would

probably be difficult to travel, because school would end anyway, it would be very late and there was still a road to class, and classes too, and it would be difficult. Here. Well, then I bought a membership, a membership to the fitness room, that’s it. But it was also difficult with him, because he was also not so close, but still. Here. And I went and worked out, but I understood that this was not enough for me, and I just wanted gymnastic activity at that moment. M. But you said that at that moment, well, as an adviser, well, the coach was present in this matter, and who else? Who else was involved in this situation? P. No, just me, me and the coach. So, it turns out, I told her (..) my situation, and so, well, she suggested this option. Of course, she wouldn’t have refused me if I had said that I would like to continue, so. But she outlined the situation to me, as she sees it, as a professional, as a coach. M. But if, in contrast to unsuccessful ones, we talk about successful elections, what elections do you think were successful in your life? P. I would probably highlight the choice of sports, gymnastics, as especially successful. I understand that it's mine, and I understand that it was very important and great that I liked it. And I’m probably lucky in that this sport is interesting not only to me, but also to my family, and my family really supported me in this, then the choice... (..)
M. Tell me, how did the desire to go to study journalism come about? Why did you decide? L. Well, in general, until the seventh grade I wanted to be a psychologist, so. But... M. How interesting. L. Yes, my dad is just a psychologist, that’s it. But in short, there was such a story that I began to find out how to enter the psychology department, and found out that I had to take biology there. M. Biology, yes. L. And biology was generally my favorite subject at school, and I say - no, I’ll look for biology (npt) for something else. And so I started buying all sorts of magazines there, these ones, for girls, (npt) then later, I became interested in all kinds of fashion journalism, and then it means, I don’t know, there in the eighth grade, in the ninth, I became more interested in politics, I wanted to be a journalist who travels to all sorts of hot spots, that is, something like that, little by little it became more and more boring, then I just wanted to be some kind of observer, that’s it. Well, that is, it was just some kind of interest of mine, based on some of my predispositions. That is, I was always good at writing texts, in principle, my language was so good, well, I understood that this is what I am passionate about. Here. M. That is, but it was as if it was a choice from the opposite, that if not a psychologist, then I would be a journalist. L. Well, I wouldn’t say that this is probably a choice from the contrary, that is, well, my interest in psychology never went away there, my dad always gave me some books to read, he conducted all sorts of tests with me, that’s it. It’s just, well, maybe I’m also partly burned out, because, well, I’m basically the kind of person who, if I really want something, I’ll hurt myself, but I’ll achieve it. Well, that means it wasn’t such a really great goal, because if this goal had been great, well, I would have learned biology. Well, what can you do? (laughter) M. Why did you want to become a psychologist? Because dad was a psychologist or is it somehow different? L. No, I actually found out that my dad is a psychologist after I decided to become one. But this, you know, is such a story that you never know what your parents do, and then you find out. Here. Well, in my opinion, it all started because of the series “Lie to Me”, I was very interested in this topic there, like body language here (npt) that’s all, it was so cool, then I read that There are sports psychologists, I thought that was also interesting. I wanted to be a sports psychologist. And then I began to get interested in serial killers, just reading their biographies, and I wanted to be a psychologist who works with all sorts of criminals, that is, he understands why they commit such terrible things. Well, maybe I also watched enough of some TV series there, like “The Crypt”, where I remember going to the dacha, watching with my grandmothers in the evenings, where a criminal is also sitting there, and a psychologist is working with him. Here. Well, somehow it attracted me because it was such a very socially significant profession, which, in general, was aimed at a good cause. M.

At what age did you decide to become a psychologist? L. Oh, well, it seems to me when I was maybe 10-11 years old, that is, before that I wanted to be an actress, well, let’s say, many girls in elementary school want to, and then I think - no, it’s too fickle, a psychologist - that’s the most That.
M. Yes, there is such a thing. Health? L. Health. Yours? (laughter) M. Yes. What were you even thinking about? L. Well, it often happens to me that I force my loved ones to take care of their health. At the same time, I don’t pay that much attention to it. Because for example, there, I don’t know, I think when I lived with my parents, someone’s blood pressure would rise, and I almost called an ambulance, but they didn’t do it. And in the end I understand that I did everything right. But as for mine, I’m like, damn, again I feel sorry for the money for the dentist. Well, let's probably say here then... (..) Probably a five, that is, somewhere in the middle, because, well, as far as it concerns words, I understand everything perfectly well. But when it turns into business, it is much more difficult. For example, I couldn’t get to one doctor for 3 years. But when I got down to it, I immediately realized that thank God. M. Okay. Job?
Well, not a profession, but probably just a hobby, I just understand that in fact all my energy is there, and when I step over myself, well, I forget to do this creativity at all because I have no time, but I begin to suffer from it. Sometimes I have some kind of blues there, I can even get sick, psychosomatics starts to turn on. Well, in general, these are the moments. M. Katya, tell me, have you ever had a consultation with a psychotherapist? K. Yes, by the way, it took place at the Higher School of Economics.
M. Where do you work? M1. I work in a private children's correctional center as a speech therapist.
M. Do you have any hobbies? Besides work. M1. Yes. I like to play sports, different ones, it could be yoga, dancing and I like to embroider.
M. Yes, but where, as it seems, maybe you or your peers can get information about what independence is and what an independent person is? M1. Well, probably more from... From psychology, but from some kind of popular psychology, because it is more accessible and understandable. And very popular now.
M. And in what terms is health? Can you cure yourself of an illness? Or make an appointment with a specific doctor? M1. No. (laughter) No, just decide that I need to understand on my own that I don’t feel well and go to the doctor and understand which doctor.
M. I see. Great. Do you have any hobbies or hobbies now, do you work in the summer or in general? N. Well, I worked, but I worked in Yandex, in Yandex.Textbook, in an educational project, then I left there because it was... I worked in support, and in computer science in Yandex.Textbook, Well, in computer science, I left there because I had family circumstances there, and I had to write a diploma and everything else fell in, well, just because of stress and plus the manager changed there, so it didn’t suit me and I left. So, I was in Moscow as a psychological internship in my fourth year, I did it for a charity foundation in “Shalash”, so, I don’t know whether you know or not? M.
M. Well, about this and that. N. Well, in general, I’m interested in the sphere of parent-child relationships and the sphere... Well, in short, working with children, with teenagers, as if I, which in my experience was not very clear, seems to understand what to do as a teenager, so I kind of do everything... Well, I just like this age, that the worldview is formed there, and so on, and so I kind of want... I would like, for example, to have such a person, to To whom I would come and somehow tell everything without judgment, for example, he or she would simply listen to me. Well, in general, I would like, for example, to have such a person, if anything, like a school psychologist, so. At my school everything was like that, well, very like that, not humane, that’s why... Well, in particular, that’s why I went to the psychology department. And in general, I want

to be more careful with children and teenagers, so that they can be treated, and, well, treat them in general. That's why it's like this.
And there's this very thing, I don't know when there will be grandchildren, when I'll get married, that is, there's this very insistence on a certain way of life, there are some expectations from the parents, from society, and I feel them, but at the same time I kind of went through psychology and psychotherapy, that is, it all seems to help me, somehow my beliefs, to live according to my own beliefs, and not social ones. But I still feel like it's frowned upon. And for example, when you don't listen to someone, it's condemned. There, when you are, as it were, in the minority, this is also condemned, that is, it seems even to me now, this situation, which is the war with Ukraine, that, as it were (.) some people who are vehemently opposed, or something like- then, in general, some people stand out from the masses, they are always, well, they are always perceived negatively, it seems to me. Here. And when, for example, you are different, it's just somehow scary as if people can be different, and this seems to me to be such a problem that it happens because of this condemnation that you are somehow different. Here.
M. It turns out that the Department of Psychology helped me to be more independent, if I heard correctly? N. Yes, it seems to me that yes, and it seems to me that it's just plus the people there, my friends and my husband, it's like, that is, we somehow switched to a more healthy (npt), healthier kind of then the atmosphere, somewhere there it is normal to experience some emotions, well, emotions such as anger or hatred, or... Well, in short, that is, which previously seemed to me that they should not be experienced, or that it is bad to experience them, or something else. Well, in short, there is no plus about family (npt) there (npt), that these are all sorts of non-adaptive patterns and so on and so forth, but how very upset I would have been at first when I took this subject, that this is how bad everything is and all that, but then I thought that, in general, this happens not only in my family, it happens even much worse than in my family, for example, and that parents, they just seem to try as hard as they can, that is, they seem to they also draw conclusions for themselves, based on those who raised them, based on life experience, and what they learned is what they passed on to us, so, and we can then also take it out and pass it on, and so on it will always be like this, and therefore it seems to me that this is, in general, a positive dynamic, that it is, in principle, enough, and not what you say there - this is bad, this is not the same, this is not adaptive, this is something else... That.
And then, and also the fact that I was probably not afraid to take specialized mathematics, because to become a psychologist you need specialized mathematics, and it was like, it was very difficult for me, and every time it ended... Andrey, my husband, he seemed very He's good at math, but he tried to explain it to me, I didn't understand, it ended in tears every time and all these tutors and all that, I didn't like it at all and I literally forced myself to learn it. But I'm like, I want to take psychology, I'll pass and I'll never need it again. Well, how important it was for me to pass it well and then I passed it, and I, I had such, well, it seems to me that such a low score for admission, but then I was very... At first I was upset, then I was very I was happy because it was an entrance exam for psychology and that I was going to get into it after all. And I was very happy about this, and my decision, and the fact that I passed these points, and my decision to go to the open day and to enter the Higher School of Economics, although I did not see other universities, that is, I did not go there and didn't scout anything. I sort of decided that I needed to go there.
N. I would rate it a 10, to be honest, because my parents, my dad, were very much against me going to the psychology department, he wanted me to go to law school, I have a mother, her first education was in medicine, then she studied in psychology. went, but she was like, well, it's like I want to be like her, she thought, here. But I didn't go there because of her, I was just interested, as if about the people, there, like with the people, what's in their heads, how to understand it all, that

is, it was somehow for me it was interesting about behavior and emotions, so I kind of... And so my mother says something to me: go to these courses, go to these courses, there's something else, and I say no, I won't go, I'll decide for myself what courses I should take, or my dad says, I need to go to a master's program somewhere to become a lawyer, or something like that, and I say like, no, I don't want to, or whatever Here I am this year, well, in the next academic year, for example, I won't study, because, well, like my parents, they would rather have me go to a master's program, but I just understand that I'm tired of studying (laughter) that I can't, (laughter) I need rest. (laughter) And that I just don't understand where, what kind of master's program I would like to go to, I didn't understand, and therefore I didn't go anywhere, and how would I make this decision for myself, how would I want to work and understand maybe there's a better place for me to go. About the job, I would probably rate it a seven, because my first job was in my dad's sales office, that is, my dad, he owns a construction company, and I worked in his sales office. But I wanted to work myself, as if I wanted it myself. And I kind of came there every day, stayed there for some time, they already told me like - go home, but I was like - no, I still need to do this, and I tried to come up with something there, whatever didn't come up with it. Here.
M. What about making everyday and important decisions? Here, in fact, we are, of course, slightly duplicating what we have already discussed. N. Yes, yes, I understood. (..) Well, I think, it seems to me, let it also be seven, because I, well, in general, it seems to me that this is a process that there are no correct ones... In general, I'm kind of really worried about there right, wrong there, or how it will affect others, or something like that... Well, in short, I worry about this in every possible way, and it seems that the more I work there with a psychologist, well, the more I see, that no matter how people react normally to my decisions, the more calm I am about this, the calmer I begin to feel about it, so how would I consider that it's seven, there I'll just be there later, well, what's my attitude specifically towards making a decision , it's very similar, it's quite saturated with anxiety, so I wouldn't say that it comes too easily to me, but I kind of understand that I always make adequate decisions, plus or minus, I just have problems making decisions, that's it. And the choice of lifestyle, leisure, well, right now I would probably say that (..) six, because I think that (..) well, I just feel that I'm a little burnt out and tired after studying there, after school, when things weren't very good there either, at the university, when there were some difficult moments there, that's it. And it seems to me that I just need to recover now, just somehow settle down, that sometimes I get shaky, like how I behave there, or how I need to do or something else, well, like a lifestyle, and for me, I'm just now focusing on the fact that I need to rest, and otherwise be more careful with myself. Here. And about leisure, I don't really like spending time in company, I rather like one-on-one communication, and well, I kind of, (.) well, I don't do any supernatural leisure, like, for example, I knit there, or maybe I have some kind of hobby that is very accessible, well, drums are not very accessible. That is, I disturb everyone, and it happens that they irritate everyone, but as if I could occupy myself with something, I don't feel like I'm very bored, that is, in general, in every possible way... I sometimes scold myself myself for rest, that is, it's as if I can't rest, because I'm not tired and all that. But this is again a balance, there is a work life balance and therefore... (...) Well, about leisure, I meet with friends, I read books there, watch films, some educational things there, well, educational videos on various there platforms. Well, in general, I do, it seems to me, what I want, what interests me, and it seems to me that this is enough. Here. Romantic relationship. (laughter)
M. What do you mean, what didn't coincide? I didn't quite understand? They said that it was possible, but... N. No, rather that they... Well, for example, dad said that you should, for example, not lie, that it was better to be honest, and then it turns out that he has a second family there, that he him there... Well, in short, (laughter) some secrets from us, roughly speaking, that is, that he says one thing and does another. And that is, as it were... It doesn't work out, then there is no clear

picture, you still understand that there is this duality, and not... (..) Well, what is it... And you begin to think like about some of your actions, that's it. That's what I'm talking about, that there weren't any where, for example, they told me - go and do it, for example, my mother said - go there, do something, take an interest there, something else, if only I had such opportunities as you have, then I would have done this, that, that, but she, despite the fact that she seemed to have opportunities, she practically sat at home with us all the time, that is, she developed, some She worked through her own problems there, with the help of psychology she studied there, but she didn't seem to really realize herself and it was also like this was a call, like go do something there, realize yourself, but she herself didn't do that. And it was as if I was expecting that if my parents said something, then they would do it, but that didn't happen, and so I had a question, like, how can this be, like, people say one thing, but do another, And I had such a conflict. Here.
M. Do you have any hobbies? Besides work. A. Well, probably yes, part of it is the field of education, psychology, everything connected with tutoring, with... (...) Drawing, exhibitions, studying, well, I don't know, the work of artists, this is also connected with literature, Let's say now, the main focus of interests is the book (npt), Annie Lee Lang, which describes art, artists, and, in principle, the concept of loneliness. And how artists reflected it, how it is depicted in culture, in what images. Here. Something like that. M. Great.
Well, that is, yes, you will go to medical school, Yes, doctors, doctors, this is always needed, well, they are like that, one of the pillars of society that will always come in handy, you will always need to treat people. Here. But it is very difficult to find, unlearn, firstly, spend a lot. And then, I couldn't answer why I needed this, so somehow, not finding answers, I slowly walked away from it. Well, it's a plus due to the fact that then my mother, grandmother is a pharmacist, my mother is there in the field of obstetrics, and somehow, knowing this whole kitchen, I didn't go there, so this is also an important, it seems to me, such a stage, when I reoriented myself, but it was as if it was unclear where. Further, my interests developed - social studies, history, well, closer and closer and closer I seemed to come to education and the humanities, to the humanitarian sphere.
A. So, training and professional development, I already said that. Health choices. If we talk about the second point, then for the last year, but probably I have had more changes in six months, because there was a change of place of residence and somehow I concentrate more on it. Because the choices that were made in the previous six months were not in favor of my health, but there the only important option, or rather there was a choice, was to go to a psychologist. To deal with this destructive story with the leader, with (sigh) (..) change of position. Here. And if you look more towards the physical rather than the psychological, then this is probably a choice in favor of taking care (.) of some kind about yourself, to searching for, well, understanding in general your health there through the body, changing a little nutrition, attitude towards sports, and so on Further. Well, something like that, I returned there again, to proactive history. Job. (..) One important thing, well, it seemed to happen to itself, it so happened that for a new position, again over the last six months, first there was the building of horizontal communication, despite the fact that there is a manager and subordinates. Yes, I am the director there, but these are still subordinates, a turquoise organization was built. There was this, well, how it was clearly articulated, said internally in the collective, in the team.
M. Basically from the elections. A. Well, after all, what is still very much unclear and difficult for me is the case of accepting responsibility for the whole, the position of a manager. M. Yes, I remember you talked about this in great detail. Yeah, that's what happened. A. This is really very complicated... Well, it seems to have been partially worked out, this situation, in psychotherapy, but not yet fully resolved on some emotional level, it also seems to me that this has created some kind of trauma, (laughter) here. In terms of what was wrong, why was it wrong, right? M. Yes,

why the wrong choice, do you think? A. Because, again, it was probably from the position of - well, who else? Well, very often I have such a choice when there is no other outcome, well, that is, some kind of critical point. And only then do I, as it were, completely regroup, into some kind of monster, make this decision, well, that is, I say this - yes, I can, we'll do it this way, I'll pull it off, but somehow, as if at a critical peak, I take upon myself the whole responsibility that is possible. Well, as if this were directly noticed, and then it usually works out. (xxx) But in this situation, which was and where my choice was, as it were, wrong, I underestimated it. Well, that is, the gap in this critical situation, the gap between what I can do and what definitely won't work out, it, well, happened more. Revaluation. (..) Here. M. Yes, but how do you feel about this situation now? What thoughts or feelings might arise about her? A. Well, it's cool that it was the first thing. Second, an important decision was made that in the next couple of years, I don't know, I won't take a management position at gunpoint. Not yet, well, no, I won't go there again for now. Just now I started, well, I kind of started to sniff out, if possible, from this state when you, well, got a very strong electric shock, well, you just intuitively won't get into it anymore. Here.
Another important choice, which in general, if in the future 10 years, then (..) it's probably closer to the family. Well, that is, as it were, a choice in favor of (..) building long-term relationships, and another related choice - health. Well, that is, as if I don't want to waste what I already have, because our body is only depleted, but I want to somehow fill it up, maintain balance, different areas of life that affect health, ultimately well-being, and the feeling of happiness when you feel good. This is a choice in favor of, well, taking care of yourself in different areas. Well, that is, starting there, I don't know, with some kind of tests, check-ups, regular support for physical fitness, psychological health, such a minimum of some kind of psychological hygiene, then yes, this is directly connected for me with family relationships, because in Ultimately, if you make a choice, then for now I have the feeling that I am not childfree, but I still want some kind of (.) continuation of the family and continuation of the clan, if you can call it that. Again, I want healthy children, a healthy child, and this means that I must be healthy. And the other person must be healthy. Therefore, this is a long-term construction, with certain resulting requirements for a person. (..) And probably not by requirements at all, but by similarity of beliefs. When they look in one direction. Well, probably for 10 years, well, another important one, this is first, in the short term, somewhere up to 5 years, a period of time to take, then this is a set of expertise and experience, packaging it and going out not to work for someone, but for myself.
M. You can go straight through the list, you don't have to arrange them. K. Training and professional development is... Probably there will be seven, because (..) you always have to learn everything, and sometimes it's very difficult to allocate your resources and brains, (laughter) let's put it this way. And do one thing. Health is five, (laughter) it's either there or it's not, work is also six or seven, because... M. Wait, let's go back to health, making specific decisions. In terms of health. K. Regarding my health, everything seems to be fine, I feel good, but sometimes there are times when I get sick, (laughter) Covid and so on. And I try to recover as much as possible. But if I get sick, then I don't do anything, it's immediately minus work, (laughter) minus friendships, only the place of residence will be there then. (laughter) At maximum. M. What about choices, such as choosing which doctor you need to go to, or even understanding whether you need to seek help or not? K. Choosing a doctor, I don't know. I somehow... M. How difficult or easy is it for you? K. No, it's easy for me to choose a doctor. I just see, either from reviews, or using word of mouth, that this doctor, we went to see him, he's good. I'm so good, I'll go to him too and everything will be fine. In principle, this is how it turns out, so I don't have such a difficult choice here to choose a specific doctor. Well, usually, I just probably didn't get sick with anything serious, no matter where it depended on me... The doctor's choice is between life and death. Therefore... M. In general, you said that you rate your health as a five, then what is the difficulty in making decisions

regarding health? K. Health, difficulty making decisions, (laughter) giving injections. This is where it's difficult. (laughter) Or force yourself to go... It's just that when you're sick, you don't really want to go out. M. So it's difficult for you to understand whether you need help or not? K. Yes, I can't, it's sometimes difficult to assess the extent of my illness, it just seems to me that even with a temperature of 38 you can still do something there and at work, everything is a doctor (NPT) in principle (NPT) and you can live.
M. And now I will ask you to tell me about one story of a successful choice, and also, please, in accordance with this layout from the chat. K. Successful... (..) Now, we need to think. (...) Something... (...) I thought, I think it's good that I changed one job for another. Before that, I worked in another place, not even at a university, but in a clinic, well, in a hospital. And this is very, well, at first it seemed to me that this is very cool and great, because the equipment is new, but then I realized that this does not give me any career growth and if I stay in this position, I'll be there (npt). Then, but this also happened at school, then they gave me... Work, a feeling of extreme fatigue, every day. And it's like (npt) Groundhog Day. And I realized that this had to stop somehow, otherwise I would simply burn out at this job. M. So you also seemed to be guided by your emotions? K. (..) Yes. M. It seems to me that you have someone else there? K. Yes, now there are students again, we should have opened another classroom for them. All. (..) Well, it seems like I told you everything. M. No, more about feelings and thoughts? K. Oh, (sigh) feelings, then this situation, (laughter) it seems to me that this whole situation sucked out any emotions and feelings from me at that moment, because working in a hospital is very energy-intensive. And now I'm looking at it too, the same thing, the only thing I've gained is experience, put it in my work book and just to fill out my resume. M. Tell me, Karina, how do you determine which choice is successful for you and which is unsuccessful? How do you separate them? K. (..) Successful, if I feel some kind of return, I feel needed. And unsuccessfully, if I feel some kind of return, I feel needed. And it's unsuccessful if I feel like I'm wasting time and wasting it.
D. It turns out that at the age of 11 I was told that I had the fourth degree of scoliosis, idiopathic, and that this could no longer be treated, perhaps I should have started going to some massages there a couple of years ago, doing something, but I had already encountered with a severe form of this scoliosis, and they told me that... Well, I was again given a choice, I remember how my mother seriously decided to talk to me, at the age of 11, that Dasha, you have such a situation, you can (..) live with this scoliosis, that is, it's like, well, I would have developed a hump, very strongly, it had already begun to appear, or I would have to undergo surgery, and they would put me in a metal structure for life. Into the spine. And when you're 11, you don't really understand that they're going to do something to you now, and you'll have to live with it, well, for the rest of your life, and I made up my mind, and basically, my mother told me that I walked this path very steadfastly , and lay in the ward after the operation. The girls who were 10 years older than me were there, they cried, it was hard for them, and my mother said that I went through it all with courage (laughter). Here. This is probably also an important path. M. Yes, I really sympathize, well, it's really a choice, and it's such a significant one, and I think that you are probably faced with some of its consequences. Does this have anything to do with tennis or not? It seems like... D. My mother thinks yes, that it's because of tennis, but I think that... (..) Perhaps, but there is a reason, there is a reason. Perhaps this somehow prompted, well, aggravated the situation, but obviously it was not the reason. Most likely it's something genetic, perhaps I had crooked feet from birth, and they tried to correct them for me somehow, here. Did not work out. And you understand, yes, that when the weight is unevenly distributed, then all sorts of scoliosis results. That's probably because of this.
Then health. Well, from the last one, probably... But this is probably some kind of funny situation, there's not much choice here, I, I felt bad, not so long ago, a month ago, and I called an ambulance,

I didn’t want to call it for a very long time, because that it always seems to me that my situation is not serious enough to call an ambulance, for someone it is now perhaps more important, more necessary, so. And when we arrived, it turned out that the situation was very serious, I had some kind of intestinal inflammation. And they just gave me a choice - to be hospitalized or not. (laughter) And I, too, somehow without really thinking, decided that it was necessary, let them check me and all that. And it was also an unpleasant situation that I was out of work there for a week, and I was ashamed, but I thought that if this, (..) if I don’t get cured now and then it all somehow piles up, and it will be like like some kind of lump of snow, just like that. Well, in general, some more pain will be added, and then the consequences will be more difficult to deal with than now. I hope I made myself clear. (laughter)
When I went to the hospital, I didn’t immediately tell my parents, only when I felt good there
want to be the kind of person who (..) takes responsibility for all the negative events that happen to him, and for example (..) does not ask, well, that is, it’s normal to ask for help, but when you really need it, that’s it. I (npt) did this, and I started to practice it now, and if I feel bad, I don’t call right away, but before I immediately picked up the phone, immediately dialed and cried. (laughter) And now, first I’ll cry to myself, listen to music, maybe talk with friends about some abstract topics, and then, when I’ve calmed down, I’ll share. Like this.
How could it be, what else? (laughter) (..) I think, I think. (laughter) I still have something from childhood, like, let’s say, principle, choice, I never drank alcohol, well, cigarettes, that’s understandable. And even when I... Well, that’s how it is in Russia, they are always trying to offer something to you, and as if in theory, the majority drinks and considers it normal. (laughter) Yes, and when you don’t drink, in general, they are all so surprised, well, come on, well, try it, (laughter) but I always had a clear motive, this has been since childhood, that I am not going to drink and I will never. Well, as an example. Well, even when, relatively speaking, I was moving into a hostel, the neighbors somehow, well, there are three of us, and they suggested how to celebrate a housewarming, they asked me - will you? I'm like, no, I won't. (laughter) And they were like - well, okay. But I always hear this phrase - you will start someday anyway. That is, I don’t drink at all. Not for the holidays, well, it doesn’t matter. Just never and not at all. Here. This, so to speak, (sigh) is my principle and choice. (laughter) (...) So, I don’t know how many minutes have already passed there. (laughter) It just happens, you don’t remember everything right away. (..) Damn, I guess I, I can’t think of anything yet, it’s hard to come up with, that is, come up with.
M. But you also said that mom changed a little, softened her views. What does this have to do with? L. Oh, (laughter) with her life experience, probably. Well, oh, in general, yes... (..) Well, she looks, reads these psychological books, and something else. In general, it develops itself, so we plant it like that. (...) Because she doesn’t like her life, she wants to change something in it. M. Lisa, was it really noticeable to you that suddenly she changed there and became softer? Or how? L. Yes, yes, she just talks a lot, and since she has no friends, in our city, well, they were, they all went to Tyumen. (laughter) And a new friend appeared, and she also left for Tyumen, (npt) And so it always turned out that she was talking to me and all these topics, well, I don’t know, she talks a lot in general, as if about herself, then about how she lived there (sigh) then she speaks about herself, about her changes. Or it says that you can reprimand me there if you see that I’m going too far there. Well, in general it was not difficult for her. (laughter)
..) Rather, this is how it is, somehow significant, this is health. (...) So, health. (laughter) (...) I would probably classify it more for me as some kind of sport, some kind of sport, (..) they are connected. And, (npt) I also said that alcohol, (laughter) (npt) let’s say, refuse (npt). That is, I don’t want this one (npt) yet, but even before that, this is the last one. And as for sports, I, I have a new kind of sport, that is, always, (NPT) development, I liked it, there was a subscription for eight classes. This is something new for me. But as they say, well, jumping on a trampoline, doing (npt)

or something like that, in my opinion. (npt) Then work. Work, well, work, here I chose, “Yandex.Food”. I was choosing between Yandex.Food and Delivery Club, well, it seemed that in Yandex.Food, well, how to work there, on the contrary (npt), with large orders, but since I could, I had initial information... M. So, now everything is turned on, now, I hope, everything will be heard well and there will be no interruptions. L. Well, yes, I said that I was choosing between Delivery Club and Yandex.Food, and there was more difference, that is, I went to Yandex.Food. Oh, and then I also worked at Pyaterochka, but I initially went to the order area, since it turned out to be there from morning to evening, and I needed (..) evening part-time work, or for a number of hours. But, I said, they offered me a cashier job, and I, I had a choice... M. Yes, Lisa, something went wrong. Yes, about the order picker, and you were offered cashiers. Here in these words. L. Yes, I had a choice whether to agree or refuse. And I thought, okay, I’ll agree, I’ll try, why not. Although it was a little scary and exciting, in the end everything, so to speak, worked out, I was trained and I worked calmly, so to speak. Here. Later, but then I had to leave, so I quit. But I warned that I wouldn’t be around for long and, in principle, (laughter) since they didn’t quite suit me, (laughter) and it would have been a choice in general, (laughter) probably to quit. And, so, (npt) in my opinion, (..) I would roughly give an example, like choosing a vacation. Since it’s difficult for me to get from Rostov to Nizhnevartovsk, so to speak, at the moment, since the airport is closed, and you can get there by roundabout routes, but it’s expensive. Because from Moscow (laughter) to us one way it’s 10 thousand, but I just had a direct flight, I could fly (npt) when I was lucky in principle to get it and when you already flew for 3, and for 5, and for 7, then you don’t want this, well, yes, you don’t want to go one way for 10. But in principle, it happens, it’s a lot, so to speak, well, I would come to my city, but there’s not much there, there is nothing to do, and in general, my mother and I agreed that we would meet in the summer, but not in the city, but somewhere, so to speak, for us on neutral territory. Well, we went to Sochi, that is, she (npt) flew, and I had to get there by train, and... (...) Well, we generally chose tickets, well, where is the best place to go, we chose these ones, where Sochi is. But since I was already there, a year ago, for a few days, it was somehow very, there is such an example, but... (sigh) So, (..) friendly relations. (...) Well, choice. (..) Well, here, probably, who would you like to be a friend, (laughter) and who would not. Don't know. Or, let’s say, where there is a person like this, I would like someone... (sigh) (...) So... (laughter) In general, they offered me to take a walk there, and I, (..) let’s say, I didn't want it in general. I don’t like this person, so I don’t want him to be (laughter) my friend. And you probably... Well, I kind of refuse. Don't want. Don't know. (laughter) What’s a better example to give, honestly. Well, I don’t know how I could have one friend here, (..) I don’t even know. I'm moving on for now. Place of residence, regarding the place of residence, by the way, I live in a dormitory, and initially I didn’t want to live in a dormitory, because, well, it seems to me that here, well, all sorts of events are held, as if there was a place to sit, let’s say I had to film apartment or something like that. And plus I have a hostel, it’s very comfortable, well, I just won a competition, (laughter) and the hostel in which I live, it’s like, well, it’s an apartment type, and that’s why (laughter) it’s here for me in general, not only that , that I’m like this in a hostel, where I have connections, but it’s also almost home. (laughter) But, of course, I didn’t know which one I would end up in, but initially I wanted to go to the hostel, so that there would be all sorts of these events in which I could participate. That is, renting in one there, in an apartment, is not interesting, it is boring. Here. (...) Everyday and important decisions. (sigh) (..) Well, about important decisions, everyday ones, probably just like where, where to go, if everyday, where to go, to have fun, to see. Well, also, for example, I took part in the race there in May, for me it was such an important decision. In such a major event, because it is not held here, and I have never participated. The Russian Federation race, which took place in May, I... Well, it costs money, and you kind of have

to decide on it. (laughter) I took the plunge, bought it and didn't regret it. And also (..) I liked it, I finished the race (npt) I also decided, I realized that I liked it, that (laughter) I need to train for it, and that, well, more.
M. Yes, but do you have any decisions in life that you consider unsuccessful? L. Solutions... Health related? M. In general, not only related to health, but are there any choices that you consider to be completely unsuccessful? L. Yes. The elections were unsuccessful. Well, (..) probably yes, yes. (laughter) But for me everything is connected with sports. (laughter) In the eighth grade, no, not even in the eighth, but earlier, in the sixth grade, they offered me to go to... (npt) To the All-Russian Olympiad for schoolchildren in physical education, but I refused then, and in the seventh they offered me, but I She also refused, or rather, at first she agreed, then for some reason she refused. In the eighth, (sigh) I also refused, and in the ninth, (laughter) and only in the tenth I came, and even then I agreed. And... I regret that I refused before, because I really wanted to go to the final stage, that is, to Russia, which took place then in the eleventh grade, in Kursk, that is, you go through the regional, well, first the school, then municipal, regional, and final. And (laughter) in general, two years of preparation is not enough, and if in the tenth grade I failed there, then in the eleventh, in the region, although I was a prize-winner, I took first place, but I was 17 hundredths short of Russia, and I was very I wanted to. And in general, I regretted then that I had not agreed to another school earlier. And in general, yes, and the fact that in addition to physical education there, let's say I would also take part in mathematics, I somehow even thought about it... Well, I didn't think about what the Olympiads are like, and what they give, and what in general is that? In general, then it was a bad choice that I refused. (sigh) In this regard. M. So you consider those elections unsuccessful, where you missed some opportunity? L. Well, probably yes. M. But out of these several situations with physical education Olympiads, which of these, well, which of these situations is the most unfortunate, in your opinion? L. (...) Well, what's the worst? (laughter) The situation, well, (laughter) I didn't have enough points, (laughter) for Russia, these hundredths... Hundredths, well, it's just literally the whole point, or (npt) in gymnastics, or a second of running. (laughter) M. And you feel like you're blaming yourself a little for not pushing through? L. Yes. I just wanted, and even more than to pass the Unified State Exam well, that is, (laughter) well, damn it, I still can't come to terms with it. It's a shame. (laughter)
So I realized that as a result, you have not yet enrolled in any specific business specialty in order to do quests. But as for sports, why wouldn't it become some kind of professional field, well, in which you would become, well, a professional athlete, why not this? L. Yes, it's interesting, I mean, in general it's, well, firstly, we don't have that many sports in our city, and if it's all over there (npt), then basically, in principle, then in our regions... Well, even if not only all kinds of sports, Moscow has everything, I recently made such conclusions, but in the regions, in one there is no such thing, in another there is no such thing, in a third this is not the case, and since we have a lot of sports, I started somewhere with basketball, then I liked it, before I was banned anymore (npt), then I went to sports tourism for 2 years, it's not like It's just mountaineering, but that's the point, there's a program there, on the ropes like that. In principle, I liked it, but for some reason I got tired of it. I don't know. (laughter) Honestly, I got tired of it and just quit. (laughter) The same about athletics. And athletics, it's even more boring. Well, again, it's a cyclical sport, where you only do the same thing, and that is, we only have running and nothing else. There is no throwing or jumping, just running. And I'm not interested in running. (laughter) It's the same thing, in general, I'm truly not an athlete. I understand this because, well, I don't like it. Well, I don't know, and somehow other sports, I didn't try much, then I just somehow in the ninth, or even the eighth grade, (laughter) forgot that I was an athlete, and to some extent a sport was absent from my life, and then, in the tenth grade, I somehow remembered this, and here, when we were just preparing for the Olympiad in physical education, we have, in addition to, well, theory, gymnastics and

running. And since I liked gymnastics, it's clear that it's not like real, professional, sports gymnasts, there's a lot of other things, and I (laughter) by the way, I regretted that damn, it's a pity I don't do this, but they only take from childhood. And my mother told me that she somehow brought me when I was 5 years old, but they refused me. But I don't remember this, they kind of told me that I was too big. (laughter) But I kind of liked it, and because of this I kept looking in Rostov for some kind of acrobatics for adults, gymnastics for adults, that's it. And trampoline jumping, but it is combined with gymnastics for adults. But the problem is that damn, it takes a day. (laughter) Well, yes, sort of. And so, well, I didn't manage to go to any professional sport, because I couldn't find one for myself. M. That is, if, perhaps, when you were younger, you would have been sent, well, sent somewhere... L. Yes, perhaps.
Z. Yes, yes, yes. Here. Choice of lifestyle and leisure. I really want (laughter) for this area of activity to change for me, because over the past year, all I did was study. True, I don't have time for anything else, I didn't have time. I'm trying to change, I'm trying to change this moment, I really want to do... Well, in general, I would like to do yoga, go to group classes, I realized that I really like it when (..) probably a new team appears, and there are new people, meeting new people, it's very interesting, it's something new and unusual. I would really like this area of mine to improve, because again, all I'm doing right now is learning. And this drains you and you need to recharge somewhere. Where can I get it? Well, I realized for myself that I would like to go to a slightly different space, to other people, and replenish my resources with the help of sports, yoga, etc.
M. Okay, let's now take a closer look at your elections, and now I want to ask about the elections that you may consider unsuccessful in your life, were there any? And why do you consider them unsuccessful? Z. (...) Yes, it was a bad choice in terms of health, so I already told you that I turned to a surgeon, and... Well, I regretted it. I had an operation that was not required at all, that is, in vain, they completely removed my nail plate just like that. Why this was done is unclear. When I came to a highly qualified specialist, a podiatrist, they told me that this was a complete disgrace, it was impossible to do this, and now we are correcting this whole problem. Here. That's why for me this is such an important choice, a health experience. And now I think this is very important. When they wish you health, now I don't just, well, health, happiness. Happiness, health - yes, this is what I need. (laughter)
M. Great, great. Yes, it seems we have now discussed some better choice. Maybe you can remember, for example, how a bad choice was made? For example, with an ingrown toenail, maybe you can remember your thoughts and feelings while you were going for surgery? Z. Yes, yes, of course. At that time, I was at home, not in Rostov, and we went to the hospital. In general, it turned out that I was immediately sent to a surgeon to solve this problem. I come to the surgeon and he looks at me slightly and immediately says - that's it, operate. I'm like - what? And I had never had any health problems before. Everything was fine, no surgeries at all. Well, it was a shock for me, I was admitted to the hospital right away, that's it, they left me, my parents brought my things, and the next day I was waiting for the operation, so. So it turns out (..) day X comes, the time approaches, they take me to the operating room, perform the operation, and the worst thing was that after the operation on the nail, well, it turns out that the nail plate was completely removed, and a bandage was wrapped around this, on this skin. It's very tender and soft there, and it's still a little unhealed, because an inflammatory process began from this nail, it was painful. Here. And they wrap this bandage around me and say, well, that's it, now lie down and rest. Everything is fine. The next day it was necessary to do (..) soak the nail in a special solution so that it, the nail... The finger in the solution so that it heals. Here. And for this it was necessary to remove this bandage. I start to unwind it, but it won't come off. He was completely stuck to this

super soft, unhealed spot, it was just terrible. The time was probably just right... (..) Well, there was a day, I was sitting, I tried to soak this bandage in different solutions, a couple of hours passed, it didn’t help, we just started trying, well, tearing it off a little, that didn’t work either helped. It was terrible. We probably sat like that until very late at night, with the nurses, they tried to take it off for me, nothing worked, it hurts, it’s a super soft, tender place... In the end they told me, let’s do this to you. In general, well, it’s like a bandage, it’s even, applied to this finger, here. They cut off the entire part of the bandage and left only the piece that was glued to my finger. And in general, they tried to remove it with tweezers, pulling out each one of these... (laughter) A piece of lint from the bandage. It was terrible. It's just a nightmare. This also didn’t help, and I’m already in tears, of course, all upset, nothing is working out... I’m also a very suspicious person, (laughter) I’ve already thought of a bunch of things, that’s it, now he’s dried up for life, I’m like with him I’ll live, (laughter) upset, that’s it. Well, they told me to wait for the doctor in the morning. But that’s all that was left to do, and I ended up sleeping through the night with this bandage, and the next morning the surgeon came and in the operating room he made me some kind of, I don’t know, glued some kind of plate on me, and just tore it off with a very sharp movement. Yes, from this soft place, they simply tore off the completely dried bandage. It was very painful, oh well, and it was all bearable, it all went away, that’s it. I thought that was it, my problem would end there, thank God, everything was cured, everything is fine. So six months pass, the nail grows back, it grows very ugly, ugly, really, ugly, yellow, clumsy, all like this... I turn to the surgeon again, already here in Rostov, he says that Well, we need to do an operation and rip it out again. I think - what kind of person are you, really? Here. I’m thinking, well, are there any more humane methods of treatment, maybe it’s possible to even it out somehow? They tell me that such treatment is done only in private clinics, this is a paid treatment, but they said - try it, maybe it will suit you. And I thought that I didn’t want to suffer anymore, I wanted to know something specific from a specialist, some exact answer as to why this was happening, not just to rip it off every time, but to find out the reason. And what to do with it. And I turned to a podiatrist, in a private clinic. And now this doctor and I are still aligning my nail plate, everything is fine. Here. Of course, I was upset that I had experienced all this, but probably without this I would not have understood that it is very important to go to, well, really good doctors and it is better to pay a little for your treatment, and be sure that they will really help you and correct you your problem, than every time, as they suggested to me, tearing off a nail. Here. M. What did you think, what did you learn... What do you think, what did this situation possibly teach you? Z. (..) Taught? Well, probably after all (..) until this moment I didn’t really think about the qualifications of doctors, that is, you come to the hospital, doctors, they’re all doctors, they probably know everything, smart people. And now, you look at it from a slightly different angle, well, at least I do. I pay attention to certificates, I pay attention to where I studied, what merits I have. Well, in general, for qualifications and I think this is still very important. And also thank you that we live in the modern world, we have the Internet, we have the opportunity to look at people’s reviews, at a certain doctor, for example, or a clinic. And now, depending on the reviews, you can make a decision whether or not to go to this specialist. Here. Probably, after all, yes. The situation taught me to take health more seriously, this is not a joke, and to pay attention to who will treat you. This is also very important.
Then I went to school, and in the first grade I did synchronized swimming. All first grade. And then, the workload became so heavy that my mother suggested that I either change schools, or that I study in the second shift and be able to devote all the remaining time to training. Or stay in school and quit swimming. And she didn’t decide this for me, she said that it was entirely my choice. And then, during that period, I decided that I would quit swimming. And I'll stay in school. Well, it seems to me that then I chose friends and some kind of social life, it seems to me, at the age I was, as if friends were more important, and sports had never been some kind of meaning of life for me. I

went, I basically liked it, but he was never a priority for me. But now I think that... Now I don't think at all, but I used to think that if I had stayed, I would now be some kind of master of sports or take part in competitions, be an athlete. And life would have turned out completely differently. But nevertheless, I never regret the decisions I made, in any way. I studied the same way, in the same school from first to eleventh grade. (..) I was like that, not very much, but that is, not a completely withdrawn child, but also not one who directly craves attention, that is, I was afraid of public speaking, but at the same time I communicated well with everyone, well, I was the kind of person who He seems to communicate with everyone, but only a few people are good there. But everyone treated me well. Then, the elections, where... In principle, even when I was in school, my parents always put the whole choice on me, they didn't decide anything for me. Which sections should I go to? I also tried a lot of different sports, only because I wanted it myself, and I left the same way because I wanted it myself. And it seems to me that this... Well, to some extent it's probably correct, perhaps those who... Those parents who want their child to actively engage in some kind of sport, who have their own ambitions, they somehow insist, but I, on the other hand, have tried a lot of different sports throughout my life, and I realized what I like to do. I still swim, for example, like it was with synchronized swimming, I realized that I like swimming, I still go to the pool. And when I started doing athletics, I realized that I like to run, but kind of (..) not... But I only like to run, for example. I don't like everything else we did. And I just left, just doing it for myself. Well, that is, it seems to me that we need to somehow give the child a choice, although they already asked me then... (...) What... (...)
M. If in terms of the degree of difficulty of the choice, how would you rate it between one and ten? Well, you actually talked about a series of elections, but on average, how do you evaluate your passage of these forks? You chose between a bachelor's degree and a bachelor's degree, this advanced one, you chose not to work there, but you chose to work there. J. It seems to me, seven, approximately. M. Thank you. J. Health, I don't even know what I can say about this. Because I've always tried somehow... (..) Well, I just have very good health, some kind of good immunity, I've never really... In terms of paying some attention to my health, I make a choice in favor of some kind of regular check-up. I go to doctors, donate blood, take vitamins, and exercise. I'm trying. Sometimes I get very lazy, but I'm trying to instill this habit in myself; in fact, I still really want it to be some kind of regular. Also, I go to the doctor, well, that is, I don't have the position where I will never go to the doctor in my life, like some people, they don't like it at all, no, I'm somehow very sensitive to myself, to my health. It's better to go get checked there, once again, than not. M. And how would you rate the severity of choosing these, developing habits, even laziness, but it's important to go to the doctors, get checked, get tested, how easy or difficult is this for you? G. Four. M. Four yeah. Thank you. J. (..) And work, for me it's a little bit like that, for now... (..) Well, I'm still studying there in general, what suits me, what doesn't, I don't understand yet, I generally... At the fact that it seemed to me that I was a rather diligent person and that I could somehow do some monotonous work for a long time, but it turned out that not as long as I thought. And I'm still feeling out in general what type of activity I'm comfortable doing for a long time, because somehow, well, in my head it's as if there should be one activity for, well, a long period of time. But I always somehow get tired after a couple of months, I need some kind of change of activity, or some kind of development. That is, right in one place, something is the same, like accounting, for example, or in a bakery, doing something the same every day, I start to get tired and fall into some kind of despondency, which is like this it will happen, I immediately have some thoughts that it will be like this all my life. It's the same there every day. That is, I also realized for myself that in this regard I need some kind of variety, and I want... Well, my dad, for example, never worked in an office, maybe that's why I subconsciously somehow chose a profession similar

to his . Because he travels around objects all the time, doing something, something like this is closer to me, some kind of active activity. Even in terms of studying, I could never sit like this and constantly listen to something and that’s it, that is, when we receive knowledge, we immediately put it into practice, some kind of more creative training or something. And apparently I’m inclined to have a job, I also need some kind of work, well, more active or creative, I don’t know. I'm still searching. Choosing a job is actually quite easy for me, I don’t know, maybe (..) 6... I somehow start easily, finish easily. (...) Well, six, probably not, let's give five. Let's put it at five.
J. Because it’s such a difficult topic, because I still... (..) Well, that is, I still live, for example, with my mom and dad, I communicate every day, I’m still building some kind of relationship with them. I myself go to a psychologist, somehow I’m already trying, from the side of some more... Well, not a teenager anymore, but some more adult person, I’m trying to look at any situations from both sides. Both from my position and from the position of my parents. Before that, at school, I always had some kind of very blaming position. That is, I thought that the way I think is exactly like that, and I never really wanted to consider any other points of view on this situation. Not how I see it from my side, but how other people could see it. Well, my parents. And that’s why it’s always been very difficult for me to understand them. And I was like that, a rebellious child inside, that is, on the outside I was quite calm, but I always disagreed with them, with something, I always somehow kept my opinion about you. And now I’m just trying to take it easier, it’s easier to accept that someone may have a different opinion and that’s normal. Other views on some situations. I don’t know, I don’t understand at all what choice means in terms of relationships with parents, but (..) in any case, it was always somehow very difficult for me, I... We always had good and bad, then good again. It was such a thorny road.
M. I think that this decision is also very difficult, and somehow very worthy. But you speak warmly about your father, and about your similarity with him, in principle, in part. J. Well, yes. Well, I went, as soon as I had the opportunity, I immediately went to a psychologist. And I still go to him. We have a free psychologist at our faculty. They did it for us. This turned out to be a very popular service. A lot of guys go. And this is really very cool. Yes, I’m working on my relationship with both my dad and my mom, how it all affected me, and where in this whole situation I am, and where are my parents’ opinions about me, (npt).
Somehow it’s always been (...) hard for me to open up. Yes, it’s probably some kind of, well, childhood trauma. I’m still working with this, with a psychologist, and it’s hard for me to open up to people, because it’s as if... (...) It’s as if, when I open up, I start to take everything too close to my heart, and then it’s very difficult I'm experiencing this. Therefore, in terms of friendly relations, choosing some new friend for me, well, probably an eight.
Now, I have some kind of frantic energy, I want to meet someone all the time, learn something new, go somewhere new, to theaters, museums, concerts, walks, etc. whatever. I feel like I'm being filled up by this, I don't know. So I went, when I went to a psychologist, somehow after that I became more open, and then people themselves began to reach out, after that. (..) Oh, romantic relationships, that’s also a ten. I have a very hard time with this, because it’s also about opening up, you have to open up to people, and at some point I start to quickly, quickly close myself off, as if when I understand that this is some kind of... Well, it’s not like that anymore, (..) when I realize that I am becoming a little vulnerable, I, (..) my brain seems to automatically begin to push away, that is, it is so much so that I even (..) at first may not notice it, and only when I analyze it later, that is, everything is so quirky that it seems to me at the moment when this happens that the reason that I chose is really important, but in fact then I understand that I just had some kind of fear and (..) I just, well, sort of decided to run away. Now I’m actively working with this, but this one is hard. For ten.

K. Metaphorical associative cards are a tool for a psychologist so that a person associates his situations with the help of pictures. That is, it's easier to sort of reveal it all, from different sides, from different points of view.
M. Did you decide to do this yourself, or... How did this idea come about to do this? K. So I was on an educational shift here, at the Republican Center. And just like that, the psychologist worked with us, and she talked about these cards, which are associative psychological. I bought them because I was interested in all this, I bought them and they are generally so beautiful, (laughter) and I began to sort of rummage within myself, with the help of them, and then I realized that I was more interested in going deeper into all of this. climb, and this just became popular, these Tarot cards and so on. And I think - why not? I bought them. And so it went from there. And astrology is always interesting, because from childhood it's like, oh, who is he according to his zodiac sign, do we match with him or not? (laughter) Something like this.
Well, firstly, I didn't want to lie, I never had a need to lie or hide something, and (.) secondly, it's always obvious from me if I'm lying. Since I apparently didn't learn this, my mother always knew that something was wrong, even if I kept silent about something. She says - so, do you want to tell me something? She always (xxxx) saw and read me. Right always. Well, at the age of 12 I had a rebellion, at 13, maybe it lasted until I was 15, and then it started that I don't want to tell my mother this, she will scold me there or she forbade me to do this How can I tell her about this? After this, this situation, which lasted maybe a year or a year and a half, with that boy, no more situations like this were repeated, that is, this was enough for me, I completely lost all her trust, it didn't suit me, I stopped feeling this connection , so that I needed it, that is, she stopped believing me, stopped supporting me, it became hard for me. And this was not a reason for me to stop communicating with him, I just matured a little and realized that he really was something strange. What did I even find in him? (laughter) Here. And then I realized that I still regretted what I did. And from that moment on, I no longer deceive in any way. (.) And that is, my mother and I discussed this, I say, like it happens, my girlfriends drink, and she says - okay, like if they drink, then wait a little, from 16 I will officially allow you, come on, (. .) whatever... (..) Only you will warn me, write and, as it were, you must come home, you don't have to go anywhere, and so on. Well, okay, that is, there were no secrets from my mother at all.
M. That is, from the point of view of even some health issues, you usually consulted with your mother. K. Yes, of course.
Now I'm asking myself what I want. I also recently found such a method, with a coin, that is, what should I do, do this or that, and instead of going up to my mother or asking my friends, I take a coin and if I'm not satisfied with the answer, it means that I've already decided what I want. For some reason I'm just afraid of getting it out of myself, I don't know why this happens, but I realized that what happens most often in these cases is that I already knew the answer. Here. It's very interesting, so I say, I watch myself, and I like that I'm growing. In terms of personality, in terms of the fact that it is separation from parents, this worries me first of all. Here.
I immediately signed up for the dances, as if to continue, because I liked it there and the team that was there, so I came and then stayed. That is, I thought that for an hour and a half twice a week, nothing would come of it, I would remain in shape, I mean physically, that's it. And in general, studying...
D. And I, you know... Yes, I have friends. I have very close girlfriends, and my boyfriends are also close. I also decided to take care of my mental health, since I can't go to a psychologist, since we have one free psychologist in the city, and I went, I didn't like it, because it was at the level of simply abstracting from problems. Here. That is, sometimes it helps, yes, so as not to overextend yourself, but sometimes not. Here. That's why my friend gave me a book for New Year... (..) "The

child in you must find a home.” This is some German psychologist, Stephanie Stahl, here. There are so many exercises, why we do this, that is, in general, it all comes from childhood, and inside us there is a child who... Ah, a gloomy child and a sunny child. And all our tantrums, or when we get angry, it’s all a gloomy child, that’s it. And we need to find a common language with him, and we also have an inner adult who sometimes succumbs to the whims of this gloomy child. Here. And there are a lot of interesting exercises there. I try to do this sometimes.
M. Yes, in general, undoubtedly. Are your relationships with your parents too distant, distant? Or somehow you quarrel, or... D. Sometimes they are very close, and most often these are just situations when we become close, which is extremely rare now, they make me feel guilty that I’m so bad and I offend them. But in general, well, I don’t know, they certainly didn’t do much harm to me, but purely morally, they did. That is, there is some kind of financial support at all. (..) But psychologically, of course, it’s very (.) difficult. (..) Here. Varya, I'm a little busy. They are shorter in the kitchen. OK. In short... (..) So what should I tell you? M. I asked about relationships, about support or about conflicts, well, a little, in general terms, in order to somehow understand. D. My father drinks often, and as if before it didn’t directly affect him very much, it was limited to the fact that we were walking along the streets, he was drunk, and just did all sorts of bullshit, I was ashamed of him. And then, in the ninth grade, when I began preparing for the Olympics, I studied them seriously for the first time, I almost didn’t leave the room, because I wanted to seriously prepare. And my parents said that you sit so much and seem to be moving away from us, well, they fought because of this. And my dad also came home drunk, and my mother asked me to sit with him in the kitchen and talk every evening, he drove me to hysterics, and in ninth grade I can’t even imagine how I pulled it out, but it was... Emotionally it was difficult . Here. And with my mother, well, I don’t know, in general I’ve always been my father’s daughter, well, I thought so, but for some reason I don’t have a particularly good relationship with my dad now either. Mom sometimes, sometimes she somehow tries, like, when she sees that I’m really really bad, and that’s not always the case. Here. But she doesn't take my problems seriously. Well, yeah, she doesn't take my problems seriously. Yes, they don't take my problems seriously. That is, if I say - I’m tired, I worked, they’re like - why are you tired, what problems can you have at 18 years old? Yes, it’s true that there are none. Here. Well, in short, not being perceived as an adult also depresses me. Here. (...) So, it’s very difficult now all at once, in fact, the relationship with parents is quite complicated, that’s it. And now I’ve simply protected myself from them. When they are at home, I practically don’t leave my room. Now my mother is offended by me, and I gave up, I don’t care what she was offended by, I don’t even want to find out, let her really be offended, if she doesn’t want to enter into dialogue, it’s her problem. Somewhere in the middle of the tenth grade, I tried with her, no, or at the beginning of the tenth grade, I tried to talk and say that I need support, at least just come and say that you’re great, you can handle it, you’ll succeed. And that's it, I don't need anything else. And this dialogue boiled down to the fact that I’m ungrateful, they spend a lot of money on me, they spend almost their last money on me, and I’m ungrateful. Then I got sick of them saying that they were spending a lot of money on me, so in April I went to work. That is, school and work were certainly difficult, but I managed. And since April, I haven’t asked them for any money, I’ve bought clothes and something to eat here, well, I’ve gone to the neighboring town, all with my own money. And then they say something like this to me - why don’t you go to the village with us, like you’re at work again, you’re cutting yourself off from us. Are we not giving you enough money? Something like that. Well, well, yes, I don’t have enough money. So I went to work. Here. (..) In general, they are (..) children. M. Children? D. I can honestly say, they are not trying to change themselves, they are not trying to hear (..) me, that’s it. It's quite difficult. M. Yes, I’m very sorry, what you’re saying is very sad to listen to. And it’s even so paradoxical, that is, well, it’s clear that you’re trying very hard and learning, and it’s true, a lot of things are working out, and so, when

they tell you that you’re studying too much, and maybe you need to stop and go do something else, yes, I think it’s very disappointing to hear. D. Yes, and when I wrote the Olympiad poorly and walked around upset, they told me that I just didn’t study enough. Here. Something like this... M. Well, yes, in such conditions, of course, it is difficult to get support. Apparently we really need to look for it somewhere else. Please tell me, do you like being so independent, do you want to be more independent, or do you want to share this responsibility with someone? D. I began to notice that... Apparently I don’t know at what level this works, but here... (..) somewhere inside I am still a child and in most situations, sometimes stressful, I begin to behave like a child. And then I, well, kind of think about it and... Well, I analyze it, and I understand that (.) did not behave like an adult in this situation. But it was necessary. Here. Well, in short, this independence from childhood, it actually affects me. Because even more so sports, they also tried like professional sports, all the work, and there, well (..) no study, no personal life, only sports. In short, this is it. You also need to be responsible. And now sometimes it takes its toll. Here.
M. What kind of sport? D. Orienteering. M. Wow. D. I also took up skiing. M. You can do a lot of things, it seems. D. Yes. M. Does this sport experience help you now? Even though you left him forcedly. D. Yes, it helps in the sense that... Well, on the one hand, no, I mean, wait, does it help that I left him, or the fact that I was studying? M. No, no, what did you learn exactly, what were you there and what did you do? D. What I endure until the last moment is a minus. So, let’s say I get sick, and I won’t go anywhere until the last minute, I’ll endure it. I’m working until the end, I’m already feeling bad, I’ll still do something, because I need to go to the gym... Or I just went to the gym recently, and I’m thinking like this - I’ll do a light workout, because after my illness. But in the end I did it very hard, because somewhere in the middle of the workout I thought that I had worked very little, and that I had not finished myself off, and I need to finish myself off more, that’s it. Well, (..) no, sport is just that, I don’t know, I made friends because of sport. Here. (...) And in general, orienteering is a cool sport, it makes you think. And in general, the fact that you are running through the forest, I love this sport, in fact, very much, with my soul. Well, but as a hobby, not as a sport. Sport, it destroys people. Professional. And health, both mental and physical. Everything possible.
And then, in short, school ended, I went to the school playground for a month, then on July 1, my grandmother and I went to the dentist, that is, also with my grandmother.
And on July 2, they left me alone. And in short, I went for a walk with my friend from the entrance. I really loved swinging on the horizontal bar, letting go and grabbing. In general, I once thought - like, how long, how long can I stay in the air without getting caught? Well, I fell on my face. And in general, it was a very cool day, so eventful, I rode in an ambulance, and then I probably walked around for a very long time with a blue face. Well, that’s how my independent life began. (laughter) M. Yeah, what do you think this story says about you? How does this story characterize you? How do you think? How do you think about her and about you now? D. I don’t know, it’s just funny to me. M. But then it probably hurt a lot? D. No, by the way, I don’t remember being in pain at all, I just remember that I fell and lay there for a while, then I thought that I needed to get up, and I felt discomfort in the area of my face, I wiped it and saw blood on my hands. I think I’ll go home and wash myself. And in our case, in short, then they were repairing the roof, and there were guys standing at the entrance, they saw me and they lifted me up to the fourth floor, called an ambulance, and called my mother. Now, if they weren’t there, what would have happened? Or if these men turned out to be inadequate, what would have happened? Interest Ask. But everything turned out well actually, no concussions, nothing, I didn’t break my nose. M. Yes, but what did the parents and grandmother say when they came home and found out all this? D. Well, grandma was at home, mom came running, scared of course. Some guys called her and said

that your daughter is covered in blood, that's it. M. Nightmare. And on July 3rd they left you alone again? D. I don't remember anymore. Maybe. Mom works, dad works, and of course. Well, sometimes my grandmother went to the garden, probably... I don't remember. But I'm still living, so it's okay. (laughter)
And, by the way, I started going to doctors alone very early. Here. At first it was very scary, very unpleasant, we have a very inadequate woman at the site, a local pediatrician, and before she could bring me straight to tears, so. Now I just come with a straight face, immediately dissatisfied and angry, so that she feels my bad aura (laughter) and does not start putting pressure on me. (laughter) In short, I begin to put pressure on her first. Here. And everything seems to be fine. Of course, it's still sometimes scary to go to doctors, I don't really like hospitals, that's why. Well, yes. As I already said, sometimes I endure until the last minute and don't go when I need to, on time, that's it. This is of course a minus.
M. Did you end up in the hospital because of this? D. (..) No. But... Well, yes, I was at Sirius this summer, and in short I was slightly ill, I had a sore throat and a runny nose. Well, I didn't go right away, but I went to the first-aid post, they prescribed antibiotics, I took it for 4 days, that's all, I didn't go to the first-aid post and then say that everything was fine with me. I just didn't want to go there anymore, that's it. And in the end, after 2 days I felt very bad, I could not tilt my head, my sinuses hurt, everything was wrong, I was afraid that I had sinusitis. I had sinusitis, that's it. And I decided to go. They scolded me very much there, they said that this was impossible, they took me to the hospital, in the end they wanted to admit me, but everything worked out, and in the end I was on antibiotics for another two weeks. That is, half of the entire shift that I was there, I was on antibiotics. Now I have stomach problems because of this. And all because I didn't go and tell him again that everything didn't work out for me. Here.
M. This is the responsibility for the elections, right? D. Yes, yes. I am not responsible for my health at all. I don't know, I somehow feel very bad about my health. (npt)
M. Is someone else watching him, do they remind you? Or, well, just... D. Well, they remind me, but they don't make sure that I'm taken right by the hand to the hospital, because after all, I'm already 17 years old, I'm not a child. Here. And even more so, there was such a situation that since childhood I've been in hospitals, I don't know, I've had a lot of different things, I also have tuberculosis, some kind of problem, that is, that I have this mantu and Diaskintest are always big, something was stupidly infected there or something like that. She was registered. Then a cardiac surgeon, oh, a surgeon, (laughter) we also went to a cardiologist, I had problems when I started playing sports normally, in the fourth grade. Then, when I started playing sports even better, in the seventh grade, I walked like a disabled person because of my knees, and we also went to another city. Well, in short, a lot of things. And this is not a complete list. That's why I'm very tired of this, I don't really like going to the doctors and wouldn't like to at all, but somehow I wasn't particularly lucky with my health, to be honest.
M. I see. Are there some situations that you want to hide, on purpose and in general so that they don't find out and maybe no one finds out at all? D. (.) Probably self-harm. M. Do you do self-harm? D. (.) Not now, but through sports I can. Come to the gym and just... (..) To the point where I'm just falling. Here. M. And does this help you in any way? D. Well, (.) not to think about something, I don't know. Just to finish myself off, probably. I don't know how to come home, go to bed and that's it. M. It's a pity, this is of course an inappropriate way, but as you say, it seems to me that you seem to have more different available tools in order to somehow deal with your condition. She showed me the book, told me about the exercises, and told me about her friends. D. Well, I'm trying, I'm trying to somehow get myself out of all this, in fact, I'm trying.
M. Thank you very much. Please tell me, you are in Rostov-on-Don now, right? And I understand that you are working, while we were agreeing, you wrote that you need to catch up after work, are

you studying or just working, how is your life in general now? L. Yes, then I will answer in such a multi-layered way, I received my first education this year, a bachelor’s degree, at DSTU University. In the Don State. Here. That's where I work. Here. It’s like we’ve even gone a little towards individualization, here. I’m studying, I’ve now entered two master’s programs, the first is my 39th, this is social work as well, and the second, 44/2, is in psychology and pedagogy. M. Ah, colleague that is. It's clear. Is the bachelor's degree also in psychology? L. Bachelor's degree 39, this is social work. M. Social work. L. Yes. I became interested in psychology in the second year, in the third, the therapeutic direction was closer, I studied there and realized that I also wanted to take two master’s degrees and one of them first. M. So you will study in both at once? L. Yes. That's what I thought... M. Wow. And work a little more? L. Yes, and in different universities. Well, that is, in my 35th, also in DSTU, and I went to study psychology at the Southern Federal. M. So, the Southern Federal is Krasnodar? L. No, this is the Southern Federal District, this is also Rostov. M. Oh, I got it mixed up, yes. It's clear. Do you have any other hobbies or interests, if you have time for them? L. From a hobby, if it’s an activity, yes, I really like to organize and come up with all sorts of different thematic events, fortunately, the university also loves when it is offered to do something, I probably won’t name any right now, psychological hobbies, hobbies, but for me I like some practices, well, for which I am conditionally admitted, I mean going through, conducting training, of course that’s it. I wouldn’t call my work a hobby, although I enjoy it.
M. Can you think of, remember some example, from there, I don’t know, from a movie, from a TV series, for example, I don’t know, from another work, from a book, of a person who is independent and not independent? Maybe some celebrities, someone famous, or some bloggers, someone in plain sight? L. So, (...) I don’t know directly whether this can be such an example, that is, interpret... (..) And in the work, well, Bukowski, there is Henry Chanaski, as his alter ego, the work “ Women" is called, "Post Office" too, so, I remember the main character, in my opinion he is very infantile, he also lives the life that he wanted there, he is rather an example of this, he works there at the post office, with a huge number of sexual relations, beer, and it’s as if he has nothing else in life. He didn’t escape anywhere, and he didn’t have to do anything. And if he’s just so independent... (...) What’s his name, I watched some interview today, I don’t know, I remember, Mark Guarden, I think his name is. Something like that. March... Some psychologist, I listened to his podcast today. Some kind of person, I don’t know... (...) I can give examples, so I look, I don’t know, the rector at my university is an independent person, but I don’t know (npt) work or not.
M. Did something happen then? L. Yes, yes, yes. And there... She fixed herself in this position. M. Well, somehow you can ask the question - what? Or don't want to answer you? L. It’s possible, it’s possible. I worked through my injuries, you can ask. (laughter) In my family, well, like in a family, there was a stepfather, a case of domestic violence that lasted for 6 years, from 10 to 16. Here. And it was very difficult to fight back, I was little, then I grew a little, so. And at the final point, well, at the age of 16, I immediately understood that I had to do something, somehow fight for myself, well, yes. And, thank God, it worked out, as they say. Yes. And at that moment I immediately realized that oh, oh, oh how you have to be independent (xxxx). (laughter) Oh, oh, oh, how necessary this is. And right then it was clearly formulated and it didn’t go back from that point, thank God. M. But you moved after 3 years, as far as I understand? L. Have you moved? M. No? L. But no, just, well, it turns out that I have my dad, wonderful, beloved, but we (npt) when I was 7. Then I had a stepfather, from 10 to 16, not a very good person, (laughter) Here. Now my mother is also married, but now I have a wonderful stepfather, I respect him very much, I love him, I appreciate him, he is kind, caring and, well, my dad too. Wonderful person. I don’t know what happened to that person. And I already moved when my mother was... I already had, now it turns out, a current stepfather, I already moved away from them. I didn’t have it there, but everything

was there (npt) it all ended just then (..) then, my last trial was at 16 with him. That's all. So we (..) said goodbye and... And I directly cemented my position as an independent lady. M. I'm very sorry about domestic violence, of course, and I'm glad that you and your mother are no longer there. If I hear correctly. L. No. M. And you came up with a plan? L. I'm now... Now I'm (npt) taking pictures of the experience, it was yes, thank you, I might not have been so strong. Who knows.
And after sixteen, yes, the first independent decision I made, I remember, it was on nerves, consciously, I don't know why, why, it didn't happen, I lit a cigarette. I came straight away, and I didn't have a situation where I was wandering around somewhere, in garages, I came openly and said, like this. Here. Naturally, my mother didn't like this, of course, but due to the traumatic experience there, she somehow turned a blind eye to it then. There she said - I'm sure it will fall off you, but now, okay. Well, we'll sort of assume that these aren't some kind of steam locomotives, it's not necessary. She is the only, it seems to me, the wisest thing that she told me, she says - please, you can, just don't buy some crap for yourself there, it's better to let them lie there at home, if you want, take it, like. Well, this is something normal. Because again, she says - what's the point, well, I would take it away from you, shout at you, so that you would stop smoking? Nothing would change. I would (npt) where is it, well, I would be hiding somewhere. No, and it fell off. I don't remember, I went there for two years, I worked through the traumatic experience with a psychologist, and one day I just said, no, I don't need this anymore. I wasn't so drawn to it when it was like this - well, you want it, right? Please. How (npt) is it normal, let's not just somewhere, over there at home on the balcony, you want, well, that's it, so that I can be calm.
M. That is, after you, I don't even know how to describe it, when you broke off relations with this man who showed domestic violence, with your stepfather, when he disappeared from the family, as I understand it, or he was imprisoned, I don't know. You had some kind of surge in all sorts of independent decisions and some kind of activity, right? L. Yes, well, yes, but rather it was probably, I don't even know how to classify it. Well, because yes, then it turns out that for 6 years you seem to live in a social cage anyway, there you don't think about how you can choose or think about something, but I don't know how it is conventionally, what jacket to wear today, so that there are no visible bruises. Anyway, this stupid childish trait - I won't show it, it lived, here. And this was the only thing in which you made (laughter) any independent decisions. Previously, you already go into some kind of, it seems to me, even aggression, in relation to those decisions in which you were limited, and you are already trying to grab everything that you couldn't take there before. Well, here I need to give credit, again, probably to my mother, but she somehow directed it all correctly, what are these, well, that is, my kind of eccentric, some kind of emotional outbursts, I don't know, or what - radical decisions, like, I don't know, smoking the same thing, were not met with aggression on her part, which would make me even more willing to do something against. She manipulated it all so much that you were like, so what. And that's all there is to it, and that's okay. Yes, well, thank you, okay. Fine. Fine. Well, then, but I remember, yes, she gave... Well, the start date, oh, now, as if I didn't tear off my charger. I'll put it in. But before the start of, yes, my first year, it turns out that the first year and everything negative fell away from me.
M. Are there some things that you prefer to hide from your parents, maybe you don't tell them from everyone? You mentioned tattoos, but that was quite a long time ago. L. Yes, well, what can I say, now I wouldn't hide it, now I would come proudly (npt), to my mother and say - here it is, new. I just don't want to. I guess sometimes I can hide some of my emotional anxieties from my mother, because in general I'm such an emotional person, I can be sad, or just emotional... The only thing is, I try really hard, but I never succeed straight hide it from my mother, when I catch a cold there, or something like that, in short, it happens, I know that she will be nervous, and I don't like it so much, and I'm always trying to do something, somehow - yes, everything is fine , Fine. (laughter) These are things, yes, I try not to tell my parents, because they will worry, well, that is, I

don’t know, ordinary ARVI, but mom already - oh God, you live there alone, who will give you tea? do? Now, well, that is, these are not some kind of terrible things, I just (npt) don’t want (npt) only dad. No one will lose anything from this, I just don’t know, they will save 1000 nerve cells by doing this (npt). Without thinking about the tea I made for myself or not.

3.7 Table 7. Therapeutic networks members and their functions

**Citations**
Yes, it turns out that after that I trained for three more years, I also went to different competitions, in different cities, I already understood, well, since I refused that chance, that chance, a second one most likely won’t come to me, but I’ve already trained, let’s say So, semi-professionally, I played for our academy, CSKA, but I (.) didn’t dream of going there to some Wimbledon and so on. And one day, when I went to a competition in Taganrog, I was fighting for first place, the last one turned out, the last game was for first place, and I fell on my back, so. I injured my back, then I couldn’t walk at all for a week, I was thinking that it was a fracture, or that it was a bruise. They took a picture and said that it was just a spinal bruise, well, it would go away soon, but after that I already quit tennis, because even after rehabilitation there, about six months, when I returned to tennis, the indicators were completely different and, accordingly, , guys who were plus or minus my age, they continued to increase the pace while I was rehabilitating there and so on. Well, basically, I was, roughly speaking, no longer needed, even in this, at this level of tennis, and I decided to leave.
Yeah, health. Until I was 17 years old, well, I was an athlete, and so on, and so for a very long time, well, until I was 17 years old, I always told myself that there is no smoking, no drinking, I will never be there, it’s not for me, especially for me Dad smokes there, I think no. But then, at the age of 17, after (.) the death of my friend, my second, I started smoking. I still smoke to this day, I reacted at first... I still have thoughts, why did I do it? Maybe it's worth quitting? But then I think that, well, for now this, for example, brings me pleasure and some kind of outlet, in its own way, so I think for now I’ll make a choice in this direction, and while it develops in this way, well, I’ll drink there, I don’t know, there’s a glass of wine on holidays, roughly speaking. That is, not a fan. The choice to start smoking was conscious; I purposefully wanted to try smoking and start smoking. That is, I don’t know how to describe it, one day I think - I want and will smoke, and that’s it. And I kind of tried it, and that’s what I wanted. M. It worked. M. Yes. (laughter) That's how it all happened. Therefore, the choice was also probably easy, I probably don’t really regret this choice, well, although sometimes, perhaps some thoughts slip through there again, but in terms of complexity this choice was, I also think there were about three or four, probably maybe, something like that.
As a result, I smoked from the second to the fourth grade, that is, I had been smoking for almost 2 years, being generally a schoolboy who had his head on the table (npt) himself because he was running. But it turned out that I had to quit for two reasons. First, and most importantly, health problems began. I’m sitting there, this is all for me, I’ve played enough, I won’t.
And in the end, the actions reached the point where the teachers all scolded me for a very long time, and were about to label me as, well, like, not a street child, but whatever it is, well, in short, register as a child. Here. But there it turned out to be somehow simple, I was simply put on the internal school register. And they said that if something happens again, then we will have the same horror. A little time passed, a week later the fight started again and then they started beating me, probably about seven people beat me, on the street, it was in winter. But at some point, I kind of

just lay there angry, I don’t know, something confused me, I abruptly shoved everyone who was standing, fought off with steps, quickly jumped up, kicked someone else there and caught up with one, and just I took the snow that was lying, and then there was some kind of ice and I decided, I thought that I would just wash his face with snow, it so happened that I took a piece of ice and it accidentally hit him in the temple. Nothing terrible happened there, the ice just cut the skin a little, that is, not even a cut, it was so strong. But in the end, I was still scared too, that’s it, I won’t go to school anymore. Let's go to some doctor and say that blah blah blah, let's check the child, I'm there, I don't remember exactly what happened. Well, in short, the doctors there determined that there was some kind of prolonged depression, they prescribed the child, well, fifth grade, antidepressants, and they forced him to sit on antidepressants. I spent a long time on them, on the course, I don’t really know, me, well, not systematically, but at times, my mother seemed to say to them - let’s continue to drink this. And these were, I really don’t remember, either some very mild tranquilizers, or antidepressants, I remember... They were probably called something, I’ve already forgotten. But the point was that my studies failed because of this, I just stood at the blackboard like a vegetable. I didn’t have a single thought in my head and the teachers often looked sideways at me, and to themselves, that is, like this drug addict, to the narcologist... What’s going on there. Carefully. I'm sorry, the girl dropped something. Here. We went to a narcologist, where he told me that, buddy, everything is fine, they showed him a prescription for these antidepressants, and for a long time they refused. But somewhere around the eighth grade, I realized that there was a huge difference in interaction between me and my classmates, that is, more and more of the time that I sat at my desk, I simply watched how my classmates’ adolescence was passing. And at some point, like in some “Rocky,” I stood up in my room and said - I won’t drink, that’s it. I refused. And somewhere from the eighth to the ninth and eleventh grades, I began a time of real rehabilitation, when I, well, there my first interactions began, just like those who were more mature. Here. There's a story with friends, one there, others, the fourth, it still didn't work out with the girls, that's it. But somewhere around the tenth or eleventh grade, I was already completely rehabilitated, both as if just on my own, and in the eyes of my peers, who saw me both in the sixth and seventh grade. And we kind of fell into the same sort of path, that is, the same groups, and on our own we seemed to communicate normally. And this, this dynasty that I carried with me from the bad events that were covered by my pills, somehow left. Then it so happened that even by my first year it all continued; I began to experience a simply stormy, simply huge, stormy life as a freshman. And I slept, it was so that I slept at night every other night, one night. That is, I worked in the morning, afternoon and evening on projects at the university, at night we went to parties, the next day I still worked in the morning, afternoon and evening, and at night I slept. There were nights when I didn’t sleep, not because there were parties, but because I didn’t have time to complete projects by the deadline, and I also had to work at night, because, of course, there were some situations, they just calculated it wrong , one of the teammates did not fulfill his duties there, and so on. Here. And I, as the leader of the project, just needed it to be completed. No matter what a person does, I will try for him now. Here. And my classmates, whom I later met, joked that I was speedrunning my life. Speedrun, this is true, just in case, for those who will decipher this later, this means accelerated, accelerated passage. That is, among the players there are those who compete to see who can complete the game faster. This is called speedrun. I was told that I was in the speedrun of my life. That is, in terms of (..) everything that they felt there during the entire time when they studied there from the fifth to the eleventh grade, I felt literally in 3 years, and even further, what they had not yet had time to experience, I already felt it. That is, I was carried away somewhere there.

But I met one woman, I think she was 27 years old at that time, and I was 19. I was just turning 19. Who fell in love with me, and she is a psychologist by training. Clinical psychologist. There was

already a master's degree and so on, she worked in a children's school, I don't know, in a children's school of additional education, as a teacher of robotics, and at the same time as a psychologist.
M. Ilya, very detailed, thank you. In general, I'll start with your first phrase, when you started speaking, you said that reflection is always welcome. And as if from your story, it also came up a couple of times that you have, as it were, experience communicating with different psychologists. But tell me, tell me, are you, in general, inclined to reflect on your own, or how did this experience influence you, why are you talking about yourself in such detail, analytically? I. It's like two in one. Like these non-cafés. But it turned out like, the story itself from the 8th grade there, when I came off these antidepressants, well, it turned out that in order to understand this, it was necessary, of course, to reflect there. I have minimal communication with a psychologist, well, except for this psychologist I just talked about, 27 years old. Because every time I went to a psychologist, it ended, well, there were one, two, three or four trips. And they told me what I already know. And most of the psychologists that I just went to said, "I don't need to go to a psychologist, I calmly delve into my own head." That is, I will scatter if I can. The only times when I actually went to a psychologist with benefit was when I went for IQ tests. This was when I went, I sat there for an hour with him, he solved the test, then yes, this was my only useful trip. And the rest, if they didn't just interfere, somehow changed life for the worse. Although it is also unclear which one, good or bad. Probably a good one after all. Then the rest were useless. Well, it probably turned out that I myself am a reflective person, I even think at the Skolkovo university the moderators told me that at times I was even hyper-reflective, let me look for meaning where there is simply none. (..) But it so happened that the element in which we learn, it implies reflection after the end of each activity, project. Necessarily. That is, we have to sort everything out, what, why, but they didn't teach it in a very good way. But when I practiced with the Skolkovo team in game technology, a prerequisite there was, as it were, reflection itself, game technology, and in detail, that is, I had to write a plan, with the tools that I used, that is, I said what I want to try on the guys there such and such, such and such a method, there is interaction between them, maybe there (npt) according to Weldon a couple of people there can move from an idea generator to an executor, and the like. And then come back and write a reflection. It didn't work out because like this, like this, like this. You need to try this, maybe something like this. So much so that I needed to read the reflections of those guys whom I sort of moderate myself. Therefore, it's like I'm reflective myself, and it so happened that I also needed to practice this intensively.
M. But I'll immediately clarify this episode, when you already felt that you, well, smoking began to affect your health, and now your parents burned you, this moment, in general, you wanted your parents to burn you in these activities, or did you not care at all? I. On the contrary, I wanted them not to burn me, (laughter) to receive this strongly, and then for them to know, in principle. Yes, it's just that my father, in my earlier time, probably around the sixth grade, had big problems with alcohol, very big ones. (..) And there it got to the point where I was just sitting, he told me - look, cigarettes, smoke the whole pack there, so that you can get more, so that you feel that this is not the same, and don't smoke there anymore. Well, the like. Well, basically, since I was in school, my father probably doesn't drink anymore. That is, I don't know how many years have passed there, maybe even 7 years for sure. (..) Yes, in general, it's been more than that, probably almost 10 years or something, since my father doesn't even drink anything strong. Well, similar. He didn't even seem to imply that smoking a pack of cigarettes would just be like, well, a pleasure. Here. But no, I didn't want my parents to burn me at all, it happened by accident, a classmate burned me, that is, she complained. Oh, I forgot to specify, so that my parents wouldn't burn me, I sprayed cologne into my mouth. It was an old children's cologne, I don't remember, it was unlikely that it was triple aged, because it was a regular children's cologne. But it was definitely alcohol-based, and I even

remember it was “Pirates of the Caribbean”, then these colognes were also coming out. And I just poured cologne into my mouth. Here. It was terrible, but what can you do, you had to make some sacrifices.

M. And now there are those moments on which you consult with your parents? I. (sigh) (h) (...) Well, probably for health reasons, I always consult with my mother, because she is a nurse. Here. That is, if I suddenly start to get sick there somehow, in a way that I have never treated myself before, that is, if I start some kind of ARVI or something there, I can basically cope with it myself. Here. And when it comes to some symptoms that I haven’t had before, I call my mother and talk, if they don’t help, then I go straight to the doctor. Here. (..)

M. I hear some kind of conflict between what you think and how you act. And how is it generally given to you, is it easy to make a choice in such a situation or not? That is, why do the scales still start to tip in one direction every time? M. (sigh) It's hard to say. Probably because... (..) How to say... Well, again, probably there is a point in psychology that people often treat others the way they want to be treated. And among other things, I give people, other people, a chance because I would like to be given a chance one day if I stumbled. This is exactly why I give people there some kind of warmth and so on, that is, everything, all these moments, because I myself lack it. That is, what happens is, let’s say, the opposite situation. That is, I give what I lack. Here. M. But this is generally interesting. How did you come to such an understanding, to such an experience? And to your own behavior? M. I unconsciously stumbled upon all sorts of psychological things a lot. (NPT) I unknowingly came across all sorts of psychological things, I don’t know, because you scroll through your VK feed and there, for example, there are some posts about psychology, something else, in the same TikTok you come across a lot of all sorts of psychological analyzes, oh behavioral types of a person and so on. So it all stuck to me like that and, in principle, some kind of awareness came. And that includes still coming. Here. And so... M. So, that is, you are not a naive respondent, right? And pumped up.

M. Well, yes, it seems that you hear yourself very well and understand what you need and what your needs are. Tell me, with your activities, what you called a hobby, did you also listen to yourself or perhaps it was imposed by your parents? M. Regarding sports, I probably always liked to do something like that, to feel some kind of power over my body, that is, when you grow, including over yourself. Including remembering, as they say, (laughter) the classics of fight club, development through self-destruction, that’s also a wonderful thing. This is also wrong, but I probably came to this too.

M. The choice of a doctor, the choice to seek medical help or not. M. (..) (h) Probably (..) (laughter) two or three. Here. Because, in principle, I practically don’t get sick, I’m a follower of a very strange method, the one that does everything on its own, but there is such a joke that a man goes to the doctor only when a piece of a spear in his back prevents him from sleeping. On the back. Here. Therefore... I probably have a lot of them, by the way, yes, it will probably be important to record that I have a lot of things taken from my personal stereotypes that I have formed of what a man should be. A lot of things come from this too, that is, I literally have a list of men there, too, to be like this, like this, like that. That is, well, I understand that stereotypes about men, in principle, stereotypes are a very disastrous story, but I myself, here is my list, (laughter) I should be like this. That's how it is in this regard. This also affects a lot of things in my life, so... Yes, regarding health, in principle I probably don’t really value my health, so it’s easy for me to make decisions in this regard. Here. I kind of understand that I do sports there and so on, all the things, but at the same time I can drink there calmly, well, I try to know my limits and so on. It’s as if I understand that if I drink a lot, then (laughter) I will die in one moment.

M. (laughter) Yes. Here. Therefore, I probably don’t particularly value my health, and making decisions here is quite simple for me, I still try to control my health and so on, in the sense that,

well, I try to see something in myself if something hurts me and so on, but otherwise, it’s as if I’ve never had such a thing that I have to go straight to the doctor, that is, it seems to me that I’m more than healthy. I recently took tests, and they told me that they could at least launch me into space.
Health. Health is the area that suffers greatly in my life, I’m sick right now, right now. And I recently took a Covid test there, yesterday, and I’m waiting to see whether it will happen or not, but it seems that it won’t, I already feel fine. But, damn it, I haven’t been doing much about my health lately, but I just support it with sports and that’s it. But I would like to undergo some kind of examination, the fact is that the right side of my head has been hurting all my life, and it’s such a noise, it’s just as if, I can describe this pain as a noise. I've already gotten used to it, because I know that damn it, this never happens, once in the tenth grade I did an examination that did not give any intelligible results, they told me that I was healthy, I'm into it I don't really believe it. (laughter) Here. Still, something is wrong. Otherwise, health...
Health - ten, it’s very difficult to make a choice, I have some kind of masculine fear, I don’t know, or in general it’s a universal fear of going somewhere, or arranging an appointment with a doctor, going there, I don’t know, to the dentist once every six months. Well, in general, it’s just some ten, and I don’t know what’s stopping me.
Next, health. (..) Yeah, health, health. Here, you know, there is a rather difficult aspect, because for probably several years my health has fluctuated, (laughter) let’s say, like the exchange rate of the ruble on the stock exchange. Probably until 9-10 grade, I was quite (.) a chubby boy. And I probably made the choice in favor of a healthy lifestyle on the basis of some, let’s say, personal oppression and personal motivations. But for the most part this was due to teenage conflicts. And, accordingly... (..) Any problems. M. Did someone offend you? I didn’t quite understand about oppression. A. Let's just say, (..) at some point it began to seem to me that at school, on the part of friends, on the part of teachers, at some sharp moment the attitude towards me changed. That is, they stopped noticing me and (.) paying any attention to me. Although I didn't seem to do anything wrong. Nevertheless, I didn’t really understand what happened, but at that moment it was probably quite difficult for me to communicate with people, because I didn’t understand what this attitude was connected with. That is, if I had a teacher with whom I was in close contact, and (.) with whom, in principle, I was conducting some kind of project activity, then at that moment it happened, I don’t know how some day came and as if at the snap of a finger What happened was that this person’s entire attention switched to another audience. And essentially, you know, it's like a child who has been sharply deprived of attention. You want it, but they don’t give it to you. And I probably began at that moment to look for disadvantages in myself and in my appearance, in my health. I began to think that this was probably connected with some characteristics of my body or characteristics of my thoughts. And then I probably started soul-searching. (.) Therefore, the choice in favor of health, it was probably given to me, let’s say, through a battle, due to some experiences, through an attempt to prove to myself and many others that everything could be different, that I can do something... something else. Therefore, in terms of severity, probably (..) well, let it be a ten. At work... M. So you connected this deterioration in your relationship with the fact that you somehow gained weight, or somehow didn’t look the way it seemed right, right? Because you were talking about the love for sweets, and about being chubby, you said? Or did I misunderstand you? A. Well, for the most part, I’ve been pretty chubby since childhood, and I probably stayed that way until the 9th grade. Therefore, purely physically, I didn’t change much, due to weight gain or loss. There was simply, let’s say, one consistency of the body, which was maintained as it grew. Therefore, I don’t know, I probably connected this not with an increase or change in weight, but with the fact that at some point some skills and shortcomings that people did not want to take into account simply became more noticeable. M. Did you say something to you, did you somehow become

yourself, did you say about choosing a healthy lifestyle? Something like this. A. Yes, I decided that... The Unified State Exam had just ended... the Unified State Exam, in the 9th grade, I thought that since such a situation was happening, I needed to rethink myself, I needed to somehow start taking care of myself, to show myself, well, to change something in myself, not only mentally, but also physically, so I probably made the choice in favor of a healthy lifestyle, in terms of athletics. That is, I got up at about 5 in the morning, slammed the doors, everyone was scared of what was happening so early in the morning, and I just left the house and started running at the stadium, ran to some park and spent my time there.
really don’t like this, so I’m trying to gradually return to my usual way of life. And somehow connect sports to your activities. But for the most part, probably due to my (..) fear of people (xxx), some kind of tightness, I don’t always succeed. That is, I cannot, relatively speaking, sit in a hostel, do something, get up and start doing push-ups there. Or, I don’t know, stand in the plank. Because, (.) you know, this is an internal experience when you look stupid in someone’s eyes...
M. I see. How do you help your friends? Well, in fact, were there any similar interesting situations? A. I don’t remember them so often, but most often these are situations related to a psychological and moral point of view, because they fall into some kind of depression, something bad happened to them, I try to support them, I remember sitting at night , talked to them, tried to support them, although he himself took the test in the morning. It was so. Here.
A. (..) Well, in general, in order to get straight on where to go, they told me banal things about don’t drink, don’t smoke, love your mother and everything like that. But the smaller parts, somehow I don’t even remember this, but it’s already on my mind, that is, not to forget our neighbors, those who are nearby, to help them not to forget. Here. These are the things. And if we talk specifically about independence, then somehow... Well, I don’t even remember, they just said that you will grow up, you will be independent, you will do everything for yourself, cook soups and all that. Here. Well, they just taught me and I remembered it, I put it into practice and then I cook borscht for myself now. Here. M. Is this specifically about borscht, about this kind of food, or about some other things too? A. Well, besides this, yes, for example, hammering the same nail into the wall is understandable, my father taught me this too, it was also not without bruised fingers. Here. So... Besides this there is a lot of other things.
T. And I remember myself probably from the age of one... Well, probably from the age of six, from five, somewhere like that, directly consciously, so to speak. Well, as a child, I initially wanted to become... (..) To become a policeman, that is, at that time, a policeman, and, in principle, my parents did the same, if we talk about independence, that is, they always signed me up somewhere, to some clubs, some sections, that is, since childhood I was kind of fond of sports, that is, I was such an activist, that is, both at school and in some extracurricular activities, social events, that is, always for the most part, they attracted me, too, and (..) it seems so, and some leadership qualities were probably emerging, so this is where the roots probably go, maybe some of my independence in making some decisions. That is, as I have already said, the sphere, so to speak, of my activity is quite wide, that is, starting from the public... That is, this has been going on since childhood, that is, now I, let’s say at work, am the chairman of the council of young scientists specialists, these leadership qualities that have been going on since childhood also manifested themselves. And I finished school, that is, until the eleventh grade, passed the exam, and entered the university. At the university, of course, it’s also a little less, since after school, for some reason, I began to devote more... More time to studying, not in the periphery, but specifically to studying, and therefore, a little less, I had classes that were, so to speak, excellent from studies. But still, no matter how I gave it up, I also took part in all sporting events and social events. So I studied and studied, and naturally, after that, after that, I went to work. And at work, from the first, as they say, days, that is, he showed himself as a fairly active person, went out with his colleagues somewhere, that is, he

was always, so to speak, easy-going. And, that is, after that the management noticed and, as they say, I am still, so to speak, in plain sight. That is, just like the leadership, as it was before, like at the university, like at school, and now, let’s say, the strategy can be action, that is, just independence, leadership qualities, and so on, then are present at the moment. Here. In principle, I think, more or less answered. (laughter)
T. Well, I have this character trait: sometimes I follow the majority. That is, in order, as they say, to go against the flow, namely, my closest circle. That is, my environment was quite like this, just sports, that is, we were engaged in dancing, and naturally this was vocals. I was, I looked at other people, I looked at how they were, whether they were achieving success in this regard, and then I tried it on myself, whether I was even interested in the same activities that were popular at that time, and I already made a decision. And if, so to speak, in percentage terms, they probably have their own opinion and people’s opinion, well, in general, the opinion of the current, the opinion of society, so to speak, is probably about 50/50. Well, maybe 60/40, in my favor. In this plan.
M. In general, well, now it turns out that you are discussing with someone some things that may be difficult for you, well, be it some psychologist, I don’t know, a close person? T. Well, yes, there were moments, that is, when I visited a psychologist, that is, in principle, there were moments when I was just put in a leadership position, and I, having worked for only 2 years, yes, about two years , one and a half, in general at work, that is, I was put in a leadership position, naturally, there was stress. In general, there was a lot of stress and I needed some kind of psychological help, (..) it turns out to be qualified. Yes, I also consulted a psychologist. And in principle, this gave its micro-fruits, that is, well, perhaps, including the fact that I have already gotten used to it, that is, this so-called adaptation period has passed, and now I am more or less in a normal, so to speak, psychological rut and everything is fine.
M. And if we talk about health, then when you make decisions in this area, what helps you do this, for example, a consultation with a doctor or how do you even choose a dentist? T. Well, it turns out that in this regard I’m probably suspicious about my health, that is, if some kind of sore pops up, I start there, well, not exactly panicking, but it’s faster, faster to take some action to remove this pain. Just with teeth, and everyone knows that this is a rather expensive procedure, namely dental treatment. And therefore, finances did not quite allow, or rather allowed, but there were other, well, one might say, demanding, that is, aspects that required an urgent investment of money. And that’s why I put off just about the teeth. Here. And therefore, in principle, since I have already said that I am suspicious, I quickly, quickly tried to fix all this as quickly as possible, so as not to start all this. Then don’t pour in even more finances, already eliminating the consequences of all this. M. But I’m even asking more about how you understand who is a good dentist there and who is bad, about this. So, where do you get this? T. Well, here again, already through acquaintances, that is, I found out from people who treated there, and about prices, and so on. There was no such thing here that, yeah, I typed dentists there into Google, and went to the first link that came up, and immediately went there. That is, naturally, at first there was some (..) research, so to speak, on this matter, that is, I found out from friends there who was doing good work and, accordingly, I went there.
T. Well, yes, that is, before, I’ll interrupt, I’m sorry, I just had a problem before in that sense, that I, well, when you see a cat trying to attack a mouse, that is, it first prepares itself there, sits for a long time, waits, and at some point pounces. I’ve always had this in anticipation and preparation, just before communicating with a psychologist and so on, that is, I’ve always had this for a very long time, that is, I’m trying to calculate every little thing that is impossible to calculate, and sometimes just you miss your goals. This is exactly what is needed, it seems to me, we simply mean to wait, but not to wait out in general, roughly speaking. That is, to evaluate yes, more or less seriously, (npt) you act as you see fit. Here. Something like this.

I. So health... What elections, elections, elections, elections, elections, well, it seems to me that with my health pah-pah-pah, everything is fine, I try not to get sick, (laughter) here. It seems to me, well, if we take health in general, well, my mother just had a stroke there. I had to make certain choices there, I don't know, about apartments, about inheritance, and so on and so forth. This all also falls on me, in any case, so for me, well, it was hard for me, it's still hard for me to resolve all these issues, because in many areas, with many of these things, I'm meeting for the first time, there the insurance company, the hospital, I don't know, they have to process certain documents, it's incredibly difficult for me. As a child, my health was bad. As a child, after childhood bronchitis, I had very severe bronchial asthma, I was sick for a very long time. I rarely, I didn't go to kindergarten at all, and I went to school, but I was sick very often, of course. (..) It was very difficult for me as a child, so it seems to me that I would rate my health as exactly this, I would rate it as an eight
M1. What was prohibited, damn it, no such thing actually happened. That is, I don't know, well, it was forbidden to steal, yes, to do something else, but no, even some kind of morality, alcohol and cigarettes, we just don't seem to discuss these topics, in general, there was no such thing as it's impossible, smoking is harmful, I don't seem to know, through conversations maybe, through some other information, I realized that it's kind of harmful and I didn't do it, about cigarettes, never in my life, that alcohol is just something to try, understand that that's it, that's all. Well. We just didn't talk about this topic, that's all. It's like I didn't study, I kind of understood that they wouldn't be very happy if I smoked and so on, I kind of thought, why do I need this? Well, plus, I'm very frugal, and I understand that cigarettes are a huge waste of money, and so is alcohol, so why bother with it? Oh, I'll give you an example, here's one pack of incomprehensible cigarettes, then let's say you can buy a certain amount of sweets with this money. I'm like, candy, it's cool. That is, it's like, well, everything is measured by equivalents, even this, as I was taught financial literacy, that - mom, mom, I want Kinder, I want Kinder. Mom tells me that look, it costs so many rubles, but you can go to the store and buy so many candies by weight. I'm like, wow, let's have better candy. And I sort of understood that.
M1. Oh, I'll start now from the earliest, it's just that my mother has such a perception of life that she was against kindergarten, due to negative experiences in childhood, that she remembers by her example that teachers are very like that, not so much, and may not monitor the health and so on of the child. And she always sent me to some private development centers, and if you take me from a very early age, there are speech therapists with me, reading, something else, that is, in addition to the fact that my mother worked with me, that she is like me in fact, she taught me everything, before that at school, I went to such educational programs, and when I became more conscious, as an adult, probably at 4-5 years old or something, well, at 4-5 probably, yes, at 5 somewhere, I went to the children's center, combined with a fitness center for adults, and there were all sorts of sections like judo, fencing, and all sorts of other things, that is, I kind of went, it just sounds cool, interesting, some kind of dancing, gymnastics, acrobatics, absolutely went different. Accordingly, later, when I became even older, my range of interests was outlined, these are creativity and sports. Here. As a sport I went to judo most of my life, then 2 years to boxing, 2 years to karate. And I probably practiced judo for 10 years, or 12, I don't remember. I graduated from music school with honors, so in terms of interests, you can say, well, little by little, let's say take a swimming pool, creativity is a guitar, some kind of drawing, singing in different sections, that is, I actually did drawing in many different sections. I won't say that it was some kind of cool level, but just purely for myself, to communicate, to spend time, it was. M. And at school, it turns out that you also managed to study very well, in addition to these sections? M1. Yes, I can say that I, this is not my assessment, this is the assessment of others, that I am smart, and I had an interesting situation, that I understood that I could easily be an excellent student, I am not a fool, I can prepare, I can do

everything, but I was just kind of lazy to spend so much time, and this is probably not very good, but I never had problems at school, any difficult subjects, the most difficult subject for me is chemistry. Somehow, out of interest, I just took it and decided, I'd better learn the topic, and then I didn't study it anymore, this topic, because it wasn't interesting to me, that is, I understood that I was doing things that were more useful for myself . Accordingly, I had no problems at school; if you look at the boys, I was among the smartest, that is, in terms of grades, and everything was fine. And this despite the fact that my gymnasium is one of the best schools in my city, where studying is quite difficult.
That is, my schedule is like this: at school I communicate with them, after school I go to the music room, there I communicate with these people, and from the music room, let's say I go to sports, there I communicate with these people. And this is how it went cyclically for me. That is, I communicated with many, but I won't say that all of them were my very good friends, they just communicated well, well, in some respects they were friends, naturally, in each section I had one or two people with whom I was most communicated, I think these are adequate things. Well, something like this.
M. But about the karate trainer, tell us how he helped you develop independence, since you speak about him so warmly and in detail. M1. Yes, I speak very warmly about him, about the coach, because, well, this overlaps with the fact that I spent a lot of time in training, a lot of time, from the tenth grade I did karate, it got so bad that I had, let's say Monday, Wednesday, Friday, two workouts a day, 2 hours each, and the remaining time, let's say one. Each training session lasts somewhere for 2-3 hours and I was with him all the time, like listening to how much he loved to share some stories, how he not only enlightened us, but simply talked about all sorts of life topics, I heard him talk about it, I drew some conclusions for myself, what I can do, how I can do, similar things. Here. In general, just in my opinion, even in terms of education with a child, the main thing is to talk to him stupidly. Just talk. Tell some moments, life situations, how he got out of these situations, so that the child has some kind of template example that he could use in the event of some new situation. And then impose your own, having some kind of behavior model. That is, it seems to me that it is from conversations that a certain model of behavior is formed so that the child can use it and accept it as a given, in order to develop it later. He can completely redo this pattern of behavior, in case of some extreme situation he won't have to think, but what should I do, he will remember - yeah, my dad did this, or my mom did this, you can do it like this. He will do this, and then he will think, what is best for me? He will do what is best for him. That is, just in terms of conversations, all this is being formed. That is, the coach said that in reality martial arts are very connected with personal perception, and you cannot teach martial arts if you just come, roughly speaking, once a week to training and just somehow teach the technique. He said that all the masters, they lived with the teacher and seemed to watch him, how he behaved in the natural environment. They seemed to adopt the image, likeness, and mind of the teacher, how he behaves in a normal environment, outside of training, outside of sports. And this is the difference between a coach and a teacher. I can call my coach a teacher. For example, I can't call a judo coach a teacher, because his task was to make athletes out of us, well, he made athletes out of us, he is a professional in this matter, but there were no such life conversations. Because that was not his task. My karate coach had a different task. He talked to us a lot. Accordingly, well, somehow he is still just a teacher by training, I'll tell you what, at the university, so he knows what to talk about and how to teach. So it was very interesting and educational. And it seems to me that it was karate that revealed a certain personality in me, in terms of absence, removed some edges, that I can live calmly, socially, not be afraid to meet someone, do something else, because I don't know, how it happened, but I'm sure it was after karate, because martial arts associated with a certain kind of

combat, they greatly change the worldview, perception, and seem to remove some kind of framework, boundaries. You become more confident. And quite independent. You could say. Because you are not afraid to defend your point of view in some dispute, knowing, like, that if someone comes at you, you will be able to give him an answer and give him a very good answer. M1. About the karate teacher and dash teacher, I was wondering if he has an education, you said that he teaches at a university, but what does he teach? M1. He is the dean of the physical education department of our Ryazan State University. M. How did you even get into his section? M1. This is also a very interesting story, I... (..) I just somehow saw how training was going on, in karate, in a completely different place, in a completely separate place, in the Krasnodar region, when I was with my grandparents in away, I saw how the training was going on. I was so impressed by this, because at that moment I was doing boxing, and I lacked some kind of morality, some kind of cultural core, when before training everyone bowed, sat down, closed their eyes, well, not just prayed, but they just sat down as if getting ready for training. After training, a bow, ritual phrases, when there is movement and intelligence, not just a straight punch, a side kick, but some beautiful Japanese names, Japanese language, culture, kimono, that’s it, equipment, I really liked it, it went in, and I I started looking for this section in my city, I found a section in my city. It was another coach there, who reported, in fact, to the head coach, whom I spoke of as a teacher, I went to him first, but then this coach said, that there was an opportunity to go to some training camp, go to another building, with this head coach, so I went and somehow it happened that the coach liked me and he decided that I would work with him in the training camp team, national team . And I began to study with him. And I spent, in about six months, actually, I actually studied for six months, I went along the path to a black belt, in fact, because I worked a lot, plowed, studied, learned new things. This is my story of meeting this karate trainer. Now we don’t communicate, but I remember him very often, that is, in some conversations, and I am very glad that such a person appeared on my life’s path.
M. Listen, but about dependence on someone, you say that you don’t like to be dependent on someone, but why, that is, what do you put into this dependence? M1. Well, I had an offer from my coach to become a personal student, in Japanese traditions, as it were, that is, roughly speaking, to live with him, that is, to do what he does... As if he essentially becomes a certain , not exactly a master, but kind of dictates to you what you need to do in order to develop. Yes, I would become an athlete, but again I understand that I might not become if I had some kind of injury. That is, I did not want to put everything on this card. I didn’t like this concept, that you are completely dependent on a person, that... For some, this is good, you don’t have to think about what to do tomorrow, but for me it’s not good because I kind of control myself and do what what will I need. That is, as much as I don’t like being limited by someone. Any limitation here is not very good. That is, there you go. I like the Higher School of Economics because, by the way, there are no restrictions on studying, that is, you don’t even have to attend lectures, that is, you are, as it were, responsible for who you will be. That is, there is no such strict control, so what - oh, you need to learn. If you don't study, you'll fly out. (laughter) As if only this fact stops you and that’s all. There are no mentors there to control them, no micro-management...
Well, this is what happens in life, in general, it always happens, for example, if there was a need to get answers to some questions and close your own questions, whether for business or personal reasons, I found a good psychologist, went to a psychologist, solved the questions, closed questions. Somehow like this. Should I answer in this format? Is that normal? Is it clear now?
A. For health reasons. Well, in fact, in terms of health, what can you say, what can you say in terms of your health... Well, I’ve had problems with health for quite a long time, it was decided that health needs to be dealt with systematically, and the decision was precisely on the advice of on the advice of parents. I adhere to the following approach - once a year you need to do a complete

Check-Up of your health, and regularly take tests and monitor them. And this decision was made very simply, because there was advice from my parents, accordingly, after the advice was received, I understood why this was needed, why you need to regularly track the indicators of your body, I realized how important the energy that you have is, to exist well, and there would be enough of it for what you do, what you do. Well, that’s why I regularly take care of my health, track and live happily and energetically.
M. What brings such warmth to memories of school? In primary school? M. There was somehow more friendly communication there, with the team, but the only thing was that I was very far behind in sports. When they played football, volleyball and so on, I usually sat on the bench. M. Did you want to continue, did you want to play together? Or were there some reasons why you didn't play? M. To be honest, it’s difficult to answer, because on the one hand, it seems like you want it, but you don’t seem to want it, this. M. Was it your choice to sit and not play? M. (..) No, I think my lack of skills played (..) in this.
M. But in general, this is the period before yours, before your state now, right? M. Yes, there was also the military registration and enlistment office story. M. Tell me more about this. M. Well, I was in such a disgusting state of mind that the doctors and the military commissar said, no dear, you are somehow really bad, let us observe you in the hospital and sort of decide whether you need to go there or not. In general, I spent 7 unforgettable days in the Gannushkin Design Bureau, well, I lay there, I just lived there, like, I don’t know, in a hotel. Here. And... Well, I was declared unfit, given category “B”, I never left to serve anywhere. M. How would you characterize this period of your life? M. Well, these 7 days, it would seem, should have been quite difficult, but the only difficult thing was the fact of being there. The stay itself, in fact, gave me some kind of respite or something, because it was even harder at home then. Here. M. So it was some kind of escape? From what was going on at home? M. Escape, escape. M. What happened, how did you react later when you were declared unfit? M. Well, it’s a relief, because if I left, I would feel even worse.
M. Yes, then you can move on to the next point. M. Choice of lifestyle and leisure. Well, this is where it gets more interesting, by the way, because I decided to change my lifestyle. He was like this, he is like this now, more sedentary, not very active, I found myself in such a rather interesting situation that after my previous job and previous company, all my interests simply died, because all my time was taken up by this sweatshop work and gatherings there with drinking there and so on. The rest just somehow fell away. And now I think what is interesting to me, what is not interesting to me, I restored my physical activity to a minimum there, in the future I will... In the future I will return to sports. I’ve even already decided what exactly it will be, what it will be.
This means health is seven out of ten, it’s more likely just a dislike for specific healthcare institutions. Otherwise, I can calmly go to the specialist I need.
Q. I changed, listen, I changed, and I changed a lot of times. I studied to become a psychologist-educator during my undergraduate degree. M. Oh, how similar. V. Yes. I studied to become a psychologist-teacher, entered the Faculty of Philosophy, well, the School of Philosophy, in general, the Higher School of Economics, so (NPT) himself is not very far away.
V. No, there was no such question, but my mother tried to compensate for this so that I would somehow interact with society, so I went to an English language club, to drawing, in general, they took me to all sorts of different events where there were other children, Mom often went out with her friends who had children, so it cannot be said that I was deprived of communication with children, just food, catering, my mother did all this at home, that’s it.
And I started, that’s how I played computer games for 16 hours, in the same way, for 16 hours, I read literature and classics of literature. And of course I tried to write poetry. Here. And then, in all the blogs that I kept on games, I began to write about the fact that games are, of course, cool, (npt)

but it is necessary, well, the trick is that they greatly, they very much limit the space in which you're cool. And I really love being cool, still do. Here. And how could I, of course, be the top one in the game, but if I go for a walk around Moscow, it will give me little. Here. I wanted to develop something with more generally accepted values, in some more generally accepted values. Well, that is, for example, knowledge of foreign languages, erudition, career prospects, and so on. So, at the age of 16, at 16, yes, at 16 and a half, in short, somewhere there, at the end of 11th grade, I realized this and began to work in this direction.
V. Yes. Yes, I wouldn't even call it stress. I kind of liked this state of competition again, another one, here. Well, so did the entrance exams, and I was like, oh, I entered Moscow State University without a basic philosophical education, that’s fine, now I’m also a translator. In general, this energizes me. So, in short, I always loved this state of competition, and as a child, when you are not competing in the professional market, this is probably one of the only niches where you can compete, these are games, well, such games, board, computer, or sports games. Here, by the way, I would actually dwell here in a little more detail, because I love, since I love this whole business, I did both, and the third, that is, I always played board games with pleasure, a la cards and sports games. I say, I played chess as a child and table tennis. Here. But... But just at the age of about 12 years old, that is, at 11, I started playing computer games, and at the age of 12, I left sports and stayed only in computer games. Here. I wonder why this happened, well, I think I don’t know. (laughter)
In general, in principle, I don’t like, I don’t think that anyone knows better than me, especially in pedagogy, in matters of education, because I have a psychologist’s education, pedagogical, I know perfectly well that no one knows my educational does not diagnose needs. Moreover, no one has as much knowledge as I do about which method of presenting material is suitable for me, so this education system does not give me a choice, well, let’s say this happens often, the curriculum says that there are many disciplines by choice, but in fact there is nothing there anywhere, plus the budget needs to be distributed correctly, in the end there is one, well, at most two, you have a choice. In general, I don’t like this all the time, I like it when I build this educational route for myself. M. Well, that is, studying at the psychology department helped you understand yourself? Q. I don’t think it was specifically my training at the psychology department, although... I think that it structured my thinking, of course, certainly my thinking and worldview. In principle, psychology, pedagogy, and philosophy, which I studied, help me understand myself better.
V. Yes, well, accordingly, I created my own group, and spent the whole last year working with this group. And now I want to try again, now I really don’t know, but I still hope to somehow go to study in France, so here I have the same problem, that I’m not a psychologist, that I’m a psychologist-educator, and France is more strict system in terms of changing qualifications, so I need a year at a Russian university. Here. Therefore, I want to study at a Russian university for a year at the Russian State University for the Humanities, and then try to transfer to... Well, there is the city of Caen and there is training for translators in three languages - Russian, English, French, and so on.
V. Well, yes, with health, well, I’m generally not the healthiest right now, I have allergies, and a bunch of other diseases, but our medical system, it’s usually in no hurry to help, and usually, until you get completely ill , somehow they’re just trying to tell you, like, go drink oak bark there and everything will be fine with you. In general, my health taught me that no one except me will worry about my health. Therefore, you need to show maximum will to get a referral, to get the tests that are needed to get a referral to a research institute, and so on. And there are elections, that is, here, too, it’s probably difficult to implement, that is, to withstand the doctor’s pressure, because the doctor has authority, that is, like, are you going to teach me, puppy? You (npt) like me, did not undergo residency training. But, you need to show pressure, I then wrote the first complaint in my

life to healthcare, to the Ministry of Health, here. This is just such a space of struggle for me, health, so I can say that this is... M
A. This means that what happened, well, I had certain health problems, (..) they are related to reproductive function. She was questionable to me. Accordingly, I needed to get a referral for surgery, this is why the health sector is such an important thing. So, I needed to get a referral for surgery and it's very difficult to do, it was, well, this whole story began in 2019, that is, the diagnosis came to light. So, I tried for 3 years to get a referral, and at one time I received it, but then I got sick, it expired, and they didn't want to give me a new one, so. Who were the participants in this situation? Well, the main participant in this situation was me, naturally, and doctors were involved in this situation, but they were more like passive observers than participants. I would say my mother participated more, because she asked some doctors she knew there and so on. My girl too. Where did this happen? So, okay, let me tell you a little further, in general, I tried to get a referral, I didn't succeed, then, in one private clinic... Well, in general it turns out that this operation is very rarely done without any additional shady payments, that's it. And I, I have a principled position that I don't give bribes for anything, well, in general, I don't want to violate this life principle. Here. Well, in the end I decided to do this operation for a fee and it was unsuccessful. And if... Which I perceive, in fact, as a wrong choice, that I probably should have taken a more careful approach to choosing a doctor.
Q. A year ago. A year ago I had this operation, here. Then I felt pretty bad for six months, well, it was very bad for probably three months, so then I made a decision for myself that, well, sort of... Well, first of all, I don't blame myself for this, for the fact that I there may not be enough... First of all, there is no guarantee that if I tried another doctor it would be better. M. Well, yes, there's no way to check it. V. Yes, there is no way to check. Here. And I was guided by the recommendation that was given to me, that is, in principle, I'm not just like - oops, I'll go to any doctor. I've really come a long way, but the fact that it ended in failure does not mean that the path was wrong. M. Well, yes, it's not your responsibility. You did, you approached this situation responsibly, as far as I hear. That is, they did everything. V. Well, yes. So, in this regard, I calmed down. At first I kind of thought, damn, maybe it could have been better, then I thought - no, better, well, much better, as if I did everything I could, for my part. This is the first moment. And then, then I decided that I needed to get a good examination now, that I could... And then I kind of asked myself - what can I do now, that's what I need to get examined again now, I started knocking out all sorts of directions, there same level of hormones, what can be done about it? Here. And I began to move in this direction, received a referral to doctors, so. And then I started dating the girl I'm dating now. When I was just going through the referrals, and before I started dating her, that is, well, it was as if we had already started to have some kind of romantic relationship, so. And I tell her that well, right now I'm not at all sure about any sexual things that I can give you, so I say that let me first go to the doctors, they will prescribe me some kind of adequate treatment, and.. It's just that if I'm in a relationship, then I will feel uncomfortable, that maybe I'm not giving something that I should give in terms of my ideas about relationships. Here. And in general, somehow in the end I... By the way, this motivated me to speed up in that direction, to move, specifically to go to doctors faster, and to get directions with greater intensity, that's it. Well, somehow we continued to communicate with her, with this girl, and in the end all my functions returned to normal on their own. Here. Yes, but this happens, there are paired organs, the functions of one are taken over by the other. Here.
M. Tell me, do you remember the transition from your state of complete hopelessness to the fact that I can do something and in general, well, I can be responsible for my life and can take some action? So what was this connected with? Any external circumstances or more of your internal transformation? V. Tax. (...) Now, I'm trying to remember. So so so. (...) Well, at first, yes, I would

probably describe it this way: at first I was just in shock, I didn’t understand what was happening at all. Here. Then I began to feel the consequences, that I began to gain weight, and then I began to worry. Well, that everything is not going according to the best scenario that could have been hoped for. Here. Then, it’s not like there was some kind of sudden event, it’s just me, this question bothered me, it’s like, I like to talk to myself, so. According to the method, like these two chairs, like what are you experiencing. How would I do something like this? So, this guy talked, so, okay, what do we want? We want to worry, or we want something, not to worry. Here. Let's think about what we can do. So, well, we, I mean, I just address myself like that, (laughter) here. M. Well, maybe some parts of you. V. Yes, yes, we are... M. They decided to act together. V. Yes, yes, yes. Well, yes, we are me, torn apart by the contradictions that exist within me. Here. As a result, I talked to myself, came to the conclusion that I... Yes, another thing... Yes, I remembered, I was very worried, really very much, and it always scared me... And I was even worried not so much because of what happened, but because I didn’t understand what to do next. Here.
M. So the uncertainty was scary? V. Yes, I didn’t understand, that is, which doctors to contact, where to go, that’s it. Both then and there there was still such a problem that everything is very slow in our healthcare system, that is, there, for example, they give a referral, but the appointment is only a month and a half later. I’m like damn, what am I supposed to do for a month and a half? I, I’m very worried about this, but I can’t do anything other than this recording. M. Yes, it's terrible. V. Well, that’s when I started writing complaints, this greatly speeds up our healthcare system, by the way (laughter). Here. Moreover, even those to whom I did not write, you just tell them that you have already written one complaint, immediately become more and more talkative. Here.
Well, a good choice, a good choice, I would probably attribute to my first university, that is, psychological and pedagogical education. And again, this is one of those choices that I made, guided by one criteria, and with the hope of one result, but it turned out to be good, that is, the results turned out to be different, but better. Than I could have imagined. That is, I chose a psychological-pedagogical course, because I knew biology, chemistry, mathematics, Russian, with these subjects I could enroll either in some kind of biological-chemical thing, like there you could go to Timiryazevka to study genetics, or to honey didn't pass. Well, in short, there was an option, either to go for something like that, biological, I was basically interested in genetics, at school I loved problems on genetics, but on the other hand, the option was to go to a psychologist, a psychologist-teacher, that’s it. And, in fact, I thought, damn, now I’ll go see this geneticist. Although I like solving these problems, is it that I will spend my whole life developing new plant varieties, animal breeds and stamps, strains of microorganisms? This is like the definition of genetics, actually. (npt) I think, God forbid, this is boring. I want to communicate with people. And I went to become a psychologist-educator. And why is this still a good choice, because I really liked my university, the Moscow State Pedagogical University, it’s not very overloading, I can, as a person who has already studied at a bunch of universities, and who has many friends from different universities, I know that it is so, moderately stressful. And there was a little more tension in the first year, and then just not at all, not very much. And I really liked it, because I think that if I had studied at another university, I would not have been able to simultaneously learn French, for example, to a high level, because I would have to complete some endless tasks there. And I would not have been able to gain work experience either at Artek or at Detsky Mir, or as a chess teacher, I was still working. In general, I would not have gained much work experience. I would describe the choice of university as successful, but now let’s talk about your criteria. So, when this happened - in 2015, who was among the participants in this situation? Well, I can say that naturally I was the main actor, but here my parents were naturally involved, like the parents of many children after the 11th grade, so. They gave me some advice there. I probably made this choice at the dacha. I was just wondering where, well, I was looking. I sat with the lists and in the end decided that, well, I

went to different ones, and decided that psychological and pedagogical education, it still sounds universal, like a psychologist and a teacher. Of course, this is actually a trap, if anything, it's a trap. In fact, you won't be able to work as both a teacher and a psychologist (laughter) because you don't have a subject to teach. (laughter) And the psychologist is also only a school psychologist. Here. (laughter) But I didn't know that then.
Well, next is probably health. A year ago, well, a little over a year ago, (..) I had an operation there on my leg, to straighten my toes, that too, it was a necessary operation, and after which there was six months of recovery. Here. And... M. Necessary, that is, it really interfered with your life? Has this problem been around for a long time, or has something happened? K. Well, about two years ago, even 3 years ago already, it appeared, at first I didn't do anything, it went away after some time, there after ointment or some other such exercises, but after that the condition began to worsen, and It got to the point where it was painful to walk. Here. And so a decision was made there, here. And this summer I also thought about having the same operation on the other leg, but now all this is being postponed for me. Perhaps next year, perhaps even the next year. Because there are (..) various issues, including financial, time, because you definitely need to spend a week there under observation in the hospital, and after that you need to wear special shoes, and now it will soon be quite cold. It will be uncomfortable to walk in such shoes, so I am putting off solving this problem.
Here. Finance. Well, here the choice is probably only within the framework of finances, it's about health and about resolving some issues in this regard. M. Is this about leg surgery? K. Yes, including, plus other various problems, there in the summer I decided to do dental treatment, so. Also when it started to become quite expensive, and then other ailments appeared that needed to be urgently addressed medically, so. Therefore, dental treatment there, too, has been postponed and is being postponed there indefinitely.
M. Yes, Dima, thank you for the detailed story. I have a few clarifying questions. Firstly, I notice, well, naturally, here there is a moment of professional sociological education, albeit lasting one year, that you use a lot of terms, and with them you seem to indicate some kind of processes that arise in your life, but according to Basically, I have the following question, sometimes you say, for example, that there's this, this stupid procrastination, or... That is, you give some kind of assessments, and in connection with this it's interesting, but you're with someone... Are you discussing it now, or have you discussed those difficulties, including psychological ones, that you have encountered, and sometimes, well, naturally, they arise in life? D. I discuss them, but I discuss them mainly with friends. With my family, (.) I don't have the habit of sharing anything in particular and asking for advice, for some reason I have a big prejudice in this regard. It just seems to me that older people, my mother is a provincial teacher there, she is unlikely to understand (.) the things that I can tell her about. And you don't discuss it with friends, but only after the fact. Now here, with newfound acquaintances, I can discuss what I went through, but every time I go directly through a crisis, I usually don't share it with anyone, well, also probably because I don't think that anyone something can help me with this. Therefore, this is only after the fact.
I try to be kinder to myself and to others. Excessive self-flagellation and excess severity towards your friends, it rarely bears fruit, I try to refuse it. (...)
B. Well, it's quite hard for me to say about my peers, but I don't think that everyone actually thinks about this in any specific way, well, that is, it's still some kind of process that happens one way or another, in most cases, on its own with myself. Personally, I'm just getting this from, in principle, this is part of why I decided to take part in this interview, I'm interested in the topic of psychology, I have a good friend who is studying at Moscow State University of Psychology and Education, if you know, to become a (NPT) psychotherapist.

B. I personally find out about this from some sources, like Telegram channels, Instagram of some well-known psychotherapy services that run, some of them make short posts on some topics, that’s it. Well, sometimes I read some articles, but just considering that I don’t seem to know much, I approach it carefully. But in general, this is probably the most important source of information about this topic. Well, about other, some psychological topics too.
K. (.) Yes, these are some restrictions on the part of the parents, because of vision, for example, you couldn’t play on the computer for more than one hour a day, there are some restrictions on the time spent on the street, that is, when to take a walk there , restrictions...
M. Did you have any prohibitions in childhood that you definitely shouldn’t, both in childhood and in adolescence? S. Only if you consider it an allergy, that’s all. M. That means taking care of your health, right? S. Yes. M. Was this somehow instilled in you by your parents and did you want to break it, or how did it happen? S. No, it’s just sometimes... Well, it’s like I’m allergic to nuts, but I really like nuts. But I understand that... What will be the consequences. And, well, like, as a child, I somehow didn’t attach much importance to this, but I go to camps, let’s say some sanatoriums, my mother always tells me - do you remember that you can’t have nuts? Do you remember what will happen? And then I, here. And in terms of alcohol and smoking, I was somehow not prohibited at all, but I also had no desire. Here. And my parents forbade many of my friends and now they are just having a nightmare there. M. That is, on the contrary, the ban gave rise to... S. Yes. Because with this ban, they seemed to have more and more desire to try. They didn’t forbid me, my mother always told me that you can try, but it’s not a fact that you will like it. Here. I tried alcohol for the first time at the age of 18, at graduation. M. That is, quite recently, right? S. Yes. And somehow, I don’t know, now I drink a maximum of champagne, wine, and on holidays. All. Smoking, I have it in general, I don’t even have the desire, I’ve been offered to try it so many times, I say, I don’t know, I don’t want to. There is no desire at all.
decided to carry it myself. (..) Well, I went up to the guys, near the tents, to the boys, I said - boys, can you please help me carry the suitcase, I can’t do it myself. They told me - no, we can't do it now. I say, okay, okay. I'll carry it myself. Here. And somewhere in the middle of the journey, I already realized that I simply couldn’t cope, that this rib was starting to ache a lot, there was pain. And I wrote to the chat with the guys, I said - guys, the boys really need you now, please help. Here. And no one reacted at all. Here. And I just walked, roared, carried this suitcase, so I carried it. But most of all, I don’t know, what made me laugh was that I was walking, and so I met many young people, they were just walking like - damn, she’s so cool, really, strong, independent, carrying a suitcase herself, in general. I walk and think... M. And it was very painful for you. S. And I walk, roar and think - you didn’t think of offering help, somehow, I don’t know. I even, I don’t know, I always somehow offer help... (..) I, I don’t know, I’m used to doing this, so. Then I just went to the doctor, they told me a lot of things about how I shouldn’t carry heavy things, but I went and did all this, that’s it. So. Do you often think about it, about this situation? (.) Well, recently, often, because it happened recently. (laughter) Here. I told my parents, my mother said that everything can be expected from me, and what emotions arise? The emotions are so mixed. M. Can you explain? S. Well, in the sense that yes, I myself was able to convey all this, but it was to my own detriment, but also that, as it were, I don’t know, we were always told from childhood that boys should help girls and all that. And then they just go like this...
M. Where do you study and for whom? M1. I'm studying at the Higher School of Economics to become a psychologist.
M. Now I will ask you to give an example of a situation that you especially remember when you showed independence. I will send the main points to the chat, which I will ask you to indicate. Please look at them and tell us what kind of situation it was and then down the list. M1. Okay, now, I'll think about it. (...) Well, we can say that this is admission to HSE, the Higher School of

Economics, because many questions arise when you go to study psychology at the Higher School of Economics. Here. Because I am studying commerce, but I also submitted my documents to RANEPA, the institute of the President of the Russian Federation, and there I got into the budget. And how it would have been, one might say, a year ago, during the period of admission, and I decided that I wanted to go to HSE, because there is really strong psychology there, there are cool subjects, teachers and in general I like all this, and at RANEPA I I don't really want to. And who was with me... Well, I decided it myself, I just brought the decision to the family table, so to speak, and that’s it. Well, parents. Well, that is, they didn’t press, they just said - choose where you want, and that’s it. And how did I understand that I was doing it on my own? Well, probably because many were unhappy with this decision, well because probably, for example, my grandmother did not understand why I was entering some kind of Higher School of Economics, although I could go to a university under the President of the Russian Federation. And so on. M. Didn’t that stop you? M1. No, that is, like grandmothers, they are generally like that, you know, well, they’ll say something, I’m not much different there, because after all, it’s a different generation, and somehow I’m very loyal to them, so I just said - Yes, I’m such a fool. The situation says something about me... (.) Well. probably that I am following the goals that I wanted, that is, in the 11th grade in September, I opened the top psychology universities, saw HSE in first place, and that’s it, I went there. And she achieved what she wanted. Do you often think about her... Yes, like boasting, of course, that you could go to the RANEPA budget, yes, I love it, and my grandmother often talks about it, remembers it because she would like it. But life turned out differently. Yes, I tell my parents, well, as if they know everything, sometimes I tell my friends, because in principle the situation is cool and interesting. They said... Well, I said about parents, relatives, but friends, they usually, well, somehow just laughed, like something like that and that’s probably all. That is, there was no violent reaction there, because choosing a university, as it were, is a situation that every one of my peers has encountered, so they basically understand and there is no condemnation here and so on, despite the fact that I like it Studying at HSE is the opposite - well done for choosing a university that you still like. Emotions... Well, probably pride, joy and some kind of nostalgia for those times, (..) probably these are the main emotions.
Well, perhaps, when my neighbor became ill, she began to choke, and... That is, a person is suffocating, he doesn’t know what to do, this is his first time, and it’s like... That is, I called an ambulance, there I woke up everyone , she said that yes, we started googling something together, what to do and so on, that is, as if she had something like this, nervous, panicky, that’s it. But then she went to the doctor, well, it was like, you know, an independent decision that at the moment I should help the person. Because she can hardly help herself. M. This was probably a very stressful event and somehow you reflected on the moment when - now I’m doing it, or did you just start doing it yourself because you realized that the responsibility was only on you at this moment? M1. I guess I just started doing it on my own. M. Do you think this situation played any role for your independence, or did you simply show what you already had? M1. I think I just manifested what I already had, because my sister is often sick, (laughter) more precisely constantly, so I kind of got used to packing things in bags when I have to go to the hospital, that is, for me it’s a familiar situation, so it was sudden in terms of its appearance, but in terms of (..) how should I say it, in terms of factors, it was similar to many situations in life.
M1. Now I'll think about it. (...) So, well, one successful one. (..) I was in the eleventh, no, tenth grade, I wanted a dog, and at that moment I already understood that I definitely (xx) would not stay in my hometown, in Rostov, that I would definitely move. It doesn’t matter which college I go to, but I’ll definitely move. And... But I really wanted a dog. And as if we were looking for a dog, we found it, we had already paid a deposit, well, like, in a few days I have to go pick it up, and I

understand that how can this be, then I will leave the dog with my parents, because I will live in a hostel , well, for sure, I had a choice either not to get a dog, and let's say, not to fulfill my dream, because I never had my own dog, we have a lot of dogs in our family, but we didn't have our own. And no one knows when I would be able to get myself a dog. Or cancel everything, let's put it this way, and simply not burden the parents. And that was the choice. Either you blindly follow your dream, then you burden other people because they need to take care of your animal, or you care about other people more than your desires. This happened in the tenth grade, I was probably 17 years old at the time, and me, my mom and my dad were involved in this situation. Because my sister doesn't live with us, therefore she won't take care of her. Where this happened, this happened in my hometown, this situation made me feel like I needed to decide something, that I needed to do something and that I was afraid of making a mistake. I'm afraid of ruining the dog's life there, I don't know, causing some kind of trauma to myself, or burdening my parents with this too. That is, the situation was not pleasant. The choice was not a pleasant one. But now, (..) I treat her calmly, I can't say that she directly somehow excites something in me, probably, I just remember with some pleasantness all this then... All These are my decisions that led to the fact that I now have a dog. (..) It evoked different thoughts. (...) Don't even know. I think, probably, I was just very afraid to hang some kind of responsibility on another person, I was very afraid that the animal would forget its owner... And it's not very pleasant for me either. (NPT) kind of like for myself, but like for others, too, so. Now this gives me (..) also mixed feelings. Well, that is, I don't know why I did this then and decided to get a dog after all, and I don't know what I would do with this situation now, well, that is, it's like you just decide in the moment, yes or no, and probably Then I just let go of all the consequences and lived in this moment, that yes, now I want a dog, now let's live in this gorgeous moment, and then I will accept the consequences and decide what to do with them. Here. But I think that it turned out well, because I love her very much, I have a gorgeous friend who will always hug you, lick something, and so on, make you laugh, play with you, that is, how These would be very pleasant moments, memories, so I think that I made a good choice.
probably my sister also has health problems, so she, a little like this, somehow maximally, (sigh) I don't know, reaches maximum heights during education, and therefore, probably, this is also a little bit of my responsibility, (laughter) also everyone, and they also pay a lot of attention to her education, even more than mine, because it was more difficult for her to study, but certain hopes were probably pinned on me in this regard, but I'm not exactly (npt) on this topic of approval, that you should do something there, always do everything well, I don't know. (laughter) Well, yes, in general, this was also important to my grandparents, but what my grandparents on my father's side, basically, my grandmother was more likely to just say, go ahead, well, it was important to her that I do in basically anything, and I moved somewhere forward, and managed to do everything. But it didn't matter to her in what area I was doing it. Here. Probably so. M. But it turns out that both pairs of grandparents have higher education? I. Yes, everyone has a higher education, yes. M. I'll carefully ask about my sister: what health problems did she have, were and still have, what is the reason for this? I. She has autism spectrum disorder. But she is quite highly functional, that is, there is no such thing that she, I don't know, it's probably not very noticeable, in terms of... (..) For people who are unfamiliar with this, they probably won't immediately understand that something is wrong specifically. That is, they may think that she communicates in some strange way, or something, I don't know. Well, in general, not so much that she couldn't study, for example, but in life it interferes with her. M. But you said that due to the fact that your sister has such difficulties, sometimes they even paid less attention to you, and so you, well, how much did this bother you? That is, did it evoke any emotions? I. In general, no, probably (laughter) only because they always paid a lot of attention to me, and sometimes, I would say that it was even some kind of

overprotection, so for me, I don’t know, it’s just very difficult to assess the effect and the cause , that, for example, they looked after me a lot, because my sister has difficulties, for example, for a very long time I was not allowed to go anywhere on my own, (..) well, on the subway, or on some public transport, and they were always driven, and it annoyed me because I wanted to travel on my own. And I don’t know what this is connected with, maybe because my sister couldn’t drive for a long time, and I started driving earlier, my parents were afraid to let me go. It seems to me that some of my parents’ fears, as it were, related to my sister’s health, are transferred to me. Well, at least it was like that before. Probably not anymore. But maybe, maybe I’m just thinking, in general, I’ve never had the feeling that they pay more attention to my sister, because I also received a lot of attention. And vice versa, probably at some moments it was good that this attention did not go to me and I, I had more freedom.
M. Did you feel older precisely because she had just such health difficulties? I. Yes, because for quite a long time, but even now, she still doesn’t have it so strongly, for a very long time she behaved as if she were much younger. Therefore, at some point, well, I remember, right when I realized that she had some peculiarities, I asked my mother, by the way, I even remember this moment, I was driving, we were going either to a dance or with dances, and my mother began to tell me, I was about eight or nine, and she gave me a book to read, well, it’s such a cool book, “Autism through the eyes of a sister,” it’s like from the point of view of a girl whose brother has autism and she tells . I think yes, it’s quite similar, because some patterns of behavior are described there very, very similarly. Here. And when I realized this, I somehow began to relate to her easier, because when you know why she behaves so strangely and it annoys you, and it’s not clear what to do about it, when you know it, it’s easier. But still, I noticed that my sister acted as if she were younger, as if she were younger than me. Therefore, I didn’t have the feeling that she was older, and that somehow... In general, I was probably ahead in terms of development after all.
Regarding health, there are probably no solutions. At least nothing comes to mind. There, every year you just go to the doctor, and he tells you what to do and that’s it. (laughter) M. So you are generally calm about your health and therefore somehow don’t even remember? I. Well, I probably haven’t had anything like serious health problems lately, if something happens to me, I go to the doctor, but I don’t know there, I recently had poisoning, I go to the doctor, he me right away (npt) (laughter) but there were no such, well, particularly important ones, but probably if I had had any health problems lately, I would have remembered it better, but so, (laughter) nothing there was no specific one.
M. But it seems as if you have done a lot of work compared to the way you talk about your adolescence and the way you behave now. You are very open, you are very emotional, and talking about such experiences is a great courage. In fact. N. (laughter) I have chosen a psychotherapist several times and now I finally go to a normal one, and everything, plus or minus, yes, is being worked out, so it’s just... Well, that’s exactly what I’m talking about, about the fact that from the age of eighteen, I can finally make choices myself, and I can, as it were, in an emotional way, that is, choose what I feel, what I don’t feel, and what, I can behave this way and be like this, that is, in some moral things, and in some actions. That is, I decide where I will do the actions one day, and I chose this, and I will, well, kind of lead you like this. And be like that. This is also like a choice, in my head, it’s considered, because, well, just then, all these things, they were very strong, I cried there for months, and in general, I felt even more downtrodden, but fortunately, some things started there. then a fairly adequate relationship that lasted quite a long time, and this somehow made me feel that I was not at all... There is someone who loves me, someone who appreciates me, the same one like this... Replacing unconditional love with parental love, that's it. And probably, in parallel with all this, probably in parallel with this, there were also elections in the 11th grade... M. Can I

have a second? Tell me, do you attribute your transformation to a greater extent to psychotherapy, or perhaps there was also some event that made you look at yourself differently and begin to behave differently. N. More likely, to a lesser extent with psychotherapy, but that is, I started adequate psychotherapy literally four months ago, because before that I just went to psychotherapists, and somehow, well, I couldn't find someone with whom I would be comfortable, Here. So I associate it rather with the fact that the most important thing is that now I can, since I was eighteen, I do not depend on my parents, that is, if before that my parents could forbid me something and somehow control me, now, well, this is absolutely not the case, and we have some kind of, well, adequate distance, Well, you know, there is an expression, healthy indifference, something like this, probably with us. It's clear that I love them and this is unconditional and absolute, but how could I...
M. And after 18 years, what important elections did you have? Besides work. Maybe in some other areas? Independent travel, I heard this spring, maybe something else? Choosing a psychotherapist? N. Yes, choosing a psychotherapist. That's for sure. (laughter) An important choice, I'm very glad that I found my current therapist, she's very strong there. Yes, independent travel, because at first I started traveling around Russia, it was also funny, of course, because I turned 18 and Covid began. Like hello, thank you. Nastya, who had never been abroad before, or even in any other cities, here. Yes. So I myself began to travel slowly around Russia, sometimes in a company, sometimes on my own, and then I decided - damn it, I've never been abroad. Here. And yes, in general, I'm very glad that I went to Istanbul, so. Because I went there myself too, that's it. That is, completely alone, not knowing much (laughter) English, that's it. But it was great and I'm very, very glad that I went after all, because well, that's exactly my trip, I bought tickets there a few weeks before February 24, and the tickets that I repurchased were canceled 10 thousand times , and I was already thinking, damn, how am I going to go in such an atmosphere, but I went anyway. And I'm very glad, because... Well, at least this ticked some box, because you still felt as if you were in some kind of geographical cage and (npt) get me out. And this summer I also went to Dagestan, on my own. And this was also wonderful, it seemed to slightly support your own feeling that you are doing something with your life, and you are sort of solving not only some work or romantic processes, so. But you also allow yourself to receive some kind of pleasure. Here. Yes, what other elections, well, probably a partner, we still have to talk about it, because this is my first breakup with that young man with whom we dated for two and a half years, this is probably an important decision that I accepted. And after that we dated for a year, because I, in fact, I left him for another partner, with whom we were in a relationship for a year, it was such a very cinematic relationship, with... I think you know the expression Dead Inside, here, (laughter) this is it. (laughter) So, in the end, we parted ways, well, that is, I can generally say that he abandoned me there... Because of his own pens, because he was somehow against me projected, as they say, and this was also a rather difficult period, just around March - April, all this was also layered on February 24, and (..) there I did not make any choices, (laughter) this is exactly there was a moment when I directly felt that I had lost some control, and because of this, well, it was really very difficult. Well, it's also related to this, that is, I found a psychotherapist right on the same day. Here. And thanks to the fact that I decided to find her at all, and because I found her, I somehow, well, generally survived all these moments, because of course the first separation was very difficult, well, actually without your will, which was done. Here. Well, then there, after some time, it seems that I met a young man with whom we now have, well, some kind of relationship begins, this can also be considered some kind of, well, choice in the romantic part is important, because Well, there is 50 percent of work that calms you down, and there is also, well, it's clear that it's not 50, there are other breakdowns, probably 30 percent of work, 30 percent of relationships, 30 percent of friends, and then there's 10 percent, this is some kind of totality , oh well, the family still needs to give some part of the

interest, so. Well, that is, I just remember that just when I was in Dagestan, I felt very happy, because finally everything in my life, plus or minus, seemed to work out, and this, well, if you look at all these segments, Well, they're pretty good. Here. That's why...
M. No, on the contrary, one is easy, but ten is difficult. So then you have three in training, right? N. Yes, yes, yes then for training three, and (..) health - (..) one. That is... M. Easy? N. Yes, well, you go to the doctor, make an appointment there (laughter) if something worries you.
N. Well, then, I think that I can go back to the choice that I made when I was 16, when I was in the summer, when I decided to go to the young man with whom, who hinted to me for sex, for some kind of romantic relationship, that’s it. And to his friend's house. Well, accordingly, I was 16, that is, what year was it, 2018, yes, it seems. Yes, summer 2018. Here. (..) Accordingly, I was there, this young man was there, and his friend was there. And so I ran away from home to spend time with them, because it seemed to me that it would be cool and that I would probably get closer to him, and indeed, he would kind of like me as a person, and that’s how he would like me probably appreciate it more. Well, in principle, I liked the feeling that he was paying some attention to me, that a person seemed to appear who appreciated me, at least for something. Here. Maybe sexual desire, and it seemed to me that, well, in general, I could probably somehow, well, show interest, and he would appreciate me even more, but for something else. Here. And all this took place in the Moscow region, at his dacha, in his house. And... Well, it happened, (laughter) how to describe it, I just don’t want to go into any super details, but (..) in the end, after we first talked with his friend, who told me what - there with these vodkas, so, and then this friend himself came, oh, not a friend, but the young man himself, with whom we had some kind of fuss, so, and he took me upstairs, and then I continued I remember it very well, (..) just in very small snatches, here. But it was all connected with some kind of (sigh) (..) well, sexual actions. In the end, I don’t know how he would have entered me or not, but he tried, let’s say so. Here. And what kind of feelings this situation caused then, obviously - not the most pleasant ones, well, that is, I was probably in some kind of teenage age, so that everyone, I was just, I was just worried about everything. Well, it seems to me that everything was just finishing me off gradually, that is, it wasn’t so much the situation itself that was finishing me off, that is, the fact that... Although, well, in general, it’s difficult for me to separate this for me, the fact that we then stopped communicating with him , that is, he seemed to write to me at the beginning, well, that is, maybe he wrote there twice, so. And that’s all for me... (..) Well, sort of, the fact that he’s no longer interested in me at all, and the fact that he absolutely doesn’t need me, and the fact that no one loves me, doesn’t appreciate me, I I felt absolute loneliness. I remember that I imagined that I, firstly, well, that is, I just had pictures in my head, like I was alone in the middle of a white sheet of paper, and in general there was no one close to me, as if my parents don’t give a fuck, my friends and I are so close We don't communicate, that's it. And, well, that is, it just felt like such total loneliness, rather, this whole situation led to these feelings, so. And what feelings now, (sigh) does this situation evoke, now, for a second. (...) (sigh) Well, probably (..) I just thought it through, thought it over 10 million, billion times, turned it over in my head, and somehow the psychotherapist and I seemed to have worked through it, give or take. .. And now I just feel some kind of, I don’t know, sadness, sympathy, probably for myself, but it’s global, because, well, obviously none of this would have happened if I felt somehow there much better, that is, as confident and calm as she is now.
N. (sob) Thank you, it’s very nice to hear all this. It’s just that now it’s actually much easier for me to tell all this, because it’s all already agreed upon with my loved ones, and well, my friends, my former partner, or my current partner. And to the psychotherapist, here. That is, like another year or two after what happened, of course it would have been more difficult for me to tell all this, but now it’s like... Well, you just understand that... Well, somehow in general, it’s all rational,

meaningful and... But thank you very much for the words of support, it’s very (..) nice every time, me too...

M. I really like the way you praise yourself, it’s really great that you acknowledge your victories, understand and build it into your story, you understand that yes, I really did it right, and now I get what I wanted, and even unsuccessful elections in the past become not so unsuccessful; on the contrary, I am very glad that I made a different decision then. N. Well, I think that this is all kind of connected, plus or minus, with the fact that I turned eighteen, I began, plus or minus, to somehow adequately perceive myself and the world, and then again, now it’s like I’ve added to all this psychotherapy, and but I just, as if my emotional intelligence is growing a little, you can, well, somehow listen to what you feel and already determine what you feel, and not just feel and be like - oh, I feel bad, (laughter) here.

As for health, well, as such, well, firstly, I chose to do a health check-up in order to identify some... Some areas in which there are some problems, this was already a big step, because I couldn’t get to a regular doctor. And so I chose not to put off some moments. Of course, now, with a new workload, it’s been postponed for some time, but I think I’ll get back to it soon. (..) And I will start again the procedure that I was doing. M. And what about regular health checks? How regularly will this happen? A. Regularly - this is six months. (..) And about work, work... M. Sorry, please, can I ask you more, about your decision about regular health checks? A. Yes, of course. M. How did you decide to do this? How did you make this decision? A. (..) Well, since we now had Covid, I... And I never had confirmed Covid, I decided to go and find out if everything was fine with my lungs and everything else, and since now there are pavilions in Moscow, health pavilions, and there is one of them literally next to my house, I thought that it would take an hour, an hour at most, and I have an hour of free time, I can go there and do it. And also, well, when... (..) When you underwent medical examination at work, mandatory, it’s also like a choice that we are not given, (..) what you are obliged to do, and also this, well, some your own feelings that something is wrong and you should go to the doctor, and he will refer you where you need to, according to your needs.

M. Yes, of course. If you think that's it, move on. A. Relationships with parents. This is a very difficult question, because... Well, I have always had a very (..) (h) great attachment with my parents, because they literally did not let me go anywhere, and it was this year that I decided that I would completely separate from them, I worked with a psychologist about this. And... (..) And I tried to distance myself from them as much as possible, because I understood that I was already independent, I was already old enough to make decisions on my own. And, (..) well, not even listen to any advice, realizing that my experience, conditionally, of living in Moscow, studying and working is completely different, like theirs, in another city, and since we are completely different generations , because, well, my parents are adults. Here. And in this regard, it turns out that relations with parents may have cooled somewhat, and they are not as active as they were before. (...) Are there any additional questions on this point?

M. Please tell us in more detail how it happened that you wanted to start volunteering? A. I accidentally saw an announcement about holding a marathon, in general, in my city, I had to run it, but there was some kind of large fee, because, well, I used to do athletics, and I thought that I could run, but there there was some kind of large contribution, and I thought that well, I don’t really want to ask my parents for money, and I saw that volunteers were needed there, and I simply wrote to the post office. And after 2 months they answered me, here, come. And then again, again, again, again, and so on, now several projects a month. (laughter) Or maybe even a week, and in general, now no longer as a volunteer, and not only there on all-Russian, but also on international projects. Even now, no longer as a volunteer, but as a manager of volunteer projects. M. Great. Is this related to sports, are these different projects, or is it one direction? A. We did this with sports, but now sports are a big, well, sports projects, they are a big part of my life. And the rest of the

projects are of different directions, just the very fact of volunteering, the very fact of helping, it remains unchanged. M. Can you tell me what feelings the situation caused you to decide to volunteer? A. Then it was very interesting to me, because this is new communication, this is a new community, these are new opportunities, because I really missed communication exactly in adolescence, then it caused a feeling of such, perhaps instant admiration, that is happiness is in the moment, now this also happens from time to time, precisely at some event, but you just realize that you have something to do, and you can go to any event and go help someone. M. How do you feel about this now? A. Now I'm completely delighted, because you live this, you love doing this, when you're at some event that you really like, you just almost cry with happiness that you're there.
And probably one of the things that helps me very, very much is that I have been involved in sports since childhood, at first I went to a circus studio, probably from the age of 4 to 9, I really liked it. And I liked the fact that there is freedom of creativity, and there seems to be some kind of framework, in which you have a coach, you have your leader, and you, for example, put on a number with him, that's it. We also traveled a lot and participated in some international events, and I probably even received my first salary, well, as I believe, I received it abroad. And it was interesting, because these (.) were moments that relieved the fears of the unknown, because for example, we performed in front of a huge audience, there on the streets and did some kind of performances, despite the fact that we were all small, we in fact, they were very shy, that's it. We somehow overcame ourselves, and it was somehow unobtrusive or something, because we perceived it all as a game. And now I understand that I can also use this approach in some life situations.
And I kept asking, asking my parents to bring me to the gymnastics group, and then I still don't know, maybe my persuasion somehow influenced my family, maybe something else, that's it, but I still They brought me in and I started playing sports. And I understand that it's probably sport, it has played a lot in my life and in my character, too, a lot from sport. I have there, regarding the achievement of any goals that I set for myself, that I can gather there, even if I understand that it's hard for me, bad, or something else, I understand what I have after all, there's this kind of core that probably doesn't allow you to give up when you just need to pull yourself together and do something, come up with something, that's it. Or somehow cope with the situation that exists. Here. And probably the fact that in my family, in general, (..) there is such a moment of complete freedom. That is, I chose, after school I entered a technical school, because I did not enter the university, and my parents said that you choose a profession and direction yourself, because we want you to be a good person. And of course, perhaps, I think that this was bad advice, because when you are 16 years old, and you want to get specific, well, some steps, how to choose, what to do, maybe how to look at the test results, according to career guidance, which shows you 16 areas of activity, and you don't understand what to choose. Here. But it seems to me that this is my path in education, it seemed to be a little in different directions, but it seemed like it was almost cultural, almost literary, probably. Therefore, somehow everything comes together, as if like a puzzle, here you are, seemingly from different places, but you find puzzles that your picture is missing. It's probably something like this.
stopped playing sports at the age of 16, and probably now, after a certain number of years, I would tell myself that I still need to continue to exercise, even a little, even a little bit at a time, maybe a couple of times a week , but still do it. Because at that moment I had completely finished studying, that is, I had some activities, not much at all, but still, here you go. But I wouldn't want to quit. This is probably the choice I regret. M. But if you go deeper, when exactly, how old were you when this happened? P. I was 16 years old, it turns out that I graduated from school, began studying at a technical school, and I simply did not study in the area in which I live, and not in the

area, and not even close to the area in which I played sports. And plus it so happened that at that time the group in which I was studying was disbanded, and then I thought that, well, the coach and I also discussed that it would probably be better for me to look at some university or technical school somewhere. activity, because I trained with girls who are younger than me and from a sports point of view, this is kind of a step back. And significant, because if you train with those who are on the same level as you, or a level or two higher, that's good. And when you train with those who are kind of far behind you, it's not good. Because you, too, will roll back to approximately their level. Here. And then, yes, at that moment I thought that yes, it would probably be difficult to travel, because school would end anyway, it would be very late and there was still a road to class, and classes too, and it would be difficult. Here. Well, then I bought a membership, a membership to the fitness room, that's it. But it was also difficult with him, because he was also not so close, but still. Here. And I went and worked out, but I understood that this was not enough for me, and I just wanted gymnastic activity at that moment. M. But you said that at that moment, well, as an adviser, well, the coach was present in this matter, and who else? Who else was involved in this situation? P. No, just me, me and the coach. So, it turns out, I told her (..) my situation, and so, well, she suggested this option. Of course, she wouldn't have refused me if I had said that I would like to continue, so. But she outlined the situation to me, as she sees it, as a professional, as a coach. M. But if, in contrast to unsuccessful ones, we talk about successful elections, what elections do you think were successful in your life? P. I would probably highlight the choice of sports, gymnastics, as especially successful. I understand that it's mine, and I understand that it was very important and great that I liked it. And I'm probably lucky in that this sport is interesting not only to me, but also to my family, and my family really supported me in this, then the choice... (..)
M. Tell me, how did the desire to go to study journalism come about? Why did you decide? L. Well, in general, until the seventh grade I wanted to be a psychologist, so. But... M. How interesting. L. Yes, my dad is just a psychologist, that's it. But in short, there was such a story that I began to find out how to enter the psychology department, and found out that I had to take biology there. M. Biology, yes. L. And biology was generally my favorite subject at school, and I say - no, I'll look for biology (npt) for something else. And so I started buying all sorts of magazines there, these ones, for girls, (npt) then later, I became interested in all kinds of fashion journalism, and then it means, I don't know, there in the eighth grade, in the ninth, I became more interested in politics, I wanted to be a journalist who travels to all sorts of hot spots, that is, something like that, little by little it became more and more boring, then I just wanted to be some kind of observer, that's it. Well, that is, it was just some kind of interest of mine, based on some of my predispositions. That is, I was always good at writing texts, in principle, my language was so good, well, I understood that this is what I am passionate about. Here. M. That is, but it was as if it was a choice from the opposite, that if not a psychologist, then I would be a journalist. L. Well, I wouldn't say that this is probably a choice from the contrary, that is, well, my interest in psychology never went away there, my dad always gave me some books to read, he conducted all sorts of tests with me, that's it. It's just, well, maybe I'm also partly burned out, because, well, I'm basically the kind of person who, if I really want something, I'll hurt myself, but I'll achieve it. Well, that means it wasn't such a really great goal, because if this goal had been great, well, I would have learned biology. Well, what can you do? (laughter) M. Why did you want to become a psychologist? Because dad was a psychologist or is it somehow different? L. No, I actually found out that my dad is a psychologist after I decided to become one. But this, you know, is such a story that you never know what your parents do, and then you find out. Here. Well, in my opinion, it all started because of the series "Lie to Me", I was very interested in this topic there, like body language here (npt) that's all, it was so cool, then I read that There are sports psychologists, I

thought that was also interesting. I wanted to be a sports psychologist. And then I began to get interested in serial killers, just reading their biographies, and I wanted to be a psychologist who works with all sorts of criminals, that is, he understands why they commit such terrible things. Well, maybe I also watched enough of some TV series there, like “The Crypt”, where I remember going to the dacha, watching with my grandmothers in the evenings, where a criminal is also sitting there, and a psychologist is working with him. Here. Well, somehow it attracted me because it was such a very socially significant profession, which, in general, was aimed at a good cause. M. At what age did you decide to become a psychologist? L. Oh, well, it seems to me when I was maybe 10-11 years old, that is, before that I wanted to be an actress, well, let’s say, many girls in elementary school want to, and then I think - no, it’s too fickle, a psychologist - that’s the most That.
M. Yes, there is such a thing. Health? L. Health. Yours? (laughter) M. Yes. What were you even thinking about? L. Well, it often happens to me that I force my loved ones to take care of their health. At the same time, I don’t pay that much attention to it. Because for example, there, I don’t know, I think when I lived with my parents, someone’s blood pressure would rise, and I almost called an ambulance, but they didn’t do it. And in the end I understand that I did everything right. But as for mine, I’m like, damn, again I feel sorry for the money for the dentist. Well, let's probably say here then... (..) Probably a five, that is, somewhere in the middle, because, well, as far as it concerns words, I understand everything perfectly well. But when it turns into business, it is much more difficult. For example, I couldn’t get to one doctor for 3 years. But when I got down to it, I immediately realized that thank God. M. Okay. Job?
Well, not a profession, but probably just a hobby, I just understand that in fact all my energy is there, and when I step over myself, well, I forget to do this creativity at all because I have no time, but I begin to suffer from it. Sometimes I have some kind of blues there, I can even get sick, psychosomatics starts to turn on. Well, in general, these are the moments. M. Katya, tell me, have you ever had a consultation with a psychotherapist? K. Yes, by the way, it took place at the Higher School of Economics.
M. Where do you work? M1. I work in a private children's correctional center as a speech therapist.
M. Yes, but where, as it seems, maybe you or your peers can get information about what independence is and what an independent person is? M1. Well, probably more from... From psychology, but from some kind of popular psychology, because it is more accessible and understandable. And very popular now.
M. And in what terms is health? Can you cure yourself of an illness? Or make an appointment with a specific doctor? M1. No. (laughter) No, just decide that I need to understand on my own that I don’t feel well and go to the doctor and understand which doctor.
M. What is the best way to contact you now, how old are you, what city do you live in now? N. So, Nastya, you can, I’m 22 years old, I’m still studying. Well, that is, I graduated from HSE - Higher School of Economics, Faculty of Psychology, this year, bachelor's degree, and now I am in Omsk, because I myself moved from Omsk to Moscow to study, so, and at the moment I live... Well, I’m just visiting my parents now, but in general I live in Moscow. M. I see. And will you be a psychologist? N. Well, yes. This is by design.
M. Well, about this and that. N. Well, in general, I’m interested in the sphere of parent-child relationships and the sphere... Well, in short, working with children, with teenagers, as if I, which in my experience was not very clear, seems to understand what to do as a teenager, so I kind of do everything... Well, I just like this age, that the worldview is formed there, and so on, and so I kind of want... I would like, for example, to have such a person, to To whom I would come and somehow tell everything without judgment, for example, he or she would simply listen to me.

Well, in general, I would like, for example, to have such a person, if anything, like a school psychologist, so. At my school everything was like that, well, very like that, not humane, that’s why... Well, in particular, that’s why I went to the psychology department. And in general, I want to be more careful with children and teenagers, so that they can be treated, and, well, treat them in general. That's why it's like this.

And there’s this very thing, I don’t know when there will be grandchildren, when I’ll get married, that is, there’s this very insistence on a certain way of life, there are some expectations from the parents, from society, and I feel them, but at the same time I kind of went through psychology and psychotherapy, that is, it all seems to help me, somehow my beliefs, to live according to my own beliefs, and not social ones. But I still feel like it's frowned upon. And for example, when you don’t listen to someone, it’s condemned. There, when you are, as it were, in the minority, this is also condemned, that is, it seems even to me now, this situation, which is the war with Ukraine, that, as it were (.) some people who are vehemently opposed, or something like- then, in general, some people stand out from the masses, they are always, well, they are always perceived negatively, it seems to me. Here. And when, for example, you are different, it’s just somehow scary as if people can be different, and this seems to me to be such a problem that it happens because of this condemnation that you are somehow different. Here.

M. It turns out that the Department of Psychology helped me to be more independent, if I heard correctly? N. Yes, it seems to me that yes, and it seems to me that it’s just plus the people there, my friends and my husband, it’s like, that is, we somehow switched to a more healthy (npt), healthier kind of then the atmosphere, somewhere there it is normal to experience some emotions, well, emotions such as anger or hatred, or... Well, in short, that is, which previously seemed to me that they should not be experienced, or that it is bad to experience them, or something else. Well, in short, there is no plus about family (npt) there (npt), that these are all sorts of non-adaptive patterns and so on and so forth, but how very upset I would have been at first when I took this subject, that this is how bad everything is and all that, but then I thought that, in general, this happens not only in my family, it happens even much worse than in my family, for example, and that parents, they just seem to try as hard as they can, that is, they seem to they also draw conclusions for themselves, based on those who raised them, based on life experience, and what they learned is what they passed on to us, so, and we can then also take it out and pass it on, and so on it will always be like this, and therefore it seems to me that this is, in general, a positive dynamic, that it is, in principle, enough, and not what you say there - this is bad, this is not the same, this is not adaptive, this is something else... That.

And then, and also the fact that I was probably not afraid to take specialized mathematics, because to become a psychologist you need specialized mathematics, and it was like, it was very difficult for me, and every time it ended... Andrey, my husband, he seemed very He’s good at math, but he tried to explain it to me, I didn’t understand, it ended in tears every time and all these tutors and all that, I didn’t like it at all and I literally forced myself to learn it. But I’m like, I want to take psychology, I’ll pass and I’ll never need it again. Well, how important it was for me to pass it well and then I passed it, and I, I had such, well, it seems to me that such a low score for admission, but then I was very... At first I was upset, then I was very I was happy because it was an entrance exam for psychology and that I was going to get into it after all. And I was very happy about this, and my decision, and the fact that I passed these points, and my decision to go to the open day and to enter the Higher School of Economics, although I did not see other universities, that is, I did not go there and didn’t scout anything. I sort of decided that I needed to go there.

N. I would rate it a 10, to be honest, because my parents, my dad, were very much against me going to the psychology department, he wanted me to go to law school, I have a mother, her first education was in medicine, then she studied in psychology. went, but she was like, well, it’s like I

want to be like her, she thought, here. But I didn't go there because of her, I was just interested, as if about the people, there, like with the people, what's in their heads, how to understand it all, that is, it was somehow for me it was interesting about behavior and emotions, so I kind of... And so my mother says something to me: go to these courses, go to these courses, there's something else, and I say no, I won't go, I'll decide for myself what courses I should take, or my dad says, I need to go to a master's program somewhere to become a lawyer, or something like that, and I say like, no, I don't want to, or whatever Here I am this year, well, in the next academic year, for example, I won't study, because, well, like my parents, they would rather have me go to a master's program, but I just understand that I'm tired of studying (laughter) that I can't, (laughter) I need rest. (laughter) And that I just don't understand where, what kind of master's program I would like to go to, I didn't understand, and therefore I didn't go anywhere, and how would I make this decision for myself, how would I want to work and understand maybe there's a better place for me to go. About the job, I would probably rate it a seven, because my first job was in my dad's sales office, that is, my dad, he owns a construction company, and I worked in his sales office. But I wanted to work myself, as if I wanted it myself. And I kind of came there every day, stayed there for some time, they already told me like - go home, but I was like - no, I still need to do this, and I tried to come up with something there, whatever didn't come up with it. Here.
M. What about making everyday and important decisions? Here, in fact, we are, of course, slightly duplicating what we have already discussed. N. Yes, yes, I understood. (..) Well, I think, it seems to me, let it also be seven, because I, well, in general, it seems to me that this is a process that there are no correct ones... In general, I'm kind of really worried about there right, wrong there, or how it will affect others, or something like that... Well, in short, I worry about this in every possible way, and it seems that the more I work there with a psychologist, well, the more I see, that no matter how people react normally to my decisions, the more calm I am about this, the calmer I begin to feel about it, so how would I consider that it's seven, there I'll just be there later, well, what's my attitude specifically towards making a decision , it's very similar, it's quite saturated with anxiety, so I wouldn't say that it comes too easily to me, but I kind of understand that I always make adequate decisions, plus or minus, I just have problems making decisions, that's it. And the choice of lifestyle, leisure, well, right now I would probably say that (..) six, because I think that (..) well, I just feel that I'm a little burnt out and tired after studying there, after school, when things weren't very good there either, at the university, when there were some difficult moments there, that's it. And it seems to me that I just need to recover now, just somehow settle down, that sometimes I get shaky, like how I behave there, or how I need to do or something else, well, like a lifestyle, and for me, I'm just now focusing on the fact that I need to rest, and otherwise be more careful with myself. Here. And about leisure, I don't really like spending time in company, I rather like one-on-one communication, and well, I kind of, (.) well, I don't do any supernatural leisure, like, for example, I knit there, or maybe I have some kind of hobby that is very accessible, well, drums are not very accessible. That is, I disturb everyone, and it happens that they irritate everyone, but as if I could occupy myself with something, I don't feel like I'm very bored, that is, in general, in every possible way... I sometimes scold myself myself for rest, that is, it's as if I can't rest, because I'm not tired and all that. But this is again a balance, there is a work life balance and therefore... (...) Well, about leisure, I meet with friends, I read books there, watch films, some educational things there, well, educational videos on various there platforms. Well, in general, I do, it seems to me, what I want, what interests me, and it seems to me that this is enough. Here. Romantic relationship. (laughter)
M. What do you mean, what didn't coincide? I didn't quite understand? They said that it was possible, but... N. No, rather that they... Well, for example, dad said that you should, for example,

not lie, that it was better to be honest, and then it turns out that he has a second family there, that he him there... Well, in short, (laughter) some secrets from us, roughly speaking, that is, that he says one thing and does another. And that is, as it were... It doesn’t work out, then there is no clear picture, you still understand that there is this duality, and not... (..) Well, what is it... And you begin to think like about some of your actions, that’s it. That’s what I’m talking about, that there weren’t any where, for example, they told me - go and do it, for example, my mother said - go there, do something, take an interest there, something else, if only I had such opportunities as you have, then I would have done this, that, that, but she, despite the fact that she seemed to have opportunities, she practically sat at home with us all the time, that is, she developed, some She worked through her own problems there, with the help of psychology she studied there, but she didn’t seem to really realize herself and it was also like this was a call, like go do something there, realize yourself, but she herself didn’t do that. And it was as if I was expecting that if my parents said something, then they would do it, but that didn’t happen, and so I had a question, like, how can this be, like, people say one thing, but do another, And I had such a conflict. Here.
M. Do you have any hobbies? Besides work. A. Well, probably yes, part of it is the field of education, psychology, everything connected with tutoring, with... (...) Drawing, exhibitions, studying, well, I don’t know, the work of artists, this is also connected with literature, Let’s say now, the main focus of interests is the book (npt), Annie Lee Lang, which describes art, artists, and, in principle, the concept of loneliness. And how artists reflected it, how it is depicted in culture, in what images. Here. Something like that. M. Great.
Well, that is, yes, you will go to medical school, Yes, doctors, doctors, this is always needed, well, they are like that, one of the pillars of society that will always come in handy, you will always need to treat people. Here. But it is very difficult to find, unlearn, firstly, spend a lot. And then, I couldn’t answer why I needed this, so somehow, not finding answers, I slowly walked away from it. Well, it’s a plus due to the fact that then my mother, grandmother is a pharmacist, my mother is there in the field of obstetrics, and somehow, knowing this whole kitchen, I didn’t go there, so this is also an important, it seems to me, such a stage, when I reoriented myself, but it was as if it was unclear where. Further, my interests developed - social studies, history, well, closer and closer and closer I seemed to come to education and the humanities, to the humanitarian sphere.
A. So, training and professional development, I already said that. Health choices. If we talk about the second point, then for the last year, but probably I have had more changes in six months, because there was a change of place of residence and somehow I concentrate more on it. Because the choices that were made in the previous six months were not in favor of my health, but there the only important option, or rather there was a choice, was to go to a psychologist. To deal with this destructive story with the leader, with (sigh) (..) change of position. Here. And if you look more towards the physical rather than the psychological, then this is probably a choice in favor of taking care (.) of some kind about yourself, to searching for, well, understanding in general your health there through the body, changing a little nutrition, attitude towards sports, and so on Further. Well, something like that, I returned there again, to proactive history. Job. (..) One important thing, well, it seemed to happen to itself, it so happened that for a new position, again over the last six months, first there was the building of horizontal communication, despite the fact that there is a manager and subordinates. Yes, I am the director there, but these are still subordinates, a turquoise organization was built. There was this, well, how it was clearly articulated, said internally in the collective, in the team.
M. Basically from the elections. A. Well, after all, what is still very much unclear and difficult for me is the case of accepting responsibility for the whole, the position of a manager. M. Yes, I remember you talked about this in great detail. Yeah, that's what happened. A. This is really very complicated... Well, it seems to have been partially worked out, this situation, in psychotherapy,

but not yet fully resolved on some emotional level, it also seems to me that this has created some kind of trauma, (laughter) here. In terms of what was wrong, why was it wrong, right? M. Yes, why the wrong choice, do you think? A. Because, again, it was probably from the position of - well, who else? Well, very often I have such a choice when there is no other outcome, well, that is, some kind of critical point. And only then do I, as it were, completely regroup, into some kind of monster, make this decision, well, that is, I say this - yes, I can, we'll do it this way, I'll pull it off, but somehow, as if at a critical peak, I take upon myself the whole responsibility that is possible. Well, as if this were directly noticed, and then it usually works out. (xxx) But in this situation, which was and where my choice was, as it were, wrong, I underestimated it. Well, that is, the gap in this critical situation, the gap between what I can do and what definitely won't work out, it, well, happened more. Revaluation. (..) Here. M. Yes, but how do you feel about this situation now? What thoughts or feelings might arise about her? A. Well, it's cool that it was the first thing. Second, an important decision was made that in the next couple of years, I don't know, I won't take a management position at gunpoint. Not yet, well, no, I won't go there again for now. Just now I started, well, I kind of started to sniff out, if possible, from this state when you, well, got a very strong electric shock, well, you just intuitively won't get into it anymore. Here.
Another important choice, which in general, if in the future 10 years, then (..) it's probably closer to the family. Well, that is, as it were, a choice in favor of (..) building long-term relationships, and another related choice - health. Well, that is, as if I don't want to waste what I already have, because our body is only depleted, but I want to somehow fill it up, maintain balance, different areas of life that affect health, ultimately well-being, and the feeling of happiness when you feel good. This is a choice in favor of, well, taking care of yourself in different areas. Well, that is, starting there, I don't know, with some kind of tests, check-ups, regular support for physical fitness, psychological health, such a minimum of some kind of psychological hygiene, then yes, this is directly connected for me with family relationships, because in Ultimately, if you make a choice, then for now I have the feeling that I am not childfree, but I still want some kind of (.) continuation of the family and continuation of the clan, if you can call it that. Again, I want healthy children, a healthy child, and this means that I must be healthy. And the other person must be healthy. Therefore, this is a long-term construction, with certain resulting requirements for a person. (..) And probably not by requirements at all, but by similarity of beliefs. When they look in one direction. Well, probably for 10 years, well, another important one, this is first, in the short term, somewhere up to 5 years, a period of time to take, then this is a set of expertise and experience, packaging it and going out not to work for someone, but for myself.
K. At first I wanted to go to become a military doctor, but in the tenth grade, when I found out that girls were not accepted into the military academy, I decided to go to the Lev Nikolaevich Tolstoy University, because a friend of mine studied there, and she responded very well about this university, I decided to go there too. I just have a similar direction, working with children, with youth, and this is closer to me. Well, it just happened that I love chemistry and biology, so I decided to go there.
M. You can go straight through the list, you don't have to arrange them. K. Training and professional development is... Probably there will be seven, because (..) you always have to learn everything, and sometimes it's very difficult to allocate your resources and brains, (laughter) let's put it this way. And do one thing. Health is five, (laughter) it's either there or it's not, work is also six or seven, because... M. Wait, let's go back to health, making specific decisions. In terms of health. K. Regarding my health, everything seems to be fine, I feel good, but sometimes there are times when I get sick, (laughter) Covid and so on. And I try to recover as much as possible. But if I get sick, then I don't do anything, it's immediately minus work, (laughter) minus friendships, only

the place of residence will be there then. (laughter) At maximum. M. What about choices, such as choosing which doctor you need to go to, or even understanding whether you need to seek help or not? K. Choosing a doctor, I don't know. I somehow... M. How difficult or easy is it for you? K. No, it's easy for me to choose a doctor. I just see, either from reviews, or using word of mouth, that this doctor, we went to see him, he's good. I'm so good, I'll go to him too and everything will be fine. In principle, this is how it turns out, so I don't have such a difficult choice here to choose a specific doctor. Well, usually, I just probably didn't get sick with anything serious, no matter where it depended on me... The doctor's choice is between life and death. Therefore... M. In general, you said that you rate your health as a five, then what is the difficulty in making decisions regarding health? K. Health, difficulty making decisions, (laughter) giving injections. This is where it's difficult. (laughter) Or force yourself to go... It's just that when you're sick, you don't really want to go out. M. So it's difficult for you to understand whether you need help or not? K. Yes, I can't, it's sometimes difficult to assess the extent of my illness, it just seems to me that even with a temperature of 38 you can still do something there and at work, everything is a doctor (NPT) in principle (NPT) and you can live.
M. And now I will ask you to tell me about one story of a successful choice, and also, please, in accordance with this layout from the chat. K. Successful... (..) Now, we need to think. (...) Something... (...) I thought, I think it's good that I changed one job for another. Before that, I worked in another place, not even at a university, but in a clinic, well, in a hospital. And this is very, well, at first it seemed to me that this is very cool and great, because the equipment is new, but then I realized that this does not give me any career growth and if I stay in this position, I'll be there (npt). Then, but this also happened at school, then they gave me... Work, a feeling of extreme fatigue, every day. And it's like (npt) Groundhog Day. And I realized that this had to stop somehow, otherwise I would simply burn out at this job. M. So you also seemed to be guided by your emotions? K. (..) Yes. M. It seems to me that you have someone else there? K. Yes, now there are students again, we should have opened another classroom for them. All. (..) Well, it seems like I told you everything. M. No, more about feelings and thoughts? K. Oh, (sigh) feelings, then this situation, (laughter) it seems to me that this whole situation sucked out any emotions and feelings from me at that moment, because working in a hospital is very energy-intensive. And now I'm looking at it too, the same thing, the only thing I've gained is experience, put it in my work book and just to fill out my resume. M. Tell me, Karina, how do you determine which choice is successful for you and which is unsuccessful? How do you separate them? K. (..) Successful, if I feel some kind of return, I feel needed. And unsuccessfully, if I feel some kind of return, I feel needed. And it's unsuccessful if I feel like I'm wasting time and wasting it.
D. It turns out that at the age of 11 I was told that I had the fourth degree of scoliosis, idiopathic, and that this could no longer be treated, perhaps I should have started going to some massages there a couple of years ago, doing something, but I had already encountered with a severe form of this scoliosis, and they told me that... Well, I was again given a choice, I remember how my mother seriously decided to talk to me, at the age of 11, that Dasha, you have such a situation, you can (..) live with this scoliosis, that is, it's like, well, I would have developed a hump, very strongly, it had already begun to appear, or I would have to undergo surgery, and they would put me in a metal structure for life. Into the spine. And when you're 11, you don't really understand that they're going to do something to you now, and you'll have to live with it, well, for the rest of your life, and I made up my mind, and basically, my mother told me that I walked this path very steadfastly , and lay in the ward after the operation. The girls who were 10 years older than me were there, they cried, it was hard for them, and my mother said that I went through it all with courage (laughter). Here. This is probably also an important path. M. Yes, I really sympathize, well, it's really a choice, and it's such a significant one, and I think that you are probably faced with some of its

consequences. Does this have anything to do with tennis or not? It seems like... D. My mother thinks yes, that it’s because of tennis, but I think that... (..) Perhaps, but there is a reason, there is a reason. Perhaps this somehow prompted, well, aggravated the situation, but obviously it was not the reason. Most likely it’s something genetic, perhaps I had crooked feet from birth, and they tried to correct them for me somehow, here. Did not work out. And you understand, yes, that when the weight is unevenly distributed, then all sorts of scoliosis results. That's probably because of this.
Then health. Well, from the last one, probably... But this is probably some kind of funny situation, there’s not much choice here, I, I felt bad, not so long ago, a month ago, and I called an ambulance, I didn’t want to call it for a very long time, because that it always seems to me that my situation is not serious enough to call an ambulance, for someone it is now perhaps more important, more necessary, so. And when we arrived, it turned out that the situation was very serious, I had some kind of intestinal inflammation. And they just gave me a choice - to be hospitalized or not. (laughter) And I, too, somehow without really thinking, decided that it was necessary, let them check me and all that. And it was also an unpleasant situation that I was out of work there for a week, and I was ashamed, but I thought that if this, (..) if I don’t get cured now and then it all somehow piles up, and it will be like like some kind of lump of snow, just like that. Well, in general, some more pain will be added, and then the consequences will be more difficult to deal with than now. I hope I made myself clear. (laughter)
When I went to the hospital, I didn’t immediately tell my parents, only when I felt good there
How could it be, what else? (laughter) (..) I think, I think. (laughter) I still have something from childhood, like, let’s say, principle, choice, I never drank alcohol, well, cigarettes, that’s understandable. And even when I... Well, that’s how it is in Russia, they are always trying to offer something to you, and as if in theory, the majority drinks and considers it normal. (laughter) Yes, and when you don’t drink, in general, they are all so surprised, well, come on, well, try it, (laughter) but I always had a clear motive, this has been since childhood, that I am not going to drink and I will never. Well, as an example. Well, even when, relatively speaking, I was moving into a hostel, the neighbors somehow, well, there are three of us, and they suggested how to celebrate a housewarming, they asked me - will you? I'm like, no, I won't. (laughter) And they were like - well, okay. But I always hear this phrase - you will start someday anyway. That is, I don’t drink at all. Not for the holidays, well, it doesn’t matter. Just never and not at all. Here. This, so to speak, (sigh) is my principle and choice. (laughter) (...) So, I don’t know how many minutes have already passed there. (laughter) It just happens, you don’t remember everything right away. (..) Damn, I guess I, I can’t think of anything yet, it’s hard to come up with, that is, come up with.
M. But you also said that mom changed a little, softened her views. What does this have to do with? L. Oh, (laughter) with her life experience, probably. Well, oh, in general, yes... (..) Well, she looks, reads these psychological books, and something else. In general, it develops itself, so we plant it like that. (...) Because she doesn’t like her life, she wants to change something in it. M. Lisa, was it really noticeable to you that suddenly she changed there and became softer? Or how? L. Yes, yes, she just talks a lot, and since she has no friends, in our city, well, they were, they all went to Tyumen. (laughter) And a new friend appeared, and she also left for Tyumen, (npt) And so it always turned out that she was talking to me and all these topics, well, I don’t know, she talks a lot in general, as if about herself, then about how she lived there (sigh) then she speaks about herself, about her changes. Or it says that you can reprimand me there if you see that I’m going too far there. Well, in general it was not difficult for her. (laughter)
..) Rather, this is how it is, somehow significant, this is health. (...) So, health. (laughter) (...) I would probably classify it more for me as some kind of sport, some kind of sport, (..) they are connected. And, (npt) I also said that alcohol, (laughter) (npt) let’s say, refuse (npt). That is, I don’t

want this one (npt) yet, but even before that, this is the last one. And as for sports, I, I have a new kind of sport, that is, always, (NPT) development, I liked it, there was a subscription for eight classes. This is something new for me. But as they say, well, jumping on a trampoline, doing (npt) or something like that, in my opinion. (npt) Then work. Work, well, work, here I chose, “Yandex.Food”. I was choosing between Yandex.Food and Delivery Club, well, it seemed that in Yandex.Food, well, how to work there, on the contrary (npt), with large orders, but since I could, I had initial information... M. So, now everything is turned on, now, I hope, everything will be heard well and there will be no interruptions. L. Well, yes, I said that I was choosing between Delivery Club and Yandex.Food, and there was more difference, that is, I went to Yandex.Food. Oh, and then I also worked at Pyaterochka, but I initially went to the order area, since it turned out to be there from morning to evening, and I needed (..) evening part-time work, or for a number of hours. But, I said, they offered me a cashier job, and I, I had a choice... M. Yes, Lisa, something went wrong. Yes, about the order picker, and you were offered cashiers. Here in these words. L. Yes, I had a choice whether to agree or refuse. And I thought, okay, I’ll agree, I’ll try, why not. Although it was a little scary and exciting, in the end everything, so to speak, worked out, I was trained and I worked calmly, so to speak. Here. Later, but then I had to leave, so I quit. But I warned that I wouldn’t be around for long and, in principle, (laughter) since they didn’t quite suit me, (laughter) and it would have been a choice in general, (laughter) probably to quit. And, so, (npt) in my opinion, (..) I would roughly give an example, like choosing a vacation. Since it’s difficult for me to get from Rostov to Nizhnevartovsk, so to speak, at the moment, since the airport is closed, and you can get there by roundabout routes, but it’s expensive. Because from Moscow (laughter) to us one way it’s 10 thousand, but I just had a direct flight, I could fly (npt) when I was lucky in principle to get it and when you already flew for 3, and for 5, and for 7, then you don’t want this, well, yes, you don’t want to go one way for 10. But in principle, it happens, it’s a lot, so to speak, well, I would come to my city, but there’s not much there, there is nothing to do, and in general, my mother and I agreed that we would meet in the summer, but not in the city, but somewhere, so to speak, for us on neutral territory. Well, we went to Sochi, that is, she (npt) flew, and I had to get there by train, and... (...) Well, we generally chose tickets, well, where is the best place to go, we chose these ones, where Sochi is. But since I was already there, a year ago, for a few days, it was somehow very, there is such an example, but... (sigh) So, (..) friendly relations. (...) Well, choice. (..) Well, here, probably, who would you like to be a friend, (laughter) and who would not. Don't know. Or, let’s say, where there is a person like this, I would like someone... (sigh) (...) So... (laughter) In general, they offered me to take a walk there, and I, (..) let’s say, I didn't want it in general. I don’t like this person, so I don’t want him to be (laughter) my friend. And you probably... Well, I kind of refuse. Don't want. Don't know. (laughter) What’s a better example to give, honestly. Well, I don’t know how I could have one friend here, (..) I don’t even know. I'm moving on for now. Place of residence, regarding the place of residence, by the way, I live in a dormitory, and initially I didn’t want to live in a dormitory, because, well, it seems to me that here, well, all sorts of events are held, as if there was a place to sit, let’s say I had to film apartment or something like that. And plus I have a hostel, it’s very comfortable, well, I just won a competition, (laughter) and the hostel in which I live, it’s like, well, it’s an apartment type, and that’s why (laughter) it’s here for me in general, not only that , that I’m like this in a hostel, where I have connections, but it’s also almost home. (laughter) But, of course, I didn’t know which one I would end up in, but initially I wanted to go to the hostel, so that there would be all sorts of these events in which I could participate. That is, renting in one there, in an apartment, is not interesting, it is boring. Here. (...) Everyday and important decisions. (sigh) (..) Well, about important decisions, everyday ones, probably just like where, where to go, if everyday, where to go, to have fun, to see. Well, also, for example, I took part in the race there in May, for me it was such an important

decision. In such a major event, because it is not held here, and I have never participated. The Russian Federation race, which took place in May, I... Well, it costs money, and you kind of have to decide on it. (laughter) I took the plunge, bought it and didn’t regret it. And also (..) I liked it, I finished the race (npt) I also decided, I realized that I liked it, that (laughter) I need to train for it, and that, well, more.
M. Yes, but do you have any decisions in life that you consider unsuccessful? L. Solutions... Health related? M. In general, not only related to health, but are there any choices that you consider to be completely unsuccessful? L. Yes. The elections were unsuccessful. Well, (..) probably yes, yes. (laughter) But for me everything is connected with sports. (laughter) In the eighth grade, no, not even in the eighth, but earlier, in the sixth grade, they offered me to go to... (npt) To the All-Russian Olympiad for schoolchildren in physical education, but I refused then, and in the seventh they offered me, but I She also refused, or rather, at first she agreed, then for some reason she refused. In the eighth, (sigh) I also refused, and in the ninth, (laughter) and only in the tenth I came, and even then I agreed. And... I regret that I refused before, because I really wanted to go to the final stage, that is, to Russia, which took place then in the eleventh grade, in Kursk, that is, you go through the regional, well, first the school, then municipal, regional, and final. And (laughter) in general, two years of preparation is not enough, and if in the tenth grade I failed there, then in the eleventh, in the region, although I was a prize-winner, I took first place, but I was 17 hundredths short of Russia, and I was very I wanted to. And in general, I regretted then that I had not agreed to another school earlier. And in general, yes, and the fact that in addition to physical education there, let’s say I would also take part in mathematics, I somehow even thought about it... Well, I didn’t think about what the Olympiads are like, and what they give, and what in general is that? In general, then it was a bad choice that I refused. (sigh) In this regard. M. So you consider those elections unsuccessful, where you missed some opportunity? L. Well, probably yes. M. But out of these several situations with physical education Olympiads, which of these, well, which of these situations is the most unfortunate, in your opinion? L. (...) Well, what’s the worst? (laughter) The situation, well, (laughter) I didn’t have enough points, (laughter) for Russia, these hundredths... Hundredths, well, it’s just literally the whole point, or (npt) in gymnastics, or a second of running. (laughter) M. And you feel like you’re blaming yourself a little for not pushing through? L. Yes. I just wanted, and even more than to pass the Unified State Exam well, that is, (laughter) well, damn it, I still can’t come to terms with it. It's a shame. (laughter)
So I realized that as a result, you have not yet enrolled in any specific business specialty in order to do quests. But as for sports, why wouldn’t it become some kind of professional field, well, in which you would become, well, a professional athlete, why not this? L. Yes, it’s interesting, I mean, in general it’s, well, firstly, we don’t have that many sports in our city, and if it’s all over there (npt), then basically, in principle, then in our regions... Well, even if not only all kinds of sports, Moscow has everything, I recently made such conclusions, but in the regions, in one there is no such thing, in another there is no such thing, in a third this is not the case, and since we have a lot of sports, I started somewhere with basketball, then I liked it, before I was banned anymore (npt), then I went to sports tourism for 2 years, it’s not like It’s just mountaineering, but that’s the point, there’s a program there, on the ropes like that. In principle, I liked it, but for some reason I got tired of it. I don't know. (laughter) Honestly, I got tired of it and just quit. (laughter) The same about athletics. And athletics, it's even more boring. Well, again, it’s a cyclical sport, where you only do the same thing, and that is, we only have running and nothing else. There is no throwing or jumping, just running. And I'm not interested in running. (laughter) It’s the same thing, in general, I’m truly not an athlete. I understand this because, well, I don't like it. Well, I don’t know, and somehow other sports, I didn’t try much, then I just somehow in the ninth, or even the eighth

grade, (laughter) forgot that I was an athlete, and to some extent a sport was absent from my life, and then, in the tenth grade, I somehow remembered this, and here, when we were just preparing for the Olympiad in physical education, we have, in addition to, well, theory, gymnastics and running. And since I liked gymnastics, it's clear that it's not like real, professional, sports gymnasts, there's a lot of other things, and I (laughter) by the way, I regretted that damn, it's a pity I don't do this, but they only take from childhood. And my mother told me that she somehow brought me when I was 5 years old, but they refused me. But I don't remember this, they kind of told me that I was too big. (laughter) But I kind of liked it, and because of this I kept looking in Rostov for some kind of acrobatics for adults, gymnastics for adults, that's it. And trampoline jumping, but it is combined with gymnastics for adults. But the problem is that damn, it takes a day. (laughter) Well, yes, sort of. And so, well, I didn't manage to go to any professional sport, because I couldn't find one for myself. M. That is, if, perhaps, when you were younger, you would have been sent, well, sent somewhere... L. Yes, perhaps.

Z. Yes, yes, yes. Here. Choice of lifestyle and leisure. I really want (laughter) for this area of activity to change for me, because over the past year, all I did was study. True, I don't have time for anything else, I didn't have time. I'm trying to change, I'm trying to change this moment, I really want to do... Well, in general, I would like to do yoga, go to group classes, I realized that I really like it when (..) probably a new team appears, and there are new people, meeting new people, it's very interesting, it's something new and unusual. I would really like this area of mine to improve, because again, all I'm doing right now is learning. And this drains you and you need to recharge somewhere. Where can I get it? Well, I realized for myself that I would like to go to a slightly different space, to other people, and replenish my resources with the help of sports, yoga, etc.

M. Okay, let's now take a closer look at your elections, and now I want to ask about the elections that you may consider unsuccessful in your life, were there any? And why do you consider them unsuccessful? Z. (...) Yes, it was a bad choice in terms of health, so I already told you that I turned to a surgeon, and... Well, I regretted it. I had an operation that was not required at all, that is, in vain, they completely removed my nail plate just like that. Why this was done is unclear. When I came to a highly qualified specialist, a podiatrist, they told me that this was a complete disgrace, it was impossible to do this, and now we are correcting this whole problem. Here. That's why for me this is such an important choice, a health experience. And now I think this is very important. When they wish you health, now I don't just, well, health, happiness. Happiness, health - yes, this is what I need. (laughter)

M. Great, great. Yes, it seems we have now discussed some better choice. Maybe you can remember, for example, how a bad choice was made? For example, with an ingrown toenail, maybe you can remember your thoughts and feelings while you were going for surgery? Z. Yes, yes, of course. At that time, I was at home, not in Rostov, and we went to the hospital. In general, it turned out that I was immediately sent to a surgeon to solve this problem. I come to the surgeon and he looks at me slightly and immediately says - that's it, operate. I'm like - what? And I had never had any health problems before. Everything was fine, no surgeries at all. Well, it was a shock for me, I was admitted to the hospital right away, that's it, they left me, my parents brought my things, and the next day I was waiting for the operation, so. So it turns out (..) day X comes, the time approaches, they take me to the operating room, perform the operation, and the worst thing was that after the operation on the nail, well, it turns out that the nail plate was completely removed, and a bandage was wrapped around this, on this skin. It's very tender and soft there, and it's still a little unhealed, because an inflammatory process began from this nail, it was painful. Here. And they wrap this bandage around me and say, well, that's it, now lie down and rest. Everything is fine. The next day it was necessary to do (..) soak the nail in a special solution so that

it, the nail... The finger in the solution so that it heals. Here. And for this it was necessary to remove this bandage. I start to unwind it, but it won't come off. He was completely stuck to this super soft, unhealed spot, it was just terrible. The time was probably just right... (..) Well, there was a day, I was sitting, I tried to soak this bandage in different solutions, a couple of hours passed, it didn't help, we just started trying, well, tearing it off a little, that didn't work either helped. It was terrible. We probably sat like that until very late at night, with the nurses, they tried to take it off for me, nothing worked, it hurts, it's a super soft, tender place... In the end they told me, let's do this to you. In general, well, it's like a bandage, it's even, applied to this finger, here. They cut off the entire part of the bandage and left only the piece that was glued to my finger. And in general, they tried to remove it with tweezers, pulling out each one of these... (laughter) A piece of lint from the bandage. It was terrible. It's just a nightmare. This also didn't help, and I'm already in tears, of course, all upset, nothing is working out... I'm also a very suspicious person, (laughter) I've already thought of a bunch of things, that's it, now he's dried up for life, I'm like with him I'll live, (laughter) upset, that's it. Well, they told me to wait for the doctor in the morning. But that's all that was left to do, and I ended up sleeping through the night with this bandage, and the next morning the surgeon came and in the operating room he made me some kind of, I don't know, glued some kind of plate on me, and just tore it off with a very sharp movement. Yes, from this soft place, they simply tore off the completely dried bandage. It was very painful, oh well, and it was all bearable, it all went away, that's it. I thought that was it, my problem would end there, thank God, everything was cured, everything is fine. So six months pass, the nail grows back, it grows very ugly, ugly, really, ugly, yellow, clumsy, all like this... I turn to the surgeon again, already here in Rostov, he says that Well, we need to do an operation and rip it out again. I think - what kind of person are you, really? Here. I'm thinking, well, are there any more humane methods of treatment, maybe it's possible to even it out somehow? They tell me that such treatment is done only in private clinics, this is a paid treatment, but they said - try it, maybe it will suit you. And I thought that I didn't want to suffer anymore, I wanted to know something specific from a specialist, some exact answer as to why this was happening, not just to rip it off every time, but to find out the reason. And what to do with it. And I turned to a podiatrist, in a private clinic. And now this doctor and I are still aligning my nail plate, everything is fine. Here. Of course, I was upset that I had experienced all this, but probably without this I would not have understood that it is very important to go to, well, really good doctors and it is better to pay a little for your treatment, and be sure that they will really help you and correct you your problem, than every time, as they suggested to me, tearing off a nail. Here. M. What did you think, what did you learn... What do you think, what did this situation possibly teach you? Z. (..) Taught? Well, probably after all (..) until this moment I didn't really think about the qualifications of doctors, that is, you come to the hospital, doctors, they're all doctors, they probably know everything, smart people. And now, you look at it from a slightly different angle, well, at least I do. I pay attention to certificates, I pay attention to where I studied, what merits I have. Well, in general, for qualifications and I think this is still very important. And also thank you that we live in the modern world, we have the Internet, we have the opportunity to look at people's reviews, at a certain doctor, for example, or a clinic. And now, depending on the reviews, you can make a decision whether or not to go to this specialist. Here. Probably, after all, yes. The situation taught me to take health more seriously, this is not a joke, and to pay attention to who will treat you. This is also very important.
Then I went to school, and in the first grade I did synchronized swimming. All first grade. And then, the workload became so heavy that my mother suggested that I either change schools, or that I study in the second shift and be able to devote all the remaining time to training. Or stay in school and quit swimming. And she didn't decide this for me, she said that it was entirely my choice. And

then, during that period, I decided that I would quit swimming. And I'll stay in school. Well, it seems to me that then I chose friends and some kind of social life, it seems to me, at the age I was, as if friends were more important, and sports had never been some kind of meaning of life for me. I went, I basically liked it, but he was never a priority for me. But now I think that... Now I don’t think at all, but I used to think that if I had stayed, I would now be some kind of master of sports or take part in competitions, be an athlete. And life would have turned out completely differently. But nevertheless, I never regret the decisions I made, in any way. I studied the same way, in the same school from first to eleventh grade. (..) I was like that, not very much, but that is, not a completely withdrawn child, but also not one who directly craves attention, that is, I was afraid of public speaking, but at the same time I communicated well with everyone, well, I was the kind of person who He seems to communicate with everyone, but only a few people are good there. But everyone treated me well. Then, the elections, where... In principle, even when I was in school, my parents always put the whole choice on me, they didn’t decide anything for me. Which sections should I go to? I also tried a lot of different sports, only because I wanted it myself, and I left the same way because I wanted it myself. And it seems to me that this... Well, to some extent it’s probably correct, perhaps those who... Those parents who want their child to actively engage in some kind of sport, who have their own ambitions, they somehow insist, but I, on the other hand, have tried a lot of different sports throughout my life, and I realized what I like to do. I still swim, for example, like it was with synchronized swimming, I realized that I like swimming, I still go to the pool. And when I started doing athletics, I realized that I like to run, but kind of (..) not... But I only like to run, for example. I don't like everything else we did. And I just left, just doing it for myself. Well, that is, it seems to me that we need to somehow give the child a choice, although they already asked me then... (...) What... (...)
M. If in terms of the degree of difficulty of the choice, how would you rate it between one and ten? Well, you actually talked about a series of elections, but on average, how do you evaluate your passage of these forks? You chose between a bachelor’s degree and a bachelor’s degree, this advanced one, you chose not to work there, but you chose to work there. J. It seems to me, seven, approximately. M. Thank you. J. Health, I don’t even know what I can say about this. Because I’ve always tried somehow... (..) Well, I just have very good health, some kind of good immunity, I’ve never really... In terms of paying some attention to my health, I make a choice in favor of some kind of regular check-up. I go to doctors, donate blood, take vitamins, and exercise. I'm trying. Sometimes I get very lazy, but I’m trying to instill this habit in myself; in fact, I still really want it to be some kind of regular. Also, I go to the doctor, well, that is, I don’t have the position where I will never go to the doctor in my life, like some people, they don’t like it at all, no, I’m somehow very sensitive to myself, to my health. It’s better to go get checked there, once again, than not. M. And how would you rate the severity of choosing these, developing habits, even laziness, but it’s important to go to the doctors, get checked, get tested, how easy or difficult is this for you? G. Four. M. Four yeah. Thank you. J. (..) And work, for me it’s a little bit like that, for now... (..) Well, I’m still studying there in general, what suits me, what doesn’t, I don’t understand yet, I generally... At the fact that it seemed to me that I was a rather diligent person and that I could somehow do some monotonous work for a long time, but it turned out that not as long as I thought. And I’m still feeling out in general what type of activity I’m comfortable doing for a long time, because somehow, well, in my head it’s as if there should be one activity for, well, a long period of time. But I always somehow get tired after a couple of months, I need some kind of change of activity, or some kind of development. That is, right in one place, something is the same, like accounting, for example, or in a bakery, doing something the same every day, I start to get tired and fall into some kind of despondency, which is like this it will happen, I immediately have some thoughts that it will be like this all my life. It's the same there every day. That is, I also realized for

myself that in this regard I need some kind of variety, and I want... Well, my dad, for example, never worked in an office, maybe that's why I subconsciously somehow chose a profession similar to his . Because he travels around objects all the time, doing something, something like this is closer to me, some kind of active activity. Even in terms of studying, I could never sit like this and constantly listen to something and that's it, that is, when we receive knowledge, we immediately put it into practice, some kind of more creative training or something. And apparently I'm inclined to have a job, I also need some kind of work, well, more active or creative, I don't know. I'm still searching. Choosing a job is actually quite easy for me, I don't know, maybe (..) 6... I somehow start easily, finish easily. (...) Well, six, probably not, let's give five. Let's put it at five.
J. Because it's such a difficult topic, because I still... (..) Well, that is, I still live, for example, with my mom and dad, I communicate every day, I'm still building some kind of relationship with them. I myself go to a psychologist, somehow I'm already trying, from the side of some more... Well, not a teenager anymore, but some more adult person, I'm trying to look at any situations from both sides. Both from my position and from the position of my parents. Before that, at school, I always had some kind of very blaming position. That is, I thought that the way I think is exactly like that, and I never really wanted to consider any other points of view on this situation. Not how I see it from my side, but how other people could see it. Well, my parents. And that's why it's always been very difficult for me to understand them. And I was like that, a rebellious child inside, that is, on the outside I was quite calm, but I always disagreed with them, with something, I always somehow kept my opinion about you. And now I'm just trying to take it easier, it's easier to accept that someone may have a different opinion and that's normal. Other views on some situations. I don't know, I don't understand at all what choice means in terms of relationships with parents, but (..) in any case, it was always somehow very difficult for me, I... We always had good and bad, then good again. It was such a thorny road.
M. I think that this decision is also very difficult, and somehow very worthy. But you speak warmly about your father, and about your similarity with him, in principle, in part. J. Well, yes. Well, I went, as soon as I had the opportunity, I immediately went to a psychologist. And I still go to him. We have a free psychologist at our faculty. They did it for us. This turned out to be a very popular service. A lot of guys go. And this is really very cool. Yes, I'm working on my relationship with both my dad and my mom, how it all affected me, and where in this whole situation I am, and where are my parents' opinions about me, (npt).
Somehow it's always been (...) hard for me to open up. Yes, it's probably some kind of, well, childhood trauma. I'm still working with this, with a psychologist, and it's hard for me to open up to people, because it's as if... (...) It's as if, when I open up, I start to take everything too close to my heart, and then it's very difficult I'm experiencing this. Therefore, in terms of friendly relations, choosing some new friend for me, well, probably an eight.
Now, I have some kind of frantic energy, I want to meet someone all the time, learn something new, go somewhere new, to theaters, museums, concerts, walks, etc. whatever. I feel like I'm being filled up by this, I don't know. So I went, when I went to a psychologist, somehow after that I became more open, and then people themselves began to reach out, after that. (..) Oh, romantic relationships, that's also a ten. I have a very hard time with this, because it's also about opening up, you have to open up to people, and at some point I start to quickly, quickly close myself off, as if when I understand that this is some kind of... Well, it's not like that anymore, (..) when I realize that I am becoming a little vulnerable, I, (..) my brain seems to automatically begin to push away, that is, it is so much so that I even (..) at first may not notice it, and only when I analyze it later, that is, everything is so quirky that it seems to me at the moment when this happens that the reason that I chose is really important, but in fact then I understand that I just had some kind of fear and

(..) I just, well, sort of decided to run away. Now I’m actively working with this, but this one is hard. For ten.
Well, firstly, I didn’t want to lie, I never had a need to lie or hide something, and (.) secondly, it’s always obvious from me if I’m lying. Since I apparently didn’t learn this, my mother always knew that something was wrong, even if I kept silent about something. She says - so, do you want to tell me something? She always (xxxx) saw and read me. Right always. Well, at the age of 12 I had a rebellion, at 13, maybe it lasted until I was 15, and then it started that I don’t want to tell my mother this, she will scold me there or she forbade me to do this How can I tell her about this? After this, this situation, which lasted maybe a year or a year and a half, with that boy, no more situations like this were repeated, that is, this was enough for me, I completely lost all her trust, it didn’t suit me, I stopped feeling this connection , so that I needed it, that is, she stopped believing me, stopped supporting me, it became hard for me. And this was not a reason for me to stop communicating with him, I just matured a little and realized that he really was something strange. What did I even find in him? (laughter) Here. And then I realized that I still regretted what I did. And from that moment on, I no longer deceive in any way. (.) And that is, my mother and I discussed this, I say, like it happens, my girlfriends drink, and she says - okay, like if they drink, then wait a little, from 16 I will officially allow you, come on, (. .) whatever... (..) Only you will warn me, write and, as it were, you must come home, you don’t have to go anywhere, and so on. Well, okay, that is, there were no secrets from my mother at all.
M. What if you give an example of some recent situation where you acted completely independently, and this situation stuck in your mind? K. (...) Well, for example, now my decision was completely independent - to get braces. And yesterday I installed them. That is, my mother told me that it’s your business, do what you want. I ask her: Mom, is it worth it or not? She says do what you want. But she also seems to already understand that I often ask her for advice, and please think with me, And she seems to be starting to quietly separate me too, saying that please make the decision yourself. That is, I have personal accumulated money for work, and I myself, first I had my teeth removed, then now I got braces, this is completely my decision.
M. Yes, in general, undoubtedly. Are your relationships with your parents too distant, distant? Or somehow you quarrel, or... D. Sometimes they are very close, and most often these are just situations when we become close, which is extremely rare now, they make me feel guilty that I’m so bad and I offend them. But in general, well, I don’t know, they certainly didn’t do much harm to me, but purely morally, they did. That is, there is some kind of financial support at all. (..) But psychologically, of course, it’s very (.) difficult. (..) Here. Varya, I'm a little busy. They are shorter in the kitchen. OK. In short... (..) So what should I tell you? M. I asked about relationships, about support or about conflicts, well, a little, in general terms, in order to somehow understand. D. My father drinks often, and as if before it didn’t directly affect him very much, it was limited to the fact that we were walking along the streets, he was drunk, and just did all sorts of bullshit, I was ashamed of him. And then, in the ninth grade, when I began preparing for the Olympics, I studied them seriously for the first time, I almost didn’t leave the room, because I wanted to seriously prepare. And my parents said that you sit so much and seem to be moving away from us, well, they fought because of this. And my dad also came home drunk, and my mother asked me to sit with him in the kitchen and talk every evening, he drove me to hysterics, and in ninth grade I can’t even imagine how I pulled it out, but it was... Emotionally it was difficult . Here. And with my mother, well, I don’t know, in general I’ve always been my father’s daughter, well, I thought so, but for some reason I don’t have a particularly good relationship with my dad now either. Mom sometimes, sometimes she somehow tries, like, when she sees that I’m really really bad, and that’s not always the case. Here. But she doesn't take my problems seriously. Well, yeah, she doesn't take my problems seriously. Yes, they don't take my problems seriously. That is, if I say - I’m tired, I

worked, they're like - why are you tired, what problems can you have at 18 years old? Yes, it's true that there are none. Here. Well, in short, not being perceived as an adult also depresses me. Here. (...) So, it's very difficult now all at once, in fact, the relationship with parents is quite complicated, that's it. And now I've simply protected myself from them. When they are at home, I practically don't leave my room. Now my mother is offended by me, and I gave up, I don't care what she was offended by, I don't even want to find out, let her really be offended, if she doesn't want to enter into dialogue, it's her problem. Somewhere in the middle of the tenth grade, I tried with her, no, or at the beginning of the tenth grade, I tried to talk and say that I need support, at least just come and say that you're great, you can handle it, you'll succeed. And that's it, I don't need anything else. And this dialogue boiled down to the fact that I'm ungrateful, they spend a lot of money on me, they spend almost their last money on me, and I'm ungrateful. Then I got sick of them saying that they were spending a lot of money on me, so in April I went to work. That is, school and work were certainly difficult, but I managed. And since April, I haven't asked them for any money, I've bought clothes and something to eat here, well, I've gone to the neighboring town, all with my own money. And then they say something like this to me - why don't you go to the village with us, like you're at work again, you're cutting yourself off from us. Are we not giving you enough money? Something like that. Well, well, yes, I don't have enough money. So I went to work. Here. (..) In general, they are (..) children. M. Children? D. I can honestly say, they are not trying to change themselves, they are not trying to hear (..) me, that's it. It's quite difficult. M. Yes, I'm very sorry, what you're saying is very sad to listen to. And it's even so paradoxical, that is, well, it's clear that you're trying very hard and learning, and it's true, a lot of things are working out, and so, when they tell you that you're studying too much, and maybe you need to stop and go do something else, yes, I think it's very disappointing to hear. D. Yes, and when I wrote the Olympiad poorly and walked around upset, they told me that I just didn't study enough. Here. Something like this... M. Well, yes, in such conditions, of course, it is difficult to get support. Apparently we really need to look for it somewhere else. Please tell me, do you like being so independent, do you want to be more independent, or do you want to share this responsibility with someone? D. I began to notice that... Apparently I don't know at what level this works, but here... (..) somewhere inside I am still a child and in most situations, sometimes stressful, I begin to behave like a child. And then I, well, kind of think about it and... Well, I analyze it, and I understand that (.) did not behave like an adult in this situation. But it was necessary. Here. Well, in short, this independence from childhood, it actually affects me. Because even more so sports, they also tried like professional sports, all the work, and there, well (..) no study, no personal life, only sports. In short, this is it. You also need to be responsible. And now sometimes it takes its toll. Here.
M. What kind of sport? D. Orienteering. M. Wow. D. I also took up skiing. M. You can do a lot of things, it seems. D. Yes. M. Does this sport experience help you now? Even though you left him forcedly. D. Yes, it helps in the sense that... Well, on the one hand, no, I mean, wait, does it help that I left him, or the fact that I was studying? M. No, no, what did you learn exactly, what were you there and what did you do? D. What I endure until the last moment is a minus. So, let's say I get sick, and I won't go anywhere until the last minute, I'll endure it. I'm working until the end, I'm already feeling bad, I'll still do something, because I need to go to the gym... Or I just went to the gym recently, and I'm thinking like this - I'll do a light workout, because after my illness. But in the end I did it very hard, because somewhere in the middle of the workout I thought that I had worked very little, and that I had not finished myself off, and I need to finish myself off more, that's it. Well, (..) no, sport is just that, I don't know, I made friends because of sport. Here. (...) And in general, orienteering is a cool sport, it makes you think. And in general, the fact that you are running through the forest, I love this sport, in fact, very much, with my soul. Well, but as a

hobby, not as a sport. Sport, it destroys people. Professional. And health, both mental and physical. Everything possible.
And then, in short, school ended, I went to the school playground for a month, then on July 1, my grandmother and I went to the dentist, that is, also with my grandmother.
And on July 2, they left me alone. And in short, I went for a walk with my friend from the entrance. I really loved swinging on the horizontal bar, letting go and grabbing. In general, I once thought - like, how long, how long can I stay in the air without getting caught? Well, I fell on my face. And in general, it was a very cool day, so eventful, I rode in an ambulance, and then I probably walked around for a very long time with a blue face. Well, that's how my independent life began. (laughter) M. Yeah, what do you think this story says about you? How does this story characterize you? How do you think? How do you think about her and about you now? D. I don't know, it's just funny to me. M. But then it probably hurt a lot? D. No, by the way, I don't remember being in pain at all, I just remember that I fell and lay there for a while, then I thought that I needed to get up, and I felt discomfort in the area of my face, I wiped it and saw blood on my hands. I think I'll go home and wash myself. And in our case, in short, then they were repairing the roof, and there were guys standing at the entrance, they saw me and they lifted me up to the fourth floor, called an ambulance, and called my mother. Now, if they weren't there, what would have happened? Or if these men turned out to be inadequate, what would have happened? Interest Ask. But everything turned out well actually, no concussions, nothing, I didn't break my nose. M. Yes, but what did the parents and grandmother say when they came home and found out all this? D. Well, grandma was at home, mom came running, scared of course. Some guys called her and said that your daughter is covered in blood, that's it. M. Nightmare. And on July 3rd they left you alone again? D. I don't remember anymore. Maybe. Mom works, dad works, and of course. Well, sometimes my grandmother went to the garden, probably... I don't remember. But I'm still living, so it's okay. (laughter)
And, by the way, I started going to doctors alone very early. Here. At first it was very scary, very unpleasant, we have a very inadequate woman at the site, a local pediatrician, and before she could bring me straight to tears, so. Now I just come with a straight face, immediately dissatisfied and angry, so that she feels my bad aura (laughter) and does not start putting pressure on me. (laughter) In short, I begin to put pressure on her first. Here. And everything seems to be fine. Of course, it's still sometimes scary to go to doctors, I don't really like hospitals, that's why. Well, yes. As I already said, sometimes I endure until the last minute and don't go when I need to, on time, that's it. This is of course a minus.
M. Did you end up in the hospital because of this? D. (..) No. But... Well, yes, I was at Sirius this summer, and in short I was slightly ill, I had a sore throat and a runny nose. Well, I didn't go right away, but I went to the first-aid post, they prescribed antibiotics, I took it for 4 days, that's all, I didn't go to the first-aid post and then say that everything was fine with me. I just didn't want to go there anymore, that's it. And in the end, after 2 days I felt very bad, I could not tilt my head, my sinuses hurt, everything was wrong, I was afraid that I had sinusitis. I had sinusitis, that's it. And I decided to go. They scolded me very much there, they said that this was impossible, they took me to the hospital, in the end they wanted to admit me, but everything worked out, and in the end I was on antibiotics for another two weeks. That is, half of the entire shift that I was there, I was on antibiotics. Now I have stomach problems because of this. And all because I didn't go and tell him again that everything didn't work out for me. Here.
M. Is someone else watching him, do they remind you? Or, well, just... D. Well, they remind me, but they don't make sure that I'm taken right by the hand to the hospital, because after all, I'm already 17 years old, I'm not a child. Here. And even more so, there was such a situation that since childhood I've been in hospitals, I don't know, I've had a lot of different things, I also have

tuberculosis, some kind of problem, that is, that I have this mantu and Diaskintest are always big, something was stupidly infected there or something like that. She was registered. Then a cardiac surgeon, oh, a surgeon, (laughter) we also went to a cardiologist, I had problems when I started playing sports normally, in the fourth grade. Then, when I started playing sports even better, in the seventh grade, I walked like a disabled person because of my knees, and we also went to another city. Well, in short, a lot of things. And this is not a complete list. That’s why I’m very tired of this, I don’t really like going to the doctors and wouldn’t like to at all, but somehow I wasn’t particularly lucky with my health, to be honest.
M. Thank you very much. Please tell me, you are in Rostov-on-Don now, right? And I understand that you are working, while we were agreeing, you wrote that you need to catch up after work, are you studying or just working, how is your life in general now? L. Yes, then I will answer in such a multi-layered way, I received my first education this year, a bachelor’s degree, at DSTU University. In the Don State. Here. That's where I work. Here. It’s like we’ve even gone a little towards individualization, here. I’m studying, I’ve now entered two master’s programs, the first is my 39th, this is social work as well, and the second, 44/2, is in psychology and pedagogy. M. Ah, colleague that is. It's clear. Is the bachelor's degree also in psychology? L. Bachelor's degree 39, this is social work. M. Social work. L. Yes. I became interested in psychology in the second year, in the third, the therapeutic direction was closer, I studied there and realized that I also wanted to take two master’s degrees and one of them first. M. So you will study in both at once? L. Yes. That's what I thought... M. Wow. And work a little more? L. Yes, and in different universities. Well, that is, in my 35th, also in DSTU, and I went to study psychology at the Southern Federal. M. So, the Southern Federal is Krasnodar? L. No, this is the Southern Federal District, this is also Rostov. M. Oh, I got it mixed up, yes. It's clear. Do you have any other hobbies or interests, if you have time for them? L. From a hobby, if it’s an activity, yes, I really like to organize and come up with all sorts of different thematic events, fortunately, the university also loves when it is offered to do something, I probably won’t name any right now, psychological hobbies, hobbies, but for me I like some practices, well, for which I am conditionally admitted, I mean going through, conducting training, of course that’s it. I wouldn’t call my work a hobby, although I enjoy it.
M. Can you think of, remember some example, from there, I don’t know, from a movie, from a TV series, for example, I don’t know, from another work, from a book, of a person who is independent and not independent? Maybe some celebrities, someone famous, or some bloggers, someone in plain sight? L. So, (...) I don’t know directly whether this can be such an example, that is, interpret... (..) And in the work, well, Bukowski, there is Henry Chanaski, as his alter ego, the work “ Women" is called, "Post Office" too, so, I remember the main character, in my opinion he is very infantile, he also lives the life that he wanted there, he is rather an example of this, he works there at the post office, with a huge number of sexual relations, beer, and it’s as if he has nothing else in life. He didn’t escape anywhere, and he didn’t have to do anything. And if he’s just so independent... (...) What’s his name, I watched some interview today, I don’t know, I remember, Mark Guarden, I think his name is. Something like that. March... Some psychologist, I listened to his podcast today. Some kind of person, I don’t know... (...) I can give examples, so I look, I don’t know, the rector at my university is an independent person, but I don’t know (npt) work or not.
M. A person who enrolled in two master’s programs and built his entire professional trajectory? L. Yes, it still is, I have absolutely such a sober argument for this, when I had the opportunity to initially enroll, I was interested, I liked social work, because I read disciplinary work (NPT), I liked the number of courses, and I looked at psychology, psycho-ped. But biology was a mandatory part there. And I thought that I had made up my mind that I didn’t want to give it up. (laughter) And that’s it, here I am, although I could force myself to do it. But she didn't. Therefore, it doesn’t reach

ten yet. I... If only I had just defined myself vectorially, yes. Although I don't regret a second, I like everything that happened, but I still think that I screwed up a little there.
M. Did something happen then? L. Yes, yes, yes. And there... She fixed herself in this position. M. Well, somehow you can ask the question - what? Or don't want to answer you? L. It's possible, it's possible. I worked through my injuries, you can ask. (laughter) In my family, well, like in a family, there was a stepfather, a case of domestic violence that lasted for 6 years, from 10 to 16. Here. And it was very difficult to fight back, I was little, then I grew a little, so. And at the final point, well, at the age of 16, I immediately understood that I had to do something, somehow fight for myself, well, yes. And, thank God, it worked out, as they say. Yes. And at that moment I immediately realized that oh, oh, oh how you have to be independent (xxxx). (laughter) Oh, oh, oh, how necessary this is. And right then it was clearly formulated and it didn't go back from that point, thank God. M. But you moved after 3 years, as far as I understand? L. Have you moved? M. No? L. But no, just, well, it turns out that I have my dad, wonderful, beloved, but we (npt) when I was 7. Then I had a stepfather, from 10 to 16, not a very good person, (laughter) Here. Now my mother is also married, but now I have a wonderful stepfather, I respect him very much, I love him, I appreciate him, he is kind, caring and, well, my dad too. Wonderful person. I don't know what happened to that person. And I already moved when my mother was... I already had, now it turns out, a current stepfather, I already moved away from them. I didn't have it there, but everything was there (npt) it all ended just then (..) then, my last trial was at 16 with him. That's all. So we (..) said goodbye and... And I directly cemented my position as an independent lady. M. I'm very sorry about domestic violence, of course, and I'm glad that you and your mother are no longer there. If I hear correctly. L. No. M. And you came up with a plan? L. I'm now... Now I'm (npt) taking pictures of the experience, it was yes, thank you, I might not have been so strong. Who knows.
And after sixteen, yes, the first independent decision I made, I remember, it was on nerves, consciously, I don't know why, why, it didn't happen, I lit a cigarette. I came straight away, and I didn't have a situation where I was wandering around somewhere, in garages, I came openly and said, like this. Here. Naturally, my mother didn't like this, of course, but due to the traumatic experience there, she somehow turned a blind eye to it then. There she said - I'm sure it will fall off you, but now, okay. Well, we'll sort of assume that these aren't some kind of steam locomotives, it's not necessary. She is the only, it seems to me, the wisest thing that she told me, she says - please, you can, just don't buy some crap for yourself there, it's better to let them lie there at home, if you want, take it, like. Well, this is something normal. Because again, she says - what's the point, well, I would take it away from you, shout at you, so that you would stop smoking? Nothing would change. I would (npt) where is it, well, I would be hiding somewhere. No, and it fell off. I don't remember, I went there for two years, I worked through the traumatic experience with a psychologist, and one day I just said, no, I don't need this anymore. I wasn't so drawn to it when it was like this - well, you want it, right? Please. How (npt) is it normal, let's not just somewhere, over there at home on the balcony, you want, well, that's it, so that I can be calm.
M. That is, after you, I don't even know how to describe it, when you broke off relations with this man who showed domestic violence, with your stepfather, when he disappeared from the family, as I understand it, or he was imprisoned, I don't know. You had some kind of surge in all sorts of independent decisions and some kind of activity, right? L. Yes, well, yes, but rather it was probably, I don't even know how to classify it. Well, because yes, then it turns out that for 6 years you seem to live in a social cage anyway, there you don't think about how you can choose or think about something, but I don't know how it is conventionally, what jacket to wear today, so that there are no visible bruises. Anyway, this stupid childish trait - I won't show it, it lived, here. And this was the only thing in which you made (laughter) any independent decisions. Previously, you already go into some kind of, it seems to me, even aggression, in relation to those decisions in

which you were limited, and you are already trying to grab everything that you couldn’t take there before. Well, here I need to give credit, again, probably to my mother, but she somehow directed it all correctly, what are these, well, that is, my kind of eccentric, some kind of emotional outbursts, I don’t know, or what - radical decisions, like, I don’t know, smoking the same thing, were not met with aggression on her part, which would make me even more willing to do something against. She manipulated it all so much that you were like, so what. And that’s all there is to it, and that’s okay. Yes, well, thank you, okay. Fine. Fine. Well, then, but I remember, yes, she gave... Well, the start date, oh, now, as if I didn’t tear off my charger. I'll put it in. But before the start of, yes, my first year, it turns out that the first year and everything negative fell away from me.
M. Are there some things that you prefer to hide from your parents, maybe you don’t tell them from everyone? You mentioned tattoos, but that was quite a long time ago. L. Yes, well, what can I say, now I wouldn’t hide it, now I would come proudly (npt), to my mother and say - here it is, new. I just don't want to. I guess sometimes I can hide some of my emotional anxieties from my mother, because in general I’m such an emotional person, I can be sad, or just emotional... The only thing is, I try really hard, but I never succeed straight hide it from my mother, when I catch a cold there, or something like that, in short, it happens, I know that she will be nervous, and I don’t like it so much, and I’m always trying to do something, somehow - yes, everything is fine , Fine. (laughter) These are things, yes, I try not to tell my parents, because they will worry, well, that is, I don’t know, ordinary ARVI, but mom already - oh God, you live there alone, who will give you tea? do? Now, well, that is, these are not some kind of terrible things, I just (npt) don’t want (npt) only dad. No one will lose anything from this, I just don’t know, they will save 1000 nerve cells by doing this (npt). Without thinking about the tea I made for myself or not.
